# Atmospheric CO₂-to-acetaldehyde artificial photosynthesis in metallo hydrogen-bonded organic frameworks

**DOI:** 10.1038/s41467-026-75384-z

**Published:** 2026-07-12

**Authors:** Jun Pang, Xinyu Bai, Junjie Dai, Sijie Liu, Shuangfeng Yin, Yong Pei, Rong Tan

**Affiliations:** 1 National & Local Joint Engineering Laboratory for New Petro-Chemical Materials and Fine Utilization of Resources, Hunan Normal University, Changsha, Hunan PR China; 2 Key Laboratory of Chemical Biology and Traditional Chinese Medicine Research (Ministry of Education), Hunan Normal University, Changsha, Hunan PR China; 3 College of Chemistry, Xiangtan University, Xiangtan, Hunan PR China; 4 School of Chemistry and Chemical Engineering, Hunan University of Science and Technology, Xiangtan, Hunan PR China

**Keywords:** Sustainability, Photocatalysis

## Abstract

Artificial photosynthesis of acetaldehyde from atmospheric CO_2_ is highly promising, yet remains severely limited by low CO_2_ concentration in air and sluggish proton transfer kinetics. Herein, we report metallo hydrogen-bonded organic frameworks (MHOFs) of HNNU-X (X = O, S, Se) which enable acetaldehyde synthesis directly from air, water, and natural sunlight. Among them, HNNU-Se exhibits a competitive acetaldehyde production rate of 557.1 μmol g⁻¹ h⁻¹ with high electron-based selectivity of 95% under outdoor conditions, without need of sacrificial agents. Mechanistic studies reveal that HNNU-X creates a proton-rich microenvironment in which [Zn(tpy)]^2+^ cations function as CO_2_ capture and activation sites for direct air capture, while [XCN]^–^ anions serve as proton shuttles, facilitating rapid transfer of in-situ generated H⁺ from solar-driven water oxidation to adsorbed CO_2_. This work enables the synergistic coupling of CO_2_ reduction and H_2_O oxidation, thereby achieving efficient artificial photosynthesis of acetaldehyde.

## Abstract (web summary)

This study develops metallo hydrogen-bonded organic frameworks (HNNU-X), which overcome the low CO_2_ concentration in air and sluggish proton transfer kinetics, achieving artificial photosynthesis of acetaldehyde from air, water and natural sunlight.

## Introduction

**Fig. 1:**
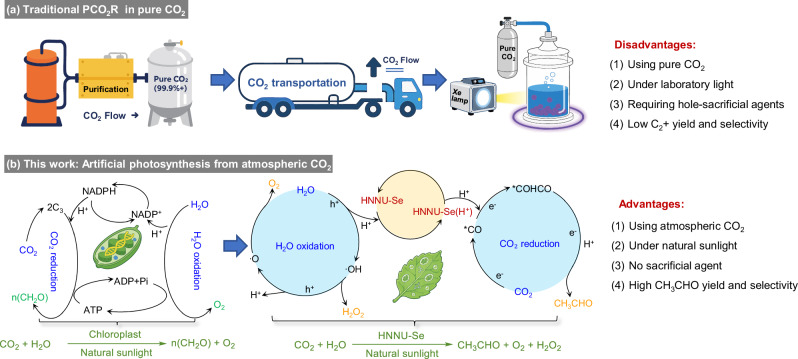
Schematic illustrating the photocatalytic CO_2_ reduction (PCO_2_R) process. **a** Schematic diagrams of traditional PCO_2_R reaction (Disadvantages: highly pure CO_2_ as CO_2_ source, laboratory light as energy source, required hole-sacrificial agents, high energy costs for CO_2_ purification, low C_2_+ yield and selectivity). **b** Artificial photosynthesis of acetaldehyde from atmospheric CO_2_ in this work (Advantages: atmospheric air as CO_2_ source, natural sunlight as energy source, no sacrificial agent required, simplified experimental equipment, high CH_3_CHO yield and selectivity).

Acetaldehyde (CH_3_CHO) is a key industrial feedstock, with global demand projected to reach ~1 million tons by 2027^1^. Solar-driven artificial photosynthesis^2^ of acetaldehyde from air and water offers a compelling alternative to conventional processes, including strong acid-depended ethylene oxidation^3,4^ and energy-intensive ethanol oxidation^5^. Apart from its synthetic value, this transformation provides a sustainable route toward a closed carbon cycle and mitigate global warming. Recent advances in photocatalysis, such as locally crystallized carbon nitride^6^ or Cd_0.6_Zn_0.4_S co-modified with sulfur vacancies and Cu sites^7^, have enabled CO_2_-to-CH_3_CHO conversion via proton-coupled electron transfer pathways. However, these systems rely on purified CO_2_ feedstocks, incurring significant energy costs associated with CO₂ separation and purification (Fig. 1a). Direct utilization of atmospheric CO_2_ is therefore highly desirable, yet remains challenging due to its low concentration (~400 ppm)^8,9^ and the competing reduction of O₂ under ambient conditions^10^.

In nature, plants overcome this limitation through specialized CO_2_-fixation enzymes that concentrate CO_2_ up to ~1000-fold near catalytic sites^11^. The enriched CO_2_ is subsequently converted into carbohydrates during photosynthesis. Chemists have integrated CO_2_-affinity sites (e.g., amine, pyrimidines, triazines, and azoles) into metal-organic frameworks for CO_2_ capture^12,13^. These frameworks exhibit high CO_2_ adsorption capacity and especially, preferential CO_2_ adsorption over N_2_ and O_2_ molecules. However, most CO_2_ absorbents effectively only under dry conditions, as water with large dipole moment binds more strongly to the framework than CO_2_^14,15^. Hydrophobization of certain absorbents has been explored to suppress competitive water adsorption under humid conditions^16^, but this approach is incompatible with subsequent aqueous phase CO_2_ photoreduction. The development of single-component photocatalysts that integrate atmospheric CO_2_ capture and H_2_O adsorption would provide a significant advancement toward artificial photosynthesis for acetaldehyde production.

Hydrogen-bonded organic frameworks (HOFs) are an emerging class of crystalline porous materials assembled by organic or metal-organic molecules via hydrogen bonding with the assistance of other noncovalent interactions, such as π-π stacking interactions and van der Waals forces^17,18^. Their structural modularity enables the integration of multiple functionalities within a single framework^19,20^. In particular, HOFs typically exhibit high hydrophilicity, facilitating water uptake in aqueous environments^21^. If we incorporate the CO_2_ affinity sites and photosensitizer in the network of HOFs, the customized H-bonded matrices should achieve a balance between atmospheric CO_2_ capture and H_2_O adsorption, meeting the prerequisite for artificial photosynthesis of acetaldehyde from CO_2_ and H_2_O. However, the 10e^-^/10H^+^ reduction of CO_2_ to CH_3_CHO confronts the further challenge of suppressing the more kinetically favorable hydrogen evolution reaction (HER) which is a competing 2e^-^/2H^+^ process^22^. Natural photosynthesis addresses this challenge by reducing CO_2_ with hydrogen (H^+^) in the form of NADPH/H^+^ arisen from solar water splitting (Fig. 1b)^23^. Mimicking this strategy requires the introduction of proton-relay pathways proximal to catalytic centers to efficiently shuttle H⁺ and promote CO₂ reduction.

Zinc(II) terpyridyl ([Zn(tpy)]^2+^) complexes are well-established photosensitizers, owing to their strong light-harvesting capability and efficient electron transfer^24^. The terpyridyl ligand can also serve as a CO_2_-binding site, enriching CO_2_ near the catalytic center^24,25^. Meanwhile, conjugated pseudohalogen anions of [XCN]^–^ (X = O, S, Se) have demonstrated effective proton-relay behavior in electrochemical CO₂ reduction to HCOOH^26^. Based on the above reasoning, we here employ [Zn(tpy)][XCN]_2_ (X = O, S, Se) complexes as building blocks to construct bioinspired MHOFs (denoted HNNU-X) for artificial photosynthesis of acetaldehyde from atmospheric CO_2_. Single-crystal X-ray diffraction (XRD) reveals well-ordered assembly via cooperative hydrogen bonding and π–π interactions. The resulting HNNU-X (X = O, S, Se), especially, HNNU-Se, exhibit strong CO_2_ binding affinity and selectivity (CO_2_/O_2_ = 54:1), broad-spectrum light absorption (visible light full region), high proton conductivity (3.23 × 10^−6^ S cm^−1^ at 298 K), and extended exciton lifetime (τ = 2.44 ns). As expected, HNNU-Se delivers high CH_3_CHO production rate of 557.1 μmol g⁻¹ h⁻¹ with electron-based selectivity of 95% under natural sunlight in air without sacrificial agents. The performance is competitive in CO_2_ photoreduction field, although most systems operate under pure CO_2_ atmosphere and visible light illumination. Combined experimental and theoretical studies reveal that the customized MHOFs architectures function as “proton pump”, enabling rapid transfer of in situ generated H⁺ from water oxidation to adsorbed CO₂. This “proton shuttling” behavior promotes the synergistic coupling of CO₂ reduction and H₂O oxidation, thereby accelerating overall reaction kinetics (Fig. 1b). Notably, the dynamic hydrogen-bonding network endows HNNU-X with self-healing capability via recrystallization, allowing at least seven catalytic cycles without significant performance loss. This artificial photosynthesis system depicts a dream-full blueprint that, using only sunlight, air, and water as the starting materials, CH_3_CHO can be continuously generated at catalyst surface without any other energy input. It paves the most green, sustainable, low-cost, and energy-saving route for acetaldehyde manufacture.

## Results and discussion

### Preparation and structural characterization of catalysts

**Fig. 2:**
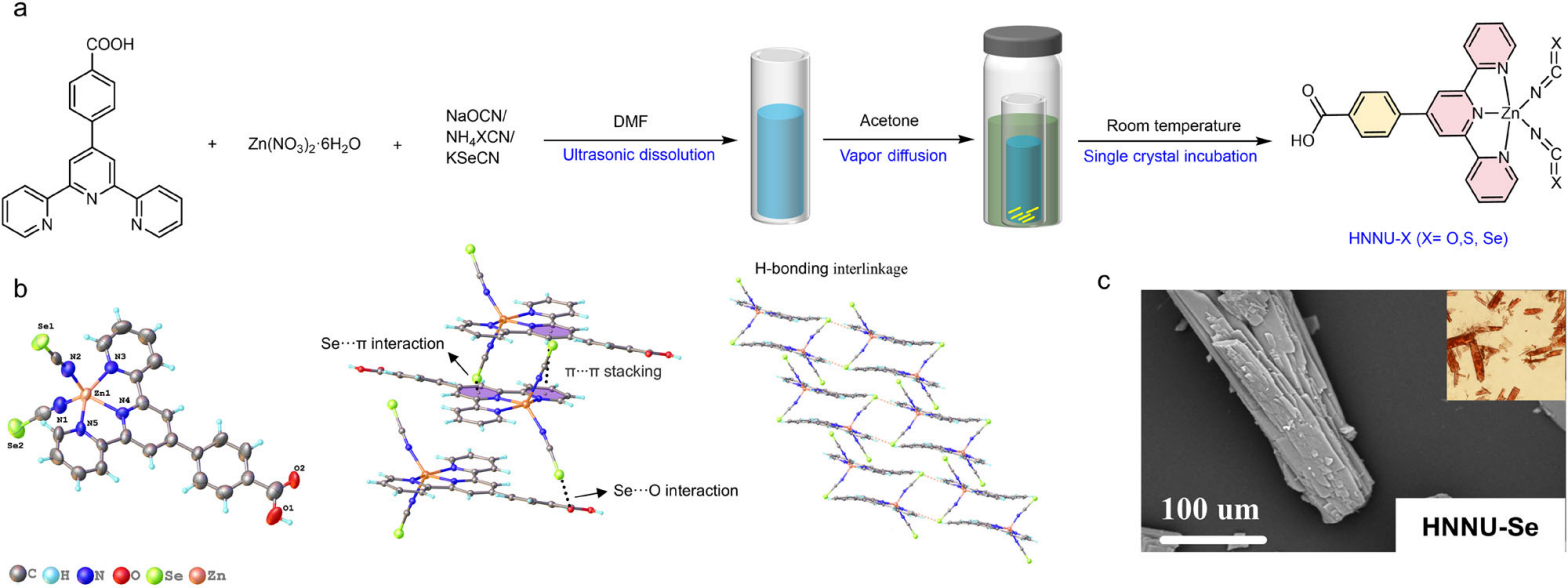
Preparation and X-ray crystallographic views of HNNU-X. **a** Synthesis of HNNU-X (X = O, S, Se) (cyan cylinder: DMF, green cylinder: acetone). **b** Single-crystal X-ray diffraction view of the coordination mode of building blocks, the nodes, and the topologies of HNNU-Se (gray C, cyan H, red O, blue N, pale green Se, orange Zn). **c** SEM image of HNNU-Se (inset is the single crystal image).

HNNU-X (X = O, S, Se) frameworks were synthesized via coordination of 4-([2,2′:6′,2′′-terpyridin]−4’-yl)benzoic acid (TPBA) with Zn(NO_3_)_2_ ⋅ 6H_2_O in a DMF/H_2_O mixed solvent system containing different pseudohalide salts (NaOCN for HNNU-O, NH_4_SCN for HNNU-S, KSeCN for HNNU-Se). The resulting precipitates were subsequently redissolved in DMF and subjected to slow vapor diffusion of acetone, yielding yellow single crystals suitable for X-ray diffraction (Fig. 2a). Detailed synthetic procedures are given in Section S1 in Supporting Information. Single-crystal X-ray diffraction revealed that HNNU-O (CCDC number: 2441865) crystallizes in the monoclinic *C2/c* space group, whereas HNNU-S (CCDC number: 2441866) and HNNU-Se (CCDC number: 2441870) adopt triclinic *P̅−1* symmetry (Supplementary Table 1).

All three frameworks are constructed from distorted trigonal bipyramidal [Zn(tpy)](XCN)_2_ units, in which two pseudohalide anions ([XCN]⁻, X = O, S, Se) coordinate to Zn²⁺ centers (Fig. 2b and Supplementary Fig. 3). In HNNU-O, the [Zn(tpy)](OCN)_2_ units assemble into supramolecular ribbon chains through π–π stacking interactions (Fig. 2b). In HNNU-S and HNNU-Se, the presence of σ-holes on S and Se enables additional directional interactions^27^, including chalcogen···π (with aromatic rings) and chalcogen···O (with carboxyl groups)^28^, which cooperate with π–π stacking to stabilize the ribbon motifs (Fig. 2b and Supplementary Fig. 3f). These one-dimensional chains are further interconnected through multiple hydrogen-bonding interactions, giving rise to long-range ordered three-dimensional architectures of HNNU-X (Fig. 2b and Supplementary Figs. 3c and g). Powder X-ray diffraction (PXRD) patterns of HNNU-X match well with the corresponding simulated patterns (Supplementary Fig. 4), confirming high crystallinity conducive to charge transport. Scanning electron microscopy (SEM) reveals their rod-like morphologies composed of stacked nanosheets (Fig. 2c and Supplementary Figs. 3d and h). Energy dispersive X-ray spectroscopy (EDS) mapping images demonstrates homogeneous distribution of chalcogen elements (O, S, Se) across the corresponding HNNU-X (Supplementary Fig. 5), supporting their structural integrity.

### Selective CO_2_ capture and H_2_O adsorption

**Fig. 3:**
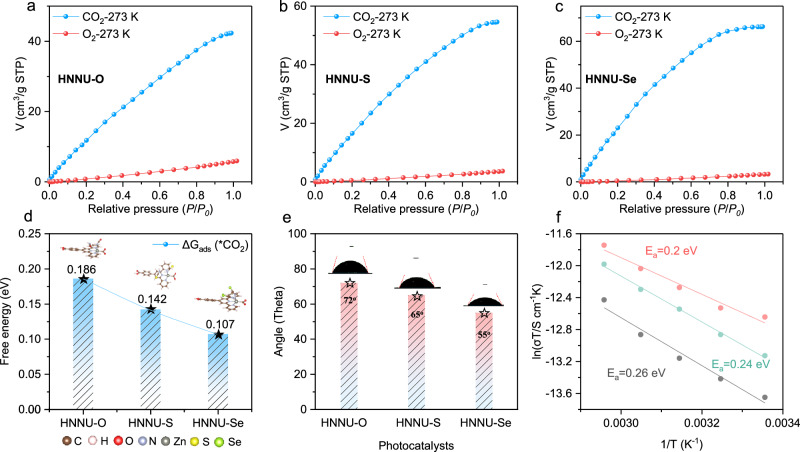
Selective CO_2_ capture and H_2_O adsorption. **a–c** The selective CO_2_/O_2_ adsorption studies for HNNU-O, HNNU-S, and HNNU-Se at 273 K. **d** Comparison of the calculated adsorption free energies of CO_2_ on HNNU-O, HNNU-S, and HNNU-S (insets are the corresponding CO_2_ adsorption models). **e** WCA values of HNNU-X (insets are the corresponding WCA images). **f** The corresponding Arrhenius plots of HNNU-X in proton conductivities.

Photocatalytic reduction of CO_2_ in air requires preferential CO_2_ adsorption over O₂, which otherwise competes for photogenerated electrons to form superoxide radicals^29^. The adsorption properties of HNNU-X over CO_2_ and O_2_ were thus investigated, and the results were shown in Fig. 3a–c. Clearly, all HNNU-X materials exhibit strong CO_2_ affinity due to the multiple N atoms in terpyridyl ligand, which act as the lewis basic sites for CO_2_ adsorption. Among them, HNNU-Se displays the highest uptake (66.2 cm^3 ^g^−1^ at 273 K), exceeding HNNU-O (54.6 cm^3 ^g^−1^) and HNNU-S (42.3 cm^3 ^g^−1^). Density of states (DOS) analysis reveals an upshifted d-band center for for HNNU-Se (−9.36 eV) relative to HNNU-S (−9.41 eV) and HNNU-O (−9.48 eV) (Supplementary Fig. 6). The higher d-band center results in a stronger interaction between catalyst and adsorbed molecule, enhancing the CO₂ adsorption^30^. Furthermore, density functional theory (DFT) calculation confirm the thermodynamic preference for CO_2_ adsorption on HNNU-Se, demonstrating the lowest adsorption free energy among the HNNU-X (Fig. 3d). Compared to CO_2_ uptake, all HNNU-X exhibit negligible O₂ uptake (3.2–5.9 cm^3 ^g^−1^ at 273 K). Selective CO_2_ adsorption over O_2_ for these MHOF networks was calculated by initial adsorption slope ratios according to the Ideal Adsorption Solution Theory (Supplementary Fig. 7). High CO_2_/O_2_ selectivity ratio is observed over all HNNU-X at 273 K. In particular, HNNU-Se shows the highest CO_2_/O_2_ selectivity ratio of 54, whereas HNNU-S, and HNNU-O show selectivity ratios of 32 and 14, respectively. Breakthrough experiments further confirm the selective adsorption capacity of typical HNNU-Se for CO₂ in ternary N_2_/O_2_/CO_2_ mixture (70/20/10, V/V/V) (Supplementary Fig. 8). Owing to the highest CO_2_ binding affinities and specific selectivities, HNNU-Se should be very promising in the photoreduction of CO_2_ from air^31^.

Apart from the selective CO_2_ capture, water adsorption of HNNU-X is also critical for CO_2_ photoreduction. Water contact angle (WAC) value was used to evaluate the water affinity of HNNU-X (Fig. 3e). Clearly, all HNNU-X samples exhibit hydrophilic nature with the WCA values lower than 90^o^. The water affinity gradually improves from HNNU-O (WAC of 72°), HNNU-S (WAC of 65°) to HNNU-Se (WAC of 55°) probably due to the most effective solvation of SeCN^-^ anion. The higher water affinity implies that more water molecules adsorb on HNNU-Se surface, creating a proton-richer microenvironment for proton conduction. Electrochemical impedance spectroscop (EIS) reveals proton conductivities (σ) of 1.18 × 10^−6^, 1.99 × 10^−6^, and 3.23 × 10^−6^ S cm^−1^ for HNNU-O, HNNU-S and HNNU-Se, respectively, at 298 K and 99% relative humidity (Supplementary Fig. 9)^32^. These σ values increase with temperature, reaching 3.40 × 10^−6^, 6.25 × 10^−6^, 7.94 × 10^−6^ S cm^−1^ at 338 K for HNNU-O, HNNU-S and HNNU-Se, respectively. According to Arrhenius plots^33^, HNNU-Se shows the lowest activation energy (*E*_a_, 0.2 eV) for proton conduction than HNNU-S (*E*_a_, 0.24 eV) and HNNU-O (*E*_a_, 0.26 eV) (Fig. 3f). It implies that HNNU-Se effectively lowers the energy barrier for proton transport through optimized hydrogen-bonding networks, which should be favorable for the ten proton CO_2_-to-CH_3_CHO reduction pathway.

### Photoelectrochemical properties

**Fig. 4:**
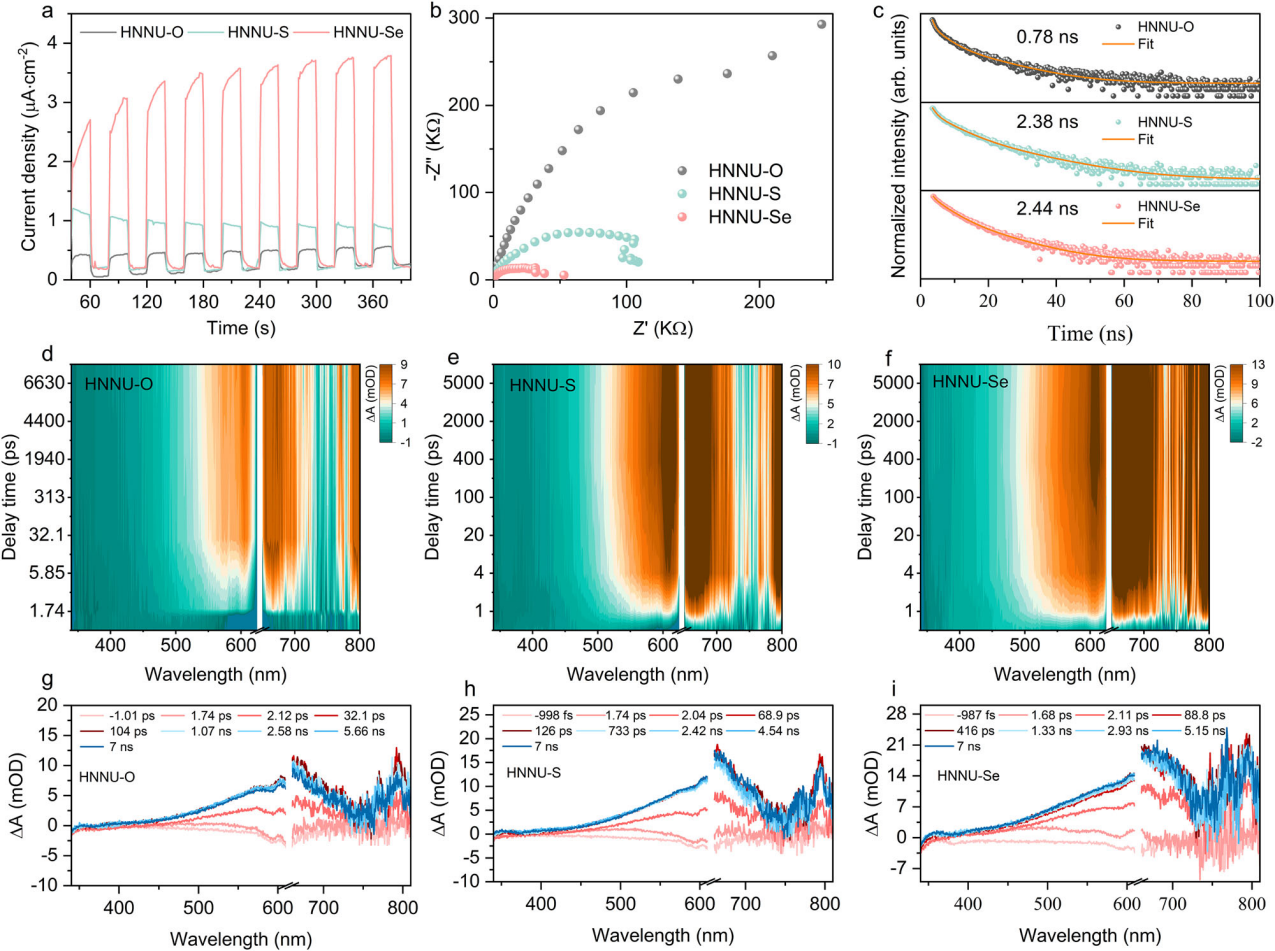
Photophysical properties and carrier dynamics of HNNU-X. **a** Photocurrent response, (**b**) EIS plots (measurements were performed at open-circuit potential over a frequency range from 0.05 Hz to 100 kHz with an AC amplitude of 5 mV in Na_2_SO_4_ aqueous electrolyte (0.5 M, 500 mL)). Potentials were not iR-corrected; the measured solution resistance was 25 ± 5 Ω), **c** time-resolved PL spectra (λ_ex_ = 440 nm) of HNNU-X. **d–i** Femtosecond transient absorption (fs-TA) spectroscopy observation of 3D pseudo color spectra and corresponding 2D ultrafast spectral signal of HNNU-X (Transient absorption spectroscopy (TAS) where 320 nm beam light was used as pump light and a continuous white light ranging from 340 to 800 nm was used as probe light).

Spectroscopic analyses were systematically conducted to investigate the photoelectrochemical properties of HNNU-X. Ultraviolet-visible diffuse reflectance spectra (UV-Vis DRS) of HNNU-X (Supplementary Fig. 11a) reveal the broad light absorption from UV to visible light region. In particular, HNNU-Se displays the strongest light absorption with the edge extended to near infrared light region. The high light-harvesting capability makes HNNU-Se a promising candidate for solar-driven photocatalytic applications. In addition, HNNU-Se shows the weakest charge recombination in photoluminescence (PL) spectra (Supplementary Fig. 12) and the strongest transient photocurrent in photocurrent response measurements (Fig. 4a), indicating the maximum charge separation efficiency among HNNU-X^34^. Electrochemical impedance spectroscopy (EIS) Nyquist plots demonstrate the minimum charge transfer resistances of HNNU-Se, indicating the fastest interfacial charge transfer in the composite (Fig. 4b). The average photogenerated carrier lifetimes (τ_n_) are measured by time-resolved PL (TRPL) (Fig. 4c). HNNU-Se shows the longest τ_n_ (2.44 ns), followed by HNNU-S (2.38 ns), and HNNU-O (0.78 ns), indicating the lowest recombination rate of charge carriers, as confirmed by PL emission spectra. These results demonstrated that the presence of SeCN^–^ anion promotes the separation and transfer of photogenerated carriers, while prevents them recombination.

Femtosecond transient absorption (fs-TA) spectroscopy provides further insight into charge carrier dynamics and separation/transport mechanisms in HNNU-X (X = O, S, Se) systems (Fig. 3d–i and Supplementary Fig. 13). Upon 320 nm excitation, all samples display a broad negative ground-state bleaching (GSB) signal (400-730 nm), manifesting the stimulation of charge from ground to excited state^35^. The charge excitation rapidly recovers within picoseconds (ps) before evolving into positive excited-state absorption (ESA) features (Fig. 4g–i). This spectral evolution suggests ligand-to-metal charge transfer (LMCT)-mediated charge separation processes. Notably, HNNU-Se exhibits the fastest GSB recovery (1.48 ps), compared to HNNU-S (1.68 ps) and HNNU-O (1.81 ps), reflecting the most rapid charge transfer. Double-exponential fitting of 602 nm decay profiles reveals two distinct relaxation pathways (Supplementary Fig. 13a–c). The fast component (τ_1_ < 0.5 ps) corresponds to exciton-exciton (hole and electron) annihilation, and the slower process (τ_2_, 9.57-49.5 ps) may be attributed to either electron–hole recombination or electron trapping. Given that the decay lifetime (τ_2_, 9.57-49.5 ps) is much shorter than nanosecond-scale electron lifetime from TRPL results, the τ_2_ pathway can’t be assigned to electron–hole recombination in HNNU-X. Thus, we can attribute this process to electron trapping, which often occurs in organic semiconductors, especially in conjugated polymers^36^. Additional ESA signals in the 750–800 nm range exhibit nanosecond-scale quenching dynamics (1.74–3.9 ns) (Supplementary Fig. 13d–f), which should correspond to the recombination of electrons and holes. We noticed that the variation of XCN^-^ ion hardly affects the exciton-exciton annihilation process, but significantly modulates both electron trapping and recombination processes. The SeCN^-^ anion leads to the most efficient separation of photogenerated electron-hole pairs, which is evident from the highest local charge density (23.63×10^17 ^cm⁻³) in HNNU-Se (Supplementary Fig. 14). Furthermore, the strongest positive absorption (ΔA) of HNNU-Se further confirms its highest concentration of photogenerated electrons, which is critical for driving the ten-electron CO_2_-to-CH_3_CHO reduction pathway (Fig. 4g–i)^37^.

**Fig. 5:**
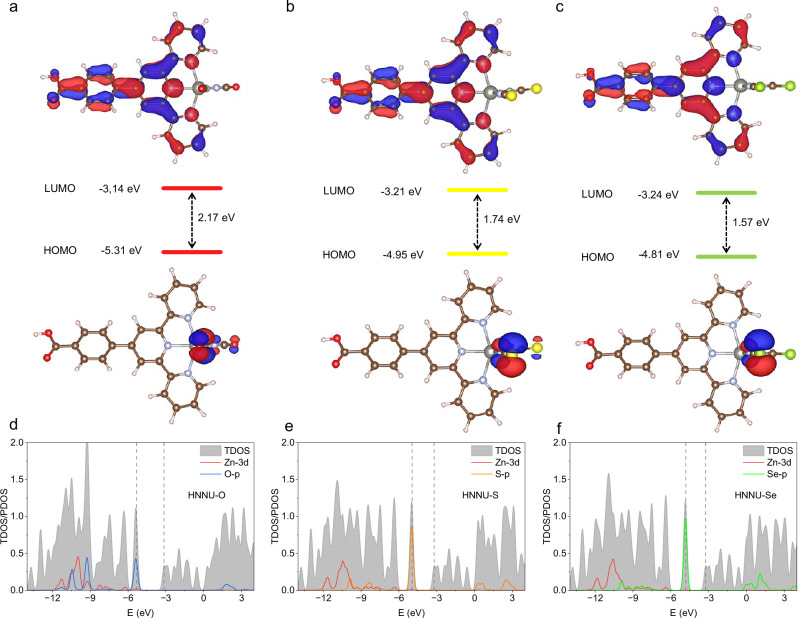
Band structure of HNNU-X. **a–c** HOMO and LUMO distributions of HNNU-X (X = O, S, Se) (the positive and negative isosurfaces represented in red and blue, carbon, hydrogen, oxygen, nitrogen, sulfur, selenium and zinc atom are depicted in brown, beige, red, light blue, yellow, green, and gray, respectively). **d–f** Density of states of HNNU-X (X = O, S, Se) (the dashed lines indicate the positions of the HOMO and LUMO levels).

To understand the role of XCN^-^ anions in modulating charge separation and transportation behavior, we conducted DFT calculations on the frontier molecular orbitals of HNNU-X building blocks. The orbital analysis reveals distinct spatial distributions of electron density: the lowest unoccupied molecular orbitals (LUMOs) predominantly localize on the [Zn(tpy)]²^+^ centers, while the highest occupied molecular orbitals (HOMOs) primarily reside on the coordinated XCN⁻ anions (Fig. 5a–c). This orbital configuration suggests an efficient electron transfer pathway from the XCN⁻ anions to [Zn(tpy)]²^+^ centers upon photoexcitation. Notably, the Se atom in HNNU-Se exhibits substantially greater HOMO contribution compared to its S and O counterparts in HNNU-S and HNNU-O, respectively (Fig. 5c vs. 5a, b). The enhanced orbital localization creates pronounced spatial separation between HOMO (Se atom) and LUMO (Tpy ligand) in HNNU-Se. As a result, HNNU-Se shows the narrowest HOMO-LUMO gap (1.57 eV) than HNNU-S (1.74 eV) and HNNU-O (2.17 eV). The narrower bad gap implies the less radiant energy required for photon excitation, which is beneficial for light absorption^38^. Density-of-states analysis shows increasing p-orbital contributions from O to S to Se near the Fermi level, with Se exhibiting the highest density of states (Fig. 5d–f). This enhanced p-orbital participation lowers the energy barrier for photon-induced electronic transitions, thereby enhancing light absorption efficiency and promoting charge separation in HNNU-Se. Actual energy band structures of HNNU-X were determined by UV-vis DRS and Mott-Schottky (M-S) plots (Supplementary Figs. 10 and 11). Notably, the *E*_*CB*_ positions of all HNNU-X are more negative than the potential for CO_2_/CHCHO (−0.36 eV), which alignment satisfies the thermodynamic prerequisites for photocatalytic CO_2_ reduction.

### Overall photocatalytic CO_2_ reduction with H_2_O oxidation

**Fig. 6:**
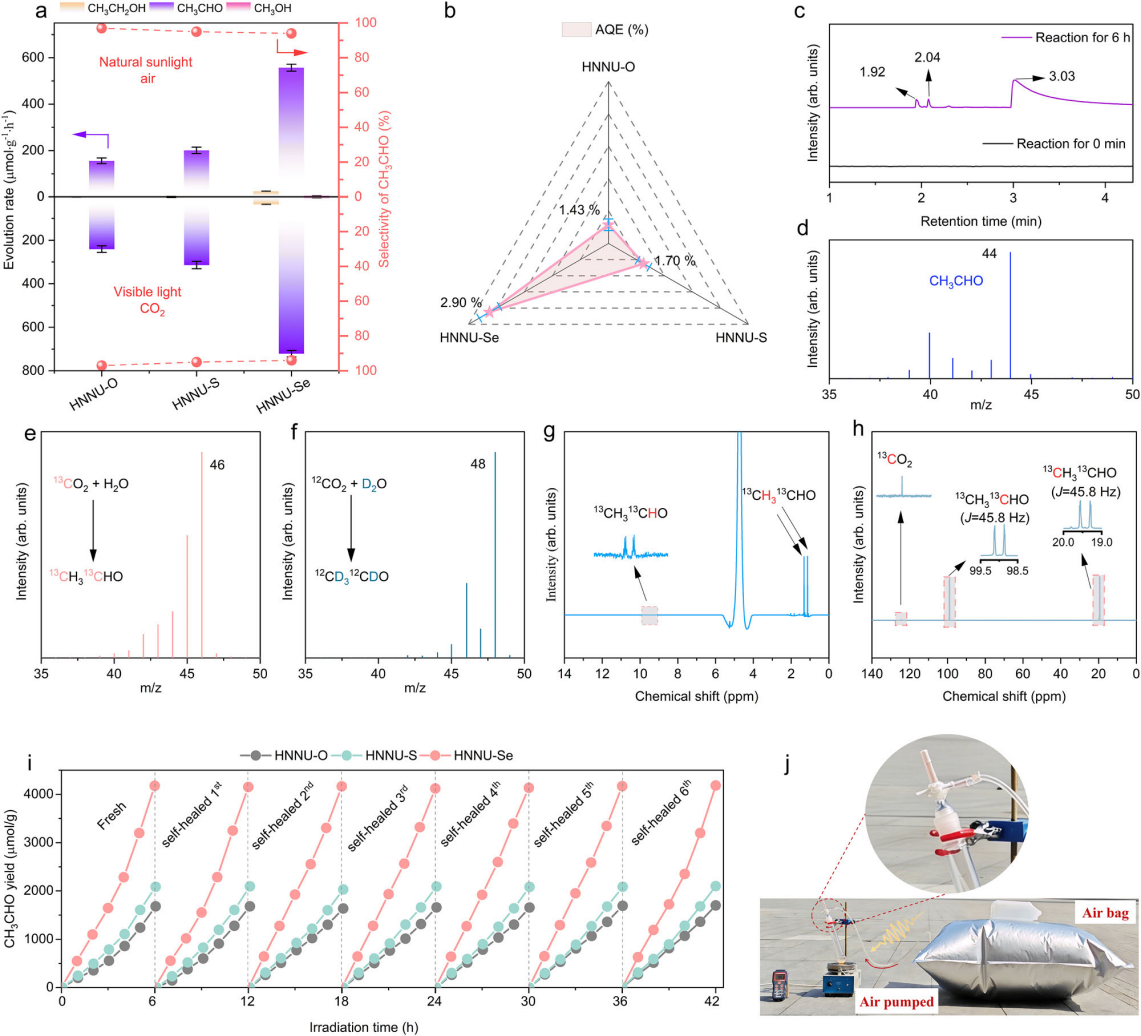
Overall photocatalytic CO_2_ reduction performance of HNNU-X. **a** Photocatalytic CO_2_ reduction activity of HNNU-X in air under natural sunlight irradiation for 6 h or in pure CO_2_ under 300 W Xe lamp irradiation (λ > 420 nm, 250 mW·cm^–2^) for 6 h (data are presented as mean ± s.d. from three independent measurements (*n* = 3). Error bars represent standard deviation). **b** The catalytic quantum efficiency (AQE) of HNNU-X in the presence of a bandpass filter of 450 nm. **c**,** d** GC-MS spectra of liquid product of photocatalytic CO_2_ reduction with H_2_O in air over HNNU-Se. **e** MS spectra of produced acetaldehyde from CO_2_ coupled with D_2_O, (**f**) MS spectra of produced acetaldehyde from ^13^CO_2_ coupled with H_2_O. **g** ^1^H NMR and **h** ^13^C NMR of the liquid products from ¹³CO₂ photoreduction over HNNU-Se. **i** Reusabil**i**ty of HNNU-X upon self-healing. **j** Photocatalytic CO_2_ reaction operates under air atmosphere and natural sunlight (the circle represents the partial enlarged detail of custom-built photocatalytic reactor). Reaction conditions (unless otherwise specified): catalyst (5 mg), deionized water (2.5 mL), air atmosphere, natural sunlight, reaction temperature (25 ^o^C).

Encouraged by the selective CO_2_ capture capability, high water affinity, sufficient light absorption, high proton conduction, and long exciton lifetimes, HNNU-X (X = O, S, Se) were employed in photoreduction of atmospheric CO_2_ with H_2_O under natural sunlight without sacrificial agents (Fig. 6a, b). As shown in Fig. 6a, all HNNU-X preferentially converted CO_2_ to CH_3_CHO in atmospheric air. The product was unambiguously identified by gas chromatograph-mass spectrometer (GC-MS) (Fig. 6c, d), with a dominant GC peak at 3.03 min and a molecular ion at m/z = 44, consistent with acetaldehyde. Trace amounts of C_2_H_5_OH and CH_3_OH were detected along with CH_3_CHO (^1^H NMR quantification in Supplementary Figs. 18–20). No product was acquired in the absence of photocatalyst, light, CO_2,_ or H_2_O, establishing that all the components were indispensable for this reaction (Supplementary Table 5). Isotope-labeling experiments were conducted to confirm that CH_3_CHO arises from CO₂ and H_2_O. The labeled products were determined by GC-MS analysis (Supplementary Fig. 21). When ^12^CO_2_ was used as carbon source, the main m/z peak is located at 44, assigning to the molecular weight of CH_3_CHO (Fig. 6c). The signal shifts to 48 with the use of D_2_O, verifying that the hydrogen in CH_3_CHO is derived from H_2_O (Fig. 6e). The appearance of +1 amu fragments in the MS is likely due to residual, pre-adsorbed H_2_O from ambient air, due to the high water affinity of HNNU-X. By replacing ^12^CO_2_ with ¹³CO₂, the peak at m/z = 44 shifts to 46, corresponding to ^13^CH_3_^13^CHO (Fig. 6f). Similarly, the presence of a minor m/z = 45 signal is attributed to partially labeled products (^13^CH_3_^12^CHO or ^12^CH_3_^13^CHO) arising from pre-adsorbed ^12^CO_2_ on catalyst surface. The protons of ¹³C-labeled CH₃CHO show ¹³C-induced splitting in ¹H NMR spectrum (Fig. 6g). ^13^C NMR spectrum of ^13^C-labeled CH_3_CHO (Fig. 6h) also shows characteristic isotope-induced splitting at ~19.4 and 98.9 ppm, attributable to the ¹³*C*H_3_ and ¹³*C*HO carbons in ^13^CH_3_¹³CHO, respectively. These results verified that CO_2_ serves as carbon source in such photocatalytic CO_2_ conversion. Actually, only CO_2_ and H_2_O were used without any other additives, which provided the greenest and cheapest process for acetaldehyde production.

The CH_3_CHO production rate strongly depends on the type of [XCN]^-^ anions in HNNU-X. Specifically, HNNU-Se achieved a competitive CH_3_CHO production rate of 557.1 ± 15.5 µmol g^−1^ h^−1^ with high selectivity (95%) under ambient conditions. This performance markedly outperformed HNNU-S (204.2 ± 13.6 µmol g^−1^ h^−1^) and HNNU-O (153.9 ± 13.1 µmol g^−1^ h^−1^), highlighting the critical role of chalcogen selection (Fig. 6a). The enhanced photocatalytic activity of HNNU-Se should arise from the synergistic combination of (1) stronger CO_2_ adsorption, (2) proton-enriched interfacial microenvironment, and (3) prolonged lifetime of photogenerated electron, which facilitates the multiple proton-coupled electron transfer process. Notably, the production rate (557.1 µmol g^−1^ h^−1^) over HNNU-Se in air is competitive in the field of photoreduction CO_2_ to acetaldehyde, although most photoreduction was performed in pure CO_2_ atmosphere under visible light illumination (Supplementary Table 4). The apparent quantum efficiency (AQE) at 450 nm reaches 1.43 ± 0.07%, 1.70% ± 0.09%, and 2.90 ± 0.15% for HNNU-O, HNNU-S, and HNNU-Se, respectively (Fig. 6b and Supplementary Table 3). HNNU-Se achieves 721.2 ± 18.2 μmol g⁻¹ h⁻¹ of CH_3_CHO production rate when the PCO_2_R operates under pure CO_2_ atmosphere and visible light illumination (Fig. 6a). Furthermore, the HNNU-Se has a competitive advantage over the state-of-art photocatalysts for CO_2_ reduction to other C_2_ products without the addition of sacrificial agent (Supplementary Table 4). Intriguingly, all HNNU-X exhibited just slightly reduced performance in ourdoor environment (air atmosphere and natural sunlight) compared to laboratory condition (CO_2_ atmosphere and visible light) (e.g., 557.1 vs.721.2 μmol g⁻¹ h⁻¹ for HNNU-Se) (Fig. 6a, j), which attributes to their broad light absorption and selective CO_2_ capture from air. No H_2_ evolution was detected in the system, ruling out the competing hydrogen evolution reaction (HER) during the reaction.

After reaction, all HNNU-X (X = O, S, Se) could be recovered from reaction system by centrifugation. Although partial loss of crystallinity occurred due to the intrinsic water sensitivity of HOFs, the frameworks could be self-healing by simple recrystallization (Supplementary Fig. 27) and maintain their high catalytic efficiency upon successive reuse. As shown in Fig. 6i, the self-healed HNNU-X (X = O, S, Se) could be reused for at least seven times without noticeable performance decay in the photocatalytic CO_2_ reduction. Indeed, no significant change in morphology of HNNU-X took place even after reusing seven times in water, as shown in SEM images (Supplementary Fig. 28). ICP-OES analysis gave Se content in self-healed HNNU-Se of 22.18 wt%, which was almost identical to that of the fresh one (22.23 wt%). Negligible amount of Se was detected in the supernatant according to ICP-OES analysis, ruling out the leaching loss of Se species in the aqueous photocatalytic CO_2_ reduction. The self-healing ability of HNNU-X is attributed to a reversible HOF rearrangement because of the dynamic nature of hydrogen bonding interactions^39^, which is practical for the large-scale industrial application.

### Reaction mechanism

**Fig. 7:**
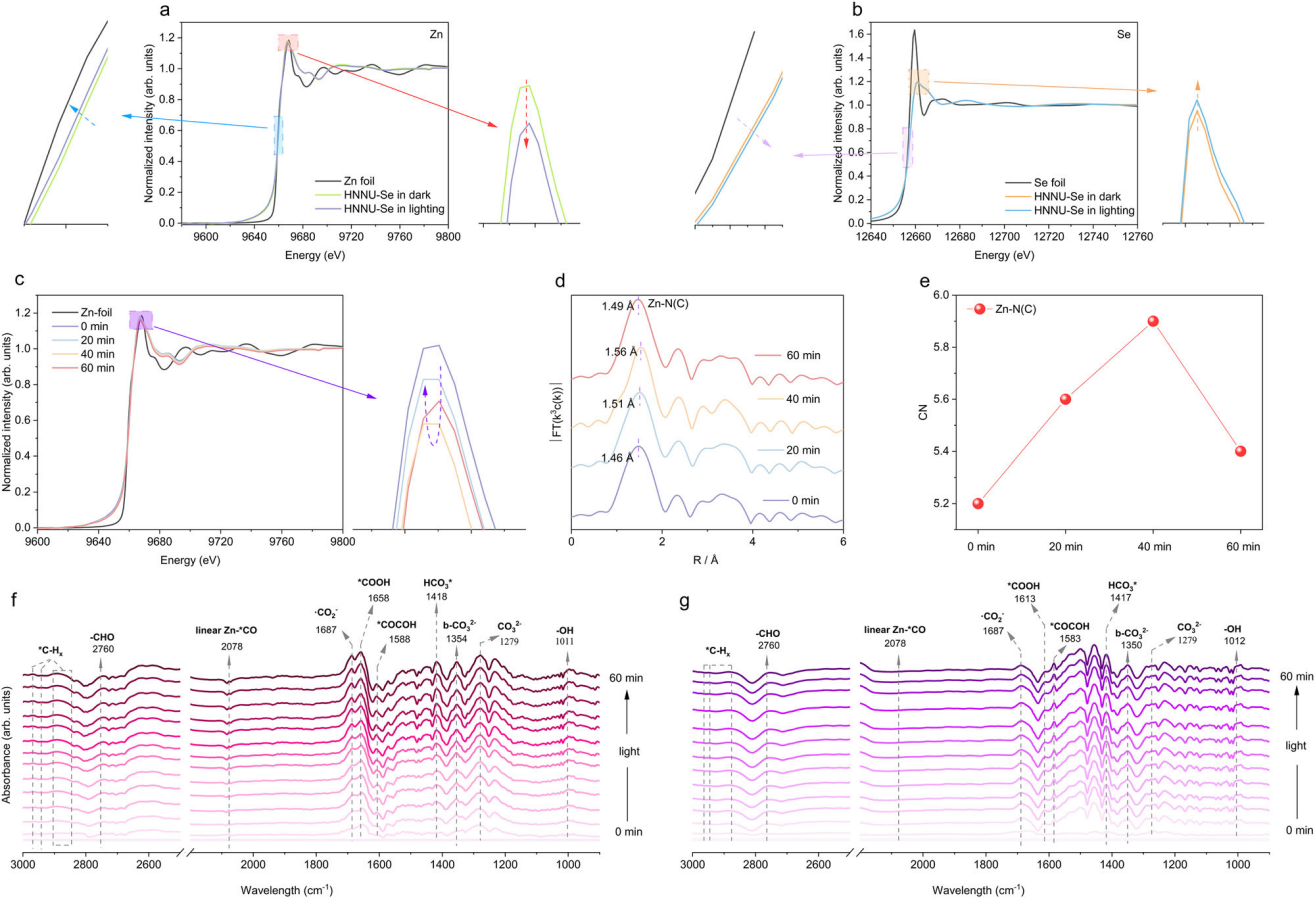
In situ XAFS and in situ DRIFTS measurement. **a**,** b** In situ Zn and Se K-edge XANES spectra of HNNU-Se before and after light irradiation (magnified views of the absorption edges and white-line peaks are shown in the left and right panels, respectively). **c** Operando Zn K-edge XANES spectra with local magnified view of the white-line peak (right). **d** FT of K^3^-weighted Operando Zn K-edge EXAFS upon light irradiation for different time (The spectrum labeled “0 min” was obtained immediately after lighting. The spectra labeled “20 min”, “40 min” and “60 min” were collected 20, 40, and 60 min after illumination, respectively). **e** Fitted changes in coordination number (CN) of Zn-N(C) shells at different states. **f** In situ DRIFTS spectra for photocatalytic CO_2_ conversion over HNNU-Se.** g** In situ DRIFTS spectra for photocatalytic CO_2_ conversion over HNNU-O.

In situ X-ray absorption fine structure (XAFS) was employed to probe charge transfer in HNNU-X (X = O, S, Se). As shown in Fig. 7a, the Zn K-edge X-ray absorption near-edge structure (XANES) exhibits a pre-edge feature at ~9661.3 eV, characteristic of Zn(II) specie^40^. Upon illumination, the absorption edge shifts to lower energy with a concomitant decrease in white-line intensity, indicating an increase in electron density around Zn²⁺ centers. In contrast, the Se K-edge XANES shows a positive shift in absorption edge and enhanced white-line intensity under light irradiation (Fig. 7b), reflecting a decrease in electron density at SeCN⁻ moieties. These in situ XAFS results provide direct evidence for photoinduced electron transfer from XCN⁻ anions to [Zn(tpy)]²^+^ center, in agreement with DFT-predicted frontier orbital distribution. This directed charge transfer within HNNU-X (X = O, S, Se) was also confirmed by the negative shifts in binding energy of Zn^2+^ species, accompanied by positive shifts in binding energies of X elements (O, S, or Se) in corresponding XCN^-^ anions in in situ XPS measurement (Supplementary Fig. 15)^41^.

Operando XAFS further reveals the change in electronic structure and local coordination environment of Zn center in HNNU-Se during photocatalytic CO_2_ reduction. The measurement was done from 0 to 60 min, and spectra were collected after an interval of 20 min. In Zn K-edge XANES spectra (Fig. 7c), the white-line intensity gradually decreases during the initial 40 min of illumination, reflecting increased electron density and/or coordination of CO_2_-derived intermediates at Zn sites, and subsequently recovers upon product desorption. Local structure of Zn was further analyzed by extended X-ray absorption fine structure (EXAFS). The pristine HNNU-Se exhibits a dominant peak at ~1.46 Å, corresponding to the Zn–N coordination distance (*d*_Zn-N_). Under illumination, this distance increases to ~1.56 Å after 40 min (Fig. 7d, and Supplementary Fig. 36). Consistently, EXAFS fitting reveals an increase in the coordination number of Zn^2+^ from 5.2 to 5.9 (Fig. 7e, Supplementary Fig. 37 and Supplementary Table 6), which can be attributed to the coordination of CO_2_-derived intermediates at Zn sites. With further irradiation, the Zn–N distance decreases, accompanied by a reduction in coordination number (Fig. 7d, e, Supplementary Fig. 37 and Supplementary Table 6), indicating the restoration of Zn^2+^ coordination environment. These local coordination change provides evidence that Zn^2+^ centers act as the catalytic sites for CO₂ activation and conversion.

In situ DRIFTS technique provides more insight into on the key intermediates of PCO_2_R (Fig. 7f, g). The measurement was done from 0 to 60 min, and spectra were collected after an interval of 5 min over typical HNNU-Se and HNNU-O. Several characteristic bands corresponding to *CO_2_^-^ (1687 cm⁻¹), *HCO_3_^-^ (1418 cm⁻¹), and *COOH (1658 cm⁻¹) appear upon illumination, indicating progressive CO_2_ activation^42,43^. HNNU-Se exhibits significantly stronger signals than HNNU-O, consistent with enhanced activation capability. Furthermore, single bonded *CO intermediate is observed at 2078 cm^−1^ upon irradiation, which is the crucial intermediate for C-C coupling^44^. Notably, HNNU-Se exhibits the more pronounced *CO band than HNNU-O, signifying the increased *CO coverage on HNNU-Se. The C_2_ intermediate of *COHCO is observed at 1588 cm^−1^, which is crucial for the conversion of CO_2_ to multicarbon (C_2_ + ) products^7^. The absence of isolated *CO hydrogenation intermediates suggests that C–C coupling proceeds cooperatively with hydrogenation to form *COHCO^44^. The rapid formation of *COHCO is the key step that results in high activity and selectivity for CH_3_CHO production. These results demonstrate that the HNNU-Se simultaneously boosts the CO_2_ activation and C-C coupling to form CH_3_CHO product. Indeed, additional vibrational modes of -CHO (2760 cm^−1^) and -CH_3_ (2894, 2943 cm^−1^), attributed to the formed CH_3_CHO, can be observed over HNNU-Se and HNNU-O with the prolonging of irradiation time^6^. A prominent peak at 1011 cm⁻¹ appears in situ DRIFTS with the irradiation time prolonged, which is assigned to the -OH intermediates adsorbed on catalysts (Fig. 7f)^45^. This provides evidence that water is concurrently oxidized during the photocatalytic CO_2_ reduction.

**Fig. 8:**
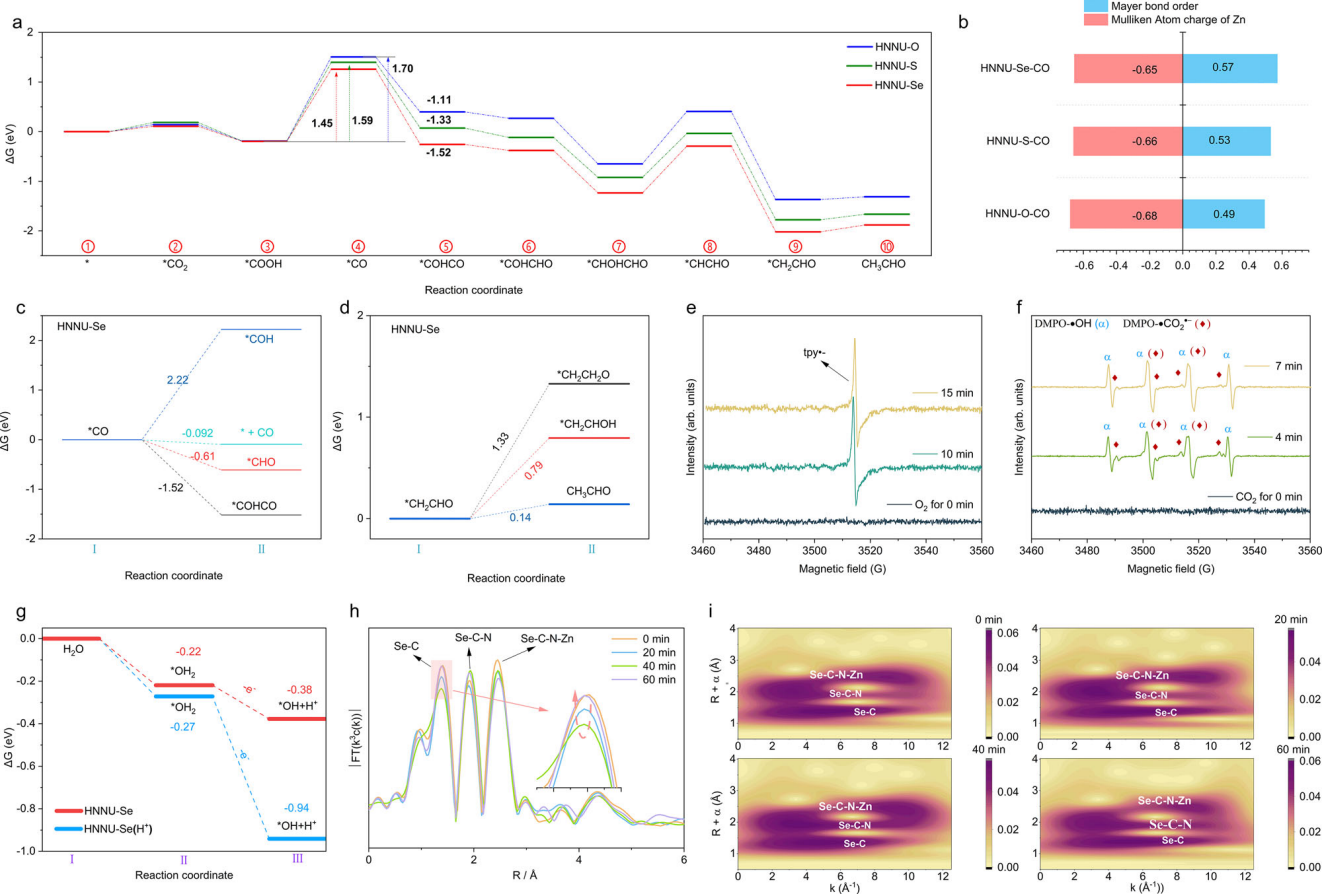
Mechanism studies. **a** Calculated free energy diagram for the reduction of CO_2_ to CH_3_CHO on HNNU-X catalysts. **b** Mayer bond orders and Mulliken atomic charges of HNNU-X with adsorbed CO. **c** Gibbs free energy diagrams for the transformation of *CO into different intermediates over HNNU-Se. **d** Gibbs free energy diagrams for the transformation of *CH_2_CHO into different intermediates over HNNU-Se. **e** In situ EPR spectra over HNNU-Se in acetonitrile under O₂ atmosphere with TEOA as hole scavenger (DMPO was added as the radical trapping agent). **f** In situ EPR spectra over HNNU-Se in water under CO₂ atmosphere without any scavengers. **g** Calculated free energy diagram for H_2_O oxidation through the formation of *OH on HNNU-Se and HNNU-Se(H^+^). **h** FT of K^3^-weighted Operando Se K-edge EXAFS upon light irradiation for different time (pink shading represents the partial enlarged detail of first-shell Se–C scattering paths) (the spectrum labeled “0 min” was obtained immediately after lighting, the spectra labeled “20 min”, “40 min” and “60 min” were collected 20, 40 and 60 min after illumination, respectively). **i** WTs for the *k*^*3*^-weighted Se K-edge EXAFS signals under different conditions.

DFT calculations were performed to elucidate the CO₂-to-CH₃CHO reduction pathway over HNNU-X (X = O, S, Se) catalysts. All the computations have been performed by the Density functional theory (DFT)^46^ within the def2-SV(P)^47^ basis and B3LYP^48^ functional using Gaussian 16 software^49^. All calculations used the DFT-D3^50^ method to describe weak interactions within the system. To account for the solvation effects, all geometry optimizations and frequency calculations were performed with the IEF-PCM model using water as solvent^51–53^. For all calculations of Gibbs free energy changes associated with proton-coupled electron transfer (PCET) steps, we used the computational hydrogen electrode model proposed by Nørskov et al.^54^. Several limitations of the computational model should be noted: the solvent environment was treated using an implicit integral equation formalism polarizable continuum model (IEF-PCM) model rather than explicit solvent molecules, the computational hydrogen electrode (CHE) treatment does not explicitly describe the real photoexcitation process, and the analysis focuses on thermodynamic free-energy trends without considering kinetic barriers. All electronic property analyses were conducted by processing the fchk files using Multiwfn^55,56^. Figure 8a depicts the Gibbs free energy profiles for the complete CO₂ photoreduction pathway. Gibbs free energies (G) of intermediates bound to corresponding HNNU-X (X = O, S, Se) are listed in Supplementary Tables 7–10. Corresponding stepwise structural models are presented in Supplementary Figs. 38–40. The conversion of *COOH to *CO is identified as the most thermodynamically uphill step along the reaction pathway, where HNNU-Se has the minimum Δ*G* change (Δ*G* = 1.45 eV). To further probe *CO formation, Mulliken charge and Mayer bond order (MBO) analyses were conducted for the HNNU-X-*CO intermediates. As shown in Fig. 8b, the Zn centers in these three HNNU-X-*CO intermediates carry positive Mulliken charges, revealing that *CO preferentially coordinates to Zn sites. Notably, the Zn atom in HNNU-Se-*CO (0.65552) is less positively charged than in HNNU-S-*CO (0.66076) and HNNUO-*CO (0.68062), suggesting stronger electron donation and enhanced Zn–CO interaction. Consistently, the MBO of Zn–C bond in HNNU-Se-*CO (0.57) exceeds those in HNNU-S-*CO (0.53) and HNNUO-*CO (0.49) (Fig. 8b), indicative of a more stabilized *CO intermediate. This stabilization accounts for the reduced Δ*G* associated with *CO formation on HNNU-Se. These results demonstrate that the incorporation of SeCN⁻ anions markedly promotes *CO formation, thereby increasing *CO surface coverage on HNNU-Se, in agreement with in situ DRIFTS measurements. Furthermore, HNNU-Se exhibits the weakest CO desorption energy (−0.092 eV), compared to HNNU-S ( − 0.23 eV) and HNNU-O ( − 0.34 eV) (Fig. 8c vs. Supplementary Fig. 41), indicating a higher *CO coverage that facilitates the key C–C coupling step.

Figure 8c, d delineate the thermodynamic landscape of *CO transformation over HNNU-Se, encompassing desorption, hydrogenation, and C–C coupling pathways. Obviously, the direct hydrogenation pathway (*CO → *COH) is thermodynamically unfavorable, whereas alternative *CO conversion routes are exergonic (Δ*G* < 0). Among these, *CO preferentially undergoes hydrogenation-assisted C–C coupling to form *COHCO rather than desorbing from the catalyst surface, which is crucial for the formation of C_2_ species^6^. To elucidate the C–C coupling mechanism from *CO to *COHCO, we first considered conventional dual-site pathways. Owing to steric constraints imposed by the coordination environment, adjacent metal centers cannot approach sufficiently to enable coupling between two adsorbed C_1_ intermediates, thereby excluding dual-site mechanisms. We further evaluated the possibility of co-adsorption of two C_1_ species (e.g., *CO + *CO or *CO + *CHO) on a single metal center; however, such configurations are unstable, with one *CO spontaneously desorbing during structural optimization due to the limited coordination capacity of Zn. Based on these results, we propose an Eley–Rideal (E–R)–type mechanism, consistent with prior reports^57^, in which a *CO species adsorbed on protonated [XCN]⁻ anions directly couples with a Zn-bound *CO to generate a C_2_ intermediate. Notably, structural optimization reveals spontaneous hydrogen transfer from *XCNH to the *CO moiety, promoting hydrogenation-assisted C–C coupling to form *COHCO. This finding highlights the cooperation between hydrogenation and C–C coupling in the formation of C_2_ products. The proposed mechanism is further supported by experimental observations that rapid proton transfer over HNNU-Se facilitates *COHCO generation, a key intermediate in CO_2_ conversion to multicarbon (C_2_ + ) products. Indeed, the formation energy of *COHCO on HNNU-Se (Δ*G* = −1.52 eV) is significantly lower than that on HNNU-S (Δ*G* = −1.33 eV) and HNNU-O (Δ*G* = −1.11 eV), suggesting more favorable *COHCO formation on HNNU-Se (Fig. 8a). This well explains the experimentally observed enhancement in CH_3_CHO production on HNNU-Se.

Regarding C_2_ product selectivity, the protonation of *CH_2_CHO is the key step governing acetaldehyde formation. As shown in Fig. 8d and Supplementary Fig. 41, the energy barrier for *CH_3_CHO formation is substantially lower than those for the competing pathways leading to *CH_2_CH_2_O or *CH_2_CHOH, suppressing the formation of CH₃CH₂OH. This energetic preference accounts for the high selectivity of PCO_2_R toward CH_3_CHO. The theoretical results demonstrate that HNNU-X catalysts, especially HNNU-Se, effectively optimize the C-C coupling pathway and lower the energy barriers of key elementary steps, thereby enhancing both activity and selectivity for CH_3_CHO production.

H_2_O oxidation process was also investigated through combined experimental studies and DFT calculations to elucidate its coupling with CO_2_ reduction. Both O_2_ gas and H_2_O_2_ were detected as the oxidation products during H_2_O oxidation over typical HNNU-Se under a CO_2_ atmosphere (Supplementary Figs. 23–25). To exclude the possibility that H_2_O_2_ originates from O_2_ reduction via superoxide intermediates, in situ EPR spectroscopy was employed to probe O_2_ reduction pathway in acetonitrile. No superoxide (•O_2_^-^) signals are detected in O_2_-saturated acetonitrile in the presence of triethanolamine (TEOA), thereby excluding the O_2_ reduction pathway for •O_2_^-^ formation over HNNU-Se (Fig. 8e). In contrast, characteristic signals assigned to DMPO–•OH adducts^58,59^ (pear-shaped features) emerge in in situ EPR spectra over HNNU-Se in water under CO₂ atmosphere without any scavengers (Fig. 8f). The DMPO–•OH signals intensify upon light irradiation (Fig. 8f), confirming the generation of •OH radicals via water oxidation. These observations demonstrate that H_2_O_2_ is produced from water oxidation rather than O_2_ reduction during photocatalysis. These results demonstrate that HNNU-Se simultaneously drives CO_2_ reduction to CH_3_CHO via photogenerated electrons and H_2_O oxidation to •OH via hole-mediated pathways. Indeed, a sextet signal attributed to •CO_2_ radicals (square-marked) appears accompanied by •OH radicals^60^, indicating efficient electron transfer to CO_2_ during the reduction process. The preferential CO_2_ reduction over O_2_ reduction is further confirmed by a more positive onset potential and higher cathodic current in linear sweep voltammetry (LSV) measurements (Supplementary Fig. 26).

Interestingly, band-structure analysis indicates that direct oxidation of H_2_O to •OH on VB of HNNU-Se is thermodynamically unfavorable, as the VB position (*E*_VB_ = 1.65 eV) is much lower than the oxidation potential of •OH/H_2_O (2.29 eV) (Supplementary Fig. 11b)^61^. The observations suggest a synergistic coupling between CO_2_ reduction and H_2_O oxidation over HNNU-Se, which overcomes the sluggish kinetics of H_2_O oxidation to •OH. This cooperative effect was further elucidated by DFT calculations. Free energy profile reveals that •OH generation via water oxidation is an exothermic and spontaneous process (Δ*G* = −0.38 eV) on HNNU-Se (Fig. 8g). High water affinity of HNNU-Se, especially, the rapid proton (H^+^) transfer to absorbed CO_2_, provides a thermodynamic driving force for •OH formation. This will accelerate the sluggish kinetics of H_2_O oxidation reaction. Indeed, HNNU-Se undergoes gradual protonation during the reaction, as evidenced by a distinct color change from orange to dark red (Supplementary Fig. 29), reminiscent of protonated covalent organic framework^62^. The protonated HNNU-Se makes the •OH generation thermodynamically more favorable (Δ*G* = −0.94 eV) than pristine HNNU-Se (Δ*G* = −0.38 eV). Spectroscopic analyses reveal that the protonation occurs at N atom in [SeCN]^-^ anion (Supplementary Figs. 30–32). XPS measurements of pre-protonated HNNU-X (X = O, S, Se) reveal a decreasing degree of anion protonation in the order HNNU-Se > HNNU-S > HNNU-O (Supplementary Fig. 33). Moreover, protonated HNNU-Se exhibits increased hydrophilicity and enhanced proton conductivity (Supplementary Figs. 34 and 35). The presence of protonated [SeCN]^-^ anions ensures sufficient proton relays proximal to catalytic active site, facilitating proton shuttling to CO_2_ adsorbed on catalytic metal center (*CO_2_).

Operando XAFS was employed to elucidate proton transfer from in situ generated N(H⁺) = C = X species to adsorbed CO₂ during photocatalysis over HNNU-Se (Fig. 8h, i). In the Se K-edge EXAFS spectra, three major peaks are observed at phase-uncorrected radial distances (r) of ~1.39, 1.93, and 2.46 Å, corresponding to the first-shell Se–C, second-shell Se–C–N, and third-shell Se–C–N–Zn scattering paths, respectively. Upon illumination under CO₂ for 40 min, the first-shell Se–C peak gradually weakens and broadens, consistent with protonation of [SeCN]⁻ by photogenerated H⁺ to form N(H⁺) = C=Se species. With prolonged irradiation (60 min), the Se–C peak intensity partially recovers, indicating deprotonation of N(H⁺) = C=Se due to proton transfer to adsorbed CO_2_. These operando XAS results provide direct evidence that CO₂ photoreduction proceeds via participation of in situ generated protons in the form of N(H⁺) = C=Se/H⁺. Owing to the HNNU-Se-mediated proton transfer from [N(H^+^) = C=Se]^-^ group to CO_2_*, CO_2_ is reduced with H_2_ equivalents (N(H^+^) = C=Se/H^+^) but rather with H_2_O, which thus inhibits the competitive hydrogen evolution during CO_2_ reduction. Furthermore, control experiment verified that acetaldehyde originated from direct CO_2_ reduction, rather than from the ethanol oxidation by in situ generated H_2_O_2_ (Supplementary Fig. 22).

**Fig. 9:**
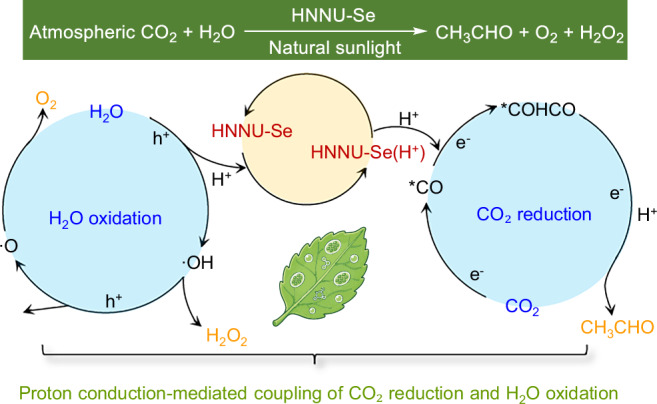
Mechanistic diagram. Proposed mechanism for the overall CO_2_ photoreduction with H_2_O oxidation over HNNU-X.

On the basis of the experimental results and DFT calculations, we propose a plausible mechanism in which photocatalytic CO₂ reduction was coupled with water oxidation over HNNU-X (Fig. 9). Upon solar irradiation, H_2_O was oxidized by photogenerated holes to yield •OH radicals, affording O_2_ and H_2_O_2_ via four-electron and two-electron pathways, respectively^63^. The released protons (H^+^) were stored within the H-bonded matrix of HNNU-X as N(H^+^) = C = X/H^+^ reducing equivalents, which are later utilized for CO_2_ reduction. The [Zn(tpy)]^2+^ centers in HNNU-X act as catalytic sites for CO₂ activation, initiating the formation of *COOH intermediate that subsequently evolve into *CO species. Benefiting from the proximal proton reservoir (N(H^+^) = C = X) surrounding the active [Zn(tpy)]^2^⁺ sites, HNNU-X facilitated C-C coupling of *CO in cooperation with hydrogenation, leading to the formation of *COHCO intermediate via a thermodynamically spontaneous step. This intermediate underwent sequential hydrogenation to *CHOHCHO, followed by dehydration driven by coupled proton–electron transfer to form *CHCHO. Ultimately, *CHCHO was converted to CH_3_CHO through two successive hydrogenation steps (Supplementary Figs. 38–40). In this way, HNNU-X harvests solar energy to drive water oxidation, storing hydrogen in the form of N(H^+^) = C = X/H^+^, which subsequently mediate CO_2_ reduction to acetaldehyde. The proton-relay function of [XCN]^-^ anions thus enables efficient coupling of CO₂ reduction with water oxidation. This synergistic interplay affords selective CH_3_CHO production from photocatalytic CO_2_ conversion under ambient conditions, without the need for sacrificial reagents.

## Discussion

In summary, we have developed the novel [Zn(tpy)](XCN)_2_-incorporated MHOF photocatalysts, HNNU-X (X = O, S, Se), which can selectively capture and concentrate atmospheric CO_2_ under ambient conditions, and in situ convert the adsorbed CO_2_ into acetaldehyde under natural sunlight. Among them, HNNU-Se catalyst exhibits competitive CH_3_CHO evolution rates of 557.1 µmol g^−1^ h^−1^ with high electron-based selectivity of 95% in an outdoor environment without any sacrificial agents. Notably, this production rate surpasses those of previously reported photocatalysts, even under pure CO_2_ and ultraviolet or visible-light irradiation. In situ characterization and DFT calculation results reveal the proton conduction-mediated cooperation of CO_2_ reduction with H_2_O oxidation over HNNU-X for acetaldehyde formation. The presence of proton reservoir of [XCN]^–^ around the active [Zn(tpy)]²⁺ sites makes the rapid transfer of in situ generated H⁺ from solar water splitting to adsorbed CO₂ molecule, allowing for the formation of *COCHO intermediate via a thermodynamically spontaneous step. It is critical for enhancing both the formation rate and selectivity toward CH_3_CHO. Remarkably, HNNU-X also exhibits self-healing capability through simple recrystallization, retaining high catalytic activity over at least seven reuse cycles. This work paves the way toward artificial photosynthesis of acetaldehyde through photoreduction of atmospheric CO_2_ with H_2_O under natural sunlight. Such artificial photosynthesis system depicts a dream-full blueprint that, using only sunlight, air, and water as the starting materials, CH_3_CHO can be continuously generated at catalyst surface without any other energy input. It provides the most sustainable, low-cost, and energy-efficient route for the synthesis of high-value chemicals.

## Methods

### Materials

2-Acetylpyridine, methyl *p*-formylbenzoate and zincnitrate hexahydrate (Zn(NO_3_)_2_ ⋅ 6H_2_O) were supplied by Aladdin. Sodium cyanate (NaOCN), ammonium thiocyanate (NH_4_SCN), and potassium selenocyanate (KSeCN) were purchased by Macklin. Acetonitrile (99%), N,N-dimethylformamide (99%), hydrochloric acid (≥37%), ammonia (≥28%), and ethanol (99%) are obtained from Shanghai Titan Scientific. 2-Acetylpyridine (99%), methyl 4-formylbenzoate (99%), and sodium hydroxide (99%) are obtained from Sinopharm Chemical Reagent Co. LTD. 5,5-Dimethyl-1-pyrroline N-oxide (97%) are obtained from Shanghai Aladdin Reagent Corporation LTD. Deuterium oxide (D_2_O, 99.9%) is obtained from Shanghai Macklin Biochemical Technology Co., Ltd. Other commercial chemicals and solvents were available from local suppliers. The reagents were used as obtained without further purification.

### Characterization methods

Single-crystal X-ray diffraction data was collected at 100 K on an Oxford Diffraction SuperNova diffractometer equipped with Cu-*K*α radiation (λ = 1.5418). The structure was solved by direct method and refined using SHELXL-2014 software package. Morphologies of samples were obtained on scanning electron microscope (SEM) (Hitachi Regulus8100), equipped with energy dispersive X-ray spectroscopy (EDS). Powder X-ray diffraction (PXRD) patterns were collected on a Rigaku 2550 diffractometer equipped with a Cu *K*_α_ radiation source operated at 40 kV and 30 mA. Surface chemical states were analyzed by X-ray photoelectron spectroscopy (XPS) measurements on a Thermo Scientific K-Alpha spectrometer equipped with an Al *K*_α_ X-ray source (λ = 1486.6 eV). Nuclear magnetic resonance (NMR) spectra were recorded on a Bruker Avance-700 spectrometer. Ultraviolet–visible diffuse reflectance spectra (UV–vis DRS) were acquired using a Hitachi U3310 spectrophotometer. Photoluminescence (PL) spectra were collected at room temperature on a Hitachi F-7000 fluorescence spectrometer with an excitation wavelength of 320 nm. Time-resolved photoluminescence (TRPL) decay profiles were recorded using an Edinburgh FLS1000 time-resolved fluorescence spectrometer with an excitation wavelength of 440 nm. Single component adsorption isotherms of CO_2_ and O_2_ were measured volumetrically at 273 K using a Micromeritics ASAP 2020 adsorption analyzer. Breakthrough experiment were conducted on BSD-MAB (Multi-component Adsorption Breakthrough Curve Analyzer) used mass spectrometer as detector in a ternary gas mixture of N_2_/O_2_/CO_2_ (70/20/10, v/v/v) at 298 K and 1 bar under dry conditions. Surface wettability of samples was evaluated by water contact angle which was estimated with a TX500TM rotating drop S6 interfacial tension tester.

### Femtosecond transient absorption spectroscopy (fs-TA)

Femtosecond transient absorption (fs-TA) measurements were performed using a Helios ultrafast Yb: KGW laser system (Light Conversion) equipped with a regenerative amplifier delivering infrared pulses centered at 1030 nm (10 W, 200 kHz, pulse duration <290 fs). Approximately 80% of the output was directed into an optical parametric amplifier (OPA, ORPHEUS, Light Conversion) to generate the 320 nm pump pulses (pulse duration ~70 fs), while the remaining portion was used to produce the probe beam. For probe generation, a fraction of the fundamental beam was focused onto a sapphire crystal to produce a broadband white-light continuum. The pump beam was modulated to 95 Hz using a mechanical chopper, and pump-on/pump-off differential absorption signals were recorded by comparing spectra with and without excitation. For measurements extending to 100 ps time delay, the temporal overlap between pump and probe pulses was controlled using a computer-driven optical delay stage. The pump and probe beams were spatially and temporally overlapped on sample, which was mounted on a glass substrate. The transmitted probe light was collected and detected using a photodiode array detector (Kymera, Andor). The resulting transient absorption data were calibrated and processed using the Harpia software package (Light Conversion). During measurements, HNNU-X samples were dispersed in CH₃CN and excited using a 320 nm pump pulse. Transient absorption data were analysed using Surface Xplorer software. Baseline correction was performed using the pre-excitation region before time zero, after which the corrected transient absorption maps were used to extract representative spectra at selected delay times and kinetic traces at characteristic wavelengths. The kinetic traces were fitted with exponential decay functions to determine the lifetimes of photogenerated charge carriers. These measurements provide information on the charge carrier dynamics and charge separation/transport behavior of the HNNU-X (X = O, S, Se) photocatalysts.

### In situ diffuse reflectance infrared Fourier transform spectroscopy (DRIFTS)

In situ DRIFTS measurements of the photocatalytic process over HNNU-X were conducted on an iS50 FTIR spectrometer (Thermo Fisher Scientific, USA) equipped with a Praying Mantis diffuse reflectance accessory and a reaction chamber (Harrick) fitted with two ZnSe windows and one SiO₂ window. All spectra were recorded at a resolution of 4 cm^−1^ with an average of 128 scans. During the measurements, CO₂ was introduced at a flow rate of 20 mL min⁻¹ through deionized water to generate a humidified CO₂ stream, which was subsequently delivered into the reaction chamber containing the HNNU-X powder. Visible light passes through the quartz window of reaction cell. Before illumination, the humidified CO₂ atmosphere was allowed to equilibrate until a stable baseline was obtained.

### In situ Electron paramagnetic resonance (EPR)

EPR spectra were measured by a Bruker-BioSpin E500 spectrometer at room temperature. To evaluate the capability of HNNU-Se to reduce O_2_ to superoxide (•O_2_^-^), in situ electron paramagnetic resonance (EPR) spectroscopy was employed using 5,5-dimethyl-1-pyrroline N-oxide (DMPO) as the spin-trapping agent. The measurements were conducted in acetonitrile under a pure O_2_ atmosphere with triethanolamine (TEOA) as a hole scavenger. Specifically, HNNU-Se (5 mg) was dispersed in acetonitrile (2 mL), followed by the addition of TEOA (0.5 mL). The suspension was bubbled with O_2_ for 1 h prior to transfer into an O_2_-filled quartz tube. A 300 W xenon lamp was used for irradiation during the in situ measurements.

### In situ X-ray photoelectron spectrometer (XPS)

In situ XPS measurement was performed at a Thermo Fisher Scientific ESCALAB 250Xi spectrometer (Waltham, MA, USA) equipped with 300 W Xe lamp (full arc) as light source. For sample preparation, approximately 10–15 mg of catalyst was pressed into a self-supporting wafer, mounted on a sample holder, and transferred into the introduction chamber. The entire introduction chamber was then evacuated for 30 min to remove surface-adsorbed species, after which the sample holder was transferred into the analysis chamber. After marking the sample position, a set of dark-state XPS data, including survey spectra and high-resolution spectra for O, N, S, Se, and Zn elements, was collected at room temperature (30 °C). Subsequently, a 300 W Xenon lamp (equipped with optional cut-off filters for 420 nm) was positioned at the window of the analysis chamber. The sample was irradiated for 20 min while continuously acquiring XPS spectra to monitor the dynamic evolution of surface chemical states and binding energy shifts under light illumination.

### Operando XAFS

All XAFS data were collected at the 1W1B XAFS beamline of the Beijing Synchrotron Radiation Facility (BSRF), National Center for Nanoscience and Technology (NCNST), Chinese Academy of Sciences, Beijing, China. The storage ring of BSRF operates at 2.5 GeV with a maximum current of 250 mA. The in situ photocatalytic XAFS measurements were carried out in a custom-fabricated in situ reaction cell, where the catalyst was dispersed in aqueous solution with a continuous CO₂ purging. Prior to light irradiation, a set of XAFS spectra was collected under dark conditions as the baseline. A 300 W xenon lamp was then applied to irradiate the reaction cell. The measurement was done from 0 to 60 min, and spectra were collected after an interval of 20 min. For the in situ XAFS spectral data acquisition at Zn K-edge and Se K-edge, we calibrated the position of absorption edge (E₀) using Zn foil and Se foil standard samples, respectively. All spectra were collected in fluorescence mode during the same beam time to ensure data comparability.

### Electrochemical measurements

Photoelectrochemical measurements were conducted using a CHI650E electrochemical workstation (Shanghai Chenhua Instrument Co., Ltd.) in a conventional three-electrode configuration. A platinum plate and an Ag/AgCl electrode serve as the counter and reference electrodes, respectively. The working electrode was fabricated by depositing the as-prepared photocatalyst onto a 1 cm² conductive substrate (2.5 mg of samples were dispersed in a mixture consisting of 700 μL H_2_O, 200 μL acetonitrile and 100 μL 5% Nafion by ultrasonication for 4 h). An aqueous solution of Na_2_SO_4_ (0.5 M, 500 mL, freshly prepared prior to use) was used as the electrolyte solution. Transient photocurrent responses were recorded at 0 V (vs. Ag/AgCl) under visible-light irradiation (300 W Xe lamp, λ > 420 nm) with alternating light on/off intervals of 20 s. Mott–Schottky measurements were performed in dark using a 5 mV AC perturbation at frequencies of 500, 750, and 1000 Hz. Electrochemical impedance spectroscopy (EIS) measurements were conducted in 0.5 M Na_2_SO_4_ electrolyte (pH = 6.8 ± 0.1) over a frequency range of 0.05–100 Hz using a 5 mV AC amplitude at the same bias voltages. The measured solution resistance was 25 ± 5 Ω. Data were recorded using the manufacturer-provided CHI650E workstation software. Linear sweep voltammetry measurements were carried out in 0.5 M Na_2_SO_4_ aqueous solution with a scan rate of 5 mV⋅s^−1^ using Ag/AgCl electrode and Pt electrode as the reference electrode and counter electrode, respectively. The voltage is not iR corrected for all photoelectrochemical tests. The solution resistance was approximately 25 ± 5 Ω as determined by EIS. Potentials were converted to the reversible hydrogen electrode (RHE) scale using E (vs. RHE) = E (vs. Ag/AgCl) + 0.197 V + 0.059 × pH.

### Proton conductivity measurements

Proton conductivity was measured using a two-probe configuration on a Solartron SI 1260 impedance/gain-phase analyser coupled with a Solartron 1287 dielectric interface. Prior to measurement, MHOF samples were pressed into pellets (6 mm in diameter and 1.5 mm in thickness) using a stainless-steel die under a tablet press. All measurements were carried out under a continuous flow of ultra-high-purity N₂ (>99.99%) to minimize the influence of ambient moisture and oxygen. Impedance spectra were collected over the frequency range of 0.05 –100 Hz with an applied AC amplitude of 50 mV. At each temperature, the system was allowed to equilibrate for 20 min, and measurements were repeated until stable impedance values were obtained. The resistance was extracted by fitting the impedance spectra using ZView software. The equivalent circuit model used for data fitting is shown below:

1 σ=l/RS

2 $$\sigma T=A\,exp\,(-{E}_{a}/{K}_{B}T)$$

In the equivalent circuit used for fitting, R1 represents the combined resistance of the wiring and electrodes, whereas R2 corresponds to the bulk resistance of pellet. CPE_1_ describes the non-ideal capacitive response associated with the bulk component. The proton conductivity was determined using Eq. (1), where *σ* denotes the conductivity (S cm⁻¹), *l* represents the thickness of pellet (cm), S is the electrode–electrolyte contact area (cm²), and *R* refers to the bulk resistance (taken as R2) obtained from fitting the semicircle in Nyquist plot using ZView software. The activation energy (*E*a) for proton conduction was evaluated according to the Arrhenius-type relationship in Equation (2), in which *A* is the pre-exponential factor, *k*_B_ is the Boltzmann constant, and *T* is the experiment temperature.

### Preparation of HNNU-X (X = O, S, Se)

Synthesis of HNNU-O. TPBA was firstly synthesized as the terpyridyl-based organic ligand, according to procedure in Section S1 of Supporting Information. The as-prepared TPBA (0.036 g, 0.1 mmol) was combined with zinc nitrate hexahydrate (Zn(NO_3_)_2_ ⋅ 6H_2_O, 0.030 g, 0.1 mmol) in a mixed N,N-dimethylformamide/water solvent (4.5 mL, 2:1, v/v). The resulting suspension was sonicated until a homogeneous mixture was obtained. Sodium cyanate (0.012 g, 0.2 mmol) was then introduced, and the reaction mixture was stirred at room temperature for 2 h. The resulting pale-yellow solid was separated by centrifugation and washed thoroughly with acetone to remove residual starting materials. The collected product was dried in a vacuum oven at 60 °C to afford HNNU-O as a pale-yellow powder.

Synthesis of HNNU-S. The TPBA (0.036 g, 0.1 mmol) and zinc nitrate hexahydrate (Zn(NO_3_)_2_ ⋅ 6H_2_O, 0.030 g, 0.1 mmol) were added in a mixed N,N-dimethylformamide/water solvent (3.75 mL, 3:1, v/v). The resulting suspension was sonicated until a homogeneous mixture was obtained. Then, ammonium thiocyanate (0.016 g, 0.2 mmol) was added. The reaction was allowed to proceed under stirring at room temperature for 2 h. A yellow precipitate was obtained by centrifugation and repeatedly washed with acetone to remove unreacted residues. The solid was subsequently dried under vacuum at 60 °C, yielding HNNU-S as a yellow powder.

Synthesis of HNNU-Se. TPBA (0.036 g, 0.1 mmol) and zinc nitrate hexahydrate (Zn(NO_3_)_2_ ⋅ 6H_2_O, 0.015 g, 0.05 mmol) were added in a mixed N,N-dimethylformamide/water solvent (3.5 mL, 6:1, v/v). The resulting suspension was sonicated until a homogeneous mixture was obtained. Potassium selenocyanate (0.015 g, 0.1 mmol) was then added, and the mixture was stirred at room temperature for 2 h. The orange precipitate was isolated by centrifugation and washed several times with acetone until the residual reactants were removed. Finally, the product was dried in a vacuum oven at 60 °C to give HNNU-Se as a orange powder.

### General procedure for overall photocatalytic CO_2_ reduction with H_2_O oxidation

The photocatalytic CO_2_RR reaction was carried out in a homemade quartz glass reactor under natural sunlight irradiation in atmospheric air or in pure CO_2_ under 300 W Xe lamp irradiation (λ > 420 nm, 250 mW·cm^–2^), as shown in Supplementary Figs. 17a and b. HNNU-X powder (0.005 g) was uniformly dispersed in deionized water (2.5 mL). The suspension was stirred in air atmosphere under natural sunlight or in pure CO_2_ under 300 W Xe lamp irradiation at room temperature. Supplementary Table 2 provided the average temperature and average solar intensity in natural sunlight during these whole studies (The incident solar irradiance was measured using a Delixi radiometer. During measurements, radiometer was placed near the reactor and oriented directly towards sun). Gaseous products were quantified using an Agilent 8860 gas chromatograph equipped with a TDX-01 column with ultrahigh-purity argon serving as the carrier gas. The liquid products were quantified by NMR spectroscopy, in which dimethyl sulfoxide (DMSO, Macklin, 99.99%) was used as the internal standard. Aliquots at an interval of 1 h were drawn from the reaction mixture and analyzed by ^1^H NMR. For ^1^H NMR measurement, 0.5 mL of reaction solution was mixed with 0.1 mL of D_2_O, and 10 μL of DMSO (equivalent to 140.8 μmol) was added as the internal standard. Detailed ^1^H NMR spectra of liquid products were available in Supplementary Figs. 18–20. Product concentrations were determined by comparing the integrated peak areas of the products with that of the DMSO signal at 2.60 ppm. For acetaldehyde quantification, the characteristic doublet at 1.22 ppm was used to determine the production rate (N) according to the following equation:

3 $${{{\rm{N}}}}=\frac{S\times V2\times 140.8\times 6}{2\times V1\times t\times m}$$

where S is the area of double peak of acetaldehyde compared to DMSO (identified to 1 as reference) (0.007 for HNNU-Se, 0.003 for HNNU-S, 0.002 for HNNU-O), *V*2 is the volume of water (2.5 mL) used during photocatalysis, *V*1 is the volume of reaction solution tested (500 µL), t is reaction time (6 h) and m is the mass of catalyst (0.005 g).

The electronic selectivity of liquid phase products was calculated according to required photo-induced electron for the observed products (CH_3_OH, CH_3_CH_2_OH and CH_3_CHO), and the related equation is as follows:

4 $$	{{{{\rm{CH}}}}}_{3}{{{\rm{CHO\; selectivity}}}}(\%)=\\ 	\frac{n\left({acetaldehyde}\right)\times 10}{n\left({acetaldehyde}\right)\times 10+n\left({met}h{anol}\right)\times 6+n\left({ethan}{ol}\right)\times 12}\times 100\%$$

(where n represents the amount of corresponding products, 10, 6 and 12 correspond to the number of electrons)

The photocatalytic CO_2_RR reaction was also carried out in a self-designed quartz reactor equipped with a xenon lamp as light source in pure CO_2_ atmosphere, as shown in Supplementary Fig. 17b. HNNU-X powder (0.005 g) was uniformly dispersed in deionized water (2.5 mL). The suspension was stirred in pure CO_2_ atmosphere. Xenon lamp (300 W) was used as light source to simulate sunlight. Condensate water was introduced to ensure the photoreduction at room temperature.

### Apparent quantum efficiency (AQE)

Apparent quantum efficiency (AQE) was obtained according to the yield of acetaldehyde over **HNNU-X** in 6 h (Supplementary Table 3). The equation was as follow:

5 $${{{\rm{AQE}}}}=\frac{n\times M\times N\times h\times c}{{{{\rm{S}}}}\times P\times t\times \lambda }$$

Where n is the number of reaction electron (10 for acetaldehyde); M is the molar amounts of acetaldehyde formed; N is the avogadro constant (6.022 × 10^23^); h is the Planck constant (6.626 × 10^−34 ^J·s); c is the speed of light (3.0 × 10^8 ^m/s); S is the irradiation area (6.25 cm^2^); P is the incident light intensity at certain wavelength (22.3 W/m^2^ for 450 nm; for the AQE measurement at 450 nm, the Xe lamp was equipped with a 450 nm bandpass filter. The incident light intensity was measured using a radiometer by placing the photodetector probe directly below the irradiation port of the Xe lamp, at the same height as reaction solution); t is the irradiation time (21600 s); λ is the monochromatic light wavelength (450 nm).

The following calculation example is based on the data from CO_2_ photoreduction with HNNU-Se for 6 h: n(CH_3_CHO) = 10; M(CH_3_CHO) = 3.27 × 10^−6^ mol; *N* = 6.022 × 10^23 ^mol^−1^; h = 6.626 × 10^−34 ^J·s; c = 3.0 × 10^8 ^m/s; S = 6.25 × 10⁻⁴ m^2^; *P* = 22.3 W/m^2^; t = 21600 s; λ = 450 nm. For HNNU-Se: AQE% = (10 × 3.27 × 10^−6^ × 6.022 × 10^23^ × 6.626 × 10^−34^ × 3.0 × 10^8^)/(6.25 × 10⁻⁴ × 22.3 × 21,600 × 450 × 10⁻⁹) = 2.90 %.

### Computational details

All the computations have been performed by the DFT within the def2-SV(P) basis and B3LYP functional using Gaussian 16 software (see Supplementary Data 1 for the atomic coordinates of all optimized computational models). All calculations used the DFT-D3 method to describe weak interactions within the system. To account for the solvation effects, all geometry optimizations and frequency calculations were performed with the IEF-PCM model using water as solvent.

The adsorption free energy of adsorbates on catalysts is calculated as follows:

6 $${\Delta {{{\rm{G}}}}}_{{{{\rm{ads}}}}}={\Delta {{{\rm{E}}}}}_{{{{\rm{ads}}}}}+\Delta {{{\rm{ZPE}}}}-{{{\rm{T}}}}\Delta S$$

∆ZPE is the change in zero-point energy;∆S is the change in entropy. Where ∆E_ads_ is

calculated using the following formula.

7 $${\Delta {{{\rm{E}}}}}_{{{{\rm{ads}}}}}={{{{\rm{E}}}}}_{{{{\rm{catalyst}}}}+{{{\rm{adsorbate}}}}}-({{{{\rm{E}}}}}_{{{{\rm{adsorbate}}}}}+{{{{\rm{E}}}}}_{{{{\rm{catalyst}}}}})$$

Here, E_catalyst+adsorbate_ represents the energy of the catalyst adsorbed with the adsorbate, E_adsorbate_ denotes the energy of the isolated adsorbate, and E_catalyst_ is the energy For all calculations of Gibbs free energy changes associated with proton-coupled electron transfer (PCET) steps, we used the computational hydrogen electrode model proposed by Nørskov et al, which is described by the following equation.

8 $${{{\rm{G}}}}({{{{\rm{H}}}}}^{+}+{{{{\rm{e}}}}}^{-})({{{\rm{l}}}})=\frac{1}{2}{{{\rm{G}}}}\left({{{{\rm{H}}}}}_{2}\right)({{{\rm{g}}}})$$

The Gibbs free energy changes of reactions are calculated as:

9 $$\Delta {{{\rm{G}}}}=\Delta {{{\rm{E}}}}+\Delta {{{\rm{ZPE}}}}-{{{\rm{T}}}}\Delta S$$

Where ∆G is the Gibbs free energy change of the reaction, ∆E is the electronic energy differenc obtained from DFT calculations, ∆ZPE is the change in zero-point energy derived from vibrational frequency calculations, and ∆S is the entropy change. T is the absolute temperature, taken as 298.15 K in this work. All electronic property analyses were conducted by processing the fchk files using Multiwfn. The charge and spin multiplicity of all species are provided in the Supporting Information.

## Supplementary information

Supplementary Information

Description of Additional Supplementary File

Supplementary Data 1

Transparent Peer Review file

## Source data

Source Data

## Data Availability

The source data generated in this study are provided in the Source Data file. Source data are provided with this paper. X-ray crystallographic data for the structures reported in this article have been deposited at the Cambridge Crystallographic Data Center (CCDC) under deposition numbers 2441865 (for HNNU-O), 2441866 (for HNNU-S) and 2441870 (for HNNU-Se). These data can be obtained free of charge from The Cambridge Crystallographic Data Center via https://www.ccdc.cam.ac.uk/structures/. Source data are provided with this paper.
