# Supplementary material for: Atmospheric CO₂-to-acetaldehyde artificial photosynthesis in metallo hydrogen-bonded organic frameworks
Source: Nat Commun. 2026 Jul 12;17:8583. doi: 10.1038/s41467-026-75384-z (PMC13486723; doi:10.1038/s41467-026-75384-z)
Supplement: Supplementary file 1 [file 41467_2026_75384_MOESM1_ESM.pdf]

Supplementary Information

Atmospheric CO_2-to-Acetaldehyde Artificial Photosynthesis in Metallo Hydrogen-Bonded Organic Frameworks

Jun Pang,[# 1,2] Xinyu Bai,[# 3] Junjie Dai,[1,2] Sijie Liu,[1,2] Shuangfeng Yin,*[4] Yong Pei,*[3,1] and Rong Tan*[1,2]

[1] National & Local Joint Engineering Laboratory for New Petro-Chemical Materials and Fine Utilization of Resources, Hunan Normal University, Changsha, Hunan 410081, PR China

[2] Key Laboratory of Chemical Biology and Traditional Chinese Medicine Research (Ministry of Education), Hunan Normal University, Changsha, Hunan 410081, PR China

[3] College of Chemistry, Xiangtan University, Xiangtan, Hunan 411105, PR China

[4] School of Chemistry and Chemical Engineering, Hunan University of Science and Technology, Xiangtan, Hunan 411201, PR China

[#] The authors contributed equally.

*Corresponding authors. E-mail: sf_yin@hnu.edu.cn (Shuang-Feng Yin), ypei2@xtu.edu.cn (Yong Pei), and yiyangtanrong@126.com (Rong Tan).

Supplementary Information:

Supplementary Figures 1-41

Supplementary Tables 1-10

Supplementary References 1-19

Section S1. Preparation of **HNNU-X** (X=O, S, Se) for single-crystal X-ray diffraction analysis....................................3

Section S2. Characterization of **HNNU-X** (X=O, S, Se)....................................5

*2.1 Single-crystal X-ray diffraction view and SEM images of **HNNU-X** (X= O, S)*....................................5

*2.2 Crystallographic data of **HNNU-X***....................................5

*2.3 Morphology characterization of **HNNU-X***....................................6

*2.4 Selective adsorption capacity of CO_2 and proton conductivity on **HNNU-X***....................................6

*2.5 Actual band alignments of corresponding **HNNU-X***....................................8

*2.6 Charge separation performance of corresponding **HNNU-X***....................................9

Section S3. Overall photocatalytic CO_2 reduction with H_2O oxidation....................................13

3.1 Photocatalytic activity test....................................13

3.2 Identification of produced acetaldehyde....................................14

*3.3 Control experiment over **HNNU-Se***....................................16

*3.4 Comparative PCO_2R performances of **HNNU-Se** with previous reported photocatalysts*....................................17

*3.5 H_2O oxidation with photogenerated holes over **HNNU-Se** (detection of O_2 and H_2O_2)*....................................18

*3.6 Characterization of the reused **HNNU-X** (X= O, S, Se)*....................................21

Section S4. Investigation of mechanism....................................23

*4.1 Characterization of protonated **HNNU-X** during the PCO_2R reaction process*....................................23

*4.2 EXAFS R-space fitting curves of **HNNU-Se** at different states*....................................26

4.3 Density functional theoretical (DFT) calculation of the reaction mechanism....................................27

Section S1. Preparation of HNNU-X (X=O, S, Se) **for single-crystal X-ray diffraction analysis**

Synthesis of 4-([2,2':6',2''-terpyridin]-4'-yl)benzoic acid (denoted as TPBA). As shown in Supplementary Fig. 1, 2-acetylpyridine (1.48 g, 12.2 mmol) and 4-methoxycarbonylbenzaldehyde (1.0 g, 6.1 mmol) were dissolved in a mixed solvent of ethanol (25 mL) and water (5 mL). Then aqueous ammonia solution (30%, 1 mL) and sodium hydroxide (0.49 g, 12.2 mmol) were added. The mixture was vigorously stirred at room temperature for 17 h, yielding a yellow suspension. Water (50 mL) was then added into the solution. After being acidized with concentrated hydrochloric acid (12 M) to achieve pH value to 5, the formed precipitation was collected by filtration, washed with water, and recrystallized from ethanol, giving TPBA as yellow power (0.85 g, yield 40%). The structure was identified by ^{1}H NMR (Supplementary Fig. 1). ^{1}H NMR (700 MHz, DMSO-d_6) δ (ppm): 13.20 (s, 1 H, -COOH_a), 8.82 (s, 4 H, -Py-H_b), 8.73 (s, 2 H, -Py-H_c), 8.22 (s, 2 H, -Ph-H_d), 8.12 (s, 4 H, -Py-H_e), 7.58 (s, 2 H, -Ph-H_f).

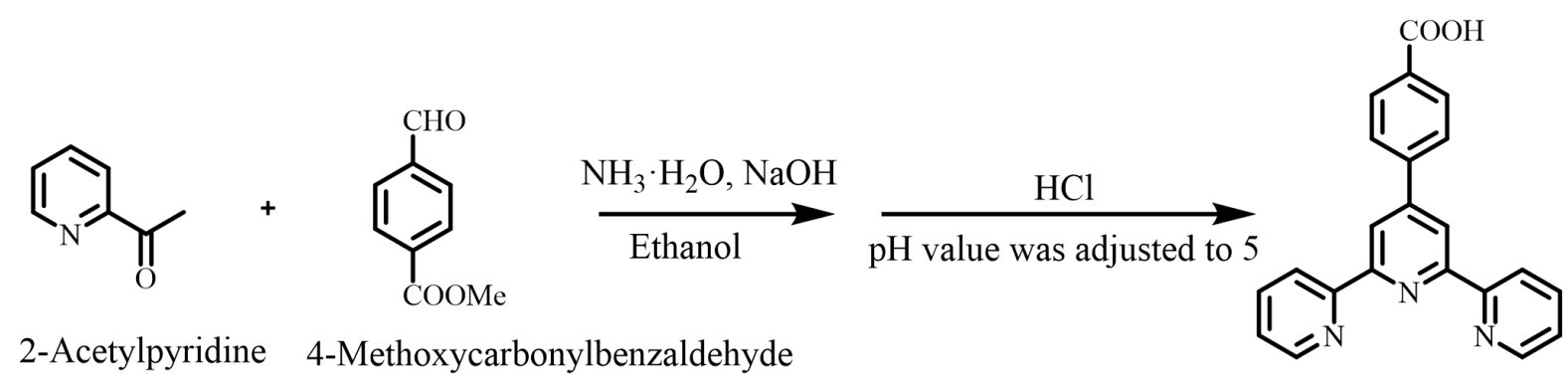

TPBA

Supplementary Fig. 1 | Synthesis of TPBA. Schematic illustration of the synthetic route used to prepare TPBA from the corresponding organic precursors.

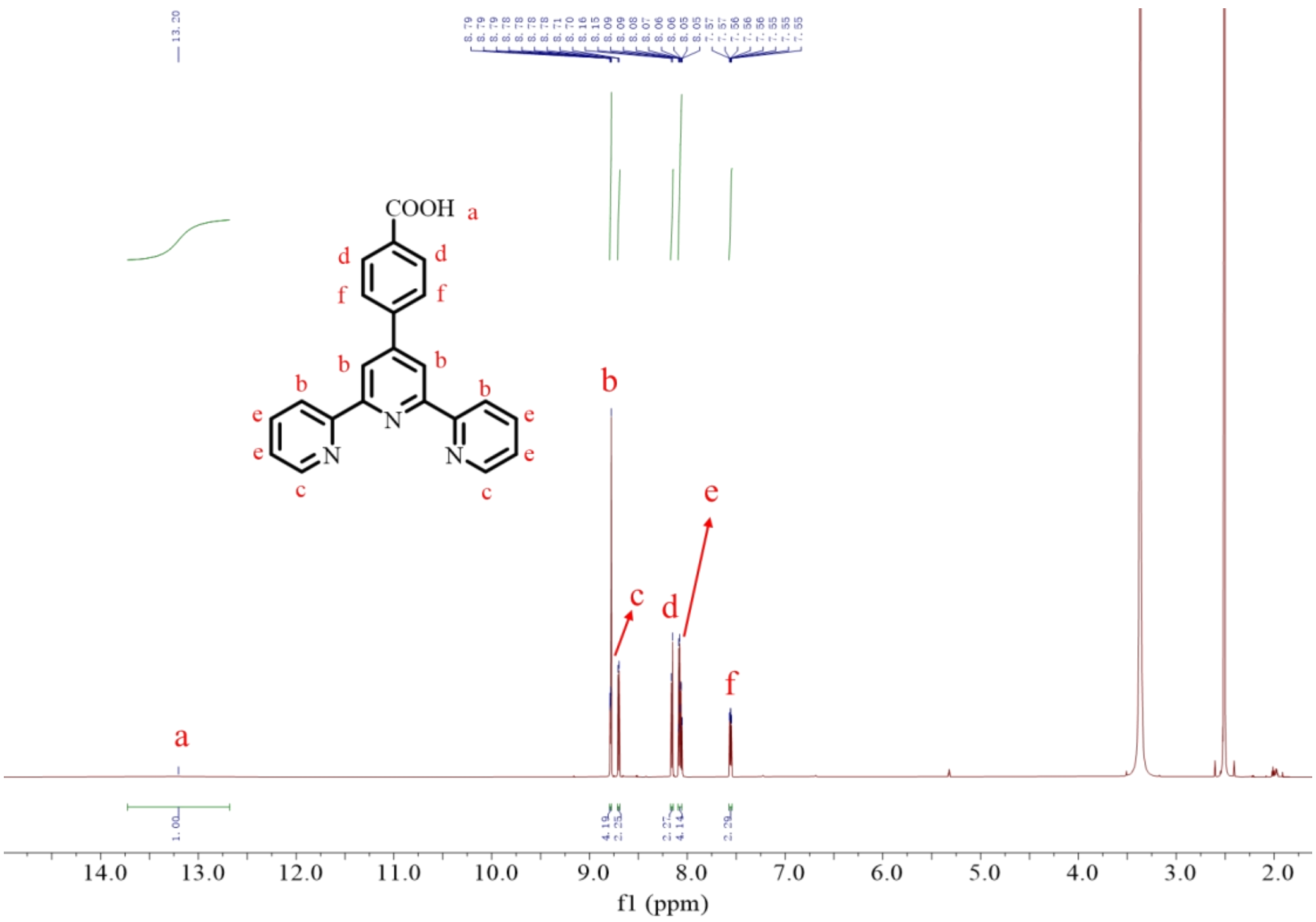

Supplementary Fig. 2 | Identification of TPBA. ^{1}H NMR spectrum of TPBA recorded in DMSO-d_6.

*Synthesis of **HNNU-O** for single-crystal X-ray diffraction analysis.* The resulting precipitate was redissolved in DMF (3 mL), and filtered to obtain a pale-green solution. The supernatant was taken and placed in a 20 mL glass tube. The vial was immersed in a beaker filled with acetone, sealed, and allowed to undergo vapor diffusion at room temperature. After 5 days, pale-yellow needle-shaped crystal of **HNNU-O** suitable for single-crystal X-ray diffraction emerged.

*Synthesis of **HNNU-S** for single-crystal X-ray diffraction analysis.* The resulting precipitate was redissolved in DMF (3 mL), and filtered to obtain a pale-green solution. The supernatant was taken and placed in a 20 mL glass tube. The vial was immersed in a beaker filled with acetone, sealed, and allowed to undergo vapor diffusion at room temperature. After 3 days, yellow needle-shaped crystal of **HNNU-S** suitable for single-crystal X-ray diffraction emerged.

*Synthesis of **HNNU-Se** for single-crystal X-ray diffraction analysis.* The resulting precipitate was redissolved in DMF (3 mL), and filtered to obtain a pale-green solution. The supernatant was taken and placed in a 20 mL glass tube. The vial was immersed in a beaker filled with acetone, sealed, and allowed to undergo vapor diffusion at room temperature. After 2 days, orange needle-shaped crystal of **HNNU-Se** suitable for single-crystal X-ray diffraction emerged.

Section S2. Characterization of HNNU-X

2.1 Single-crystal X-ray diffraction view and SEM images of ***HNNU-X*** *(X= O, S)*

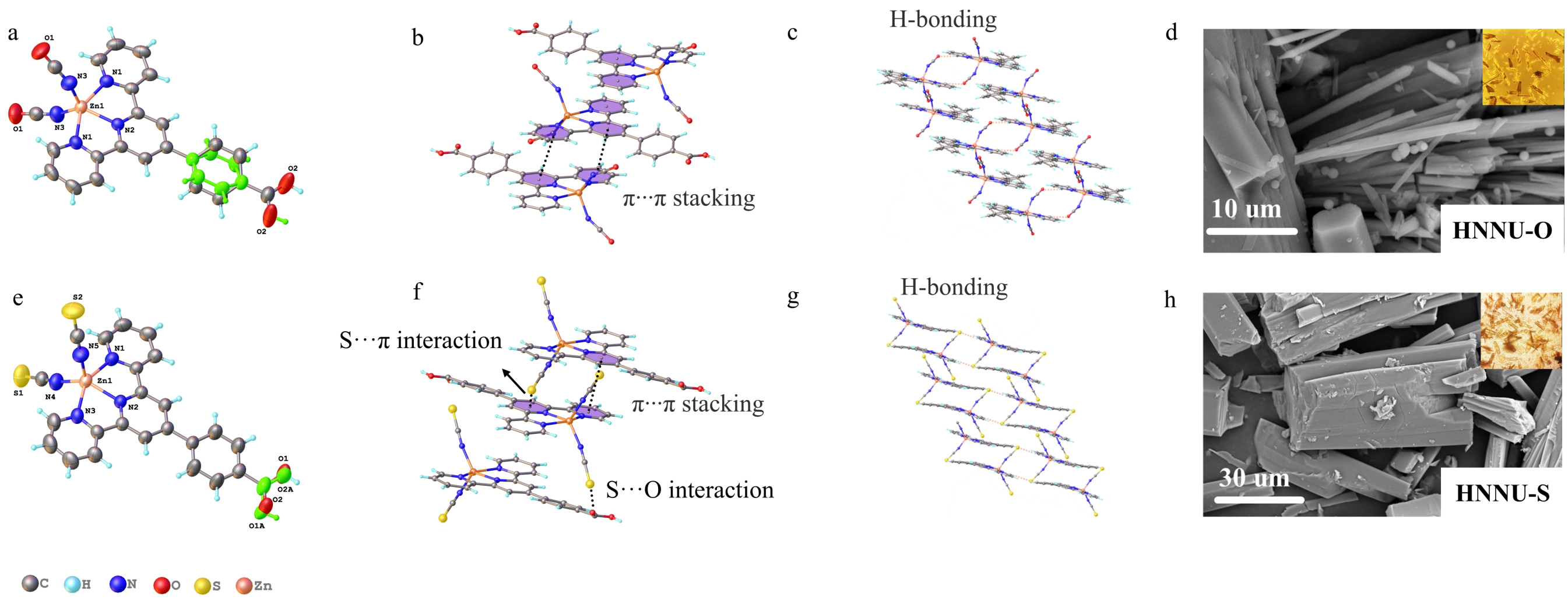

Supplementary Fig. 3 | Structural and morphological characterization of HNNU-O and HNNU-S. Single-crystal X-ray diffraction view of the coordination mode of building blocks, the nodes of HNNU-O (a, b) and HNNU-S (e, f) (gray C, cyan H, red O, blue N, yellow S, green Se, orange Zn). The topologies structures of HNNU-O (c) and HNNU-S (g). SEM images of HNNU-O (d) and HNNU-S (h) (insets are the corresponding single crystal images).

2.2 Crystallographic data of ***HNNU-X (X= O, S, Se)***

Supplementary Table 1. The crystallographic data of **HNNU-X** (X= O, S, Se).

Crystal data	**HNNU-O**	**HNNU-S**	**HNNU-Se**
CCDC number	2441865	2441866	2441870
Crystal system	monoclinic	triclinic	triclinic
Space group	*C2/c*	*P-1*	*P-1*
Molecular formula	$C_{27}H_{22}N_6O_5Zn$	$C_{27}H_{22}N_6O_3S_2Zn$	$C_{27}H_{22}N_6O_3Se_2Zn$
Formula weight	575.9	607.9	701.8
a / Å	13.1715(3)	10.916(5)	10.802(8)
b / Å	21.2524(4)	12.038(5)	11.781(13)
c / Å	11.0043(2)	12.352(6)	12.388(5)
α/°	90	109.068(13)	108.405(7)
β/°	125.649(2)	102.332(18)	101.826(5)
γ/°	90	106.249(19)	105.059(8)

Volume/ Å3	2503.14(10)	1387.1(11)	1371.7(2)
Z	4	2	2
Density/ g cm^{-3}	1.528	1.456	1.699
GOF on F2	1.060	1.032	1.010
R1a[I>2σ(I)]	0.0321	0.0502	0.0496
ωR2b[I>2σ(I)]	0.0875	0.1487	0.0981

2.3 Morphology characterization

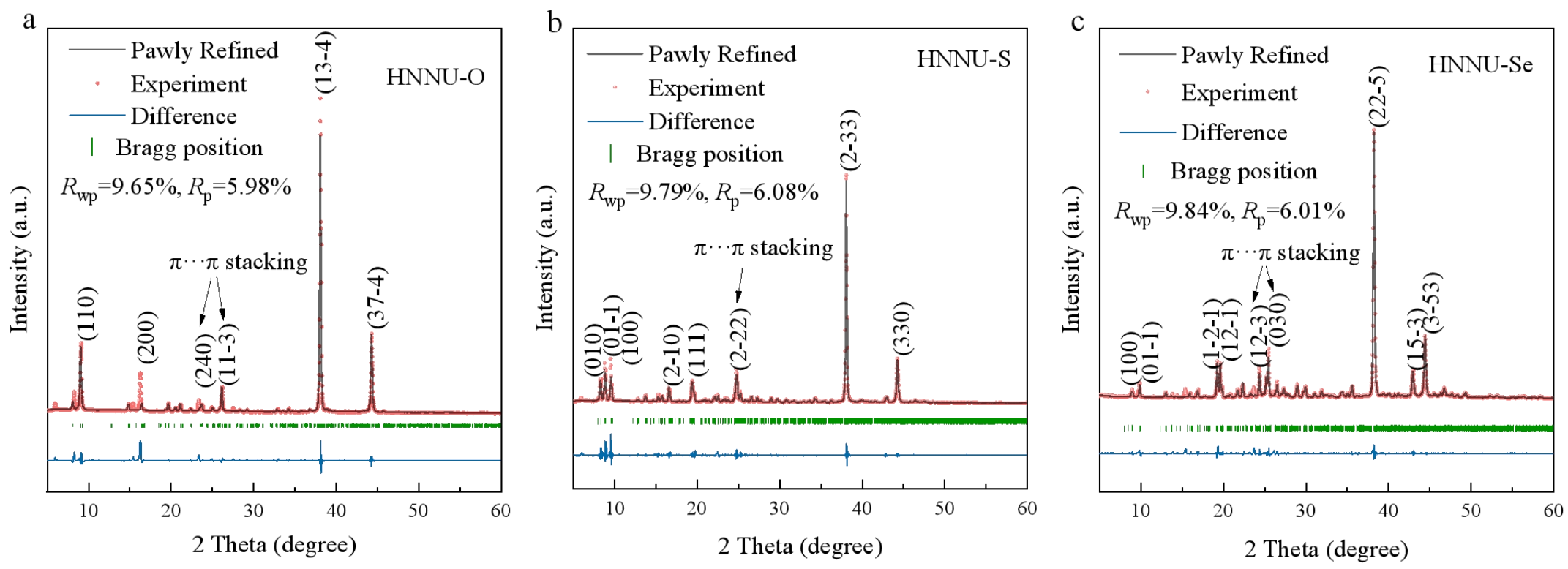

Supplementary Fig. 4 | PXRD patterns. Experimental and simulated PXRD images of HNNU-O (a), HNNU-S (b) and HNNU-Se (c)

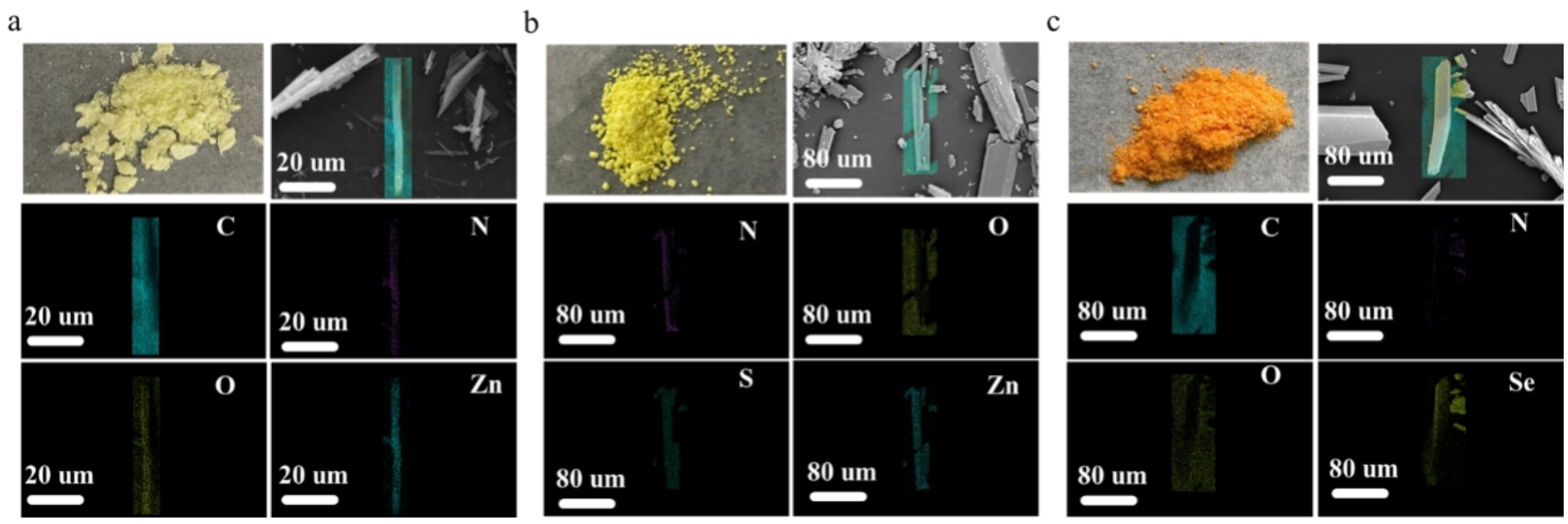

Supplementary Fig. 5 | Optical images and elemental mappings of HNNU-X. Optical images and EDS mapping images of HNNU-O (a), HNNU-S (b) and HNNU-Se (c).

2.4 Selective adsorption capacity of CO_2 and proton conductivity on ***HNNU-X***

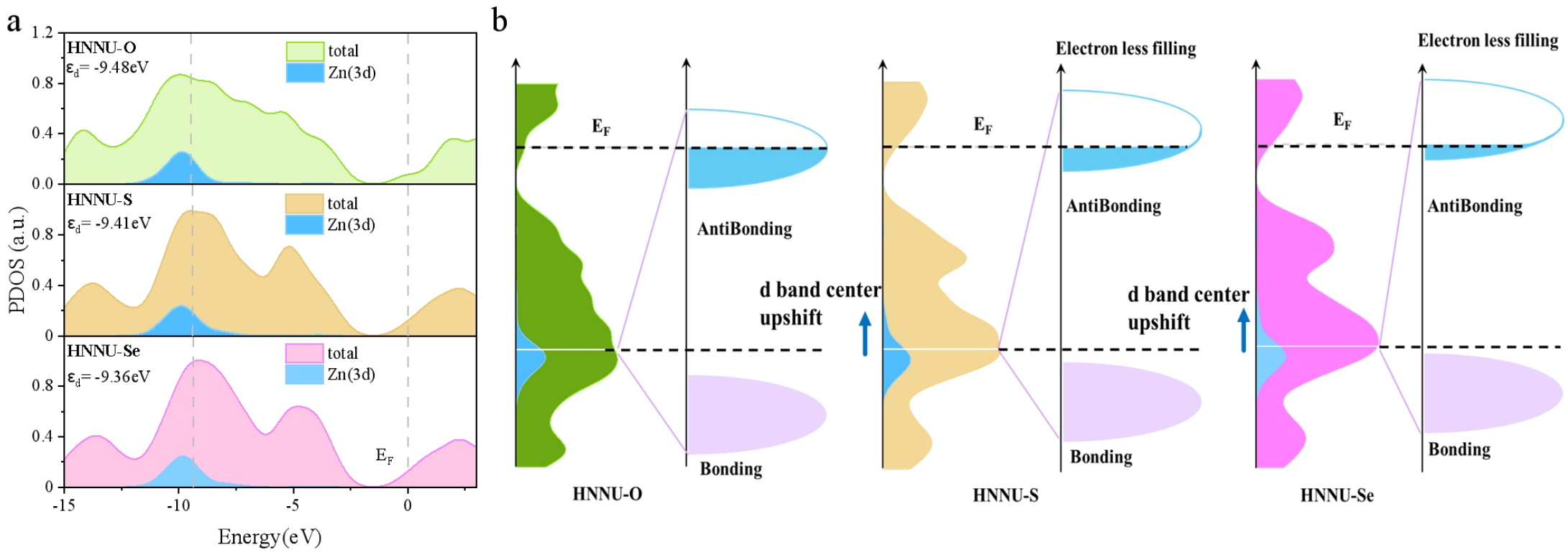

Supplementary Fig. 6 | PDOS analysis of HNNU-X. PDOS of Zn 3d orbital (a) and d-band structure diagram (b) of HNNU-O, HNNU-S, and HNNU-Se.

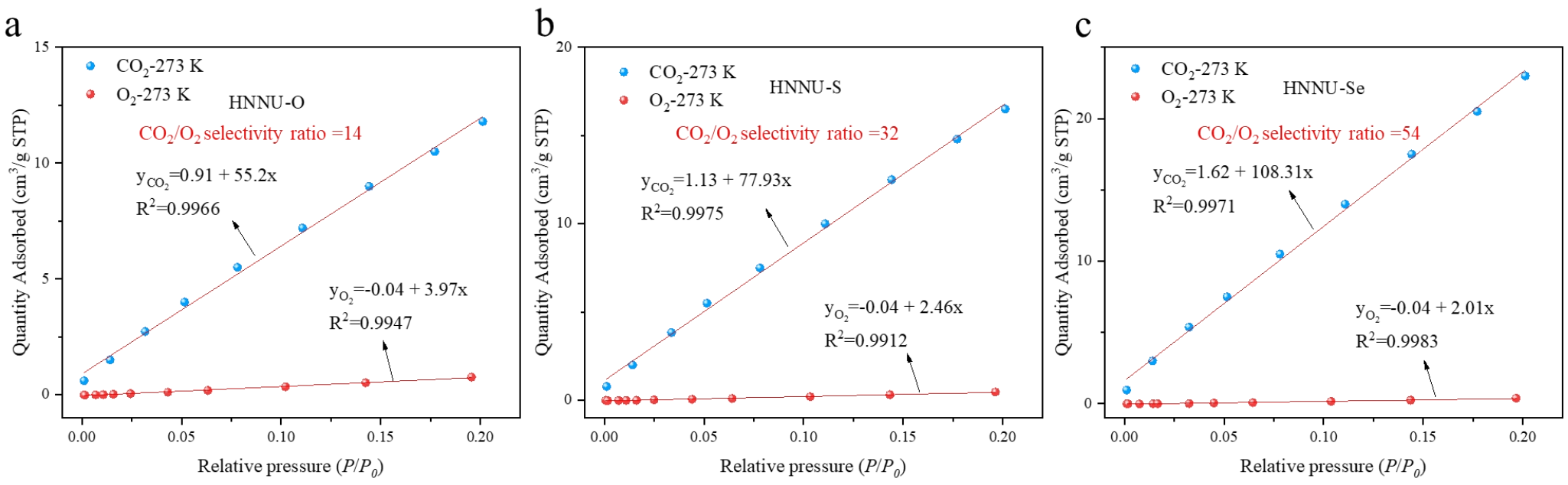

Supplementary Fig. 7 | Selective CO_2 adsorption over O_2 for HNNU-X. Selective CO_2 adsorption over O_2 for HNNU-O (a), HNNU-S (b) and HNNU-Se (c) calculated by initial adsorption slope ratios according to the Ideal Adsorption Solution Theory.

To assess the selective adsorption performance of **HNNU-Se** in air, dynamic breakthrough experiments were conducted at room temperature in simulated air ($N_2/O_2/CO_2$, 70/20/10, v/v/v) with a total flow rate of 5 mL min^{-1}. As depicted in Supplementary Fig. 8, when the ternary mixture passed through **HNNU-Se**, both N_2 and O_2 rapidly penetrated the fixed bed within 6 min, whereas CO_2 was eluted after 20 min. Consequently, a significant gap of approximately 20 min existed between the breakthrough times of N_2/O_2 and CO_2, highlighting not only the high adsorption capacity of **HNNU-Se** for CO_2 in $N_2/O_2/CO_2$ mixture but also its potential for capturing CO_2 from air.

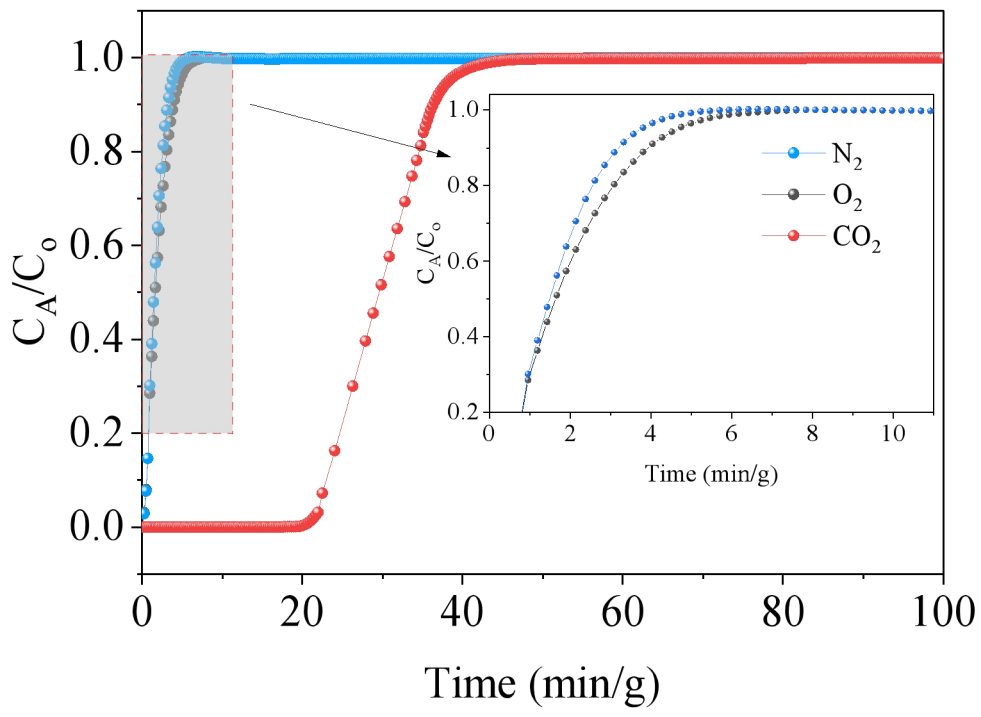

Supplementary Fig. 8 | Breakthrough curve over HNNU-Se. Experimental breakthrough curve for $N_2/O_2/CO_2$ ternary mixture (70/20/10, v/v/v) over HNNU-Se under humid conditions (RH = 90%) with a total inlet flow rate of 5.0 mL mim^{-1}.

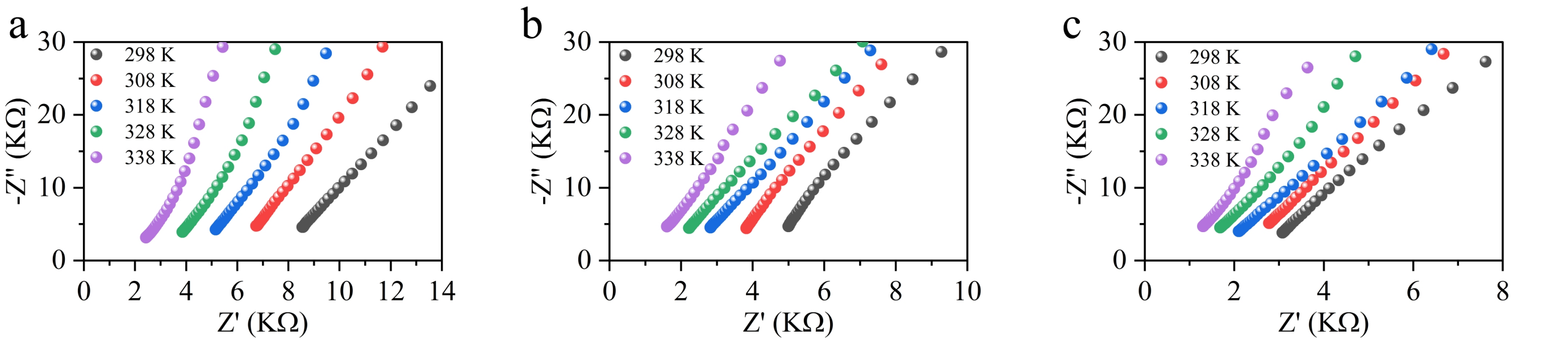

Supplementary Fig. 9 | Proton conductivity analysis of HNNU-X. Proton conductivity of HNNU-O (a), HNNU-S (b), and HNNU-Se (c) at different temperatures with 99% of relative humidity.

2.5 Actual band alignments of corresponding ***HNNU-X***

The actual energy band structures of **HNNU-X** were determined by M-S plots (Supplementary Fig. 10) and UV-vis DRS (Supplementary Fig. 11a). Tauc plot analysis reveals optical band gap energies (E_g) of 3.07 eV for **HNNU-O**, 3.04 eV for **HNNU-S**, and 2.19 eV for **HNNU-Se** (Supplementary Fig. 11a inset), consistent with characteristic semiconductor behavior. The variation of E_g (**HNNU-Se** < **HNNU-S** < **HNNU-O**) aligns with the trend observed in DFT calculations. Mott-Schottky measurements confirmed the n-type semiconductor nature of all materials,with the flat-band potentials (E_f) of **HNNU-O**, **HNNU-S**, and **HNNU-Se** at -0.81, -0.90, and -0.74 V (vs Ag/AgCl), respectively (Supplementary Fig. 10). Since E_f of n-type semiconductor is close to its conduction band (E_{CB}) location, the E_{CB} of **HNNU-O**, **HNNU-S**, and **HNNU-Se** can be estimated as -0.81, -1.03, and -0.74 Vversus Ag/AgCl, corresponding to -0.61, -0.70 and -0.54 eV versus NHE (pH =7), respectively.[1] According to the equation of $E_g = E_{VB} - E_{CB}$, the corresponding valence band potentials (E_{VB}) were determined to be 2.46, 2.34, and 1.65 eV versus NHE. The band alignments of **HNNU-X** (X= O, S, Se) was listed in Supplementary Fig. 11b.

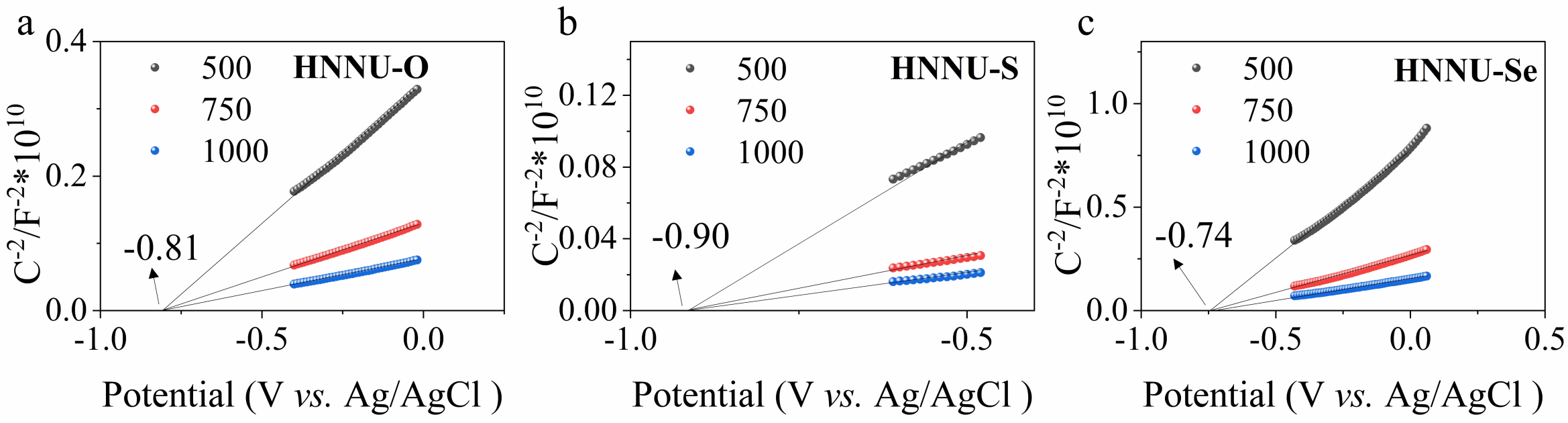

Supplementary Fig. 10 | M-S plots. M-S plots of HNNU-O (a), HNNU-S (b) and HNNU-Se (c).

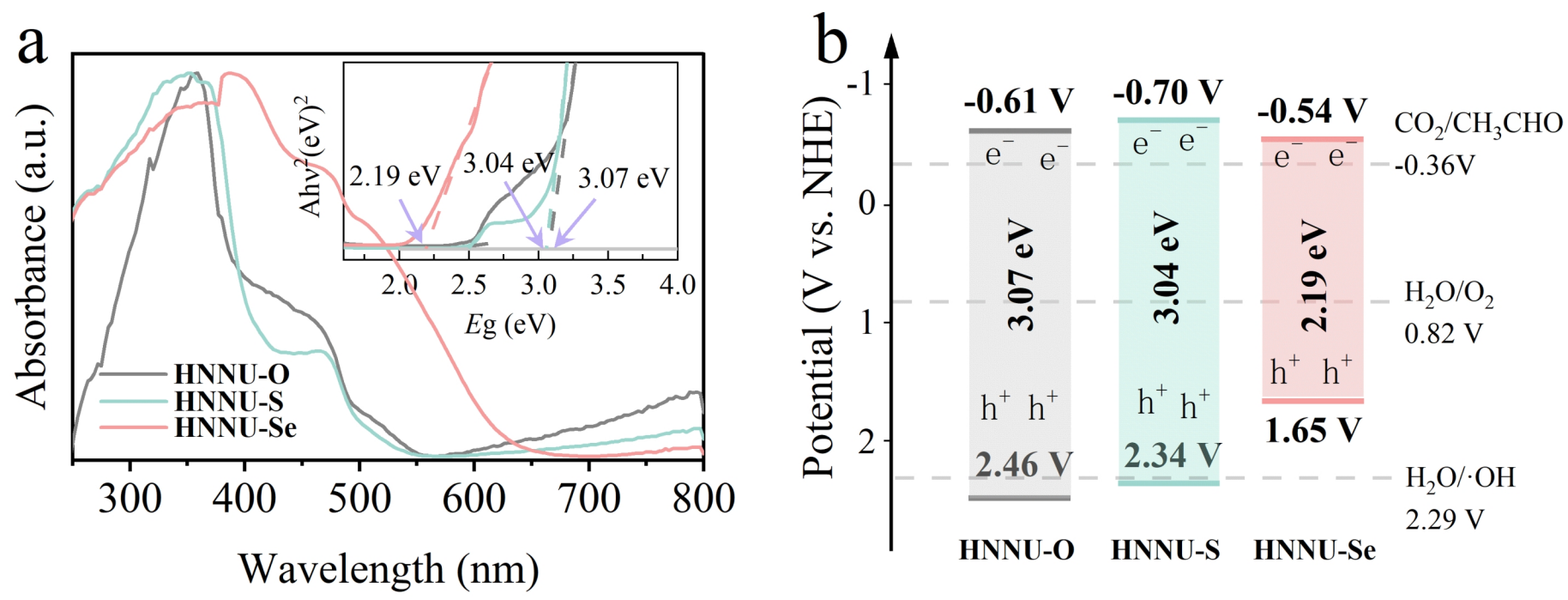

Supplementary Fig. 11 | Band structures of HNNU-X. UV-Vis DRS and Tauc plots (inset) (a), band alignments (b) of HNNU-O, HNNU-S and HNNU-Se.

2.6 Charge separation performance of corresponding ***HNNU-X***

Photoluminescence (PL) spectra were used to analyze the charge recombination in **HNNU-X**, since the PL emission originates from the recombination of free charge carriers. As shown in Supplementary Fig. 12, with an excitation wavelength of 320 nm, **HNNU-O** show the strong luminescence signal, which can be attributed to the radiative recombination of charge carriers. Both **HNNU-S** and **HNNU-Se** shows the much weaker intense signal than **HNNU-O**. This indicates that XCN^- anions can modulate the charge separation and transportation behavior in **HNNU-X**. In particualr, **HNNU-Se** shows the weakest charge recombination in PL spectra, suggesting that the charge recombination process is greatly inhibited in **HNNU-Se**.

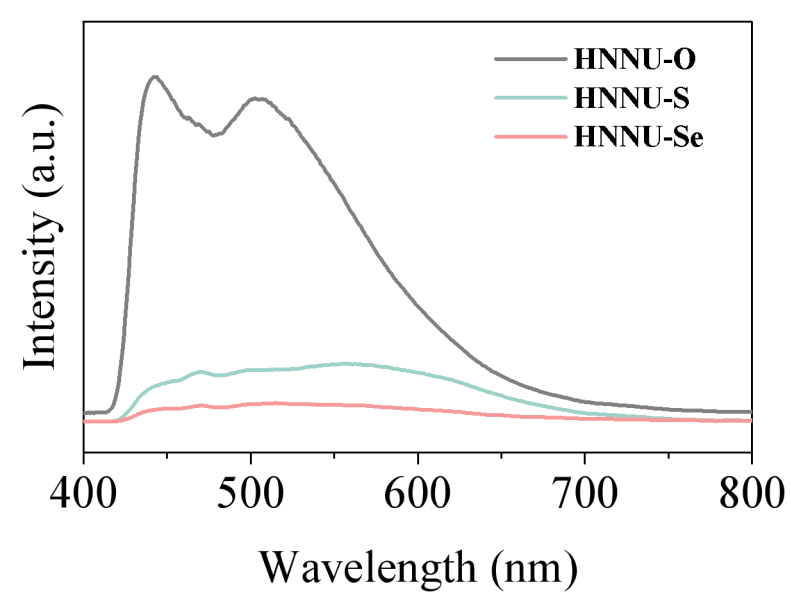

Supplementary Fig. 12 | PL spectra. PL spectra of HNNU-O, HNNU-S and HNNU-Se (excited at 320 nm).

For Femtosecond transient absorption (fs-TA) spectroscopy, double-exponential fitting of the 602 nm decay profiles reveals two distinct relaxation pathways (Supplementary Figs. 13a-c). The fast component ($\tau_1 < 0.5$ ps) corresponds to exciton-exciton (hole and electron) annihilation, and the slower process (τ_2) may be attributed to the electron trapping, which often occurs in organic semiconductors, especially, in conjugated polymers. Additional ESA signals in the 750-800 nm range exhibit nanosecond-scale quenching dynamics (1.74-3.9 ns) (Supplementary Figs. 13d-f), which should correspond to the recombination of electrons and holes.

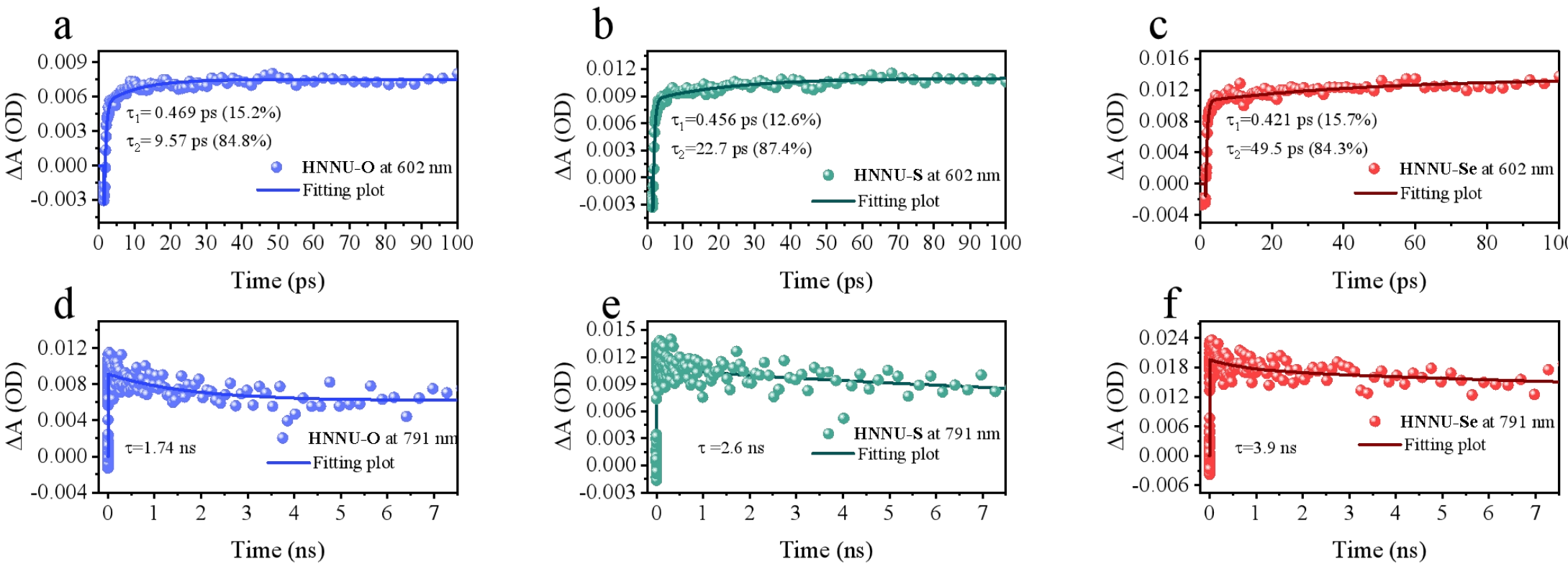

Supplementary Fig. 13 | Kinetic decay profiles. Comparative kinetics decay curves of HNNU-X monitoring at 606 nm (a-c) and 791 nm (d-f) in the ESA region.

To compare the efficient charge seperation, we have determined carriers densities (N_d) of **HNNU-O**, **HNNU-S** and **HNNU-Se** under illumination by M-S plots (Supplementary Figs. 14a-c). N_d was calculated according to following equation:

$$N_d = \frac{2}{e_0 \varepsilon \varepsilon_0} / \frac{d(\frac{1}{C^2})}{dV}$$

where ε_0, ε, e and d(1/C^2)/dV represent the vacuum permittivity (8.86 $\times 10^{-14}$ F·cm^{-1}), organic semiconductor dielectric constant (3.5 for all),[2] elemental charge (1.6 × 10^{-19} C), and slope of line fitted on the M-S plot, respectively. The carrier densities of **HNNU-O**, **HNNU-S** and **HNNU-Se** were calculated to be 14.92 × 10^{17}, 15.89 × $10^{17}$and 23.63 × 10^{17} cm^{-3}, respectively, under the light irradiation (Supplementary Fig. 14d).

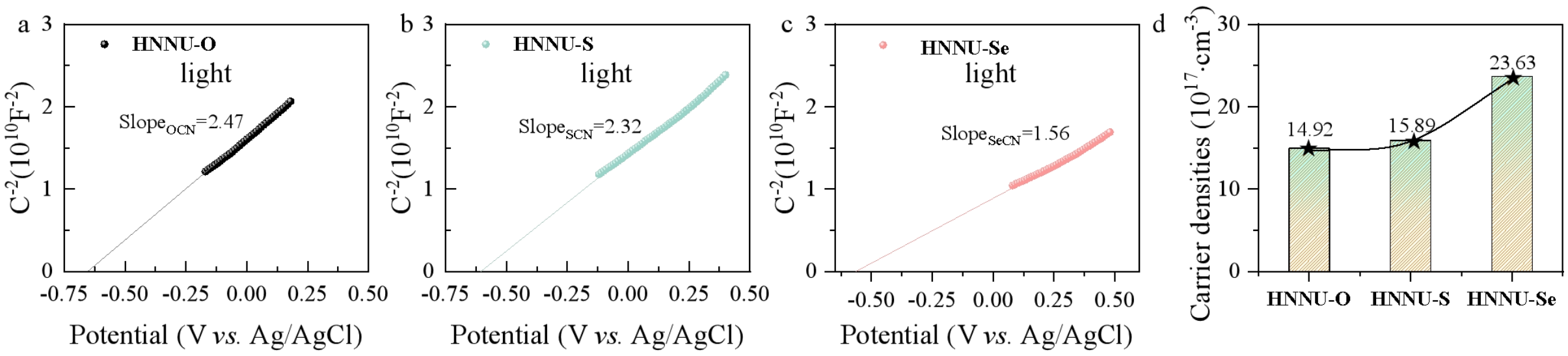

Supplementary Fig. 14 | Carrier concentrations analysis. The M-S plots of HNNU-O (a), HNNU-S (b), and HNNU-Se (c) under visible light illumination, and the calculated carrier concentrations of corresponding HNNU-X (d).

In-situ XPS measurements were performed to investigate the charge transfer in **HNNU-X** (X= O, S, Se). As shown in Supplementary Fig. 15, all **HNNU-X** exhibit two peaks in Zn 2*p* high-resolution spectra in dark, corresponding to Zn $2p_{3/2}$ (around 1044 eV) and Zn $2p_{1/2}$ (around 1021 eV) for Zn^{2+} state. Upon light excitation, the fitting peaks of Zn^{2+} shift to lower binding energy (Zn $2p_{3/2}$ from 1044.87 to 1044.50 eV for **HNNU-Se**, from 1044.79 to 1044.63 eV for **HNNU-S**, and from 1044.74 to 1044.66 eV for **HNNU-O**) (Supplementary Figs. 15a-c). The decrease in binding energy indicates the increase in electron density around Zn^{2+} center in **HNNU-X**.[3] Correspondingly, the binding energies of X (O, S, or Se) atoms in XCN^- anion shifts to higher binding energy, implying the decreased electron concentration of XCN^- anion (Supplementary Figs. 15d-f). The results provide convincing evidence for the transfer of photo-excited electrons from XCN^- anions to $[Zn(tpy)]^{2+}$ center in **HNNU-X**, which is consistent with the DFT calculation of frontier molecular orbitals (Figs. 5a-c). Notably, **HNNU-Se** shows the lowest binding energy of Zn 2*p* (1044.50 eV for Zn $2p_{3/2}$), followed by **HNNU-S** (1044.63 eV for Zn $2p_{3/2}$), and **HNNU-O** (1044.66 eV for Zn $2p_{3/2}$) under light irradiation (Supplementary Figs. 15a-c). It implies that electronic density around Zn^{2+} center follows the sequence of **HNNU-O** < **HNNU-S** < **HNNU-Se**. We reasonably assume that the higher electronic density should favor the C-end adsorption of CO_2 on Zn^{2+} sites to generate carbophilic center which is essential for C-C coupling.

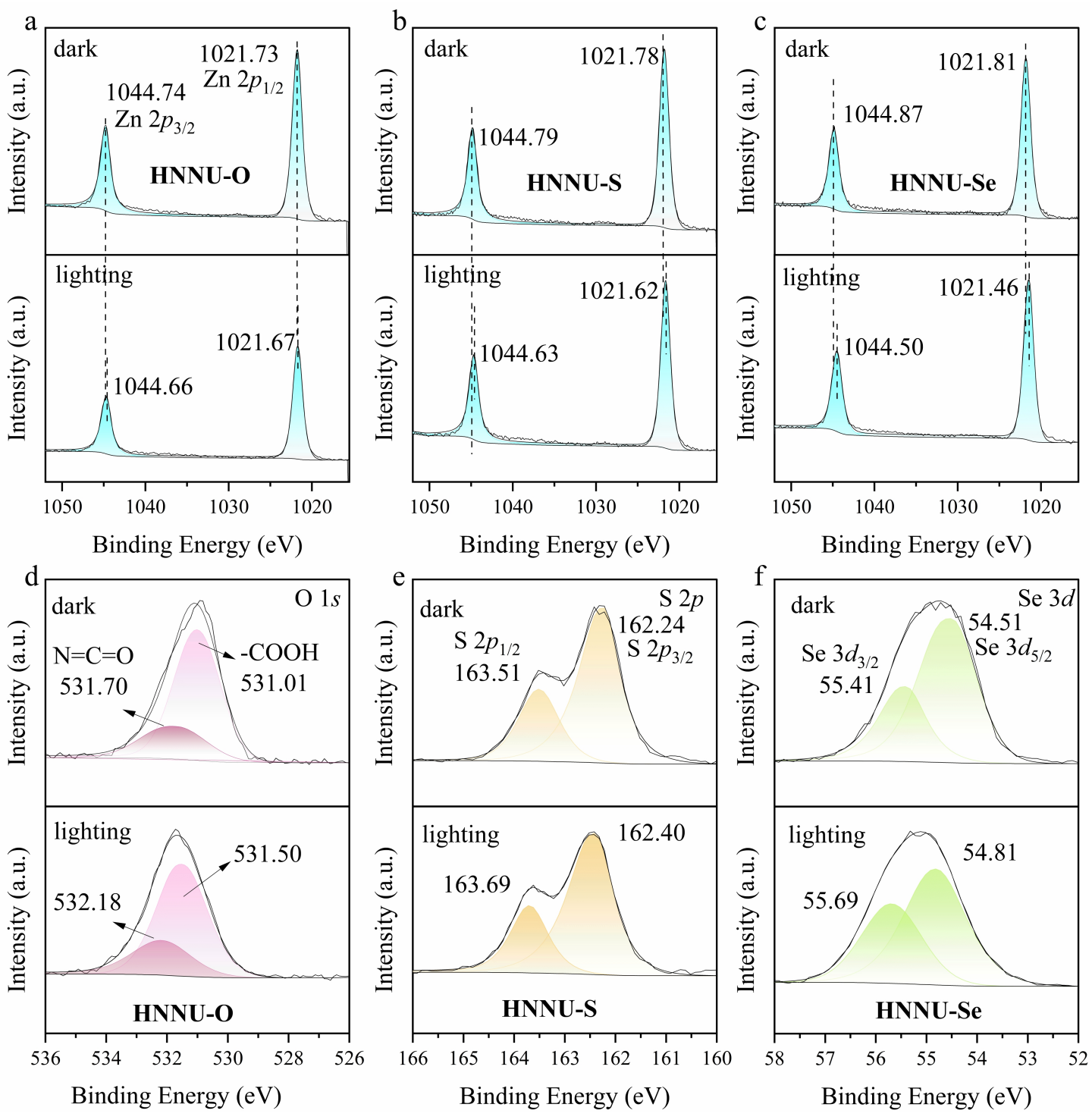

Supplementary Fig. 15 | In situ XPS analysis of HNNU-X. In-situ XPS spectra of Zn 2*p* (a) and O 1*s* (d) over HNNU-O, Zn 2*p* (b) and S 2*p* (e) over HNNU-S, and Zn 2*p* (c) and Se 3*d* (f) over HNNU-Se before and after irradiation.

In-situ X-ray absorption fine structure (XAFS) was performed to investigate the charge transfer in **HNNU-X** (X= O, S, Se). The measurement setup is shown in Supplementary Figs. 16.

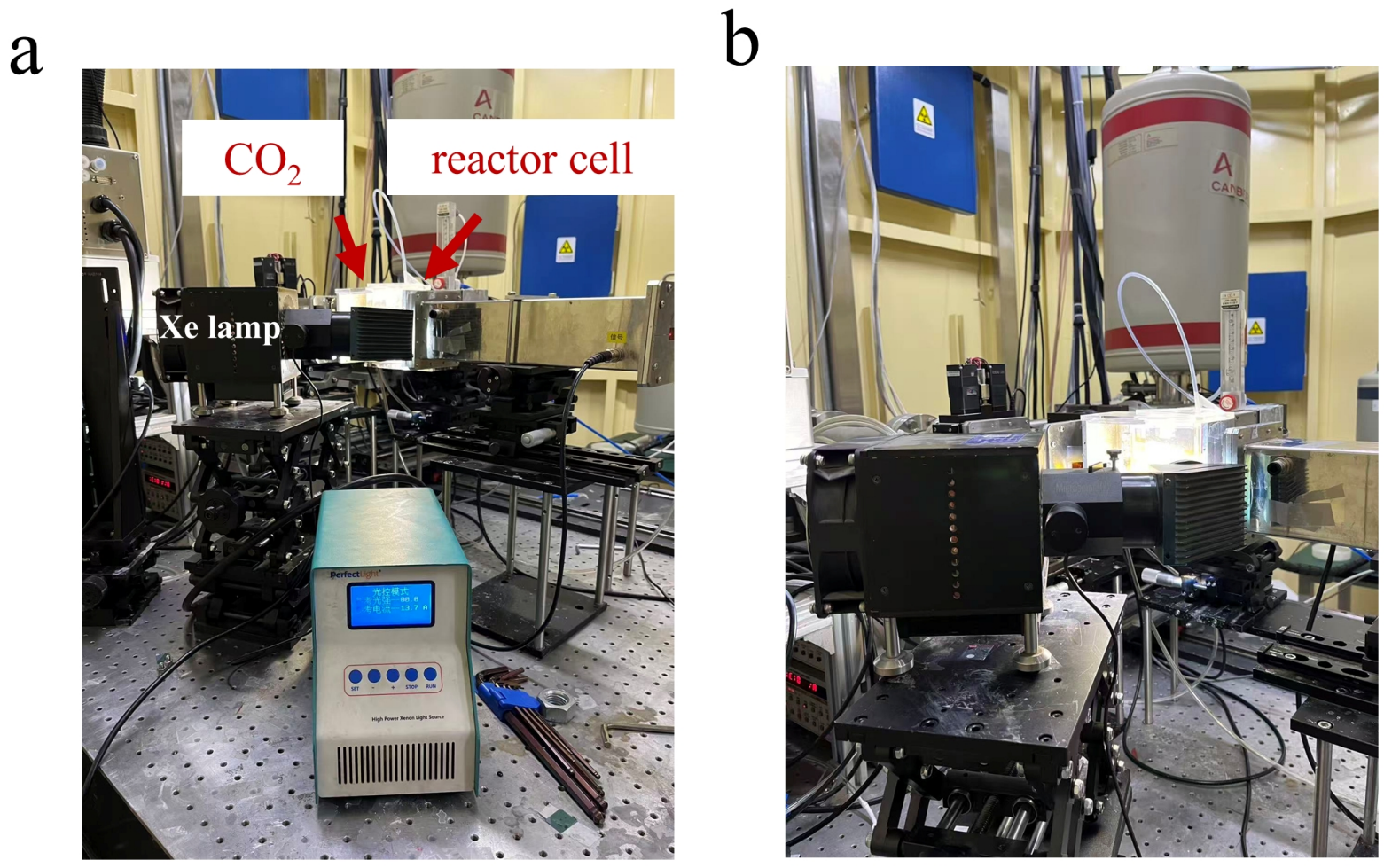

Supplementary Fig. 16 | In situ XAFS measurement setup. Photograph of the in-situ XAFS measurement setup (a and b).

Section S3. Overall photocatalytic CO_2 reduction with H_2O oxidation

3.1 Photocatalytic activity test

The photocatalytic CO_2RR reaction was carried out in a homemade quartz glass reactor under natural sunlight irradiation in atmospheric air, as shown in Supplementary Fig. 17a. Supplementary Table 2 provided the average temperature and average solar intensity in natural sunlight during these whole studies. Detailed ^{1}H NMR spectra of liquid products were available in Supplementary Figs. 18-20. Apparent quantum efficiency (AQE) was obtained according to the yield of acetaldehyde over **HNNU-X** in 6 h (Supplementary Table 3). The equation was as follow:

$$\text{AQE} = \frac{n \times M \times N \times h \times c}{S \times P \times t \times \lambda}$$

Where n is the number of reaction electron (10 for acetaldehyde); M is the molar amounts of acetaldehyde formed; N is the avogadro constant (6.022×10^{23}); h is the Planck constant (6.626×10^{-34} J·s); c is the speed of light (3.0×10^{8} m/s); S is the irradiation area (6.25 cm^2); P is the incident light intensity at certain wavelength (22.3 W/m^2 for 450 nm); t is the irradiation time (21600 s); λ is the monochromatic light wavelength (450 nm).

Furthermore, the photocatalytic CO_2RR reaction was carried out in a self-designed quartz reactor equipped with a xenon lamp as light source in pure CO_2 atmosphere, as shown in Supplementary Fig. 17b.

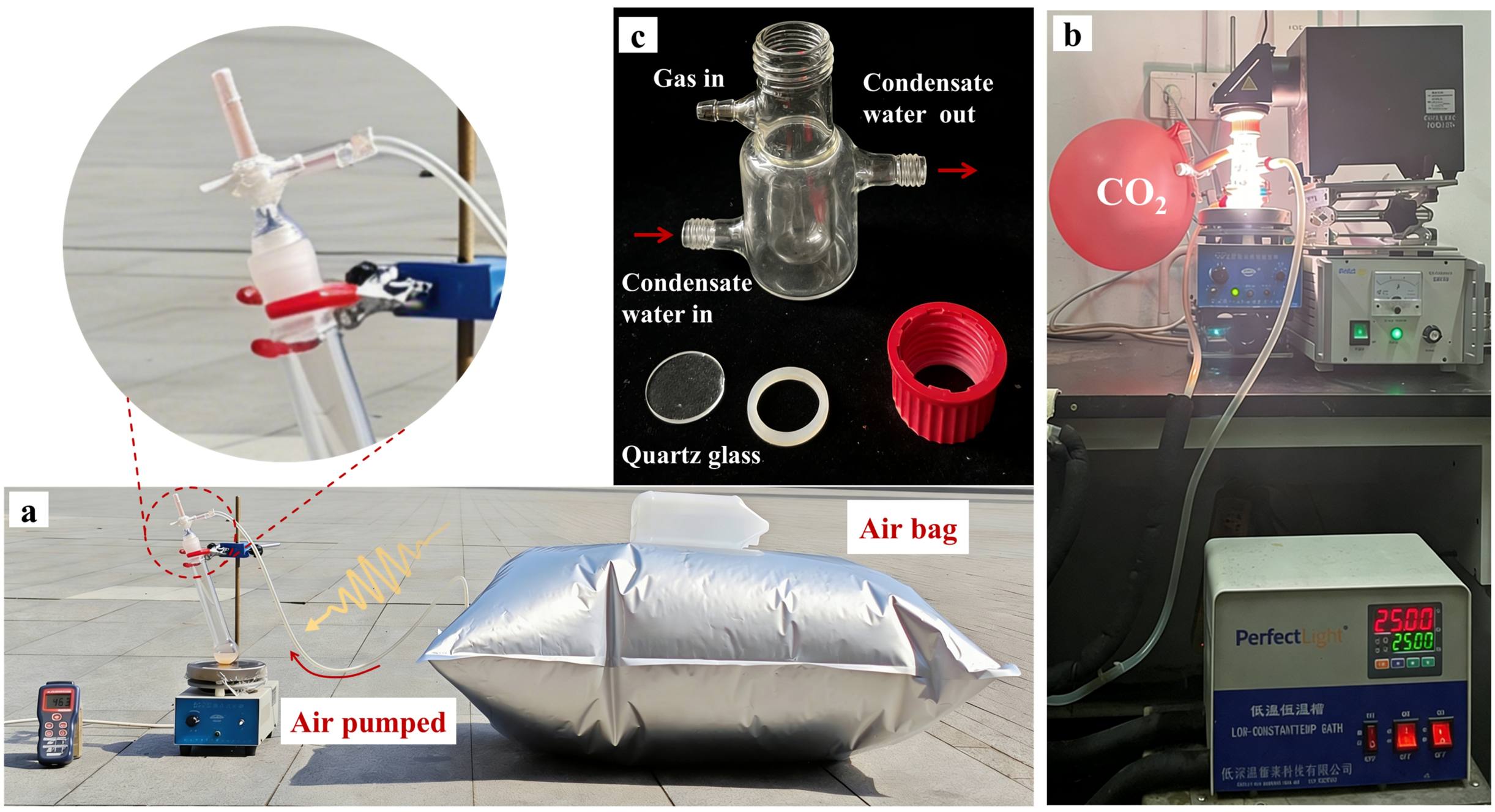

Supplementary Fig. 17 | PCO₂R reaction setup. Demonstration of CO_2 reduction under natural sunlight in air atmosphere and the structure of photoreactor (a). Demonstration of CO_2 reduction under xenon lamp in CO_2 atmosphere (b) and the structure of photoreactor (c).

Supplementary Table 2. The average sunlight intensities and temperatures during the photocatalytic study.

Entry	Date	Average temperature [a] (°C)	Average solar intensity [b] (mW/cm^2)
1	23/07/2024	35.6	45.1
2	24/07/2024	36.2	41.1
3	25/07/2024	36.3	41.9
4	01/08/2024	36.5	37.0
5	02/08/2024	36.2	38.5
6	03/08/2024	37.0	40.5
7	11/08/2024	36.7	41.5
8	12/08/2024	34.9	40.1
9	13/08/2024	35.0	39.7
10	14/08/2024	36.5	41.8

[a] The average value of ambient temperature from 9 a.m. to 5 p.m.

[b] The average value of solar intensities from 9 a.m. to 5 p.m.

Supplementary Table 3. Calculated AQE of **HNNU-X** at 450 nm wavelength.

Catalyst	λ (nm)	Yield of product (μmol)	P (W/m^2)	AQE (%)
**HNNU-Se**	450	3.27	22.3	2.90
**HNNU-S**	450	1.92	22.3	1.70
**HNNU-O**	450	0.81	22.3	1.43

P: incident light intensity at certain wavelength.

3.2 Identification of produced acetaldehyde

To further confirm the formation of CH_3CHO, we recorded ^1H NMR spectrum of the reaction solution over **HNNU-X** with a water suppression mode (Supplementary Figs. 18-20). Distinct proton signals at 1.22 and 9.57 ppm are observed, corresponding to the $C\mathit{H_3}$ and $C\mathit{H}O$ protons of CH_3CHO, respectively.

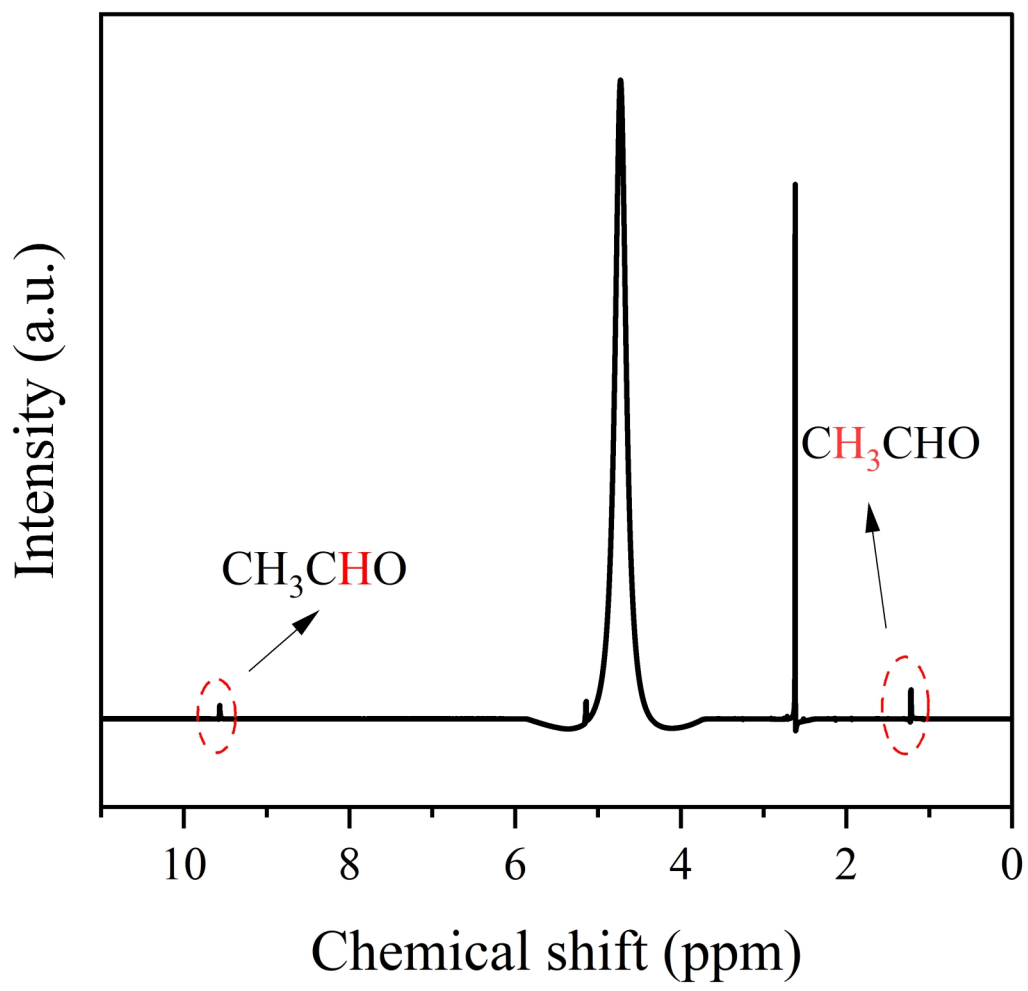

Supplementary Fig. 18 | Identification of liquid product over HNNU-O. ^{1}H NMR spectrum of the liquid products over HNNU-O.

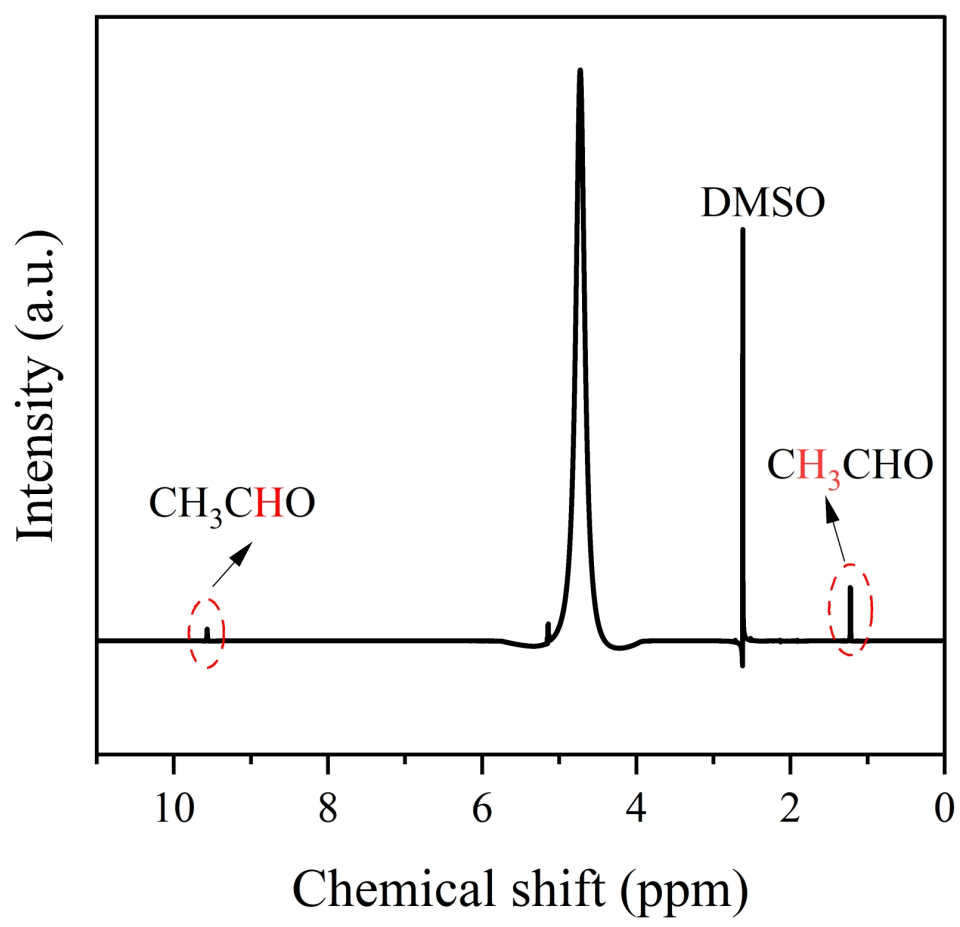

Supplementary Fig. 19 | Identification of liquid product over HNNU-S. ^{1}H NMR spectrum of the liquid products over HNNU-S.

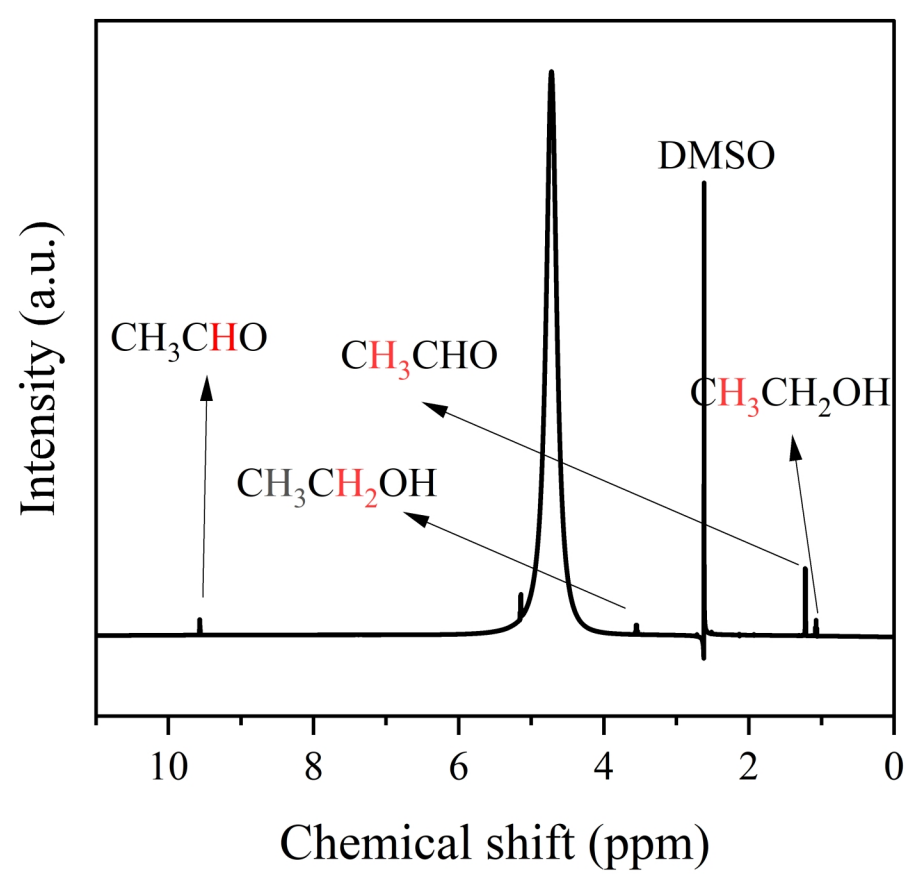

Supplementary Fig. 20 | Identification of liquid product over HNNU-Se. ^{1}H NMR spectrum of the liquid products over HNNU-Se.

To verify that acetaldehyde originates from CO_2 photoreduction, isotopic labeling experiments were performed using $^{13}CO_2$ instead of air over **HNNU-Se**. The labelled products were determined by GC-MS analysis. When $^{13}CO_2$ was used as carbon source, the dominant GC peak at 3.03 min is assigned to ^{13}C-labelled acetaldehyde (Supplementary Fig. 21a). The dominant signal appears at m/z = 46, consistent with the molecular weight of $^{13}CH_3{}^{13}CHO$ (Supplementary Fig. 21b). ^{13}C NMR spectrum of the ^{13}C-labelled acetaldehyde shows characteristic isotope-induced splitting at ~19.4 and 98.9 ppm (Fig. 6h), attributable to the ^{13}*C*H_3 and ^{13}*C*HO carbons in $^{13}CH_3{}^{13}CHO$, respectively. It confirms that CO_2 is the carbon source of CH_3CHO. When D_2O was employed, the peak at m/z= 44 shifts to 48 (Supplementary Figs. 21c and d), corresponding to CD_3CDO. These results verified that water is the real proton source of such photocatalytic CO_2 conversion.

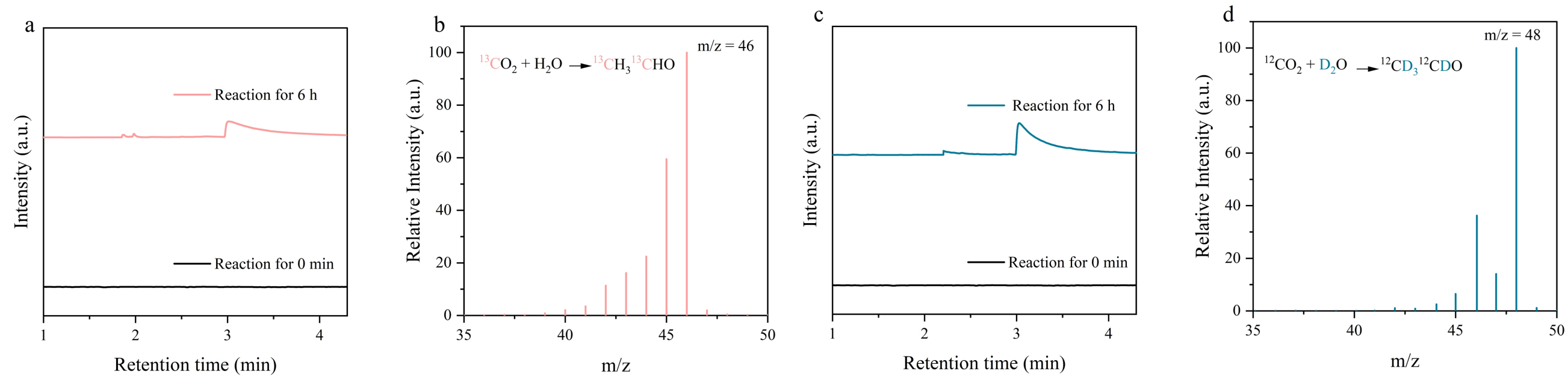

Supplementary Fig. 21 | GC-MS analysis of isotopic labeling experiments. GC (a) and corresponding MS spectrum (b) of produced acetaldehyde from $^{13}CO_2$ coupled with H_2O over HNNU-Se. GC (c) and corresponding MS spectrum (d) of produced acetaldehyde from $^{12}CO_2$ coupled with D_2O over HNNU-Se.

*3.3 Comparative PCO₂R performances of **HNNU-Se** with previous reported photocatalysts*

HNNU-Se outperforms the state-of-the-art photocatalysts reported for CO_2 reduction to CH_3CHO and the other C_2 products in the absence of sacrificial agents, as summarized in Supplementary Table 4.

Supplementary Table 4. Comparison of the state-of-the-art catalysts with **HNNU-Se** under CO_2 atmosphere without Sacrificial agent.

Entry	Catalyst	Rate (μmol g^{-1} h^{-1})	Sel. (%)	AQE (%)	Main product	Gas	Light source
1	**HNNU-Se** [a]	557.1	95.1	2.9 (450 nm)	CH_3CHO	air	Natural sunlight
2	**HNNU-Se** [b]	721.2	93.0	N.A.	CH_3CHO	CO_2	300 W Xe lamp

3	HCN-A [c]	No detected	/	N.A.	CH_3CHO	CO_2	white LED
4	Cu/CZS-Vs [d]	121.5	80	0.53 (400 nm)	CH_3CHO	CO_2	300 W Xe lamp
5	C-SnS_2 [e]	139	N.A.	0.72 (420 nm)	CH_3CHO	CO_2	300W Hg lamp
6	$AgInP_2S_6$ [f]	44.3	73	0.15 (420 nm)	C_2H_4	CO_2	300 W Xe lamp
7	d-UiO 66/MoS_2 [g]	39.0	94	N.A.	CH_3COOH	CO_2	300 W Xe lamp
8	Co_3O_4 [h]	2.95	92.5	N.A.	CH_3COOH	0.03% CO_2	300 W Xe lamp
9	BMO/RP [i]	51.8	<50	N.A.	C_2H_5OH	CO_2	450 W Xe-lamp
10	InCu/PCN [j]	28.5	92	N.A.	C_2H_5OH	CO_2	300 W Xe lamp
11	Au_1/RP [k]	1.32	96	N.A.	C_2H_6	CO_2	300 W Xe lamp

[a] **HNNU-Se** (5 mg), deionized water (2.5 mL), air atmosphere, natural sunlight, reaction temperature (25 ^{o}C) (This work).

[b] **HNNU-Se** (5 mg), deionized water (2.5 mL), CO_2 atmosphere, 300 W Xe lamp ($\lambda \geq 400$ nm), reaction temperature (25 ^{o}C) (This work).

[c] HCN-A (15 mg), acetonitrile (3.0 mL), deionized water (0.3 mL), CO_2 atmosphere, 5 W white LED lamp, reaction temperature (10 ^{o}C).[4]

[d] Cu/CZS-Vs (15 mg), acetonitrile (9.0 mL), deionized water (1.0 mL), CO_2 atmosphere, 300 W Xe lamp ($\lambda \geq 400$ nm; light intensity: 200 mW/cm^2.[5]

[e] C-SnS_2 (0.1 g), water vapour, CO_2 atmosphere, 300 W Halogen lamp, reaction temperature (25 ^{o}C).[6]

[f] $AgInP_2S_6$ (5 mg), deionized water (0.4 mL), CO_2 atmosphere, 300 W Xe lamp.[7]

[g] d-UiO 66/MoS_2 (2 mg), water-vapor-saturated CO_2 gas (pressure reached 0.1 MPa), 300 W Xe lamp (($\lambda > 400$ nm, 100 mW/cm^{-2}).[8]

[h] Co_3O_4 (2.5 mg), deionized water (5 mL), simulated air (0.03% CO_2, 79.98% N_2 and 19.99% O_2), 300 W Xe lamp (AM 1.5G filter, 100 mW/cm^{-2}), reaction temperature (25 ^{o}C).[9]

[i] BMO/RP (5 mg), deionized water (20 mL), CO_2 atmosphere, 450 W Xe lamp (with UV cut-off filter (KG-2 filter and CGA-400) filter), reaction temperature (25 ^{o}C).[10]

[j] InCu/PCN (50 mg), DMF/H_2O (24 mL, 12.5 vol% H_2O), CO_2 atmosphere (0.8 MPa), 300 W Xe lamp (1 W/cm^{-2}).[11]

[k] Au_1/RP (0.05 g), 0.5 g of $KHCO_3$ and 6 M HCl to produce high-purity CO_2 and water vapor atmosphere, 300 W Xe lamp (300 mW·cm^{-2}).[12]

3.4 Control experiments over ***HNNU-Se***

No product was acquired without photocatalyst, light, CO_2, or H_2O (Supplementary Table 5), confirming that photocatalyst, light, CO_2, and H_2O serve as indispensable components during the reaction.

Supplementary Table 5. Photocatalytic CO_2 reduction to acetaldehyde under different controlled conditions. [a]

Entry	Catalyst	CO_2	Light	H_2O	CH_3CHO [b]
1	√	√	√	√	√
2	×	√	√	√	×
3	√	×	√	√	×
4	√	√	×	√	×
5	√	√	√	×	×

[a] Reaction conditions: **HNNU-Se** (5 mg), 25 °C.

[b] Determined by ^1H NMR with 10 μL DMSO as internal standard.

To demonstrate whether acetaldehyde is generated from the oxiadtion of ethanol with H_2O_2, we performd the ethanol oxidation in aqueous H_2O_2 solution with the presence of **HNNU-Se** under light irradiation. ^1H NMR spectra revealed that no acetaldehyde was acquired after 6 hours under the persent conditions (Supplementary Fig. 22). Therefore, acetaldehyde is generated from direct reduction of CO_2, rather than from the oxidation of ethanol with generated H_2O_2.

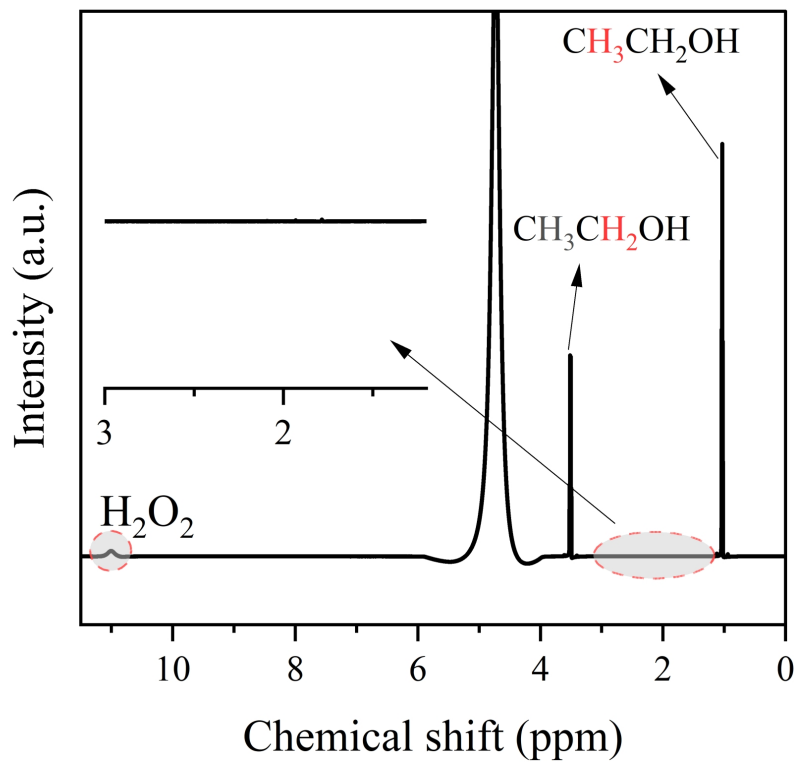

Supplementary Fig. 22 | Exclusion of ethanol oxidation over HNNU-Se. ^1H NMR spectrum of ethanol oxidation in aqueous H_2O_2 solution over HNNU-Se. Reaction condition: HNNU-Se (5 mg), H_2O_2 (1 M, 0.1 mL), ethanol (10 μL), CO_2 atmosphere, 300 W Xe lamp (full arc), reaction time (6 hours), reaction temperature (25 °C).

3.5 H_2O oxidation with photogenerated holes over ***HNNU-Se*** *(detection of O_2 and H_2O_2)*

Detection of hydrogen peroxide (H_2O_2) during water oxidation. Since H_2O_2 is a common product of photocatalytic water oxidation, we have detected the H_2O_2 generation in CO_2 atmosphere using the iodometric method, a standard

analytical technique for H_2O_2 detection.[13] Aqueous potassium hydrogen phthalate solution (1 mL, 0.1 mol L^{-1} a) and aqueous potassium iodide solution (1 mL, 0.4 mol L^{-1}) was added in the tested samples. The mixture was standed for 0.5 h befor determination. Under acidic conditions, iodide ions (I^-) can be readily oxidized by H_2O_2 to form triiodide ion (I_3^-) ($H_2O_2 + 3I^- + 2H^+ \rightarrow I_3^- + 2H_2O$), which exhibits UV-vis absorption peak at 350 nm. As shown in Supplementary Fig. 23a, water oxidation can produce H_2O_2 during the PCO_2RR over **HNNU-Se**. The I_3^- absorption peak at 350 nm gradually increases with the reaction time. Furthermore, the color of reaction system gradually turned yellow with the increased reaction times in the presence of potassium hydrogen phthalate and potassium iodide indicators (Supplementary Fig. 24). It is due to the increase in I_3^- content, which arises from the oxidation of I^- with generated H_2O_2. The generated H_2O_2 was further quantified by standard curve method using UV-vis spectroscopy technology (Supplementary Figs. 23b and c). The H_2O_2 production rate is 151 μmol/g/h over **HNNU-Se** during the PCO_2RR.

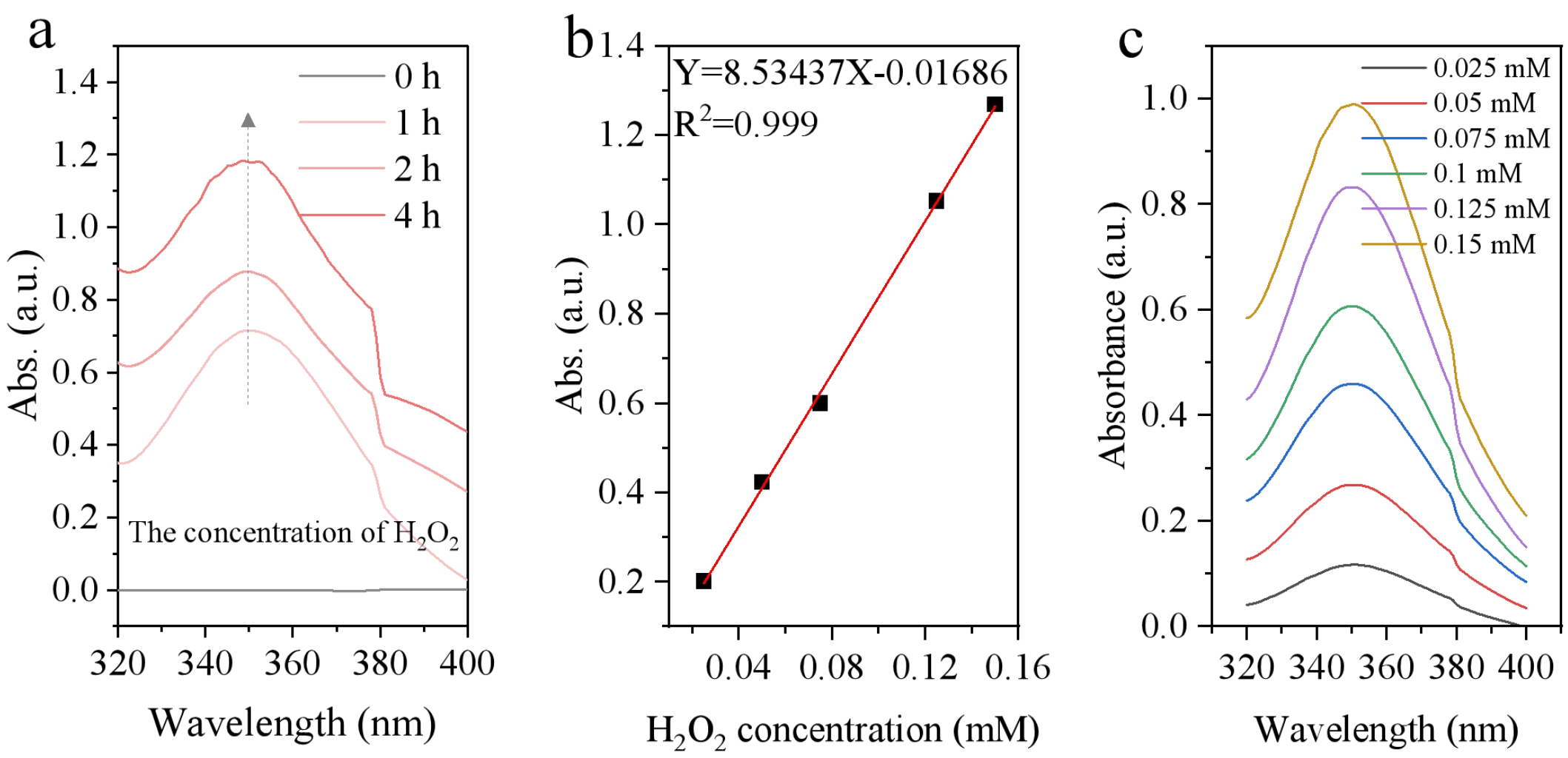

Supplementary Fig. 23 | Determination of H_2O_2 concentration over HNNU-Se. UV-vis spectra of reaction solution over HNNU-Se at different reaction time in the presence of potassium hydrogen phthalate and potassium iodide indicators (a), standard calibration curve for the H_2O_2 concentration determined by iodometric method (b), and UV-vis spectra of aqueous H_2O_2 solution with different concentration in the presence of potassium hydrogen phthalate solution and potassium iodide (c).

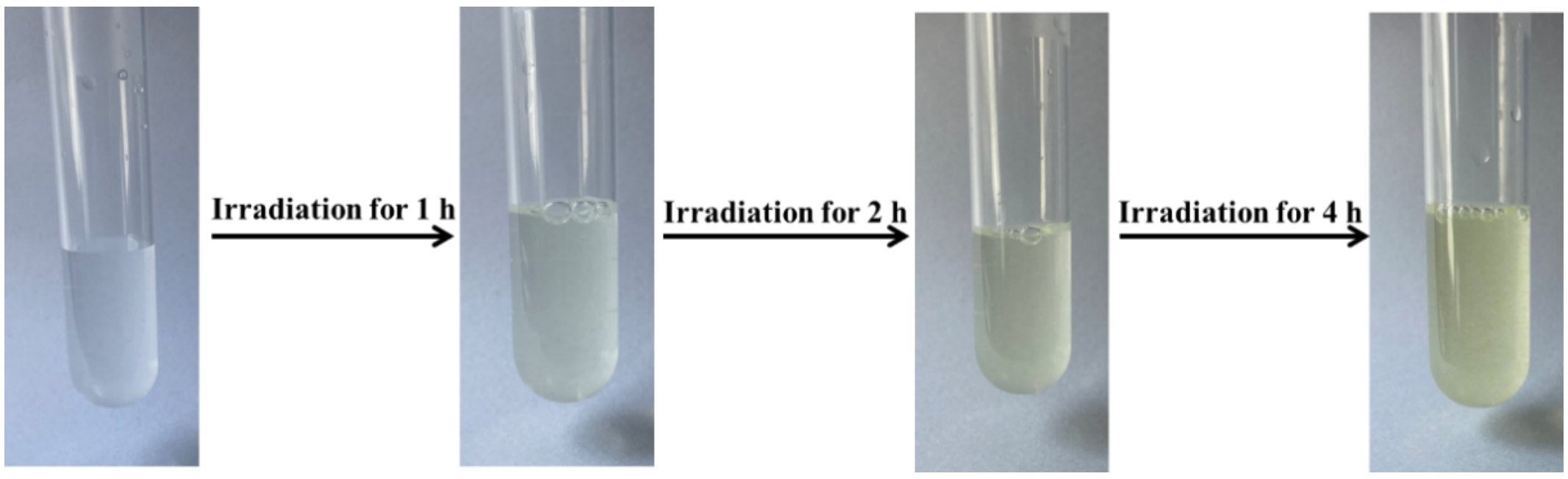

Supplementary Fig. 24 | Indicator-labeled reaction solutions with different H_2O_2 concentrations. Photographs of the reaction solutions at different irradiation time in the presence of potassium hydrogen phthalate and potassium iodide indicators.

Detection of O_2 during water oxidation. Apart from H_2O_2, oxygen (O_2) was also detected during the PCO_2RR over **HNNU-Se**. According to gas chromatography analysis, the O_2 amount increased gradually with increase in reaction time (Supplementary Fig. 25). Notably, the O_2 production didn't compromise the generation of H_2O_2, suggesting that both O_2 and H_2O_2 are oxidative products of water during the PCO_2RR over **HNNU-Se**.

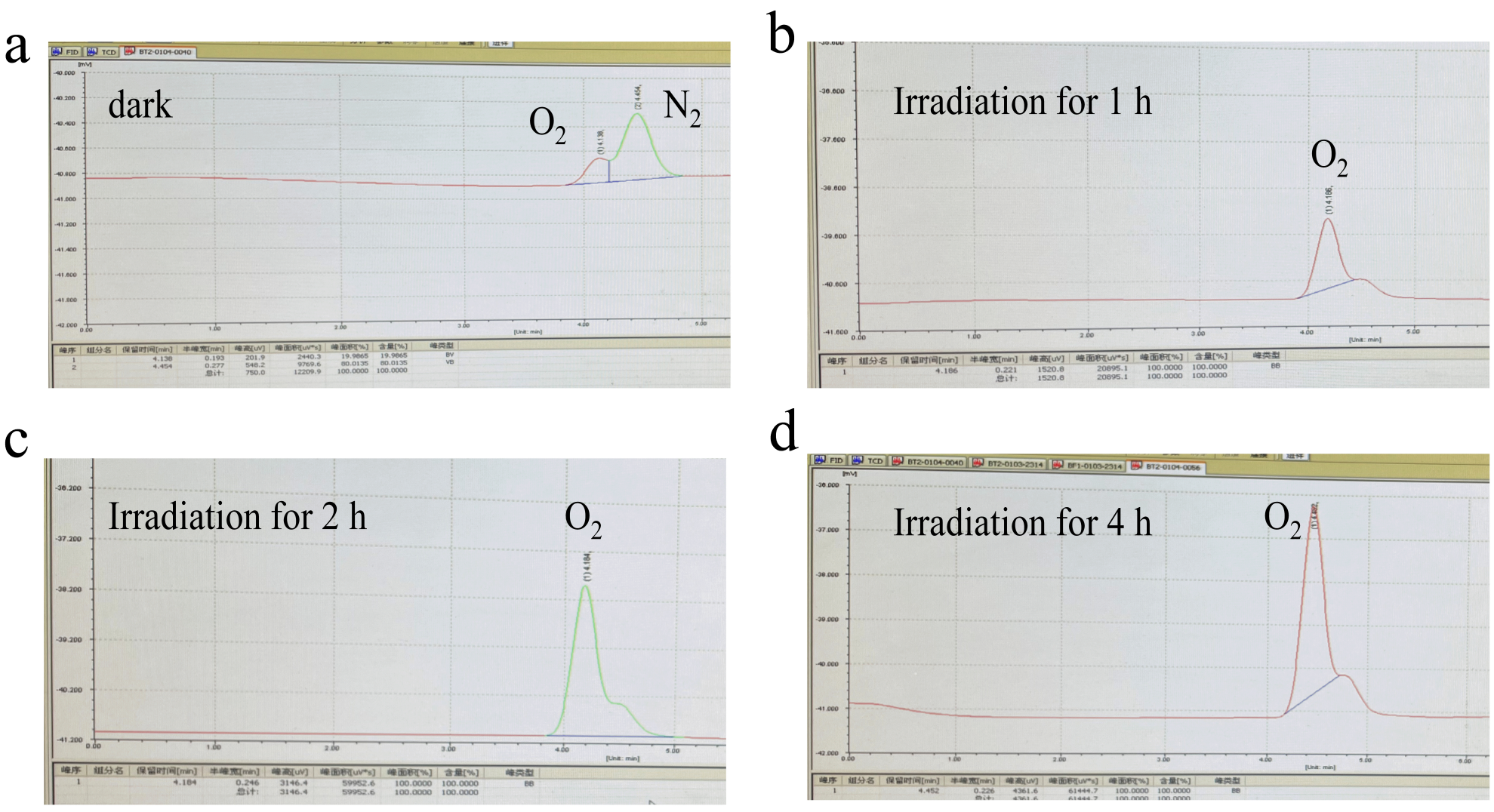

Supplementary Fig. 25 | GC analysis of O_2 evolution over HNNU-Se. O_2 content measured by GC during CO_2 photoreduction over HNNU-Se for 0 h (a), 1 h (b), 2 h (c) and 4 h (d), respectively.

Comparison the activity of CO_2 reduction and O_2 reduction. To evaluate the competitive reduction of CO_2 and O_2, linear sweep voltammetry (LSV) measurements were conducted using an **HNNU-Se**-modified electrode in CO_2- or O_2-saturated Na_2SO_4 solution (0.5 M). As shown in Supplementary Fig. 26, the onset of cathodic current occurs at -0.73 and -0.87 V vs RHE, KCl_{sat} for CO_2- or O_2-saturated electrolyte solution, respectively. It suggests that CO_2 reduction is easier to be triggered than O_2 reduction in the aqueous photocatalytic system. Moreover, only a slight

increase in current density is observed in O_2-saturated solution at potentials more negative than -0.87 V, suggesting that O_2 reduction rarely occurs in the aqueous photocatalytic system. In contrast, the current density in CO_2-saturated solution increases markedly with more negative potentials. These observations demonstrate that CO_2 reduction predominates over O_2 reduction in the aqueous photocatalytic system, in agreement with the properties of **HNNU-X**.

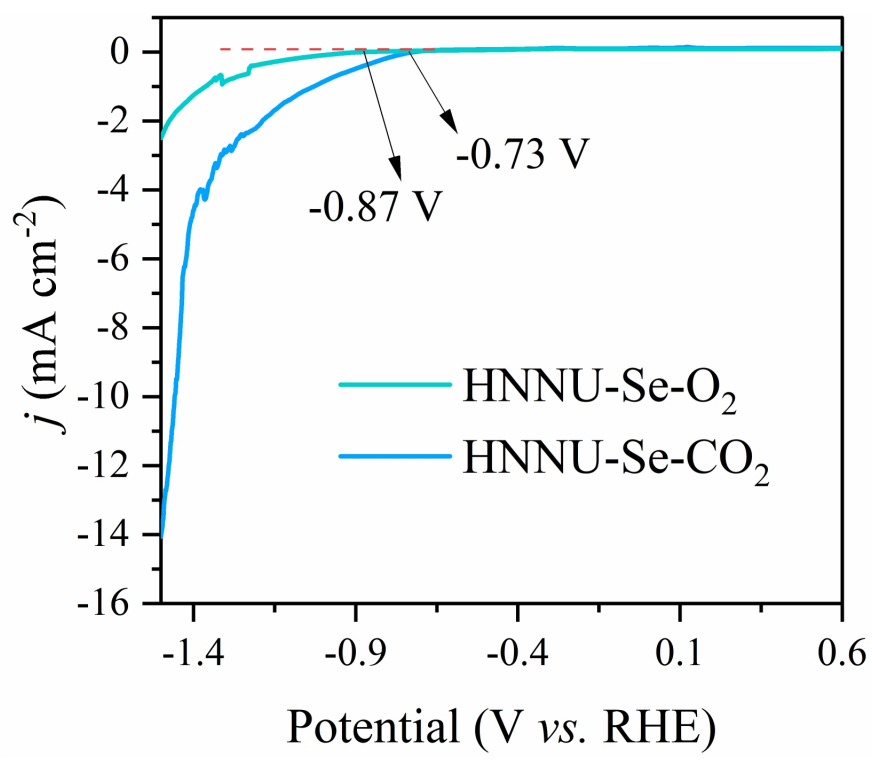

Supplementary Fig. 26 | LSV curve. LSV curves at HNNU-Se-modified electrodes (The tests were conducted in a CO_2- or O_2-saturated Na_2SO_4 aqueous solution (0.5 M) under visible light irradiation).

*3.6 Characterization of the reused **HNNU-Se (X= O, S, Se)***

*Regeneration of **HNNU-X.*** 50 mg used **HNNU-X** was dissolved in 20 mL DMF to get a clear solution. The supernatant was taken and placed in a 50 mL glass tube. The vial was immersed in a beaker filled with acetone, sealed, and allowed to undergo vapor diffusion at room temperature. The mixture stands at room temperature for 36 h to afford crystalline **HNNU-X**. XRD analysis shows that these **HNNU-X** materials can be self-healing through simple recrystallization (Supplementary Fig. 27). No significant change in morphology of **HNNU-X** took place even after reusing six times in water, as shown in SEM images (Supplementary Fig. 28).

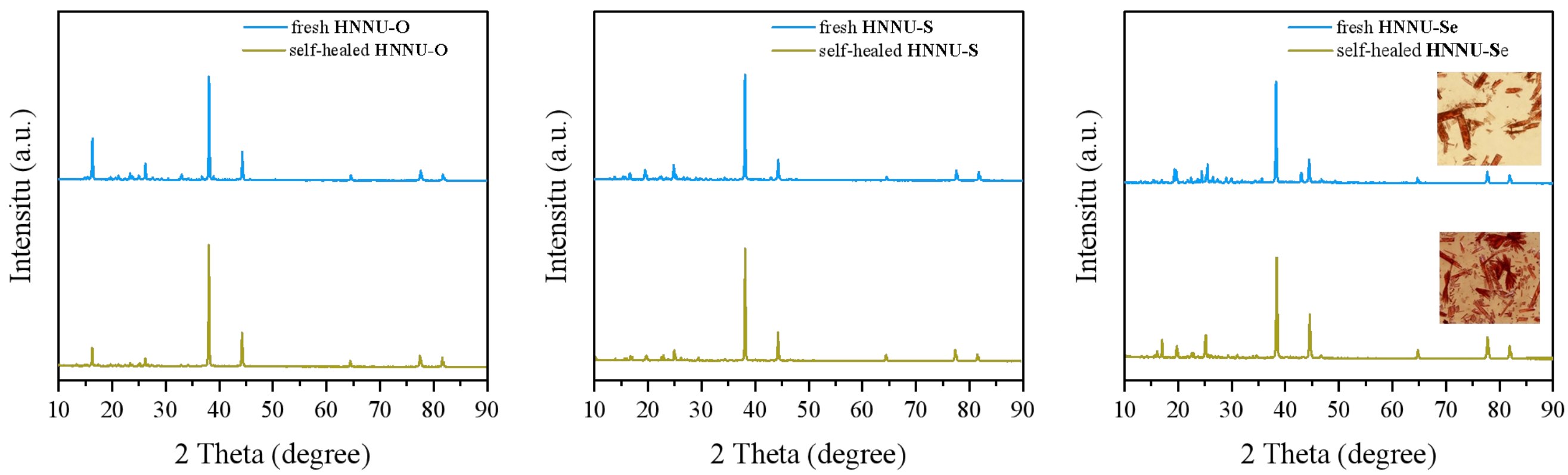

Supplementary Fig. 27 | Self-healed capability of HNNU-X. PXRD patterns of fresh HNNU-X (X= O, S, Se) and self-healed HNNU-X (X= O, S, Se) after the 1st reuse.

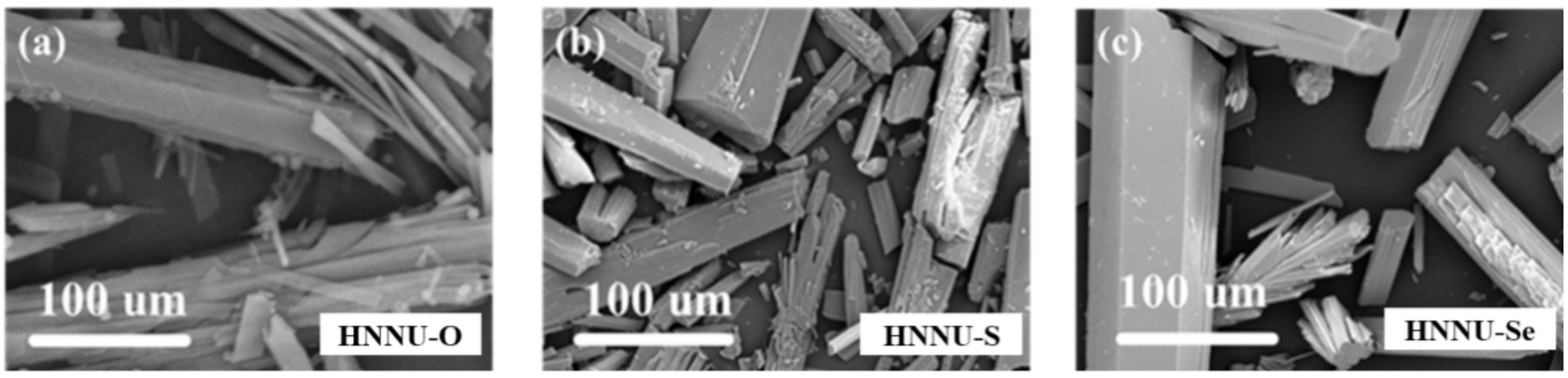

Supplementary Fig. 28 | SEM images of reused HNNU-X. SEM images of HNNU-O (a), HNNU-S (b), and HNNU-Se (c) after the 7th reuses.

Section S4. Investigation of mechanism

*4.1 Characterization of protonated **HNNU-X** during the PCO_2R reaction process*

HNNU-X undergoes gradual protonation during the reaction as can be seen from the color change (Supplementary Fig. 29).

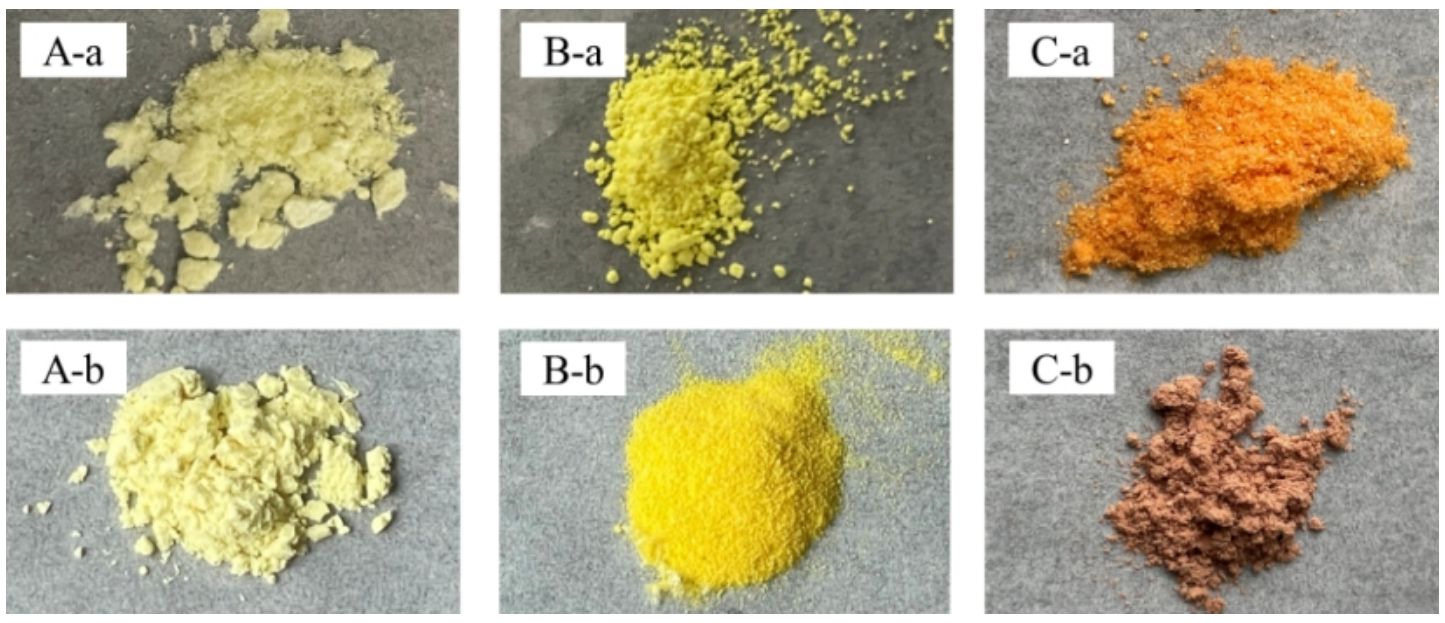

Supplementary Fig. 29 | Photographs of HNNU-X before and after PCO_2R. Photographs of HNNU-O (A), HNNU-S (B) and HNNU-Se (C) pristine (a) and upon PCO_2R reaction (b).

Zeta potential measurements were performed to gain insights into the protonation ability of **HNNU-X** during the reaction. As shown in Supplementary Fig. 30, the zeta potential of typical **HNNU-Se** changes from -0.78 to 7.10 mV after the catalytic process, indicative of the protonated state of **HNNU-Se** during the PCO_2RR reaction process.

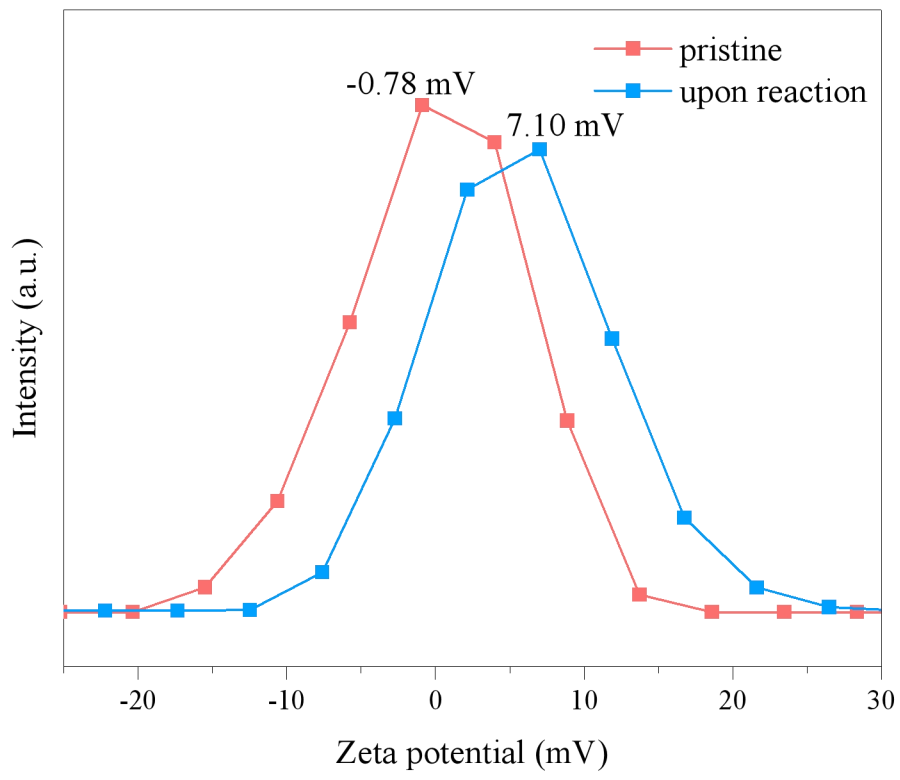

Supplementary Fig. 30 | Zeta-potential analysis. Zeta potential of HNNU-Se pristine and upon the PCO_2RR reaction.

Fourier-transform infrared (FTIR) spectroscopy reveals that the protonation occurs at the N atom in $[SeCN]^-$ anion during the PCO_2RR reaction process (Supplementary Fig. 31). The pristine **HNNU-Se** displayed a pronounced stretching vibration peak of C=N at 1642 cm^{-1}. As the reaction progressed, the characteristic C=N stretching vibration peak gradually attenuated, while a new peak (at 1652 cm^{-1}) corresponding to the $^{+}$H-N=C stretching vibration emerged and intensified.[14]

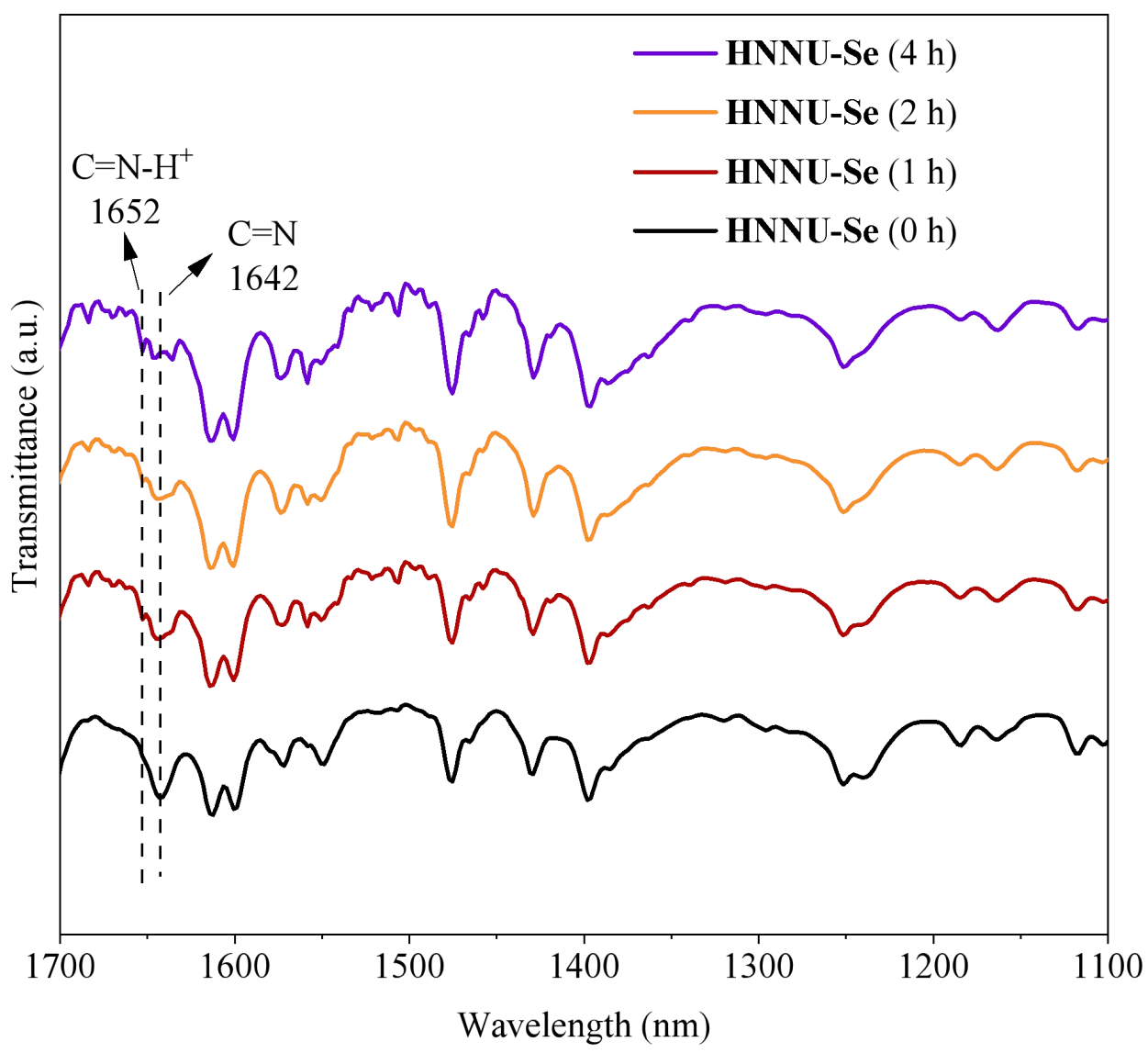

Supplementary Fig. 31 | FTIR analysis of HNNU-Se after reaction. FTIR spectra of HNNU-Se after different reaction time periods of 0 h, 1 h, 2 h and 4 h.

X-ray photoelectron spectroscopy (XPS) was performed to gain insights into the protonation state of **HNNU-Se** during the reaction. For the pristine **HNNU-Se**, the N 1*s* XPS spectrum was successfully deconvoluted into two peaks. The peak at 399.48 eV (α peak) was assigned to pyridinic N, while the one at 398.16 eV (β peak) corresponded to N=C=Se (Supplementary Fig. 32a).[15] Notably, the area ratio of these two peaks, approximately 3:2 (α:β), precisely matched the ratio of pyridinic N to $NCSe^-$ anions determined by single-crystal structure analysis. After reaction, both N 1*s* peaks exhibited the positive shift in binding energy, indicating a more electron-deficient chemical environment for nitrogen atoms. Furthermore, a new peak emerged at 400.69 eV (γ peak), which was attributed to the protonated $^+$N(H)=C=Se species derived from N=C=Se, providing direct evidence of the protonation process (Supplementary Fig. 32b).[16] Quantitative analysis of the nitrogen species revealed that the ratio of α:β decreased from 3:2 to 3:1 after reaction, whereas the ratio of α:(β + γ) remained constant at 3:2. These observations suggested that the protonation occurs at the N atom in $[XCN]^-$ anion of **HNNU-Se** during the reaction. Consistent with the N 1*s* results, **HNNU-Se** show positive shifts of Se $3d_{3/2}$ and Se $3d_{5/2}$ peaks after reaction (Supplementary Fig. 32c vs. d), due to the protonation of nitrogen atoms in $[SeCN]^-$ anion. Furthermore, a π-π* satellite peak appears in Se 3*d* XPS spectrum upon reaction, suggesting the enhanced conjugated domain relative to the pristine **HNNU-Se**.[17-19]

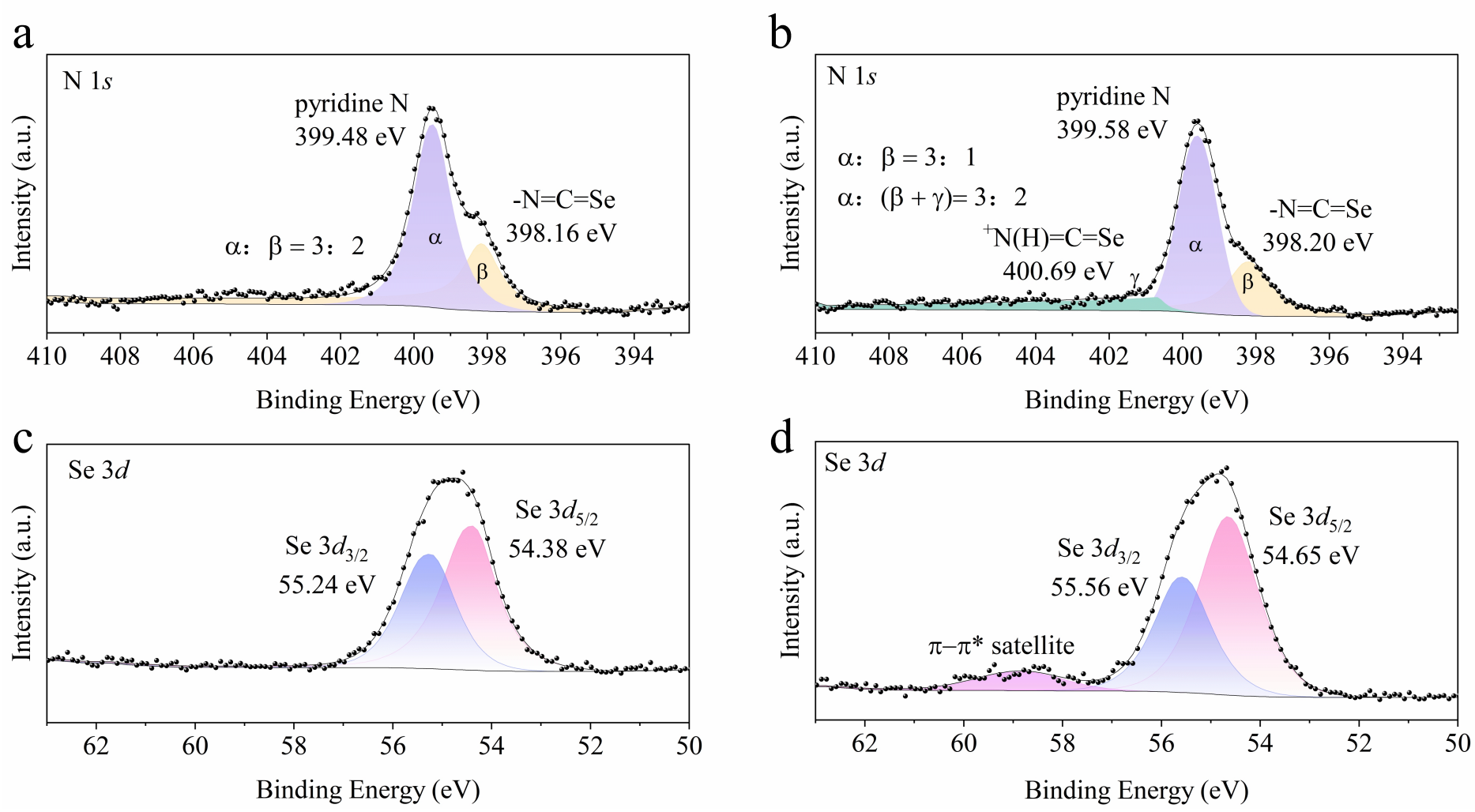

Supplementary Fig. 32 | XPS analysis of HNNU-Se before and after reaction. N 1*s* and Se 3*d* XPS spectra of HNNU-Se pristine (a), (b) and upon (c), (d) the reaction.

X-ray photoelectron spectroscopy (XPS) measurements were conducted to further probe the protonation ability of **HNNU-X** (X = O, S, Se) (Supplementary Fig. 33). Prior to analysis, the samples were pre-protonated in dilute hydrochloric acid. The N 1*s* spectra can be deconvoluted into three components at ~398 eV, ~399 eV, and ~405 eV, assigned to N=C=X, pyridinic N, and protonated N(H^+)=C=X species, respectively. According to deconvolution of the peak areas, N(H^+)=C=X/N=C=X ratios are calculated to be 0.76, 0.29, and 0.16 and for **HNNU-Se** (Supplementary Fig. 33a), **HNNU-S** (Supplementary Fig. 33b), and **HNNU-O** (Supplementary Fig. 33c), respectively. These results indicate a progressively decreased protonation degree of [XCN]⁻ anions in the order **HNNU-Se** > **HNNU-S** > **HNNU-O**.

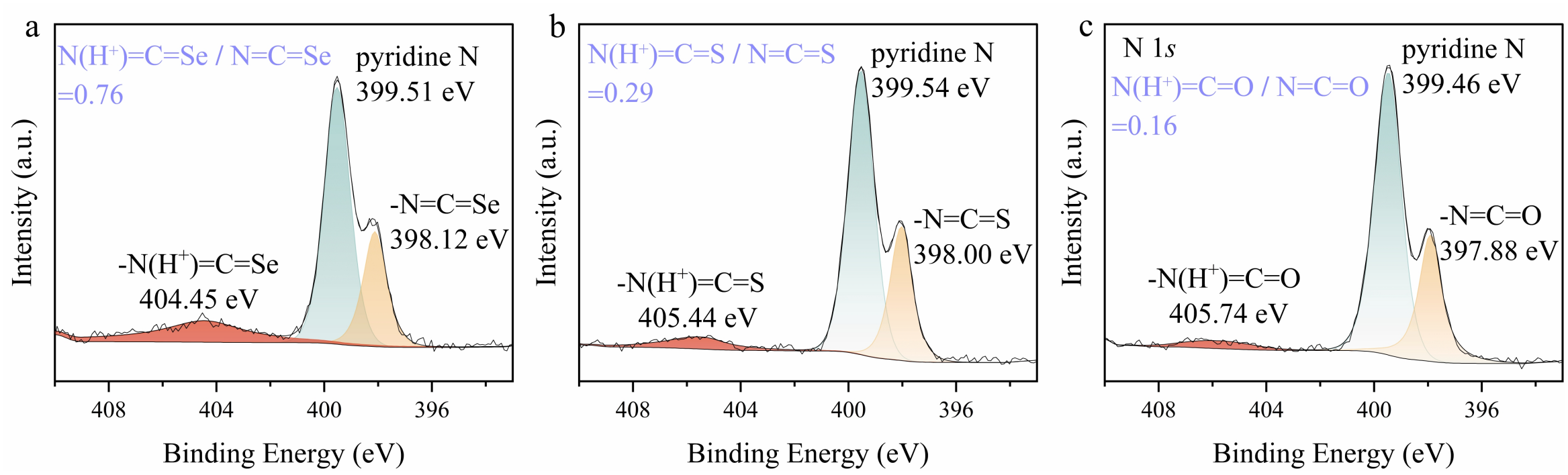

Supplementary Fig. 33 | Protonation ability investigation over HNNU-X. N 1*s* XPS spectra of pre-acidified HNNU-Se (a), HNNU-S (b) and HNNU-O (c).

Owing to the protonation of [SeCN]⁻ anion, **HNNU-Se** shows the higher water affinity with a water contact angle decreased from 55° to 36° (Supplementary Fig. 34). It ensures more water molecules adsorb on **HNNU-Se** surface,

creating a proton-rich microenvironment for efficient proton conduction (Supplementary Fig. 35).

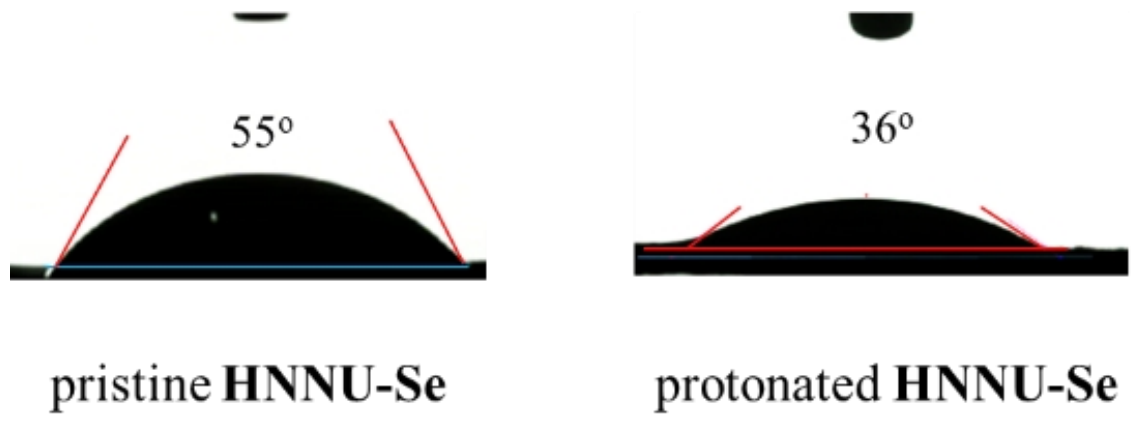

Supplementary Fig. 34 | Surface wettability of HNNU-Se before and after reaction. Water contact angle (WCA) of HNNU-Se pristine and upon the PCO_2R reaction.

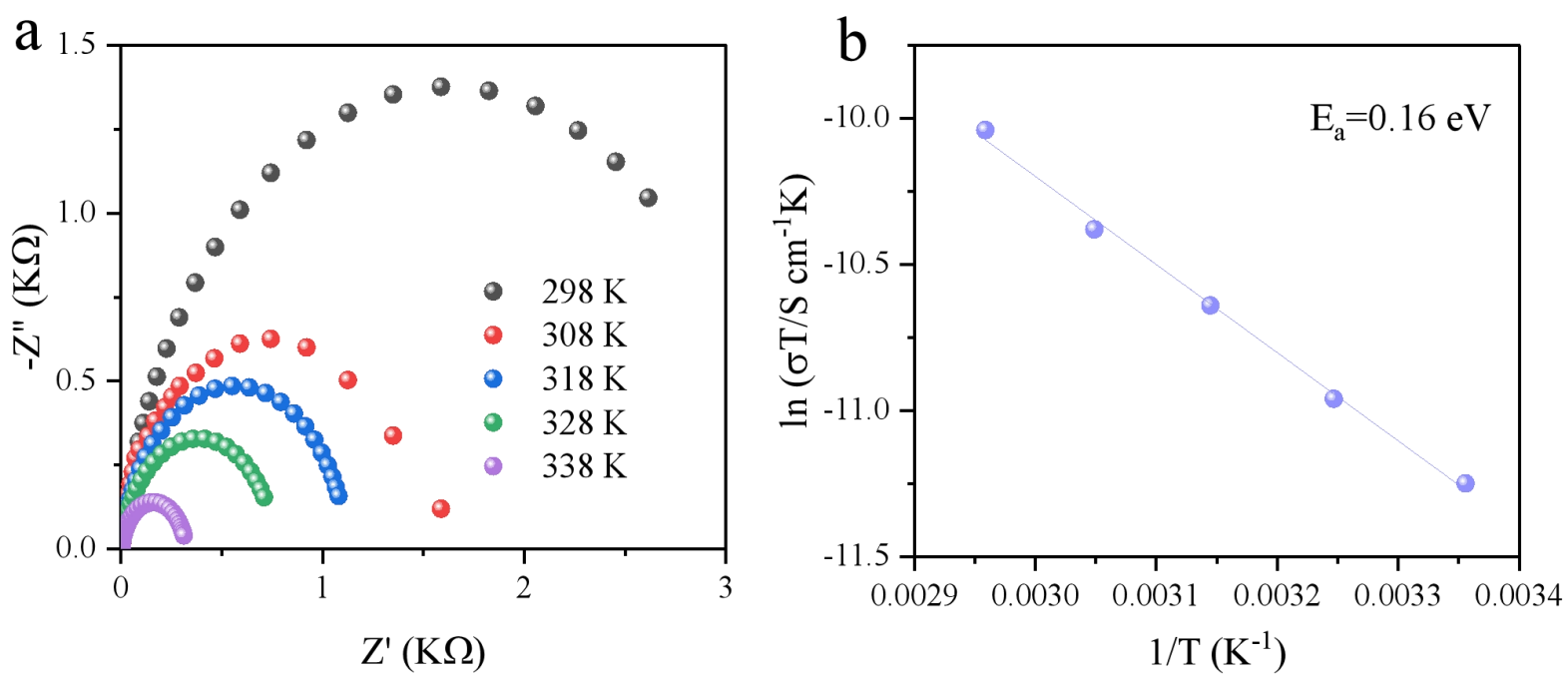

Supplementary Fig. 35 | Proton conductivity analysis of protonated HNNU-Se. Proton conductivities of protonated HNNU-Se at different temperatures and 99% of relative humidity (a) and Arrhenius plots (b).

*4.2 EXAFS R-space fitting curves of **HNNU-Se** at different states*

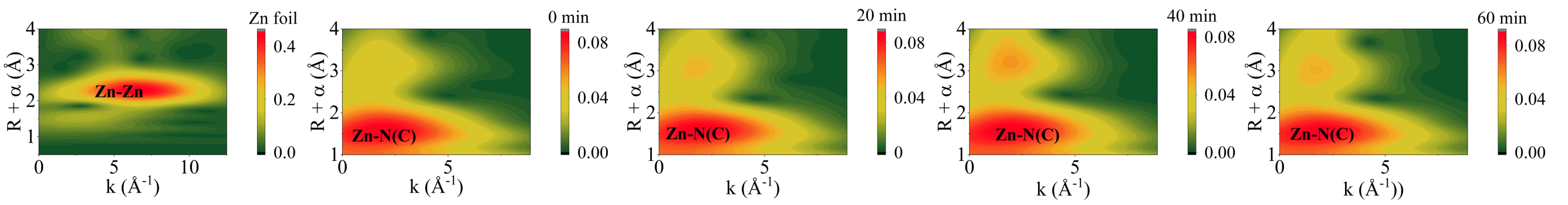

Supplementary Fig. 36 | Wavelet transforms of Zn K-edge EXAFS signals. WTs for the k^3-weighted Zn K-edge EXAFS signals under different states.

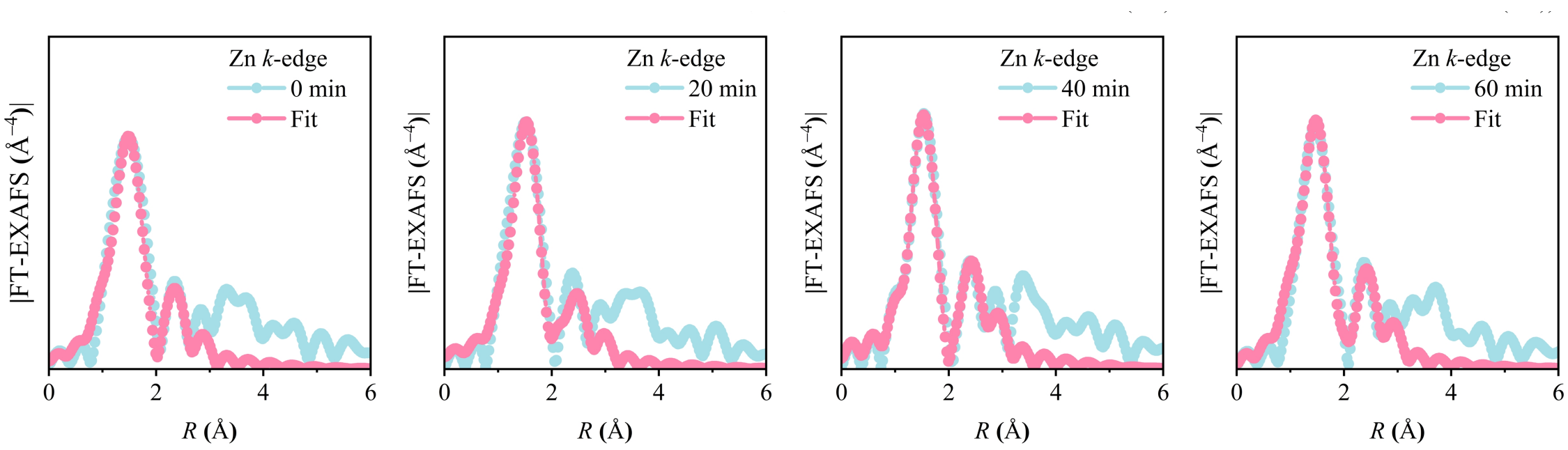

Supplementary Fig. 37 | Local coordination analysis of Zn sites in HNNU-Se. Zn K-edge EXAFS R-space fitting

curves of HNNU-Se at different states (The spectrum labeled “0 min” was obtained immediately after lighting, the spectra labeled “20 min”, “40 min” and “60 min” were collected 20, 40 and 60 min after illumination, respectively).

Supplementary Table 6. Fitted changes in coordination number (CN) of Zn-N shell at different states.

Sample	Path	CN[a]	R (Å)[b]	σ^2 (Å^2)[c]	E_0 (eV)[d]	R factor[e]
0 min	Zn-N/C	5.2	2.02	0.008	0.5	0.019
20 min	Zn-N/C	5.6	2.08	0.005	2.00	0.019
40 min	Zn-N/C	5.9	2.09	0.008	1.06	0.009
60 min	Zn-N/C	5.4	2.02	0.010	3.5	0.020

[a] CN.: coordination number.
[b] R: the distance between absorber and backscatter atoms.
[c] $\sigma 2$: Debye-Waller factors.
[d] ΔE: the inner potential correction.
[e] R factor: goodness of fit.

4.3 Density functional theoretical (DFT) calculation of the reaction mechanism

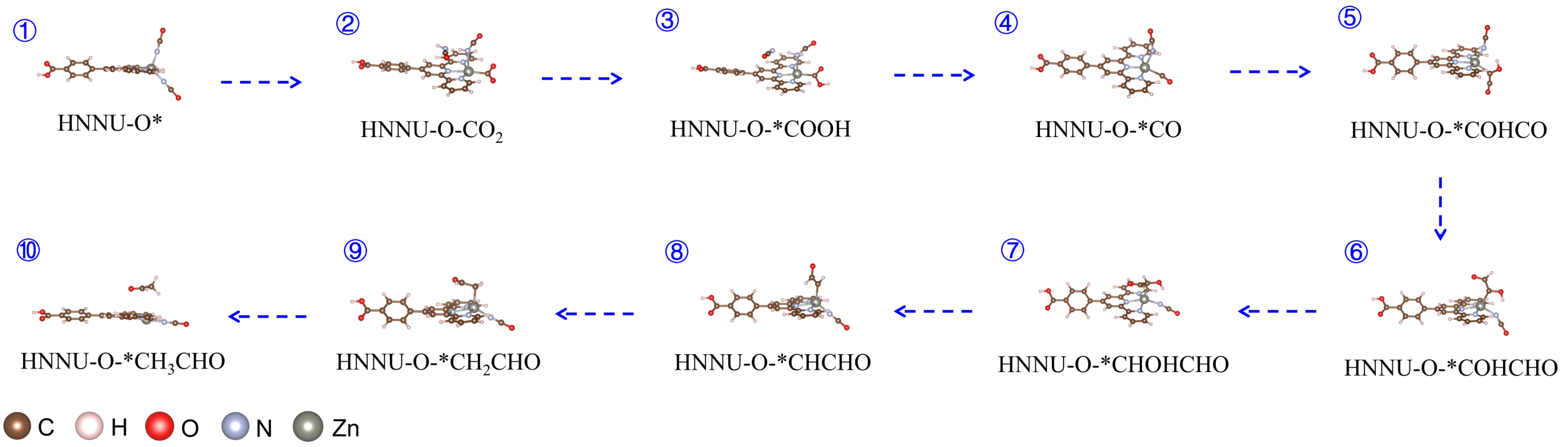

Supplementary Fig. 38 | DFT-optimized intermediates for CO_2 reduction on HNNU-O. DFT optimized structure models for every step of catalytic CO_2 reduction on HNNU-O.

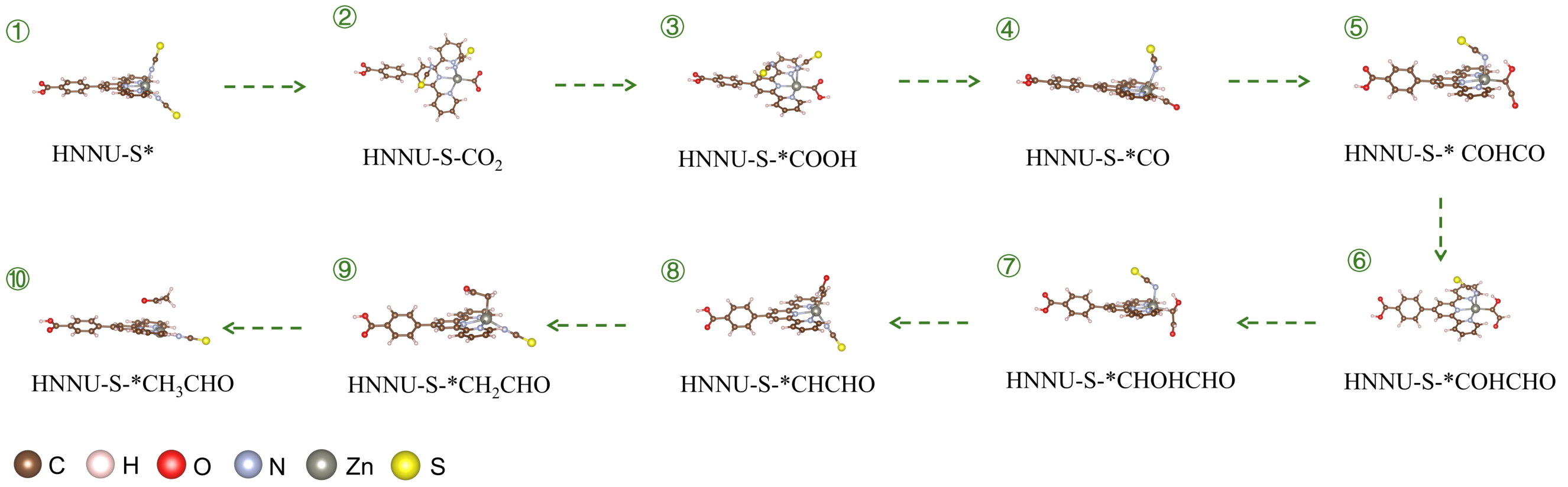

Supplementary Fig. 39 | DFT-optimized intermediates for CO_2 reduction on HNNU-S. DFT optimized structure models for every step of catalytic CO_2 reduction on HNNU-S.

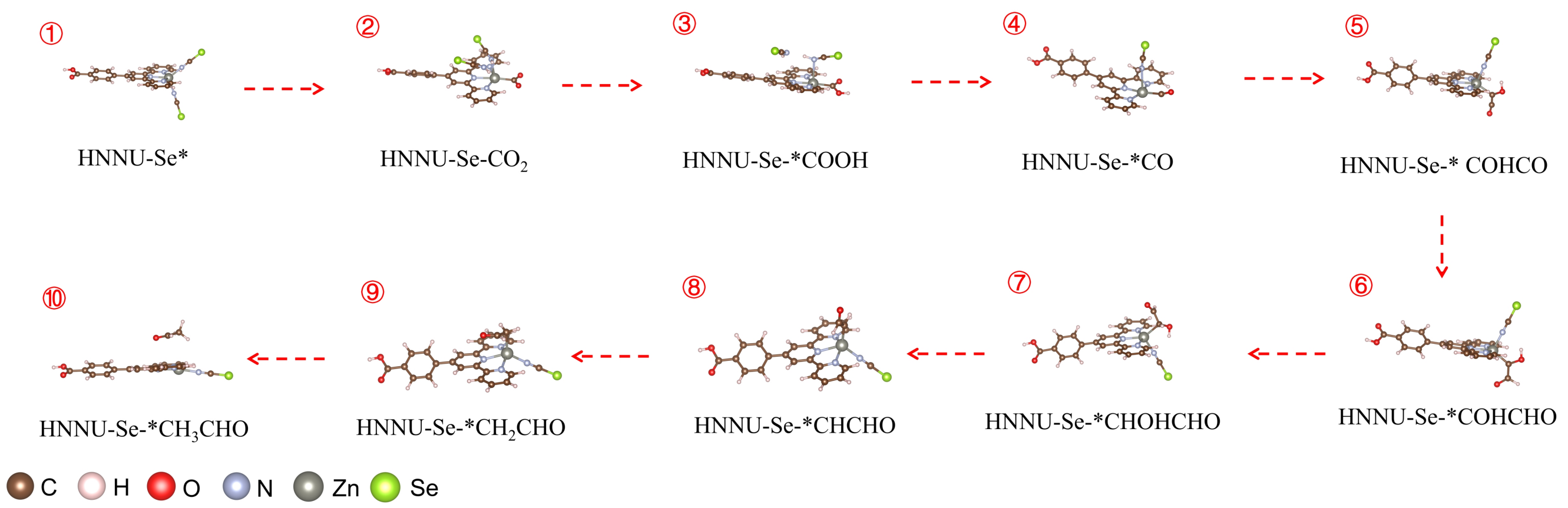

Supplementary Fig. 40 | DFT-optimized intermediates for CO_2 reduction on HNNU-Se. DFT optimized structure models for every step of catalytic CO_2 reduction on HNNU-Se.

Supplementary Table 7. Gibbs free energies (G) of the molecules obtained form the electronic energies (E) with free-energy corrections (ΔGcorr (eV)), together with their charges and spin multiplicities.

molecule	E (eV)	ΔGcorr (eV)	G (eV)	Charge	Spin multiplicity
CO	-3081.03	-0.38	-3081.41	0	1
CO_2	-5127.87	-0.24	-5128.11	0	1
H_2	-31.87	-0.04	-31.91	0	1
H_2O	-2077.50	0.06	-2077.44	0	1
OCNH	-4586.54	0.07	-4586.47	0	1
SCNH	-13373.26	-0.08	13373.35	0	1

SeCNH	-67883.30	-0.20	67883.50	0	1

Supplementary Table 8. Gibbs free energies (G) of intermediates bound to **HNNU-O** obtained form the electronic energies (E) with free-energy corrections (ΔGcorr), together with their charges and spin multiplicities.

Intermediate	E (eV)	ΔGcorr (eV)	G (eV)	Charge	Spin multiplicity
HNNU-O	-89189.33	8.21	89181.12	0	1
HNNU-O-CO_2	-94317.47	8.39	94309.09	0	1
HNNU-O-COOH	-94334.05	8.67	94325.37	0	2
HNNU-O-CO	-87683.48	7.76	87675.72	0	1
HNNU-O-COHCO	-90766.36	8.12	90758.24	0	1
HNNU-O-CHO	-87700.36	8.06	87692.31	0	2
HNNU-O-COH	-87697.52	8.03	87689.49	0	2
HNNU-O-COHCHO	-90782.75	8.42	90774.33	0	2
HNNU-O-CHOHCHO	-90799.96	8.76	90791.20	0	1
HNNU-O-CHCHO	-88736.89	8.23	88728.66	0	2
HNNU-O-CH_2CHO	-88755.05	8.67	88746.39	0	1
HNNU-O-CH_2CH_2O	-88769.76	8.85	88760.91	0	2
HNNU-O-CH_2CHOH	-88770.50	8.86	88761.65	0	2
HNNU-O-CH_3CHO	-88771.15	8.86	88762.29	0	2

Supplementary Table 9. Gibbs free energies (G) of intermediates bound to **HNNU-S** obtained form the electronic energies (E) with free-energy corrections (ΔGcorr), together with their charges and spin multiplicities.

Intermediate	E (eV)	ΔGcorr (eV)	G (eV)	Charge	Spin multiplicity
HNNU-S	106762.61	8.00	106754.61	0	1
HNNU-S-CO_2	111890.78	8.25	111882.53	0	1
HNNU-S-COOH	111907.31	8.44	111898.87	0	2
HNNU-S-CO	-96470.13	7.68	-96462.45	0	1
HNNU-S-COHCO	-99553.26	8.08	-99545.19	0	1
HNNU-S-CHO	-96486.85	8.13	-96478.73	0	2
HNNU-S-COH	-96484.13	7.93	-96476.20	0	2

HNNU-S-COHCHO	-99569.66	8.33	-99561.33	0	2
HNNU-S-CHOHCHO	-99586.78	8.69	-99578.09	0	1
HNNU-S-CHCHO	-97523.86	8.14	-97515.72	0	2
HNNU-S-CH_2CHO	-97542.00	8.59	-97533.41	0	1
HNNU-S-CH_2CH_2O	-97556.79	8.74	-97548.06	0	2
HNNU-S-CH_2CHOH	-97557.39	8.76	-97548.63	0	2
HNNU-S-CH_3CHO	-97558.03	8.77	-97549.26	0	2

Supplementary Table 10. Gibbs free energies (G) of intermediates bound to **HNNU-Se** obtained form the electronic energies (E) with free-energy corrections (ΔGcorr), together with their charges and spin multiplicities.

Intermediate	E (eV)	ΔGcorr (eV)	G (eV)	Charge	Spin multiplicity
HNNU-Se	215782.59	7.89	215774.70	0	1
HNNU-Se-CO_2	220910.82	8.12	220902.70	0	1
HNNU-Se-COOH	220927.29	8.33	220918.96	0	2
HNNU-Se-CO	150980.16	7.64	150972.52	0	1
HNNU-Se-COHCO	154063.43	7.98	154055.45	0	1
HNNU-Se-CHO	150996.98	7.89	150989.09	0	2
HNNU-Se-COH	150994.12	7.86	150986.25	0	2
HNNU-Se-COHCHO	154079.80	8.27	154071.53	0	2
HNNU-Se-CHOHCHO	154096.98	8.64	154088.34	0	1
HNNU-Se-CHCHO	152034.00	8.09	152025.91	0	2
HNNU-Se-CH_2CHO	152052.12	8.52	152043.60	0	1
HNNU-Se-CH_2CH_2O	152066.86	8.64	152058.22	0	2
HNNU-Se-CH_2CHOH	152067.52	8.76	152058.76	0	2
HNNU-Se-CH_3CHO	152068.14	8.73	152059.41	0	2

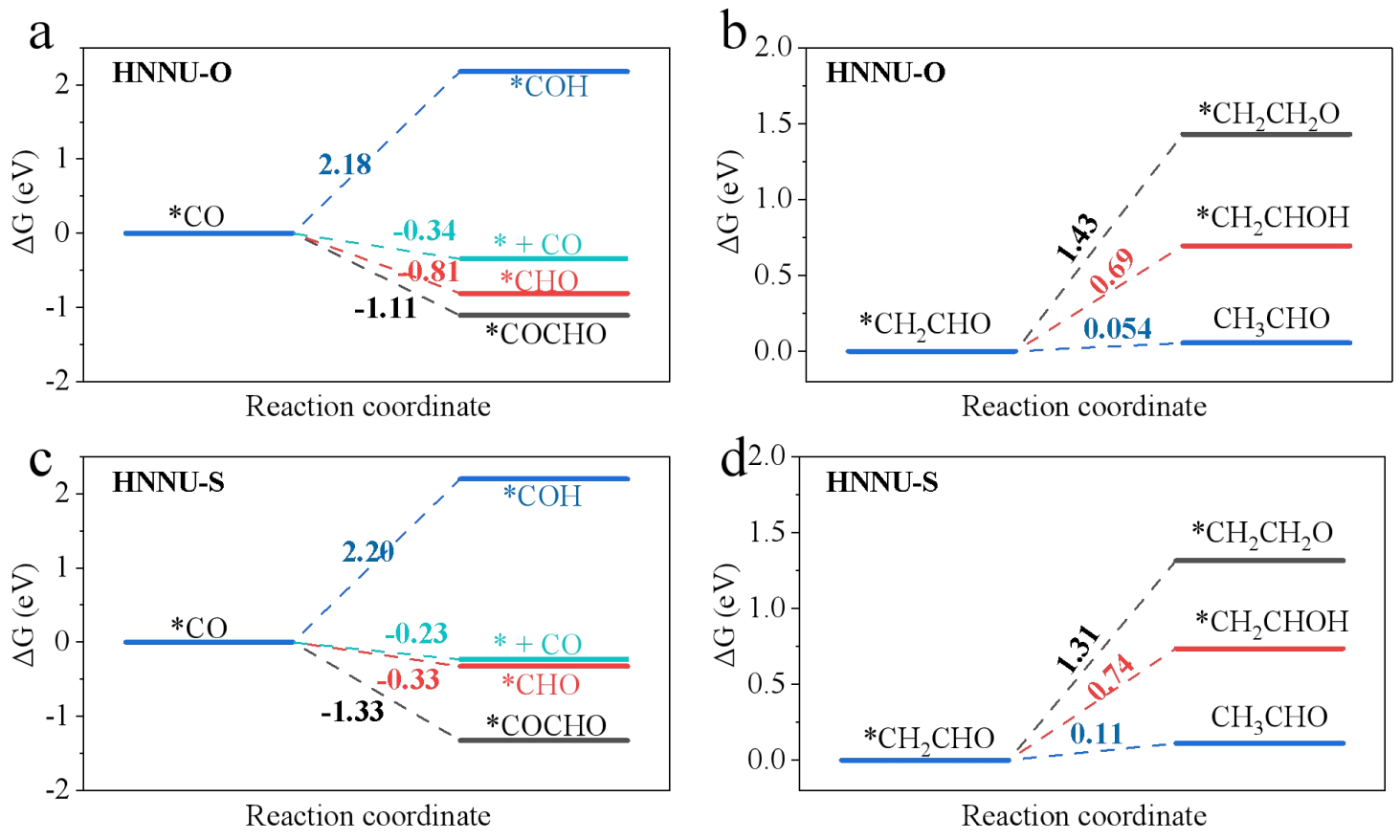

Supplementary Fig. 41 | Gibbs free-energy diagrams for key intermediate transformations on HNNU-O and HNNU-S. Gibbs free energy diagrams for the transformation of *CO into different intermediates on HNNU-O (a) and HNNU-S (c). Gibbs free energy diagrams for the transformation of *CH_2CHO into different intermediates on HNNU-O (b) and HNNU-S (d).

Supplementary References

1. Wu, K. *et al.* Linker engineering for reactive oxygen species generation efficiency in ultra-stable nickel-based metal-organic frameworks. *J. Am. Chem. Soc.* **145**, 18931-18938 (2023).
2. Guo, C. *et al.* Atomic heterointerface engineering and nonequilibrium carrier dynamics for enhanced photocatalysis in halide perovskite/MoS_2 systems. *Angew. Chem. Int. Ed.* **64**, e202506436 (2025).
3. Meng, K. et al. Interfacial charge transfer in ZnO/COF S-scheme photocatalyst via Zn-N bond. *Adv. Mater.* **37**, 2505088 (2025).
4. Liu, Q. et al. Regulating the *OCCHO intermediate pathway towards highly selective photocatalytic CO_2 reduction to CH_3CHO over locally crystallized carbon nitride. *Energy Environ. Sci.* **15**, 225-233 (2022).
5. Lei, J. et al. Visible Light-Driven Acetaldehyde Production from CO_2 and H_2O via Synergistic Vacancies and Atomically Dispersed Cu Sites. *Angew. Chem. Int. Ed.* **64**, e202422667 (2025).
6. Shown, I. et al. Carbon-doped SnS_2 nanostructure as a high-efficiency solar fuel catalyst under visible light. *Nat. Commun.* **9**, 169 (2018).
7. Gao, W. et al. Vacancy-defect modulated pathway of photoreduction of CO_2 on single atomically thin $AgInP_2S_6$ sheets into olefiant gas. *Nat. Commun.* **12**, 4747 (2021).
8. Yu, F., Jing, X., Wang, Y., Sun, M. & Duan, C. Hierarchically porous metal–organic framework/MoS_2 interface for selective photocatalytic conversion of CO_2 with H_2O into CH_3COOH. *Angew. Chem. Int. Ed.* **60**, 24849-24853 (2021).

9 Zhu, S. et al. Selective CO_2 photoreduction into C_2 product enabled by charge-polarized metal pair sites. *Nano Lett.* **21**, 2324-2331 (2021).

10 Das, R., Das, K., Ray, B., Vinod, C. P. & Peter, S. C. Green transformation of CO_2 to ethanol using water and sunlight by the combined effect of naturally abundant red phosphorus and Bi_2MoO_6. *Energy Environ. Sci.* **15**, 1967-1976 (2022).

11 Shi, H. et al. Atomically dispersed indium-copper dual-metal active sites promoting C−C coupling for CO_2 photoreduction to ethanol. *Angew. Chem. Int. Ed.* **61**, e202208904 (2022).

12 Ou, H. et al. Atomically dispersed Au-assisted C–C coupling on red phosphorus for CO_2 photoreduction to C_2H_6. *J. Am. Chem. Soc.* **144**, 22075-22082 (2022).

13 Cheng, J. *et al.* Molecularly tunable heterostructured Co-polymers containing electron-deficient and -rich moieties for visible-light and sacrificial-agent-free H_2O_2 photosynthesis. *Angew. Chem. Int. Ed.* **63**, e202406310 (2024).

14 Zhu, Q., Shi, L., Li, Z., Li, G. & Xu, X. Protonation of an imine-linked covalent organic framework for efficient H_2O_2 photosynthesis under visible light up to 700 nm. *Angew. Chem. Int. Ed.* **63**, e202408041 (2024).

15 Li, H. *et al.* Thiocyanate coordination regulates the interlayer ammonium ion storage structure of molybdenum disulfide. *ACS Energy Lett.* **10**, 168-176 (2025).

16 Dong, P. *et al.* Structural variation and charge-transfer dynamics of protonated β-ketoenamine-linked covalent organic framework for boosted photocatalytic H_2 evolution. *ACS Catal.* **15**, 18138-18154 (2025).

17 Feng, Y., Xiao, Y., Huang, X. & Li, W. Locking metal-organic framework linkers to mesoporous nanosheets via π–π conjugation for gas separation. *Chem. Mater.* **37**, 8403-8411 (2025).

18 Liu, X. *et al.* Biomimetic gourd-vine supramolecular engineering for high-performance organic cathode through dual-mode of anion coordination and conjugated redox activation. *Angew. Chem. Int. Ed.* **64**, e202511229 (2025).

19 Chen, J. *et al.* Crosslinked conjugated interlayer network for efficient and stable wide-bandgap perovskite and all-perovskite tandem solar cells. *Adv. Energy Mater.* **15**, 2500309 (2025).
