# Supplementary material for: Atmospheric CO₂-to-acetaldehyde artificial photosynthesis in metallo hydrogen-bonded organic frameworks
Source: Nat Commun. 2026 Jul 12;17:8583. doi: 10.1038/s41467-026-75384-z (PMC13486723; doi:10.1038/s41467-026-75384-z)
Supplement: Supplementary file 2 [file 41467_2026_75384_MOESM2_ESM.pdf]

Description of Additional Supplementary File

Supplementary Data 1 | Atomic coordinates of optimized computational models.

This file contains the atomic coordinates (XYZ format) of the following DFT-optimized structures:

1, SeCNH

2, SCNH

3, OCNH

4, H_2O

5, H_2

6, CO_2

7, CO

8, HNNU-Se_initial_structure

9, HNNU-Se_protonated_structure

10, HNNU-Se+CO_2

11, HNNU-Se+COOH

12, HNNU-Se+CO

13, HNNU-Se+COHCO

14, HNNU-Se+COHCHO

15, HNNU-Se+CHOHCHO

16, HNNU-Se+CHCHO

17, HNNU-Se+CH_2CHO

18, HNNU-Se+CH_3CHO

19, HNNU-S_initial_structure

20, HNNU-S_protonated_structure

21, HNNU-S+CO_2

22, HNNU-S+COOH

23, HNNU-S+CO

24, HNNU-S+COHCO

25, HNNU-S+COHCHO

26, HNNU-S+CHOHCHO

27, HNNU-S+CHCHO

28, HNNU-S+CH_2CHO

29, HNNU-S+CH_3CHO

30, HNNU-O_initial_structure

31, HNNU-O_protonated_structure

32, HNNU-O+CO_2

33, HNNU-O+COOH

34, HNNU-O+CO

35, HNNU-O+COHCO

36, HNNU-O+COHCHO

37, HNNU-O+CHOHCHO

38, HNNU-O+CHCHO

39, HNNU-O+CH_2CHO

40, HNNU-O+CH_3CHO

41, HNNU-Se_single

42, HNNU-Se_single+H_2O.log

43, HNNU-Se_single+OH.log

44, HNNU-Se_single_protonated.log

45, HNNU-Se_single_protonated+H2O.log

46, HNNU-Se_single_protonated+OH.log

Each structure begins with a comment line indicating the structure name. Coordinates are given in angstroms (Å). Calculation level: B3LYP/DEF2SVPP. Energies are given in Hartree.
