# Supplementary material for: Atmospheric CO₂-to-acetaldehyde artificial photosynthesis in metallo hydrogen-bonded organic frameworks
Source: Nat Commun. 2026 Jul 12;17:8583. doi: 10.1038/s41467-026-75384-z (PMC13486723; doi:10.1038/s41467-026-75384-z)
Supplement: Supplementary file 3 [file 41467_2026_75384_MOESM4_ESM.pdf]

nature portfolio

Peer Review File

Atmospheric CO_2-to-Acetaldehyde: Artificial Photosynthesis in Metallo Hydrogen-Bonded Organic Frameworks

Corresponding Author: Professor Rong Tan

Parts of this Peer Review File have been redacted as indicated to remove third-party material.

This file contains all reviewer reports in order by version, followed by all author rebuttals in order by version.

Version 0:

Reviewer comments:

Reviewer #1

(Remarks to the Author)
This manuscript reports the presumed photocatalytic CO2 reduction to CH3CHO promoted by a hydrogen-bonded organic framework derived from Zn terpyridine with di cyanate ligands. Although the submission has several interesting points related to the preparation and characterization of the material, there are other crucial issues that have to be solved before a final decision on its publication is taken. Therefore, I recommend to revise the manuscript addressing the following comments before reassessment:

1. Acetaldehyde is far more reactive than CO2 and its formation on a photocatalytic process, particularly in the presence of O2, should always be taken cautiously and supported on firm grounds. This is not the present case. Figs. S14-17 corresponding to 1H NMR spectra should have recorded with a water suppression mode. In any case, the spectrum of the 13C labelled CH3CHO should have been presented.
2. Mass spectra of labelled products are useful only after chromatographic separation. The corresponding chromatograms should be presented in Fig. 5. Also, what is the peak in the MS at m/z 45 in Fig. 5d? Even more important the mass spectrum of shown in Fig. 5 e should be wrong. How is possible if the compound is CD3CDO have mass fragments of 1 amu if this molecule does not contain any element of amu 1? In addition, how acetaldehyde has been extracted from the aqueous phase should be indicated.1
3. All the discussion of the preferent CO2 adsorption over N2 or O2 adsorption has no sense. To start the comparison should be made at the relative concentration of these compounds in air, in which CO2 concentration is very low and moisture is present. In addition to be in considerable excess respect to CO2, O2 reduction is much more favorable than reduction of CO2.
4. The authors should provide the calculation made to determine a CO2 reduction rate to acetaldehyde of 557.1 $\mu mol.g^{-1}.h^{-1}$. It is unclear if air flow is passed through the photocatalyst suspended in water of if this number is achieved under static conditions, just by contact with air. In my calculations, converting all the CO2 present in one litter of air at atmospheric pressure will render only 12.5 mol of acetaldehyde. Because the authors are using 5 mg, this means that at full conversion the maximum rate can be 2500 $\mu mol.g^{-1}.h^{-1}$.. So to achieve the number reported in the paper, conversion should be over 20 %, something that is doubtful particularly considering the photoreactor used.
5. According to the band potential, HNNU-Se should not be able to oxidize H2O to ·OH. The authors propose an explanation about the CO2 reduction assistance to overcome the thermodynamics, but these arguments are not convincing and unsupported. Experimental section should indicate how these irradiations were made. If O2 is present, how do the authors rule out the obvious O2 reduction to superoxide resulting in H2O2? The manuscript does not indicate in many parts how the experiments were carried out, confusing the reader in many sections.
6. There is a contradiction between calculations suggesting an electron transfer from the electron rich SeCN- anion to the terpyridyl linker with formation of SeCN radical as indicated by Figure 4 and then in the XPS discussion to identify Zn2+ as the site receiving the electron and later in line 282 they indicate that the terpyridyl ligand gives one electron to Zn.
7. Furthermore, some of the discussion is based on XPS Zn core peak shifts of 0.06 eV or 0.15 eV. In my experience this is within the experimental error. In any case, the authors should indicate the error in their measurements? Furthermore, the XPS of S and Se are extremely noisy and suggesting that these ligands have been lost, probably replaced by oxygenated groups. Atomic proportion of Se and S should be given.
8. Related with the previous point, reusability studies should provide analytical data of the Se content upon reuse, both in the liquid and the solid phase.

9. Experimental section lacks necessary details. The authors should indicate how the ESI and Mott-Schottky measurements were performed and the electrolyte. Are the complexes stable under the electrochemical measurement conditions? The authors should analyze the electrolyte for the absence of S/Se derived from ligand leaching.
10. The photocatalytic reactor shown in Figure S13 is poorly designed and should be very inefficient. Considering that sunlight comes from the top, the fact that it is opaque means that direct light rays cannot not access. What is considered the surface area of this reactor? Also, the system is open to the air or there is an air flow through the liquid phase?

In conclusion, the paper does not provide convincing data of the formation of acetaldehyde and should be rejected. However, I would like to see the author's responses before a final decision.

Reviewer #2

(Remarks to the Author)
This paper reported the novel metalo hydrogen-bonded organic frameworks (MHOFs) photocatalysts, which enabled artificial photosynthesis of acetaldehyde from atmospheric CO2. Interestingly, such photocatalysis gave the unprecedented acetaldehyde production rate (557.1 $\mu mol.g^{-1}.h^{-1}$) with high electron-based selectivity (95%) in an outdoor environment (air, water, natural sunlight) without any sacrificial agents. It is very important in the area of energy and environment, and consequently fulfills the remit of Nature Communication. I recommend to publish this paper in Nature Communication after addressing the following issues.

1. The key to produce C2+ products is the formation of C-C coupled intermediates, and this C-C coupling is critically dependent on the adsorption of intermediate *CO generated during the reduction. What is the adsorption mode of *CO on Zn active sites, linear or bridging?
2. The authors claimed that "HNNU-X allow water oxidation to produce hydrogen in the form of N(H+)=C=X/H+, and then CO2 is reduced with N(H+)=C=X/H+ to generate acetaldehyde". Direct evidence should be provided to confirm the photoreduction of CO2 with in-situ generated hydrogen (H+) in the form of N(H+)=C=X/H+.
3. MHOFs created the necessary proton-rich microenvironment for photocatalytic CO_2 reduction. What happens if the HNNU-X catalysts are pre-protonated under acidic conditions?
4. These HNNU-X materials could be self-healed upon simple recrystallization. Please provide the detail procedure for HNNU-X self-healing, which is very important to understand the reusability of catalyst.

Reviewer #3

(Remarks to the Author)
This work addresses an important and timely challenge in artificial photosynthesis by reporting the direct conversion of atmospheric CO2, water, and natural sunlight into a C2 product (acetaldehyde) without sacrificial agents (using only water). Please note that I am a computational chemist by training, and while I do not feel qualified to judge the quality or validity of the experimental techniques, I will focus on the overall manuscript presentation and, more specifically, provide a detailed assessment of the discussion regarding the computational results.

The conceptual advance combining direct air capture, proton-shuttling MHOFs, and C-C coupling under atmospheric conditions is significant and potentially suitable for a high-impact journal such as Nature Communications.

However, in its current form, the manuscript falls short in presentation and clarity. Figures are overcrowded, difficult to interpret, and sometimes of poor quality, which requires the reader to make an extra effort to go through. In my view, this reflects a lack of attention to detail. Typically, such oversights would lead me to recommend rejection, because I tend to think that if the authors do not carefully prepare clear figures, there is no assurance that they have rigorously verified their results either. Nevertheless, I believe that the underlying science is promising, and with substantial improvements in figure quality, data organization, and attention to the points outlined below, the work could be properly evaluated and considered for publication.

1. The systems described in references 6 and 7, for example, are reported to reduce CO2 into acetaldehyde using pure CO2. Has their performance been evaluated under atmospheric CO_2 conditions to enable a fair comparison with the MHOFs introduced in this work? To put these systems into context, it would be very helpful to include a table comparing their performance (e.g., product formation rates, selectivity, quantum yield) with previously reported CO2-to-CH3CHO (or even CO2-to-C2+) photocatalysts. This would also support the claim in line 75: "The performance is even greater than that of most reported catalysts which were performed with pure CO2 atmosphere and visible light illumination."

2. As anticipated in my overall assessment, figures are generally difficult to read, with text that is way too small. This is particularly true for Scheme 1, Figure 1, Figures 3g–i, 5, 6g–i, 7 and 8. Figure 1 contains unusual chemical structures, such as carboxylates with two protons or three oxygens, which I guess arise from crystallographic disorder. I suggest illustrating chemically meaningful structures while indicating disorder using ellipsoids, as is common practice. Since the interactions and packing of the three systems are similar, I suggest to show only one in the main text and place the others in the Supporting Information, removing the extraneous atoms mentioned above, of course.

3. Line 202: The statement "the reduced band gap facilitates more efficient charge transfer and optical excitation processes.36" is an overgeneralization. A smaller band gap does not automatically imply improved charge separation or faster charge transfer; in fact, narrower band gaps can increase recombination rates. Reference 36 does not provide direct

evidence supporting this claim for the current material. It would be more accurate to provide data demonstrating enhanced charge mobility or separation, or to soften the statement to indicate that a reduced band gap may increase light absorption without assuming improved charge transfer efficiency.

4. Figure 4: No clear band gap can be seen in the density of states. Could this be a consequence of using overly broad Gaussian smearing?

5. Figure 7a: The current free energy profile is extremely crowded, making it nearly impossible to read, with overlapping numbers, structures, and energy levels! I recommend simplifying or enlarging the figure to improve (or allow) visualization.

6. The labels in Figure 7 do not match those in Figures S32–S34.

7. Line 312: The statement "The formation of *CO from *COOH appears to be a rate-determining step, where HNNU-Se has the minimum energy barrier (ΔG = 1.45 eV)" is an abuse of language. The reported value represents a thermodynamic energy change, not a kinetic barrier, as no transition state is computed. Therefore, the energy levels in Figure 7a reflect thermodynamics, not kinetics, and the term "rate-determining step" is misleading. If the authors with to discuss kinetics, I suggest them to calculate the transition state for the C-O bond cleavage and check if it involves a free-energy barrier that can be surmountable. Note that the barrier height might depend on the redox state of the catalyst (ie. if it happens before or after a reductive quenching step of the *CO intermediate).

8. While the proposed reaction mechanism provides a reasonable bird-eye view of possible reaction intermediates that may be formed, it still leaves in a blind spot several key aspects of the mechanism, or how some reaction steps take place. For example, how do we go from *CO to *COCHO? Do we form a *CHO intermediate first (not shown in Figure 7a) which then couples with a neighboring *CO? Does the 3DI structure of the material allow such coupling by placing two metal centers close enough? Or this rather happens on a single site bearing two *CO or a *CO + *CHO? Where do electrons come from to promote these steps? Should we assume that after the formation of each intermediate, the system absorbs light to form an excited state that can be reductively quenched by water?? I strongly believe that these aspects deserve deeper discussion.

9. Figure 7b is referenced after Figures 7c/d. Figures should follow a logical order in the text.

10. In the computational details section:
- What solvent model was used (SMD, PCM, COSMO, ...)?
- Some references are missing (functional, basis set, quantum chemistry code, solvent model…).
- The experimental pH is not zero, yet the authors set pH = 0, making all ΔG_{pH} corrections zero. If the correction is not formally applied, its inclusion is misleading.
- Cartesian coordinates for all computed structures should be included in the Supporting Information or preferably uploaded to an open repository to enable reproducibility.
- Information about charge and spin multiplicity of all computed species should be stated (perhaps a table in the SI, along with electronic and Gibbs free energies?)

11. Finally, the manuscript would also benefit from a general revision of English, as there are numerous grammatical issues throughout.

Reviewer #4

(Remarks to the Author)
In this work, Pang and co-authurs developed a novel [Zn(tpy)](XCN)2-incorporated MHOF photocatalysts of HNNU-X (X= O, S, Se), which can selectively capture and concentrate atmospheric CO2 under ambient conditions, and in-situ convert the adsorbed CO2 into acetaldehyde under natural sunlight. The authors claim that HNNU-X creates a proton-rich microenvironment, where $[Zn(tpy)]^{2+}$ cations acts as CO_2 capture/activation sites for direct air capture, and $[XCN]^-$ anions as proton shuttling to rapid transfer of in-situ generated H^+ from solar water splitting to adsorbed CO_2. This artificial photosynthesis system depicts a very attractive pathway, using only sunlight, air, and water as the feedstocks, CH3CHO can be continuously generated at the catalyst surface without any other chemicals required. Overall the article is well-written, and the hypotheses are verified experimentally. It is suggested that it can be accepted after minor revision.
1. The authors described the MHOFs as "bioinspired metalo hydrogen-bonded organic frameworks". What is the resemblance between MHOFs and natural enzymes for photosynthesis?
2. $[XCN]^-$ anions are proposed to function as proton shuttles, facilitating proton transfer to the *CO intermediate in this MHOF system. Please discuss the proton transfer efficiency of different $[XCN]^-$ anions (X=O, S, Se) by the in-situ characterization.
3. How can Zn^{2+} ion participate in the photocatalytic CO_2 reduction? Authors should perform the in-situ mechanistic study to clarify the change of coordination environment and electron density of Zn^{2+} ion during the CO_2 photoreduction.
4. To understand the advancement of HNNU-X, it would be necessary to compare different catalysts used for photocatalytic CO_2 reduction to acetaldehyde.
5. Could the acetaldehyde production rate be enhanced by applying visible light without compromising selectivity?
6. Can authors analyze the oxidation product, eg. O2 gas evolution? Does it follow the stoichiometric chemistry?
7. Please check the language carefully.

Version 1:

Reviewer comments:

Reviewer #1

(Remarks to the Author)
The authors have taken my previous report in a constructive manner and have performed the requested additional measurements to address my comments. I appreciate this constructive revision very much. However, the authors have to understand that direct reduction of CO_2 from air in 400 ppms concentration in the presence of a very large excess of far more reactive O_2 using solar light forming a extremely valuable C-C bond product with a photocatalyst that do not seem very stable in water since it is based on hydrogen bridges is like the most difficult photocatalytic process that deserves all the necessary controls before it can be published. I am satisfied with most of the responses except in the key product identification.
To start with, although the caption does not indicate the solvent, from the original manuscript it is supposed that the 1H NMR spectrum of CH_3CHO shown in Figure 5 g is recorded in water. However, it is well known that acetaldehyde in H_2O is in equilibrium with the corresponding 1,1-ethanediol that is the prevalent species. Why Fig. 5 g does not show this species as the major one and shows exclusively the carbonylic form?
Second, in the 13C NMR spectrum of $^{13}CH_3{}^{13}CHO$ shown in Fig. 5 h and in more detail in the response, this diol form should also be observed, what is not the case. Furthermore, in the expansion provided in the response (it should also be shown in the main text), the C atoms appear as doublet with different coupling constants. This is not possible, since the coupling should be exactly the same for the two C atoms in the system.

Based on these inconsistencies, publication is not recommended.

Reviewer #2

(Remarks to the Author)
All the comments have been well addressed. I thus recommend the publication of this work as is.

Reviewer #3

(Remarks to the Author)
The authors have successfully addressed my comments, and I therefore recommend the publication of this article. However, I still have a few minor concerns that, in my opinion, should be refined to improve the scholarly presentation prior to publication:

Although the authors have made an effort to enhance the readability of the figures, the text in Scheme 1 remains difficult to read. For example, “high purity CO_2” in panel (a) is only visible upon significant zooming, and the label on the lamp is not legible. Similarly, the text in the cycles of panel (b) is too small, and the use of white text on a dark green background in the boxes at the bottom does not help much... The same applies to Figures 6f-g, where the assignment of bands is rather difficult to read.

Regarding the revised DOS figures, they are much improved, and the HOMO-LUMO gaps can now be easily observed due to the reduced Gaussian smearing. However, there was no need of shifting the HOMO levels to 0 eV. If done, though, it should be explicitly stated in the caption, and the x-axis label should be changed from E (eV) to deltaE (eV) to clarify that the energies are relative to the HOMO level rather than absolute values.

Finally, I strongly encourage the authors to include appropriate references in the computational details section for the methods, codes, algorithms, and approaches employed, as their developers deserve proper credit.

Reviewer #4

(Remarks to the Author)
The revised properly responds to the reviewer's comments. The reviewer does not have other comment.

Version 2:

Reviewer comments:

Reviewer #1

(Remarks to the Author)
The authors have solved my last concerns about the 13C NMR spectrum of the double 13C labeled acetaldehyde. Given the importance of forming acetaldehyde from ambinet CO_2, I would recommend, however, to include also the 1H NMR spectrum of this double labelled acetaldehyde. Apart from this addition that can be made in the proof correction stage, publication is now recommended.

Reviewer #3

(Remarks to the Author)
The authors have successfully addressed my concerns and I therefore recommend this paper for publication as is.

Open Access This Peer Review File is licensed under a Creative Commons Attribution 4.0 International License, which permits use, sharing, adaptation, distribution and reproduction in any medium or format, as long as you give appropriate credit to the original author(s) and the source, provide a link to the Creative Commons license, and indicate if changes were made.
In cases where reviewers are anonymous, credit should be given to 'Anonymous Referee' and the source.
The images or other third party material in this Peer Review File are included in the article's Creative Commons license, unless indicated otherwise in a credit line to the material. If material is not included in the article's Creative Commons license and your intended use is not permitted by statutory regulation or exceeds the permitted use, you will need to obtain permission directly from the copyright holder.
To view a copy of this license, visit https://creativecommons.org/licenses/by/4.0/

Response to the reviewers' comments

Reviewers #1:

1. The Reviewers **#1**: Acetaldehyde is far more reactive than CO_2 and its formation on a photocatalytic process, particularly in the presence of O_2, should always be taken cautiously and supported on firm grounds. This is not the present case. Figs. S14-17 corresponding to ^{1}H NMR spectra should have recorded with a water suppression mode. In any case, the spectrum of the ^{13}C labelled CH_3CHO should have been presented.

We gratefully appreciate for your valuable suggestions. Indeed, acetaldehyde is more reactive than CO_2. Therefore, we conducted a rigorous and systematic analysis of the liquid products from CO_2 photoreduction. The liquid product was first determined by gas chromatograph-mass spectrometer (GC-MS). The dominant GC peak at 3.03 min is assigned to CH_3CHO (Fig. R1a), and the corresponding mass spectrum exhibits a molecular ion at m/z = 44, consistent with its molecular weight (Fig. R1b). These results indicate that **HNNU-X** preferentially promotes the formation of CH_3CHO from CO_2 under ambient air and natural sunlight . Minor amounts C_2H_5OH (2.04 min) and CH_3OH (1.92 min) were also detected. The GC-MS spectra of liquid products were provided in revised manuscript (Figs. 5c and d in page 14).

To further confirm the formation of CH_3CHO, we recorded ^{1}H NMR spectrum of the reaction solution with a water suppression mode (Fig. R1c). Distinct proton signals at 1.22 and 9.57 ppm are observed, corresponding to the C*H*$_3$ and C*H*O protons of CH_3CHO, respectively. To verify that acetaldehyde originates from CO_2 photoreduction, isotopic labeling experiments were performed using $^{13}CO_2$ instead of air over **HNNU-Se**. The resulting ^{13}C NMR spectrum (Fig. R1d) shows characteristic isotope-induced splitting at ~19.4 and 98.9 ppm, attributable to the ^{13}*C*H$_3$ and ^{13}*C*HO carbons in $^{13}CH_3{}^{13}CHO$, respectively. It confirms CO_2 as the carbon source.

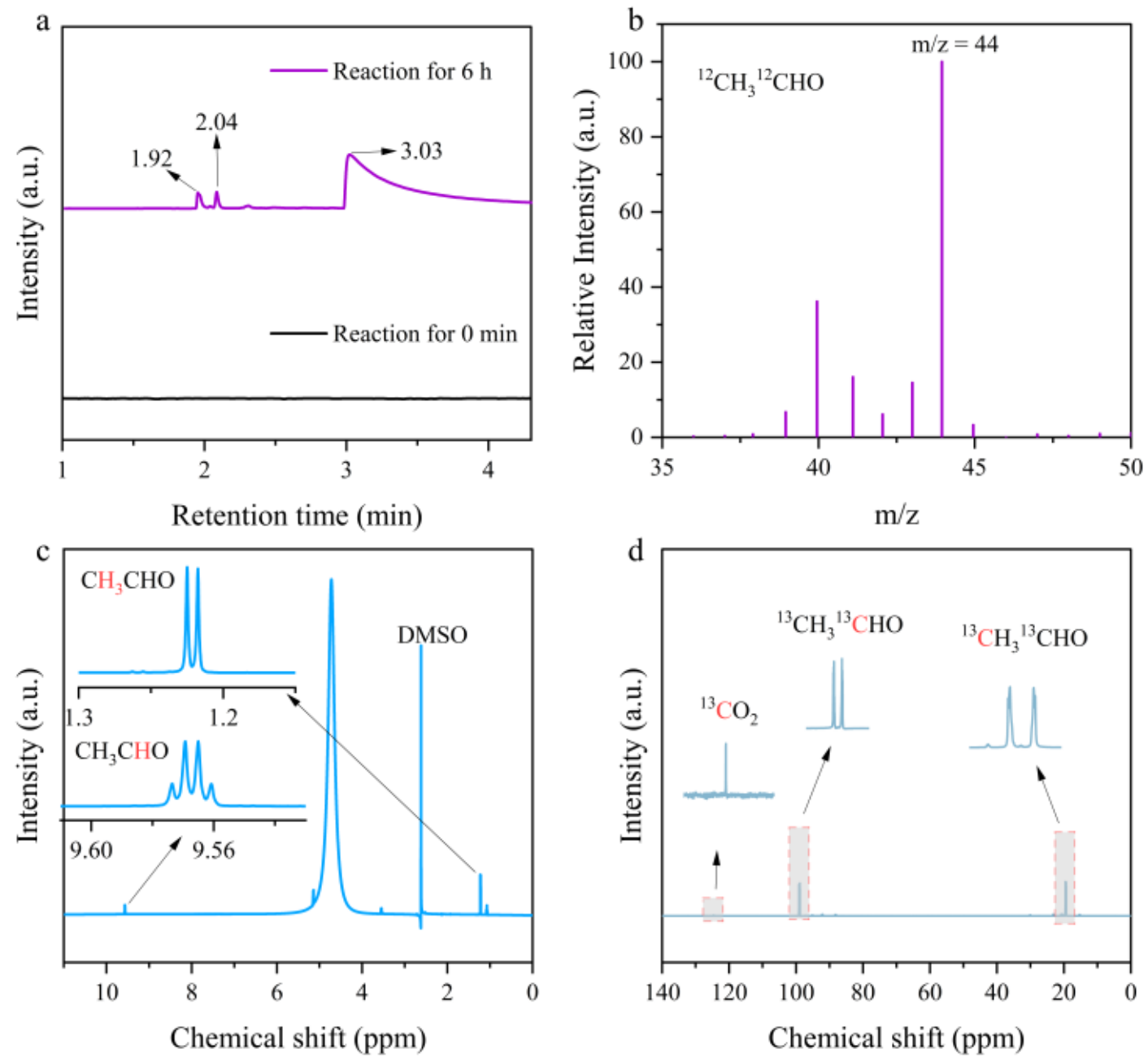

Fig. R1. GC-MS (a and b) and ^{1}H NMR (c) spectra of liquid product of photocatalytic CO_2 reduction with H_2O in air over **HNNU-Se**, ^{13}C NMR spectrum (d) of ^{13}C labelled CH_3CHO from

$^{13}CO_2$ photoreduction.

All results confirm that **HNNU-X** enables the photocatalytic conversion of CO_2 to CH_3CHO. Actually, previous study has reported a photocatalytic system that was capable of oxidizing ethanol to acetaldehyde during CO_2 photoreduction, yet unable to further oxidize acetaldehyde, although acetaldehyde is far more reactive than both CO_2 and ethanol [Simultaneous co-photocatalytic CO_2 reduction and ethanol oxidation towards synergistic acetaldehyde synthesis, *Angew. Chem. Int. Ed.*, 2023, 62, e202218720]. The observed persistence of acetaldehyde is probably due to the extremely low solubility of O_2 in water (*ca.* 28.3 mg/L), which limits its availability at the reaction interface and thereby suppresses further oxidation under aqueous photocatalytic conditions.

According to the expert's good suggestions, we have provided all ^1H NMR spectra of liquid product with a water suppression mode in revised Supporting Information.(supplementary Figs. 17-19 in page 17), as well as the ^1H and ^{13}C NMR of $^{13}CO_2$-labeled product in revised manuscript (Figs. 5g and h in page 14).

2. The Reviewers **#1**: Mass spectra of labelled products are useful only after chromatographic separation. The corresponding chromatograms should be presented in Fig. 5. Also, what is the peak in the MS at m/z 45 in Fig. 5d? Even more important the mass spectrum of shown in Fig. 5 e should be wrong. How is possible if the compound is CD_3CDO have mass fragments of 1 amu if this molecule does not contain any element of amu 1? In addition, how acetaldehyde has been extracted from the aqueous phase should be indicated.

Thank you for your valuable suggestion. Isotope-labeling experiments were performed to confirm the photoreduction of CO_2 to CH_3CHO over **HNNU-X**. We have provided the chromatograms of corresponding Ms of labelled products (Fig. R2).

When $^{12}CO_2$ was used as carbon source, the dominant signal appears at m/z = 44, consistent with the molecular weight of CH_3CHO (Fig. 1b). By replacing $^{12}CO_2$ with $^{13}CO_2$, this peak shifts to m/z = 46, corresponding to $^{13}CH_3{}^{13}CHO$ (Figs. R2a and 2b). It verifies that the carbon atoms in CH_3CHO originate from CO_2. A signal at m/z = 45 is observed, which can be attributed to the participation of pre-adsorbed $^{12}CO_2$ on the catalyst surface—consistent with the strong CO_2 affinity of **HNNU-X**—leading to partially labeled products ($^{13}CH_3{}^{12}CHO$ or $^{12}CH_3{}^{13}CHO$). When D_2O was employed, the peak at m/z= 44 shifts to 48 (Figs. R2c and 2d), corresponding to CD_3CDO. The appearance of +1 amu fragments in the MS (Fig. R2d) is likely due to residual, pre-adsorbed H_2O from ambient air, due to the high water affinity of **HNNU-X**. Similar isotope-labeling behavior has been reported previously [Visible light-driven acetaldehyde production from CO_2 and H_2O via synergistic vacancies and atomically dispersed Cu sites, *Angew. Chem. Int. Ed.*, 2025, 64, e202422667].

To further exclude interference from pre-adsorbed $^{12}CO_2$ and H_2O, **HNNU-Se** was thoroughly pretreated by drying at 80 °C under vacuum for 24 h, followed by activation at 120 °C under vacuum for an additional 24 h prior to testing. Under these conditions, the m/z = 45 signal is markedly suppressed in the mass spectra of ^{13}C-labeled acetaldehyde (Figs. R2a and R2b). Moreover, the +1 amu fragments are nearly eliminated in experiments using $^{12}CO_2$ and D_2O (Figs. R2c and R2d).

Given the extremely low concentrations and full water solubility of the liquid products, analyses

were conducted without extraction, following established protocols [Regulating the *OCCHO intermediate pathway towards highly selective photocatalytic CO_2 reduction to CH_3CHO over locally crystallized carbon nitride, *Energy Environ. Sci.*, 2022, 15, 225–233; Visible light-driven acetaldehyde production from CO_2 and H_2O via synergistic vacancies and atomically dispersed Cu sites, *Angew. Chem. Int. Ed.*, 2025, 64, e202422667]. Briefly, after reaction, the catalyst was removed by centrifugation, and 1 μL of supernatant was directly injected (10 μL syringe) into a gas chromatograph equipped with a flame ionization detector (FID) for GC-MS analysis. After each measurement, the column was aged at 200 °C to maintain chromatographic performance and ensure long-term stability.

Furthermore, the liquid products were quantified by NMR spectroscopy, in which dimethyl sulfoxide (DMSO, Macklin, 99.99%) was used as the internal standard [Photothermal CO_2 conversion to ethanol through photothermal heterojunctionnanosheet arrays, *Nat. Comm.*, 2024, 15, 5639]. Aliquots at an interval of 1 h were drawn from the reaction mixture and analyzed by ^{1}H NMR. For ^{1}H NMR measurement, 0.5 mL of reaction solution was mixed with 0.1 mL of D_2O, and 10 μL of DMSO (equivalent to 140.8 μmol) was added as the internal standard. The area of product peaks was compared to the area of DMSO (at a chemical shift of 2.6 ppm). In our case, the double peak of acetaldehyde at a chemical shift of 1.22 ppm was used to calculate the generation rate (N) as the following equation:

$$N = \frac{S \times V2 \times 140.8 \times 6}{2 \times V1 \times t \times m}$$

where S is the area of double peak of acetaldehyde compared to DMSO (identified to 1 as reference) (0.007 for **HNNU-Se**, 0.003 for **HNNU-S**, 0.002 for **HNNU-O**), V2 is the volume of water (2.5 mL) used during photocatalysis, V1 is the volume of reaction solution tested (500 μL), t is reaction time (6 h) and m is the mass of catalyst (0.005 g).

According to the expert's good suggestions, we have provided the GC-MS of labelled products in revised supporting information (Supplementary Fig. 20 in page 17), as well as the quantization method in revised manuscript (lines 483-492 in pages 25-26).

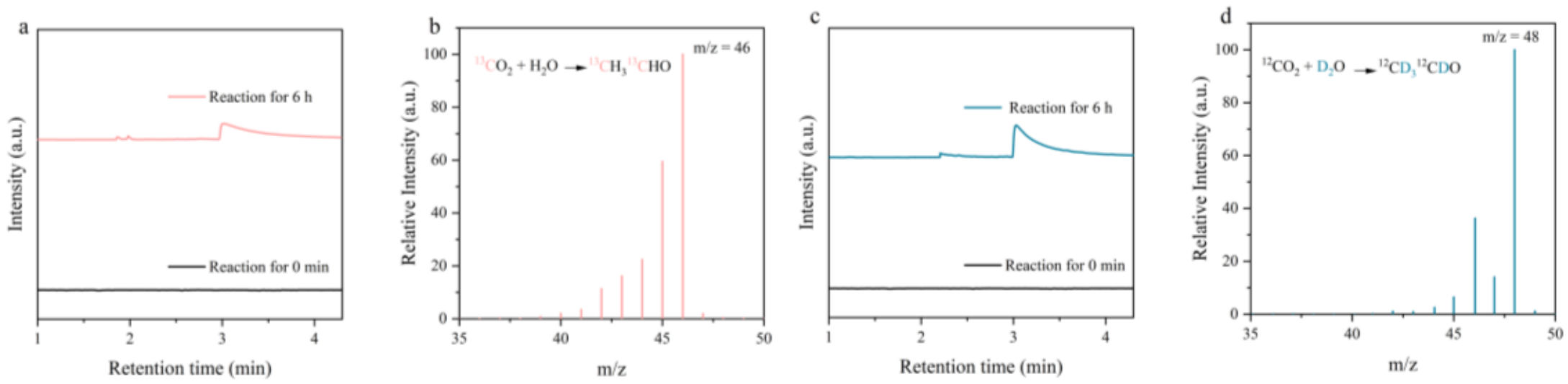

Fig. R2. GC (a) and corresponding MS spectrum (b) of produced acetaldehyde from $^{13}CO_2$ coupled with H_2O over **HNNU-Se**. GC (c) and corresponding MS spectrum (d) of produced acetaldehyde from $^{12}CO_2$ coupled with D_2O over **HNNU-Se.**

3. The Reviewers **#1**: All the discussion of the preferent CO_2 adsorption over N_2 or O_2 adsorption has no sense. To start the comparison should be made at the relative concentration of these compounds in air, in which CO_2 concentration is very low and moisture is present. In addition to be in considerable excess respect to CO_2, O_2 reduction is much more favorable than reduction of CO_2.

Thanks for the expert's good suggestions. Although the concentration of CO_2 in air (~0.04%) is

much lower than that of O_2 (~20%), the present photoreduction occurs in the aqueous phase, where the solubility of CO_2 (*ca.* 430 mg/L) is much higher than that of O_2 (*ca.* 8.3 mg/L). In addition, the hydrophilic **HNNU-X** frameworks show a strong affinity for CO_2, arising from the multiple nitrogen atoms in the terpyridyl ligands that serve as Lewis basic sites for CO_2 capture. The combination of low O_2 solubility and selective CO_2 adsorption by **HNNU-X** should effectively suppress competitive O_2 reduction, despite its more favorable thermodynamics.

Recent advances have demonstrated that integrating CO_2 capture and conversion within a single photocatalytic system enables direct utilization of atmospheric CO_2. For instance, the group of Chunhui Dai reported metal-salen-incorporated conjugated microporous polymers that efficiently capture CO_2 and H_2O, functioning as artificial leaves for solar-driven CO_2 reduction under ambient conditions [Metal–Salen-Incorporated conjugated microporous polymers as robust artificial leaves for solar-driven reduction of atmospheric CO_2 with H_2O, *Carbon Energy,* 2025, 7, e646.]. Similarly, Zhimin Liu and co-workers developed an ionic liquid-polymer hybrid system in which a task-specific ionic liquid captures CO_2 and H_2O from air, while a conjugated polymer photocatalyst converts the adsorbed CO_2 into CO [Visible-light-driven conversion of CO_2 from air to CO using an ionic liquid and a conjugated polymer, *Green Chem.*, 2017, 19, 5777-5781]. Inspired by these pioneering studies, we sought to integrate CO_2 capture and reduction within a hydrophilic **HNNU-X** catalyst for aqueous-phase photoreduction of atmospheric CO_2.

To further evaluate the competitive reduction of CO_2 and O_2 over **HNNU-Se**, linear sweep voltammetry (LSV) measurements were conducted using an **HNNU-Se**-modified electrode in CO_2- or O_2-saturated Na_2SO_4 solution (1 M). As shown in Fig. R3, the oneset of cathodic current occurs at -1.33 and -1.47 V vs Ag/AgCl, KCl_{sat} for CO_2- or O_2-saturated electrolyte solution, respectively. It suggests that CO_2 reduction is easier to be triggered than O_2 reduction in the aqueous photocatalytic system over **HNNU-Se**. Moreover, only a slight increase in current density is observed in O_2-saturated solution at potentials more negative than -1.47 V, suggesting that O_2 reduction rarely occurs in the aqueous photocatalytic system. In contrast, the current density in CO_2-saturated solution increases markedly with more negative potentials. These observations demonstrate that CO_2 reduction predominates over O_2 reduction in the aqueous photocatalytic system, in agreement with the properties of **HNNU-X**.

According to the expert's good suggestions, we have discussing the competitive reduction of O_2 with CO_2 in the aqueous photocatalytic system in revised supporting information (Supplementary Fig. 25 in page 22).

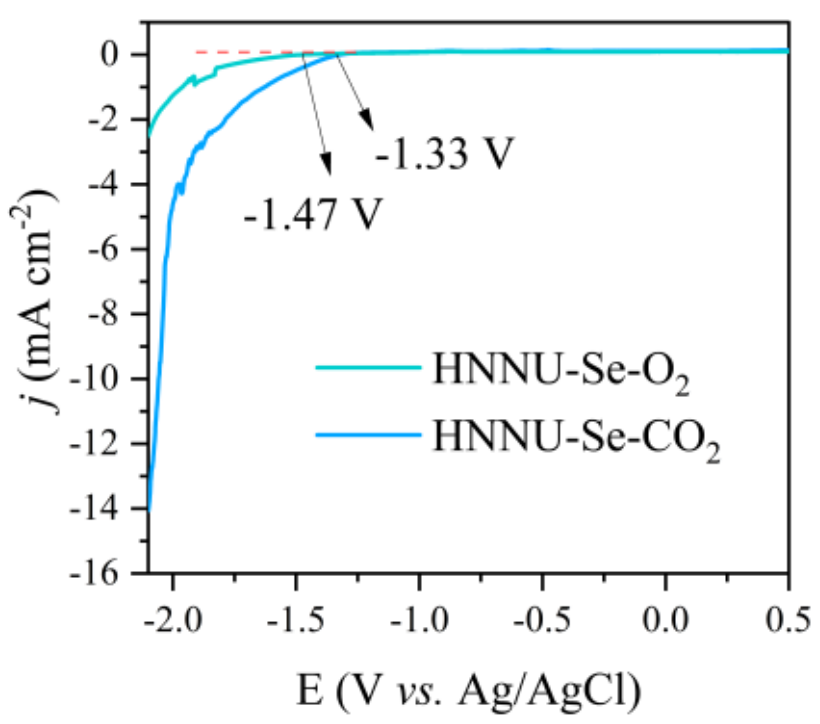

Fig. R3. LSV curves at **HNNU-Se**-modified electrodes (The tests were conducted in a CO_2- or O_2-saturated Na_2SO_4 aqueous solution (1 M) under visible light irradiation).

4. The Reviewers **#1**: The authors should provide the calculation made to determine a CO_2 reduction rate to acetaldehyde of 557.1 μmol.g^{-1}.h^{-1}. It is unclear if air flow is passed through the photocatalyst suspended in water of if this number is achieved under static conditions, just by contact with air. In my calculations, converting all the CO_2 present in one litter of air at atmospheric pressure will render only 12.5 μmol of acetaldehyde. Because the authors are using 5 mg, this means that at full conversion the maximum rate can be 2500 μmol.g^{-1}.h^{-1}. So to achieve the number reported in the paper, conversion should be over 20 %, something that is doubtful particularly considering the photoreactor used. The Reviewers **#1**: The photocatalytic reactor shown in Figure S13 is poorly designed and should be very inefficient. Considering that sunlight comes from the top, the fact that it is opaque means that direct light rays cannot not access. What is considered the surface area of this reactor? Also, the system is open to the air or there is an air flow through the liquid phase?

Thanks for your good suggestions. The liquid products were quantified by nuclear magnetic resonance (NMR) (Bruker AVANCE AV III 700) spectroscopy, in which dimethyl sulfoxide (DMSO, Macklin, 99.99%) was used as the internal standard. The specific operation methods are as follows: We first took 500 μL solution from the bottom of the reactor after the catalysis and mixed it with 100 μL deuterated water (D_2O). Then 10 μL dimethyl sulfoxide (DMSO) (equivalent to 140.8 μmol) was added as internal standards for the ^{1}H NMR analysis. The area of product peaks was compared to the area of DMSO (at a chemical shift of 2.6 ppm). In our case, the double peak of acetaldehyde at a chemical shift of 1.22 ppm was used to calculate the generation rate (N) as the following equation:

$$\mathrm{N} = \frac{S \times V2 \times 140.8 \times 6}{2 \times V1 \times t \times m} = \frac{S\ (0.007) \times V2\ (2.5\ \mathrm{mL}) \times 140.8 \times 6}{2 \times V1\ (500\ \mu\mathrm{L}) \times t\ (6\ \mathrm{h}) \times m\ (0.005\ \mathrm{g})} = 557.1\ \mu\mathrm{mol/g/h}$$

where S is the area of double peak of acetaldehyde compared to DMSO (identified to 1 as reference) (0.007 for **HNNU-Se**, 0.003 for **HNNU-S**, 0.002 for **HNNU-O**), V2 is the volume of water (2.5 mL) used during photocatalysis, V1 is the volume of the reaction solution tested (500 μL), t is reaction time (6 h) and m is the mass of catalyst (0.005 g).

This acetaldehyde production rate was obtained at atmospheric air under stirring, as shown the Fig. R4a. Actually, two types of photoreactors were employed to evaluate the photocatalytic CO_2 reduction performance.

The photoreactor shown in Figure S13 is a self-designed quartz vessel (25 mL) sealed with a high-transparency quartz lid, ensuring efficient light penetration from a xenon lamp (Figs. R4b and R4c). A double-layer structure was designed to enables the circulation of cooling water, thereby maintaining the reaction at ambient temperature. This photoreactor equipped with a xenon lamp as light source, was used as a controlled laboratory platform for photocatalytic studies under pure CO_2 atmosphere.

For outdoor experiments under natural sunlight, a cylindrical quartz reactor with a spherical bottom was specifically designed to maximize light harvesting (Fig. R4a). An airbag (100 L), connected *via* a three-way valve, was used to supply atmospheric air into the reactor, a constant airflow was achieved by applying a ~5.0 kg load on the airbag (Fig. R4a). The photocatalytic reaction was conducted under continuous stirring in ambient air. During operation, the inclination angle of photoreactor was adjusted in real time to track the sunlight trajectory, thereby maximizing the illuminated surface area and ensuring efficient utilization of natural sunlight.

We have provided the detail experiment in revised manuscript (lines 478-501 in pages 25-26)

and provided the photoreactor in revised supporting information (Supplementary Fig. 16 in page 15).

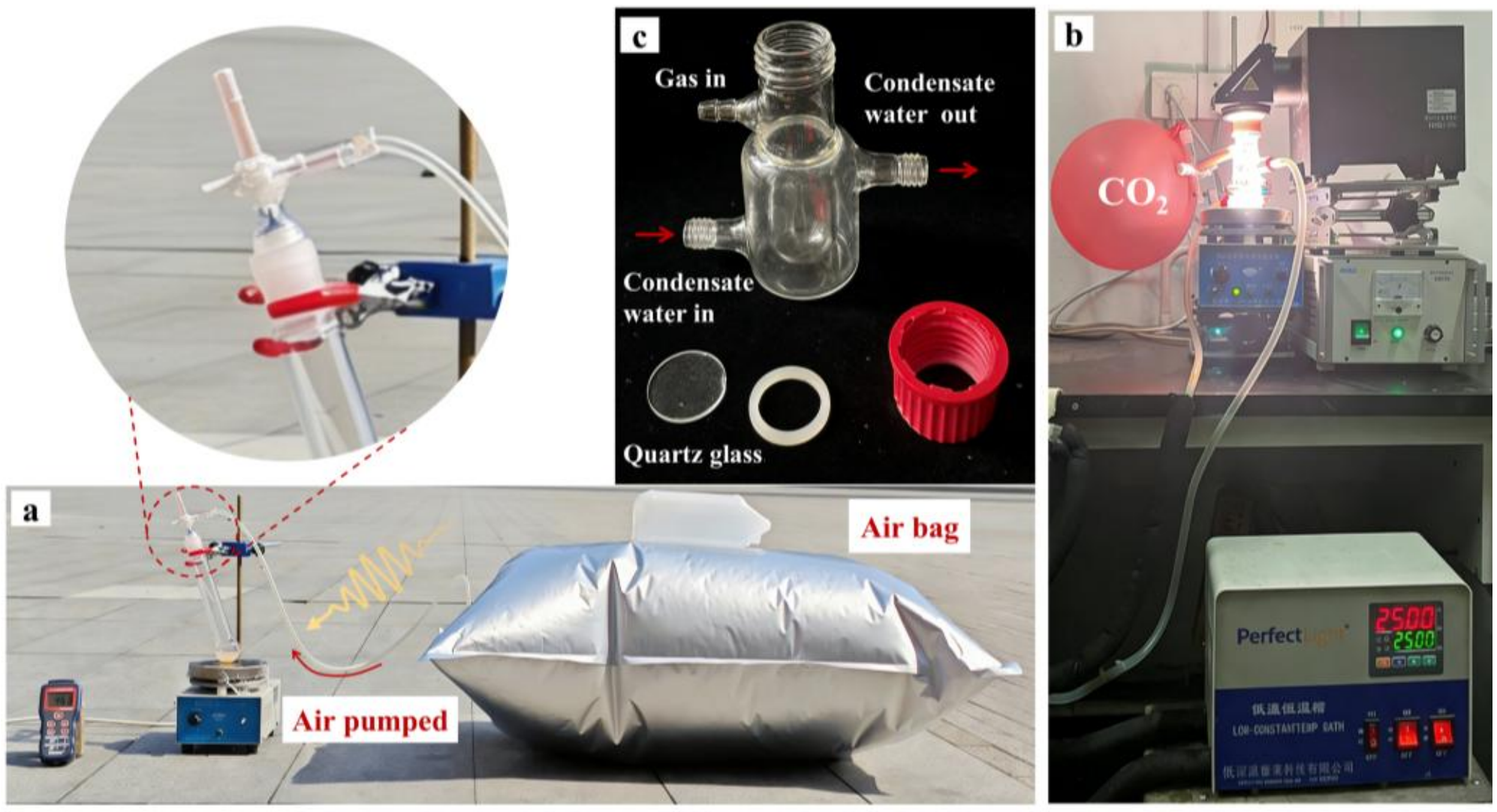

Fig. R4. Demonstration of CO_2 reduction under natural sunlight in air atmosphere (a). Demonstration of CO_2 reduction under xenon lamp in CO_2 atmosphere (b) and the structure of laboratorial photoreactor (c).

5. The Reviewers **#1**: According to the band potential, **HNNU-Se** should not be able to oxidize H_2O to ·OH. The authors propose an explanation about the CO_2 reduction assistance to overcome the thermodynamics, but these arguments are not convincing and unsupported. Experimental section should indicate how these irradiations were made. If O_2 is present, how do the authors rule out the obvious O_2 reduction to superoxide resulting in H_2O_2? The manuscript does not indicate in many parts how the experiments were carried out, confusing the reader in many sections.

Thanks for the expert's good suggestions. To evaluate the capability of **HNNU-Se** to reduce O_2 to superoxide (•O_2^-), in-situ electron paramagnetic resonance (EPR) spectroscopy was employed using 5,5-dimethyl-1-pyrroline N-oxide (DMPO) as the spin-trapping agent. The measurements were conducted in acetonitrile under a pure O_2 atmosphere with triethanolamine (TEOA) as a hole scavenger. Specifically, **HNNU-Se** (5 mg) was dispersed in acetonitrile (2 mL), followed by the addition of TEOA (0.5 mL). The suspension was bubbled with O_2 for 1 h prior to transfer into an O_2-filled quartz tube. A 300 W xenon lamp was used for irradiation during the in situ measurements. As shown in Fig. R5a, negligible signals corresponding to •O_2^- were detected under illumination, ruling out superoxide formation over **HNNU-Se**. Instead, a distinct EPR signal attributable to the terpyridine-based radical anion (tpy•-) was observed [Morphology control of supramolecular assembly for superior CO_2 photoreduction, *ACS Catal.*, 2023, 13, 2086-2093], indicating photoinduced electron transfer from XCN^- to $[Zn(tpy)]^{2+}$ center upon photoexcitation.

To probe the generation of hydroxyl radicals (•OH) from H_2O oxidation, in-situ EPR experiments were further conducted in aqueous solution under a pure CO_2 atmosphere without any sacrificial agents. **HNNU-Se** (5 mg) was dispersed in water (2.5 mL), purged with CO_2 for 1 h, and subsequently transferred into a CO_2-filled quartz tube. Under irradiation with a 300 W xenon lamp, characteristic DMPO-•OH signals emerged and intensified with irradiation time (pear-shaped mark) (Fig. R5b), confirming the formation of •OH radicals via water oxidation

[Near-infrared-activated multiphoton photocatalytic system for synergistic therapy via CO generation and •OH production, *Angew. Chem. Int. Ed.*, 2026, e24756]. Notably, a sextet signal corresponding to $\bullet CO_2^-$ radicals (square mark) was also observed (Fig. R5b), indicating that CO_2 is reduced through capture of photogenerated electrons [Regulating the *OCCHO intermediate pathway towards highly selective photocatalytic CO_2 reduction to CH_3CHO over locally crystallized carbon nitride, *Energy Environ. Sci.*, 2022, 15, 225–233]. These results demonstrate that **HNNU-Se** simultaneously drives CO_2 reduction to CH_3CHO and water oxidation to •OH radicals. Consequently, the formation of H_2O_2 originates from water oxidation rather than O_2 reduction during the photocatalytic process.

According to your good suggestions, we have described experimental details of in-situ EPR spectroscopy in revised supporting information (lines 106-112 in pages 4-5), and provided the results and discussions in revised manuscript (Figs. 7e and f, lines 382-395 in page 21).

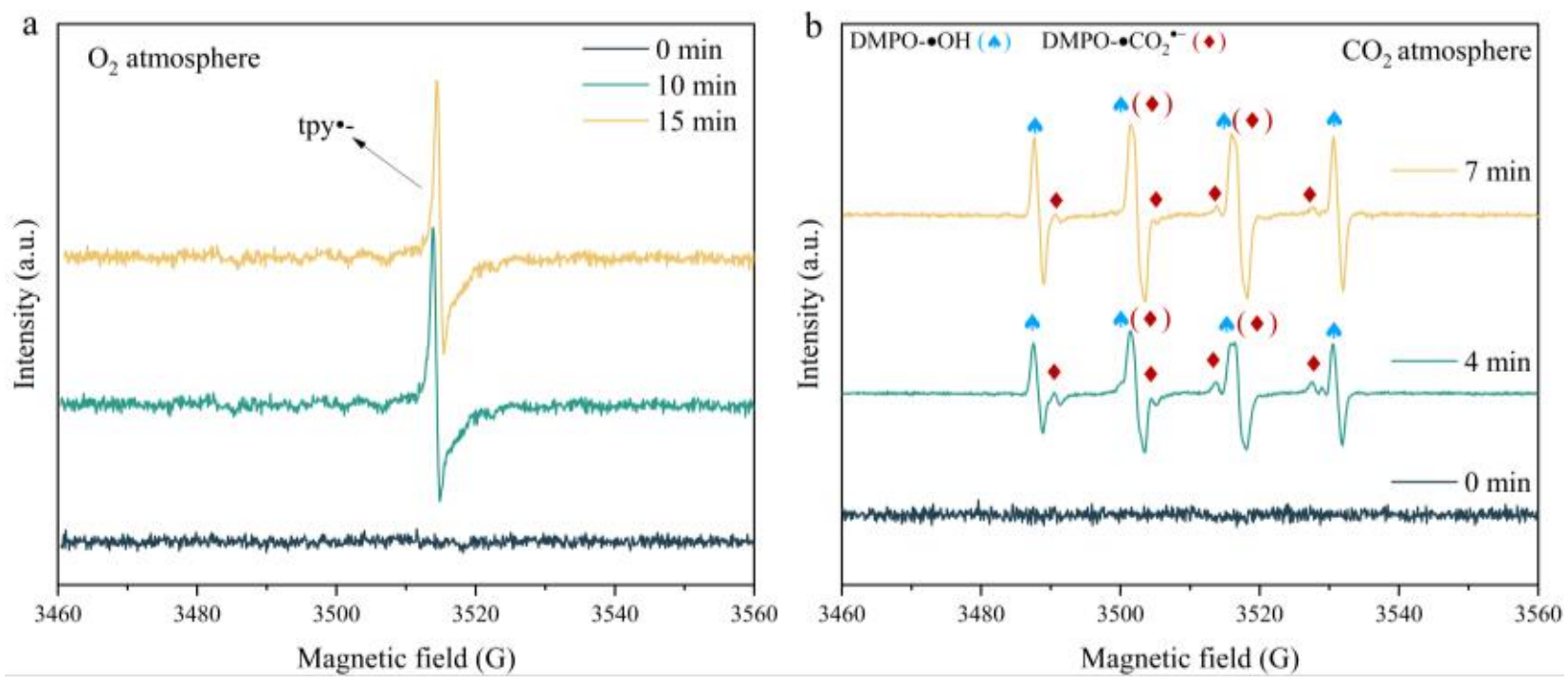

Fig. R5. *In-situ* EPR spectra over **HNNU-Se** in acetonitrile under O_2 atmosphere with TEOA as hole scavenger (a), *in-situ* EPR spectra over **HNNU-Se** in water under CO_2 atmosphere without any scavengers (b) (DMPO was added as the radical trapping agent).

6. The Reviewers **#1**: There is a contradiction between calculations suggesting an electron transfer from the electron rich $SeCN^-$ anion to the terpyridyl linker with formation of SeCN radical as indicated by Figure 4 and then in the XPS discussion to identify Zn^{2+} as the site receiving the electron and later in line 282 they indicate that the terpyridyl ligand gives one electron to Zn.

Thanks for the expert's good suggestions. According to density functional theory (DFT) calculations, the lowest unoccupied molecular orbitals (LUMOs) are predominantly localized on the $[Zn(tpy)]^{2+}$ centers, whereas the highest occupied molecular orbitals (HOMOs) reside mainly on the coordinated XCN^- anions. This orbital distribution suggests that, upon photoexcitation, electrons are transferred from the XCN^- anions (HOMOs) to the $[Zn(tpy)]^{2+}$ centers (LUMOs), consistent with previous reports [Unprecedented photocatalytic hydrogen peroxide production via covalent triazine frameworks constructed from fused building blocks, *Angew. Chem. Int. Ed.*, 2025, 64, e202416350].

To experimentally verify this photoinduced electron-transfer pathway, in-situ X-ray photoelectron spectroscopy (XPS) measurements were conducted under vacuum in both dark and illuminated conditions. As shown in Figs. R6a-6f, light irradiation induces a negative shift in the binding energy of Zn^{2+} species, accompanied by positive shifts in the binding energies of the X elements (O, S, or Se) in the corresponding XCN^- anions. These results indicate electron transfer from XCN^- to $[Zn(tpy)]^{2+}$ centers, in good agreement with the DFT-predicted frontier orbital distribution.

To further elucidate the electron-transfer process under working conditions, in-situ X-ray absorption fine structure (XAFS) of **HNNU-Se** was performed before and after irradiation with CO_2 atmosphere (Figs. R7a and b). As shown in Fig. R7c, the Zn K-edge X-ray absorption near-edge structure (XANES) exhibits a pre-edge feature at ~9661.3 eV, characteristic of Zn(II) species [In situ identification of zinc sites as potential-dependent selectivity switch over dual-atom catalysts for H_2O_2 electrosynthesis, *J. Am. Chem. Soc.*, 2025, 147, 11465−11476]. Upon illumination, the absorption edge shifts to lower energy with a concomitant decrease in white-line intensity, indicating an increase in electron density around Zn^{2+} centers. In contrast, the Se K-edge XANES shows a positive shift in the absorption edge and an increase in white-line intensity under light irradiation (Fig. R7d), reflecting a decrease in electron density at the $SeCN^-$ moieties. These operando XAS results provide direct evidence for photoinduced electron transfer from $SeCN^-$ anions to $[Zn(tpy)]^{2+}$ center in **HNNU-Se** under light irradiation.

According to the theoretical and experimental results, we have revised the expression into "an efficient electron transfer pathway from the XCN^- anions to $[Zn(tpy)]^{2+}$ centers upon photoexcitation" in revised manuscript (lines 196-197 in page 11). Furthermore, we have also provided the operando XAFS tests of **HNNU-Se** in revised manuscript (Figs. 6d and e in page 16).

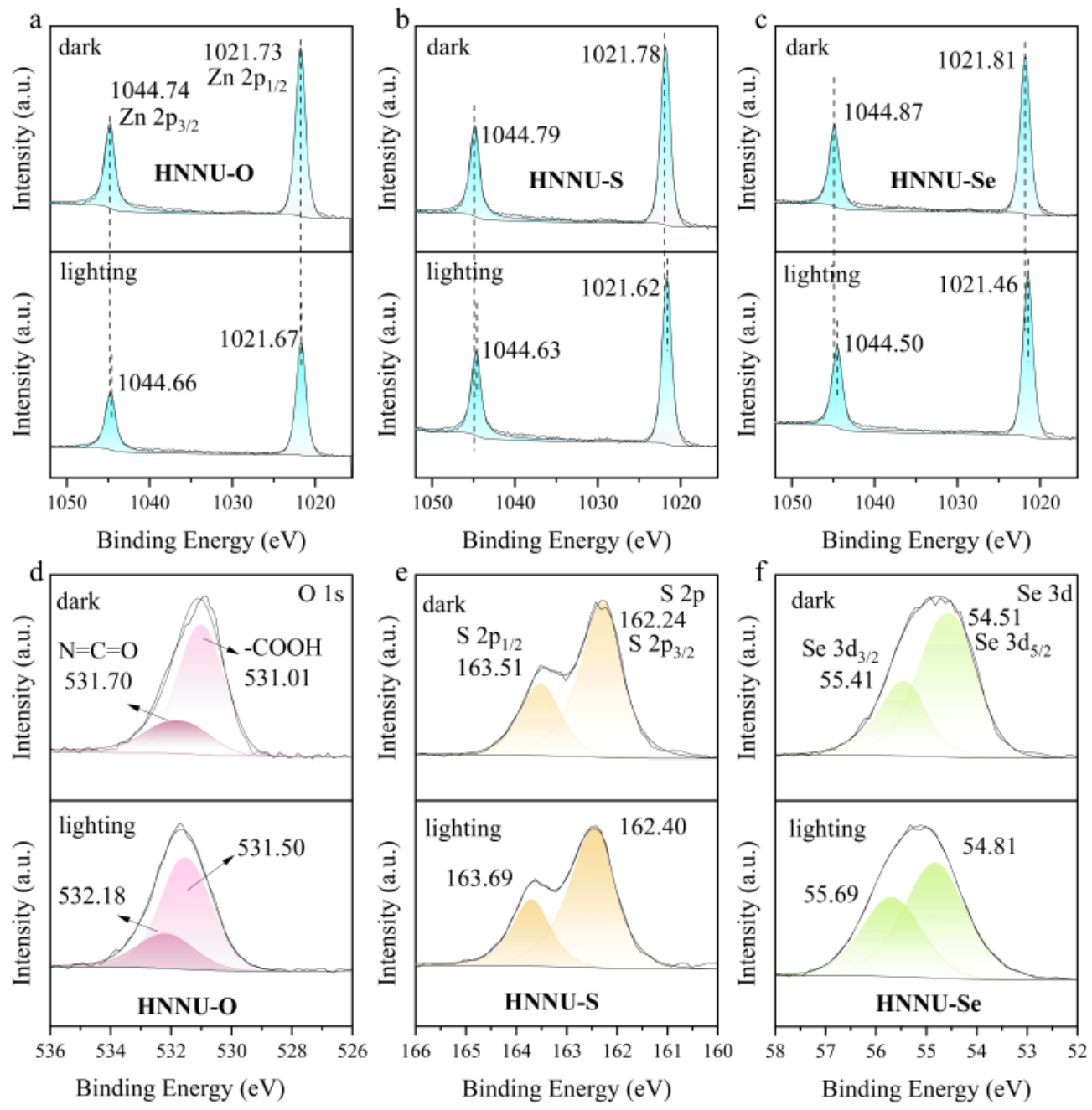

Fig. R6. *In-situ* XPS spectra of Zn 2p (a) and O 1s (d) over **HNNU-O**, Zn 2p (b) and S 2p (e) over **HNNU-S**, and Zn 2p (c) and Se 3d (f) over **HNNU-Se** before and after irradiation.

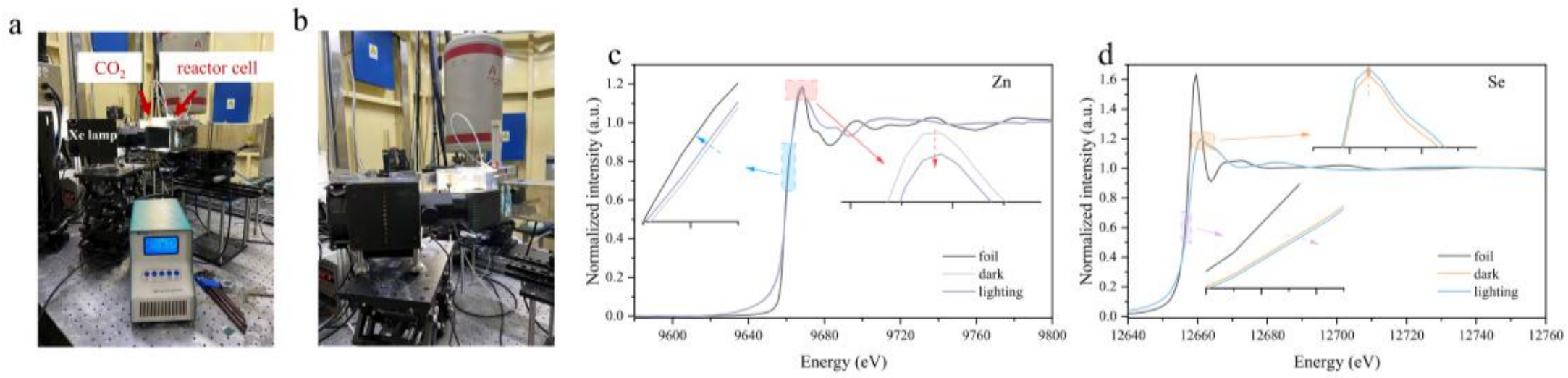

Fig. R7. Photograph of the operando XAFS measurement setup (a and b). Operando Zn (c) and Se (d) K-edge XANES spectra of **HNNU-Se** in water with CO_2 atmosphere before and after light

irradiation.

7. The Reviewers **#1**: Furthermore, some of the discussion is based on XPS Zn core peak shifts of 0.06 eV or 0.15 eV. In my experience this is within the experimental error. In any case, the authors should indicate the error in their measurements? Furthermore, the XPS of S and Se are extremely noisy and suggesting that these ligands have been lost, probably replaced by oxygenated groups. Atomic proportion of Se and S should be given.

Thanks for the expert's good suggestions. In conventional XPS measurements, binding energy (BE) shifts typically arise from changes in the chemical environment, and small variations (e.g., 0.06 or 0.15 eV) may fall within experimental uncertainty. In contrast, in-situ XPS probes the same sample under dynamically varying conditions, where BE shifts originate from changes in the reaction environment rather than sample-to-sample differences. As such, even subtle BE variations directly reflect real-time changes in the electronic states of the catalyst. Similar observations have been widely reported in prior in situ XPS studies [Modulating charge accumulation via electron interaction for photocatalytic hydrogen evolution: a case of fabricating palladium sites on $ZnIn_2S_4$ nanosheets, *ACS Catal.,* 2023, 13, 11687-11696; Zinc mediated electronic structure of CoP toward photocatalytic H_2 evolution, *Appl. Catal. B Environ. Energy*, 2025, 367, 125098; Reversed electron transfer in Cu-TiO_2@$ZnIn_2S_4$ for boosting photocatalytic CO_2 reduction, *Adv. Funct. Mater.*, 2025, 36, e19072].

According to the reviewer's suggestion, we re-acquired in-situ XPS spectra for Zn 2p and O 1s in **HNNU-O**, Zn 2p and S 2p over **HNNU-S**, and Zn 2p and Se 3d over **HNNU-Se**, both before and after irradiation. As shown in Figs. R6a-6c, all **HNNU-X** samples exhibit two characteristic peaks in high-resolution Zn 2p spectra under dark conditions, corresponding to Zn $2p_{3/2}$ (~1044 eV) and Zn $2p_{1/2}$ (~1021 eV), consistent with Zn^{2+}. Upon illumination, both Zn 2p components shift to lower binding energy: the Zn $2p_{3/2}$ peak shifts from 1044.74 to 1044.66 eV for **HNNU-O**, from 1044.79 to 1044.63 eV for **HNNU-S**, and from 1044.87 to 1044.50 eV for **HNNU-Se**. The corresponding decreases (0.08, 0.16, and 0.37 eV, respectively) are comparable to those observed in our previous measurements. These systematic shifts indicate an increase in electron density at Zn^{2+} centers under irradiation, rather than experimental uncertainty.

Because the in-situ XPS measurements were performed under vacuum, the loss of XCN^- species during analysis can be excluded. The previously observed spectral noise is likely attributable to suboptimal acquisition parameters or environmental fluctuations. In the re-measured spectra, the S 2p and Se 3d regions display well-resolved features. Notably, upon light irradiation, both S and Se signals shift to higher binding energy (Figs. R6e and 6f), indicating a decrease in electron density on the XCN^- anions. Together, these results provide compelling evidence for photoinduced electron transfer from XCN^- anions to $[Zn(tpy)]^{2+}$ centers in **HNNU-X**, in agreement with the DFT-predicted frontier orbital distribution.

Given that XPS provides only semi-quantitative surface composition analysis, inductively coupled plasma optical emission spectroscopy (ICP–OES) was further employed to determine the bulk elemental composition. The Se and S contents in **HNNU-Se** and **HNNU-S** were measured to be 22.23 wt% and 10.63 wt%, respectively, in good agreement with the corresponding theoretical values (26.15 wt% for **HNNU-Se** and 12.54 wt% for **HNNU-S**).

According to your good suggestions, we have provided the re-conducted *in-situ* XPS spectra in the revised supporting information (Supplementary Fig. 14 in page 13) and the contents of X in

HNNU-X in revised manuscript (lines 270-272 in page 15).

8. The Reviewers **#1**: Related with the previous point, reusability studies should provide analytical data of the Se content upon reuse, both in the liquid and the solid phase.

Thanks for the expert's good suggestions. After the reaction, **HNNU-X** materials were recovered from the reaction mixture by centrifugation. ICP-OES analysis shows that Se content in the recovered **HNNU-Se** (22.18 wt%) is almost identical to that of the fresh sample (22.23 wt%). Moreover, negligible Se was detected in the supernatant, confirming that no measurable leaching of Se species occurred during the aqueous photocatalytic CO_2 reduction.

According to your good suggestions, we have provided the analytical data of Se content upon reuse, both in the liquid and solid phase, in revised manuscript (lines 270-272 in page 15).

9. The Reviewers **#1**: Experimental section lacks necessary details. The authors should indicate how the ESI and Mott-Schottky measurements were performed and the electrolyte. Are the complexes stable under the electrochemical measurement conditions? The authors should analyze the electrolyte for the absence of S/Se derived from ligand leaching.

Thanks for the expert's good suggestion. Photoelectrochemical measurements were carried out on an electrochemical workstation (CHI650E, Shanghai) using a standard three-electrode configuration, with a platinum plate and an Ag/AgCl electrode serving as the counter and reference electrodes, respectively. The working electrode was prepared by depositing the catalyst onto a 1×1 cm^2 indium tin oxide (ITO) glass substrate via a conventional slurry method. Specifically, 2.5 mg of sample was dispersed in a mixed solvent containing 700 μL H_2O, 200 μL acetonitrile, and 100 μL of 5 wt% Nafion, followed by ultrasonication for 4 h to form a homogeneous suspension. A mixed aqueous solution of $K_4Fe(CN)_6$ (0.5 mM) and Na_2SO_4 (0.5 M) was used as the electrolyte. A 300 W Xe lamp (Perfectlight) served as the light source. Mott-Schottky measurements were conducted in dark using a 5 mV AC perturbation at frequencies of 500, 750, and 1000 Hz. Electrochemical impedance spectroscopy (EIS) was recorded over a frequency range of 0.05 Hz to 100 Hz with an amplitude of 5 mV at the same applied bias. Linear sweep voltammetry (LSV) was performed in 0.5 M Na_2SO_4 aqueous solution at a scan rate of 5 mV s^{-1} using Ag/AgCl and Pt electrodes as the reference and counter electrodes, respectively. ICP-OES analysis of the post-measurement electrolyte revealed negligible Se content, confirming that no detectable leaching occurred. These results demonstrate that **HNNU-X** materials remain stable under the electrochemical testing conditions.

We have provided the detailed experiments, including electrochemical measurements, fs-TA, in-situ DRIFTS, in-situ EPR, in-situ XPS, and Operando XAFS, in revised Supporting Information (Section 1.2 in pages 3-5).

Reviewers #2:

10. The Reviewers **#2**: The key to produce C_2^+ products is the formation of C-C coupled intermediates, and this C-C coupling is critically dependent on the adsorption of intermediate *CO generated during the reduction. What is the adsorption mode of *CO on Zn active sites, linear or bridging?

Thanks for the expert's good suggestion. In-situ diffuse reflectance infrared Fourier transform spectroscopy (DRIFTS) was employed to probe the *CO adsorption behavior on Zn active sites.

The CO-DRIFTS spectra were recorded using an FTIR spectrometer (Bruker INVENIO-S, Model: Spectrum 100) equipped with a modified Harrick in situ cell (Model HV-DR2), at a spectral resolution of 4 cm^{-1}. Prior to CO exposure, the system was purged with N_2 (30 $mL.min^{-1}$) to remove residual gases. CO was then introduced at 25 °C to enable adsorption, followed by collection of background spectra.

As shown in Fig. R8, bands in the range of 2150-2200 cm^{-1} are assigned to gas-phase CO and disappear upon purging, confirming their non-adsorbed nature. The bands at 2000-2049 cm^{-1} are attributed to linearly adsorbed CO species. Specifically, the feature at 2033 cm^{-1} corresponds to CO linearly coordinated to Zn active sites, whereas the band at 2013 cm^{-1} is assigned to linearly adsorbed CO molecules which form hydrogen bonding with protonated $[XCN]^-$ anions proximal to Zn^{2+} centers, as illustrated in the insets of Fig. R8. Notably, no bridge-bonded CO species are observed for **HNNU-X**.

The presence of adjacent linearly adsorbed CO species on Zn sites is expected to facilitate C–C coupling, thereby promoting the formation of C_2 intermediates enroute to CH_3CHO.

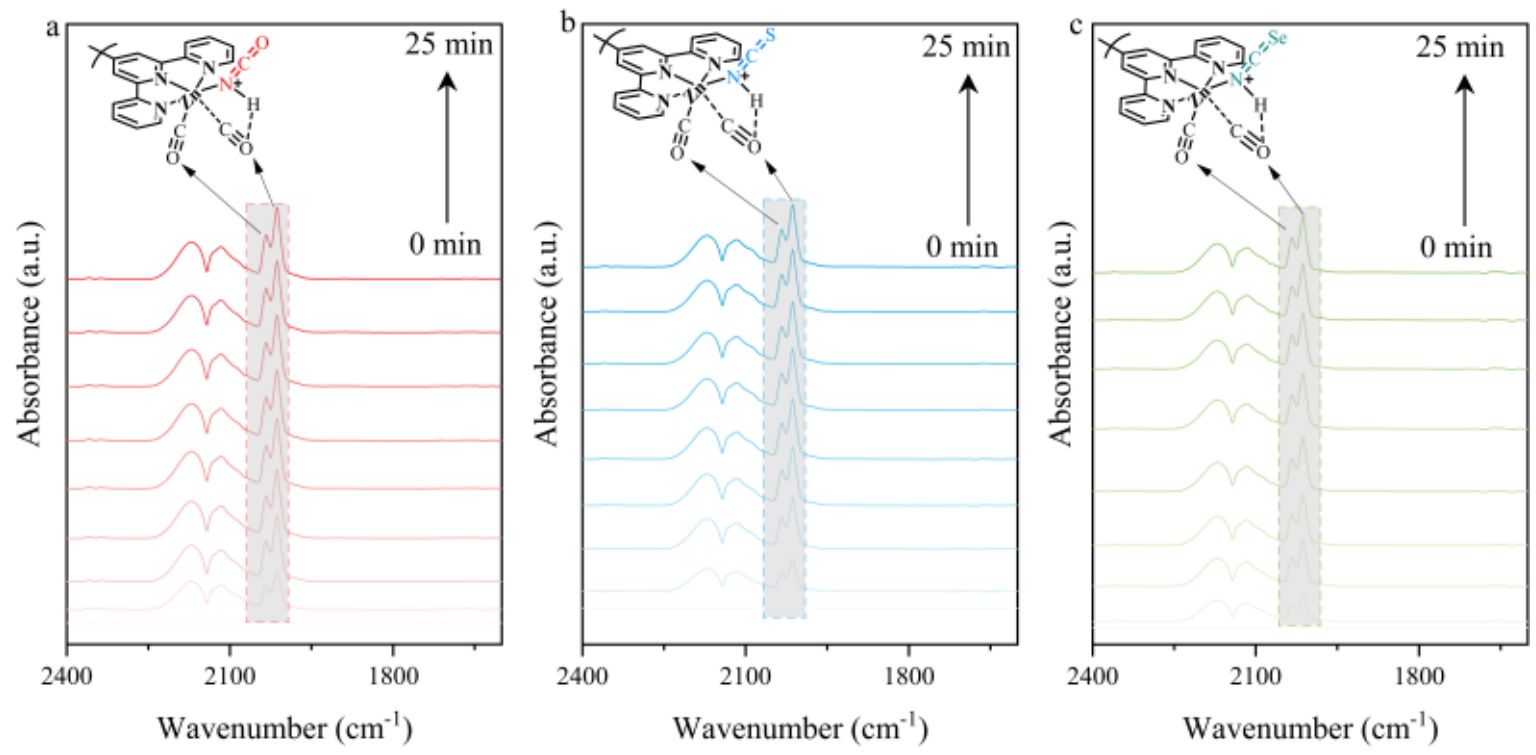

Fig. R8. *In-situ* CO adsorption DRIFTS spectra under dark condition on **HNNU-O** (a), **HNNU-S** (b) and **HNNU-Se** (c).

11. The Reviewers **#2**: The authors claimed that "HNNU-X allow water oxidation to produce hydrogen in the form of $N(H^+)=C=X/H^+$, and then CO_2 is reduced with $N(H^+)=C=X/H^+$ to generate acetaldehyde". Direct evidence should be provided to confirm the photoreduction of CO_2 with in-situ generated hydrogen (H^+) in the form of $N(H^+)=C=X/H^+$. The Reviewers **#4**: $[XCN]^-$ anions are proposed to function as proton shuttles, facilitating proton transfer to the *CO intermediate in this MHOF system. Please discuss the proton transfer efficiency of different $[XCN]^-$ anions (X=O, S, Se) by the in-situ characterization.

Thanks for the expert's good suggestion. Operando X-ray absorption fine structure (XAFS) was employed to elucidate proton transfer from in-situ generated $N(H^+)=C=X$ species to adsorbed CO_2 during photocatalysis over **HNNU-Se** (Fig. R9). In the Se K-edge extended X-ray absorption fine structure (EXAFS) spectra, three major peaks are observed at phase-uncorrected radial distances (r) of ~1.39, 1.93, and 2.46 Å, corresponding to the first-shell Se–C, second-shell Se–C–N, and third-shell Se–C–N–Zn scattering paths, respectively. Upon illumination in CO_2 for 40 min, the first-shell Se–C peak gradually weakens and broadens, which can be attributed to protonation of $[SeCN]^-$ by photogenerated H^+ to form $N(H^+)=C=Se$ species. With prolonged irradiation (60 min), the Se–C peak intensity partially recovers, indicating deprotonation of $N(H^+)=C=Se$ due to proton transfer to adsorbed CO_2. These operando XAS results provide direct evidence that CO_2

photoreduction proceeds via participation of in-situ generated protons in the form of $N(H^+)=C=Se/H^+$.

To further probe the protonation state of **HNNU-X** (X = O, S, Se), X-ray photoelectron spectroscopy (XPS) measurements were conducted (Fig. R10). Prior to analysis, the samples were pre-protonated in dilute hydrochloric acid. The N 1s spectra can be deconvoluted into three components at ~398 eV, ~399 eV, and ~405 eV, assigned to N=C=X, pyridinic N, and protonated $N(H^+)=C=X$ species, respectively. According to deconvolution of the peak areas, $N(H^+)=C=X/N=C=X$ ratios are calculated to be 0.76, 0.29, and 0.16 and for **HNNU-Se** (Fig. R10a), **HNNU-S** (Fig. R10b), and **HNNU-O** (Fig. R10c), respectively. These results indicate a progressively decreased protonation degree of $[XCN]^-$ anions in the order **HNNU-Se** > **HNNU-S** > **HNNU-O**.

We have provided the Operando Se K-edge EXAFS in revised manuscript (Figs. 7h and i in page 20) and the N 1s XPS spectra of pre-protonated **HNNU-X** in revised Supporting Information (Supplementary Fig. 32 in page 26).

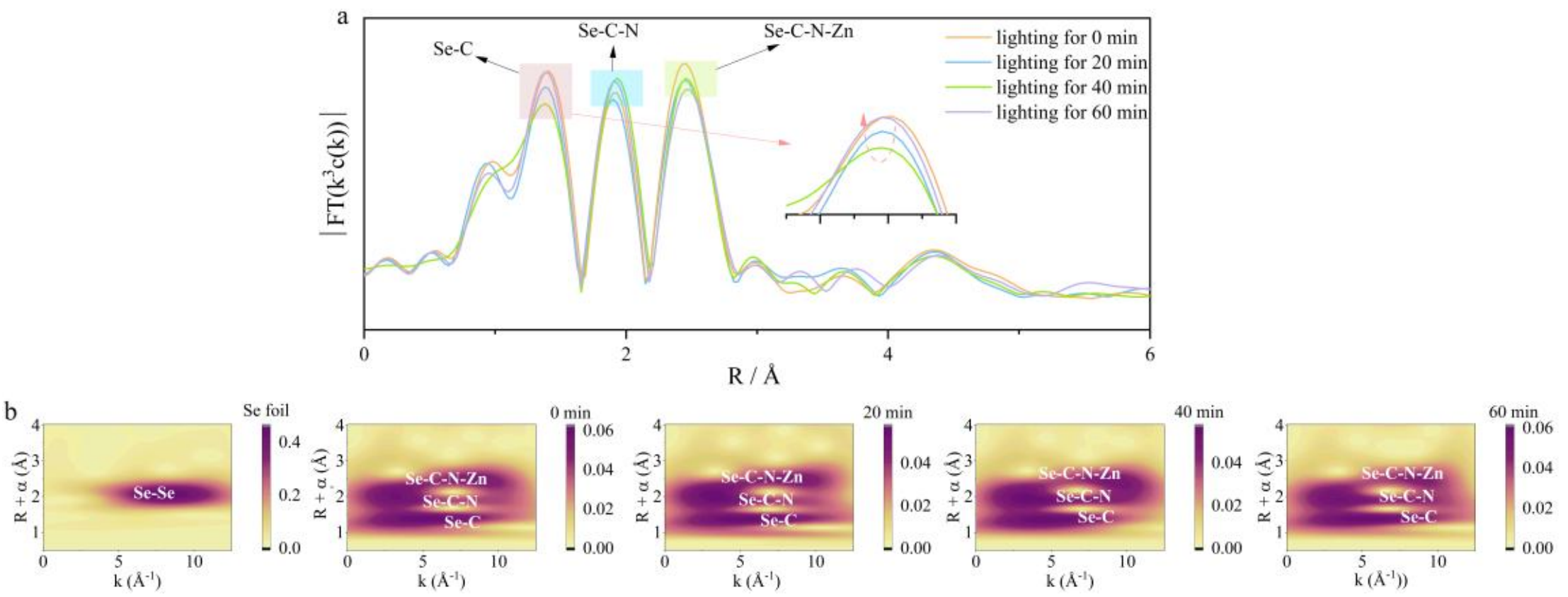

Fig. R9. FT of k^3-weighted *Operando* Se K-edge EXAFS upon light irradiation for different time (a). WTs for the k^3-weighted Se K-edge EXAFS signals under different reaction conditions (b).

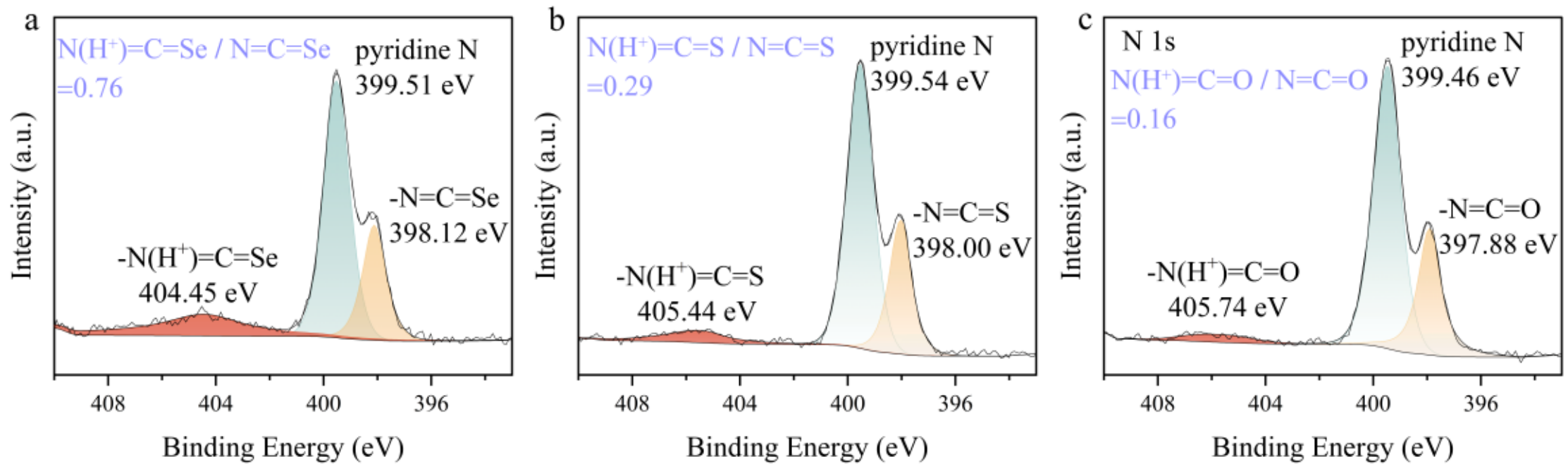

Fig. R10. N 1s XPS spectra of pre-protonated **HNNU-Se** (a), **HNNU-S** (b) and **HNNU-O** (c).

12. The Reviewers **#2**: MHOFs created the necessary proton-rich microenvironment for photocatalytic CO_2 reduction. What happens if the HNNU-X catalysts are pre-protonated under acidic conditions?

According to the expert's good suggestion, **HNNU-Se** was pre-protonated by immersion in 1 M HCl overnight and subsequently evaluated for photocatalytic CO_2 reduction in water. Notably, the pre-protonated **HNNU-Se** exhibits a slightly higher activity (572.2 μmol.g^{-1}.h^{-1}) to that of the

pristine sample (557.1 μmol.g^{-1}.h^{-1}) (Table R1). This enhancement likely arises from the intrinsically high water affinity of **HNNU-Se**, which ensures abundant proton availability at the reaction interface.

Table R1. Photoreduction atmospheric CO_2 over **HNNU-Se** and pre-protonated **HNNU-Se** in water under natural sunlight

Catalysts	Atmosphere	Sacrificial agent	CH_3CHO production rate (μmol^{-1}.g^{-1}.h^{-1})	Electron selectivity (%)
**HNNU-Se**	air	/	557.1	95.1
pre-protonated **HNNU-Se**	air	/	572.2	94.8

Reaction conditions: catalyst (5 mg), deionized water (2.5 mL), air atmosphere, natural sunlight, reaction temperature (25 °C).

13. The Reviewers **#2**: These **HNNU-X** materials could be self-healed upon simple recrystallization. Please provide the detail procedure for HNNU-X self-healing, which is very important to understand the reusability of catalyst.

Thanks for the expert's good suggestion. After the reaction, all **HNNU-X** (X = O, S, Se) samples were readily recovered from the reaction system by centrifugation. A slight decrease in crystallinity was observed, likely due to the participation of water in the hydrogen-bonding network. The recovered materials were subsequently regenerated via recrystallization in *N,N*-dimethylformamide (DMF). Specifically, the recovered **HNNU-X** (50 mg) was dissolved in DMF (20 mL) to obtain a clear solution, and the supernatant was transferred to a 50 mL glass vial. The vial was then placed in a beaker containing acetone, sealed, and subjected to vapor diffusion at room temperature. After standing for 36 h, crystalline **HNNU-X** was obtained. This efficient structural restoration highlights the self-healing capability of **HNNU-X**, which can be attributed to reversible rearrangement of the hydrogen-bonded organic framework (HOF), enabled by the dynamic nature of hydrogen-bonding interactions [Self-healing B ← N-based hydrogen-bonded organic framework for exclusive recognition and separation of toluene from methyl-cyclohexane, *J. Am. Chem. Soc.*, 2024, 146, 19425-19433; Self-healing hydrogen-bonded organic frameworks for low concentration ammonia capture, *J. Am. Chem. Soc.*, 2024, 146, 627-634].

According to your good suggestions, we have provided the detail procedure for **HNNU-X** self-healing in revised Supporting Information (lines 415-419 in page 23).

Reviewers #3

14. The Reviewers **#3**: The systems described in references 6 and 7, for example, are reported to reduce CO_2 into acetaldehyde using pure CO_2. Has their performance been evaluated under atmospheric CO_2 conditions to enable a fair comparison with the MHOFs introduced in this work? To put these systems into context, it would be very helpful to include a table comparing their performance (e.g., product formation rates, selectivity, quantum yield) with previously reported CO_2-to-CH_3CHO (or even CO_2-to-C_2^+) photocatalysts. This would also support the claim in line 75: "The performance is even greater than that of most reported catalysts which were performed with pure CO_2 atmosphere and visible light illumination." The Reviewers #4: To understand the advancement of **HNNU-X**, it would be necessary to compare different catalysts used for

photocatalytic CO_2 reduction to acetaldehyde.

Thanks for the referees' good suggestion. To date, few photocatalytic systems have been reported for the direct conversion of CO_2 to acetaldehyde. Notably, the systems described in references 6 and 7 were not evaluated under atmospheric CO_2 conditions. To enable a meaningful comparison, we assessed the performance of optimized **HNNU-Se** under the same conditions reported in these studies (pure CO_2, visible-light irradiation, and absence of sacrificial agents). The results are summarized in Table R2.

The locally crystallized carbon nitride reported in reference 6 produces negligible CH_3CHO under pure CO_2 and visible-light irradiation ($\lambda \geq 420$ nm) without sacrificial agents (Table R2, entry 3), although high activity is achieved upon addition of triethanolamine (TEOA) as a hole scavenger (Table R2, entry 4) [Regulating the *OCCHO intermediate pathway towards highly selective photocatalytic CO_2 reduction to CH_3CHO over locally crystallized carbon nitride, *Energy Environ. Sci.*, 2022, 15, 225–233]. In contrast, the Cu/CZS-Vs photocatalyst reported in reference 7 delivers an acetaldehyde production rate of 121.5 μmol.g^{-1}.h^{-1} with ~80% selectivity and an apparent quantum efficiency of ~0.53% at 400 nm under similar conditions without sacrificial agents (Table R2, entry 5) [Visible light-driven acetaldehyde production from CO_2 and H_2O via synergistic vacancies and atomically dispersed Cu sites, *Angew. Chem. Int. Ed.*, 2025, 64, e202422667]. Under identical conditions, **HNNU-Se** exhibits substantially superior performance (Table R2, entry 5 *vs.* 2), achieving a CH_3CHO production rate of 721.2 μmol.g^{-1}.h^{-1}, which is approximately 5.9 times higher than that of Cu/CZS-Vs. Moreover, **HNNU-Se** also outperforms the state-of-the-art photocatalysts reported for CO_2 reduction to other C_2 products in the absence of sacrificial agents, as summarized in Table R2.

According to your good suggestions, we have provided the comparative profile in revised manuscript (Fig. 5k in page 14), and detail comparative results were provided in revised Supporting Information (Supplementary Table 4 in pages 18-19).

Table R2. Photocatalytic CO_2 reduction performances with C_2 products in previous reported photocatalysts in the literature, compared with that of **HNNU-Se**

Entry	Catalyst	Rate (μmol.g^{-1}.h^{-1})	Sel. (%)	AQE (%)	Sacrificial agent	Main product	Gas	Light source
1	**HNNU-Se** [a]	557.1	95.1	2.9 (450 nm)	/	CH_3CHO	air	Natural sunlight
2	**HNNU-Se** [b]	721.2	93.0	N.A.	/	CH_3CHO	CO_2	300 W Xe lamp
3	HCN-A [c]	No detected	/	N.A.	/	CH_3CHO	CO_2	white LED
4	HCN-A [c]	1814.7	98.3	22.4 (385 nm)	TEOA	CH_3CHO	CO_2	white LED
5	Cu/CZS-Vs [d]	121.5	80	0.53 (400 nm)	/	CH_3CHO	CO_2	300 W Xe lamp
6	CN-KRb [e]	1212.3	93.3	7.5 (420 nm)	EtOH	CH_3CHO	CO_2	white LED

7	C-SnS_2 [f]	139	N.A.	0.72 (420 nm)	/	CH_3CHO	CO_2	300W Hg lamp
8	$AgInP_2S_6$ [g]	44.3	73	0.15 (420 nm)	/	C_2H_4	CO_2	300 W Xe lamp
9	CuO_X@p-ZnO [h]	3.88	32.9	N.A.	TEA	C_2H_4	CO_2	300 W Xe lamp
10	d-UiO 66/MoS_2 [i]	39.0	94	N.A.	/	CH_3COOH	CO_2	300 W Xe lamp
11	Co_3O_4 [j]	2.95	92.5	N.A.	/	CH_3COOH	0.03% CO_2	300 W Xe lamp
12	BMO/RP [k]	51.8	<50	N.A.	/	CH_3CH_2OH	CO_2	450 W Xe-lamp
13	NiCo-TiO_2 [l]	22.3	71	N.A.	Na_2SO_3	CH_3COOH	CO_2	300 W Xe lamp
14	InCu/PCN [m]	28.5	92	N.A.	/	CH_3CH_2OH	CO_2	300 W Xe lamp
15	P/Cu SAs@CN [n]	616.6	33.0	12.5 (350 nm)	TEOA	C_2H_6	CO_2	300 W Xe lamp
16	Au_1/RP [o]	1.32	96	N.A.	/	C_2H_6	CO_2	300 W Xe lamp
17	CuACs/PCN [p]	30.5	53.2	N.A.	TEOA	C_2H_4	CO_2	300 W Xe lamp

[a] **HNNU-Se** (5 mg), deionized water (2.5 mL), air atmosphere, natural sunlight, reaction temperature (25 ºC) (This work).

[b] **HNNU-Se** (5 mg), deionized water (2.5 mL), CO_2 atmosphere, 300 W Xe lamp (λ≥400 nm), reaction temperature (25 ºC) (This work).

[c] HCN-A (15 mg), 3 mL 10% triethanolamine (TEOA)/acetonitrile (MeCN) solution with 0.3 mL H_2O, CO_2 atmosphere, 5 W white LED lamp, reaction temperature (10 ºC) [Regulating the *OCCHO intermediate pathway towards highly selective photocatalytic CO_2 reduction to CH_3CHO over locally crystallized carbon nitride, *Energy Environ. Sci.*, 2022, 15, 225–233].

[d] Cu/CZS-Vs (15 mg), 10 mL mixture of acetonitrile and water (CH_3CN: H_2O = 9: 1), CO_2 atmosphere, 300 W Xe lamp (λ≥400 nm; light intensity: 200 mW/cm^2) [Visible light-driven acetaldehyde production from CO_2 and H_2O via synergistic vacancies and atomically dispersed Cu sites, *Angew. Chem. Int. Ed.*, 2025, 64, e202422667].

[e] CN-KRb (10 mg), 5 mL 10% ethanol (EtOH)/acetonitrile (MeCN) solution with 0.5 mL H_2O, CO_2 atmosphere, white LED lamp (100 mW/cm^2), 10 ºC [Simultaneous co-photocatalytic CO_2 reduction and ethanol oxidation towards synergistic acetaldehyde synthesis, *Angew. Chem. Int. Ed.*, 2023, 62, e202218720].

[f] C-SnS_2 (0.1 g), water vapour, CO_2 atmosphere, 300 W Halogen lamp, reaction temperature (25 ºC). [Carbon-doped SnS_2 nanostructure as a high efficiency solar fuel catalyst under visible light, *Nat. Commun.*, 2018, 9, 169].

[g] $AgInP_2S_6$ (5 mg), deionized water (0.4 mL), CO_2 atmosphere, 300 W Xe lamp. [Vacancy-defect modulated

pathway of photoreduction of CO_2 on single atomically thin $AgInP_2S_6$ sheets into olefiant gas, *Nat. Commun.*, 2021, 12, 4747].

[h] CuO_X@p-ZnO (5 mg), water-vapor-saturated CO_2 gas (pressure reached 0.5 MPa), 300 W Xe lamp (320–780 nm, 100 mW/cm^{-2}). [Photocatalytic C–C coupling from carbon dioxide reduction on copper oxide with mixed-valence copper(I)/copper(II), *J. Am. Chem. Soc.*, 2021, 143, 2984-2993].

[i] d-UiO 66/MoS_2 (2 mg), water-vapor-saturated CO_2 gas (pressure reached 0.1 MPa), 300 W Xe lamp ((λ > 400 nm, 100 mW/cm^{-2}). [Hierarchically porous metal–organic framework/MoS_2 interface for selective photocatalytic conversion of CO_2 with H_2O into CH_3COOH, *Angew. Chem. Int. Ed.*, 2021, 133, 25053-25057].

[j] Co_3O_4 (2.5 mg), deionized water (5 mL), simulated air (0.03% CO_2, 79.98% N_2 and 19.99% O_2), 300 W Xe lamp (AM 1.5G filter, 100 mW/cm^{-2}), reaction temperature (25 °C). [Selective CO_2 photoreduction into C_2 product enabled by charge-polarized metal pair sites, *Nano Lett.*, 2021, 21, 2324-2331].

[k] BMO/RP (5 mg), deionized water (20 mL), CO_2 atmosphere, 450 W Xe lamp (with UV cut-off filter (KG-2 filter and CGA-400) filter), reaction temperature (25 °C). [Green transformation of CO_2 to ethanol using water and sunlight by the combined effect of naturally abundant red phosphorus and Bi_2MoO_6, *Energy Environ. Sci.*, 2022, 15, 1967-1976].

[l] NiCo-TiO_2 (0.02 g), deionized water (50 mL with 0.1 M Na_2SO_3 and 0.2 M CsOH), CO_2 atmosphere, 300 W Xe lamp. [Asymmetric coupled dual-atom sites for selective photoreuction of carbon dioxide to acetic acid, *Adv. Func. Mater.*, 2022, 32, 2206817].

[m] InCu/PCN (50 mg), DMF/H_2O (24 mL, 12.5 vol% H_2O), CO_2 atmosphere (0.8 MPa), 300 W Xe lamp (1 W/cm^{-2}). [Atomically dispersed indium-copper dual-metal active sites promoting C−C coupling for CO_2 photoreduction to ethanol, *Angew. Chem. Int. Ed.*, 2022, 61, e202208904].

[n] P/Cu SAs@CN (25 ml, 20 mg/L water suspension), TEOA (3.0 mL), CO_2 atmosphere (0.65 atm) 300 W Xe lamp. [P and Cu dual sites on graphitic carbon nitride for photocatalytic CO_2 reduction to hydrocarbon fuels with high C_2H_6 evolution, *Angew. Chem. Int. Ed.*, 2022, 61, e202210789].

[o] Au_1/RP (0.05 g), 0.5 g of $KHCO_3$ and 6 M HCl to produce high-purity CO_2 and water vapor atmosphere, 300 W Xe lamp (300 mW·cm^{-2}). [Atomically dispersed Au-assisted C–C coupling on red phosphorus for CO_2 photoreduction to C_2H_6, *J. Am. Chem. Soc.*, 2022, 144, 22075-22082].

[p] CuACs/PCN (5 mg), deionized water (45 mL), TEOA (5 mL), photosensitizer ($C_{30}H_{24}Cl_2N_6Ru\cdot6H_2O$), CO_2 atmosphere, 300 W Xe lamp. [P-mediated Cu-N_4 sites in carbon nitride realizing CO_2 photoreduction to C_2H_4 with selectivity modulation, *Adv. Mater.*, 2023, 35, 2208132].

15. The Reviewers #**3**: As anticipated in my overall assessment, figures are generally difficult to read, with text that is way too small. This is particularly true for Scheme 1, Figure 1, Figures 3g–i, 5, 6g–i, 7 and 8. Figure 1 contains unusual chemical structures, such as carboxylates with two protons or three oxygens, which I guess arise from crystallographic disorder. I suggest illustrating chemically meaningful structures while indicating disorder using ellipsoids, as is common practice. Since the interactions and packing of the three systems are similar, I suggest to show only one in the main text and place the others in the Supporting Information, removing the extraneous atoms mentioned above, of course.

Thanks for the expert's good suggestion. We have increased the font size in all figures to improve clarity and readability. In addition, following the reviewer's suggestion, ellipsoids have been introduced to highlight disordered regions within the frameworks (Figs. R11a, d, and g).

Given the similar intermolecular interactions and packing modes among the three **HNNU-X** materials, we present the single-crystal X-ray diffraction structure of the representative **HNNU-Se**

in revised main text (Fig. 1b in page 6), while the structures of the other analogues are provided in revised Supporting Information (Supplementary Fig. 2 in page 7). For clarity, extraneous atoms have been omitted from these structural views.

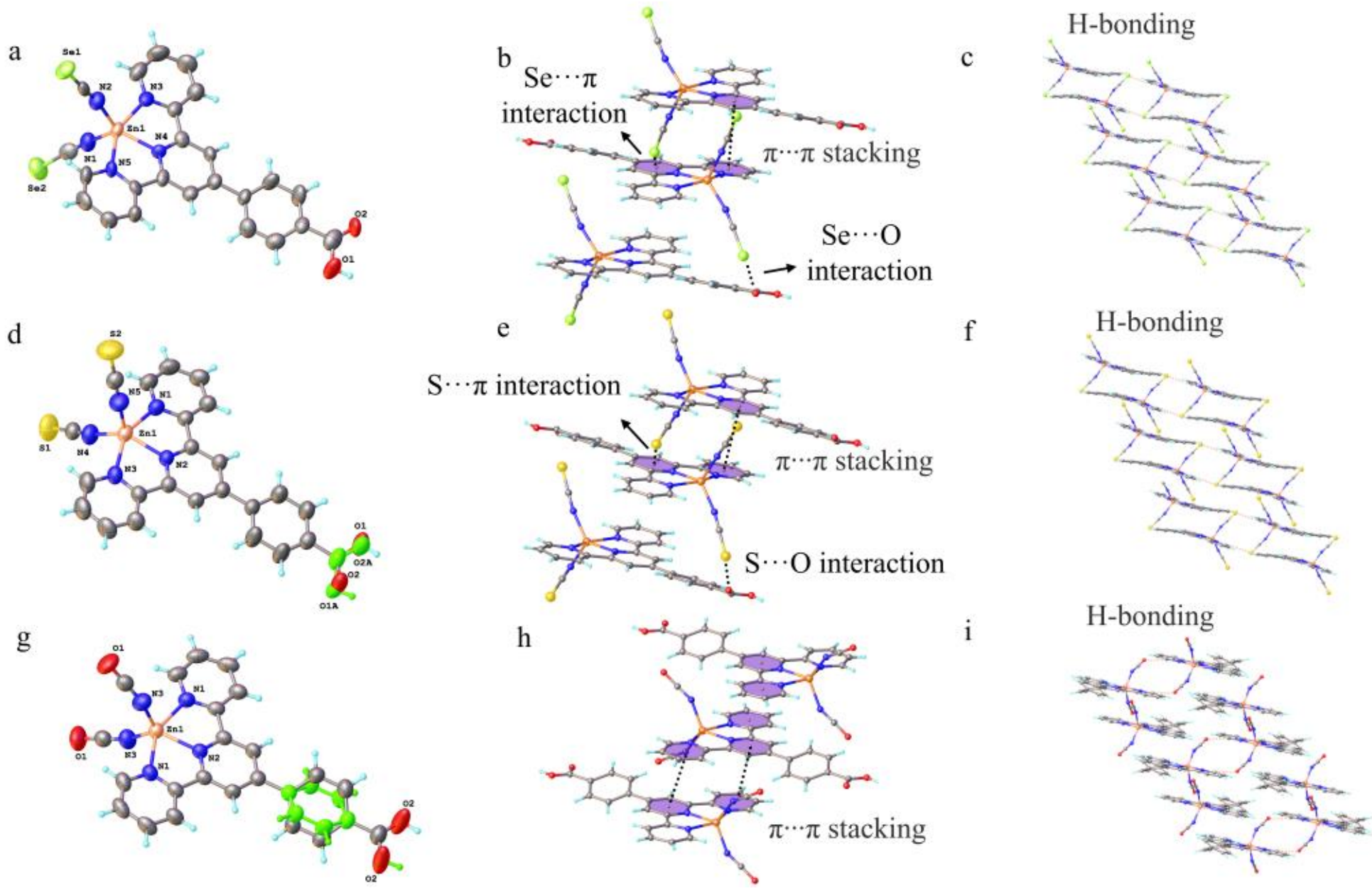

Fig. R11. Single-crystal X-ray diffraction view of the coordination mode of building blocks, the nodes and the topologies of **HNNU-Se** (a-c), **HNNU-S** (d-f), and **HNNU-O** (g-i) (gray C, cyan H, red O, blue N, yellow S, pale green Se, orange Zn, disordered components are shown in green).

16. The Reviewers **#3**: Line 202: The statement "the reduced band gap facilitates more efficient charge transfer and optical excitation processes.36" is an overgeneralization. A smaller band gap does not automatically imply improved charge separation or faster charge transfer; in fact, narrower band gaps can increase recombination rates. Reference 36 does not provide direct evidence supporting this claim for the current material. It would be more accurate to provide data demonstrating enhanced charge mobility or separation, or to soften the statement to indicate that a reduced band gap may increase light absorption without assuming improved charge transfer efficiency.

Thanks for the expert's good suggestion. Indeed, a smaller band gap does not automatically imply the improved charge separation or faster charge transfer, as it may also promote charge recombination. In accordance with the reviewer's suggestion, we have revised the statement to "The narrower band gap implies that less radiant energy is required for photoexcitation, which is advantageous for light absorption" in revised manuscript (lines 201-202 in page 11).

17. The Reviewers **#3**: Figure 4: No clear band gap can be seen in the density of states. Could this be a consequence of using overly broad Gaussian smearing?

Thanks for the expert's good suggestion. The unobvious band gap in Fig. 4 arises from the use of overly broad Gaussian smearing in the density of states (DOS) calculation. To visualize the HOMO-LUMO gap, we have revised the DOS plot using Multiwfn by reducing the full width at half maximum (FWHM) from the original 1.36 eV to 0.30 eV. Additionally, we have shifted the energy scale to align the HOMO level at 0 eV and explicitly marked the relative position of the

LUMO level (Figs. R13).

Importantly, this refinement markedly improves the clarity of the DOS plots without altering the relative orbital energies or the underlying computational results. The revised DOS data have been incorporated into the revised manuscript (Figs. 4d-f in page 12).

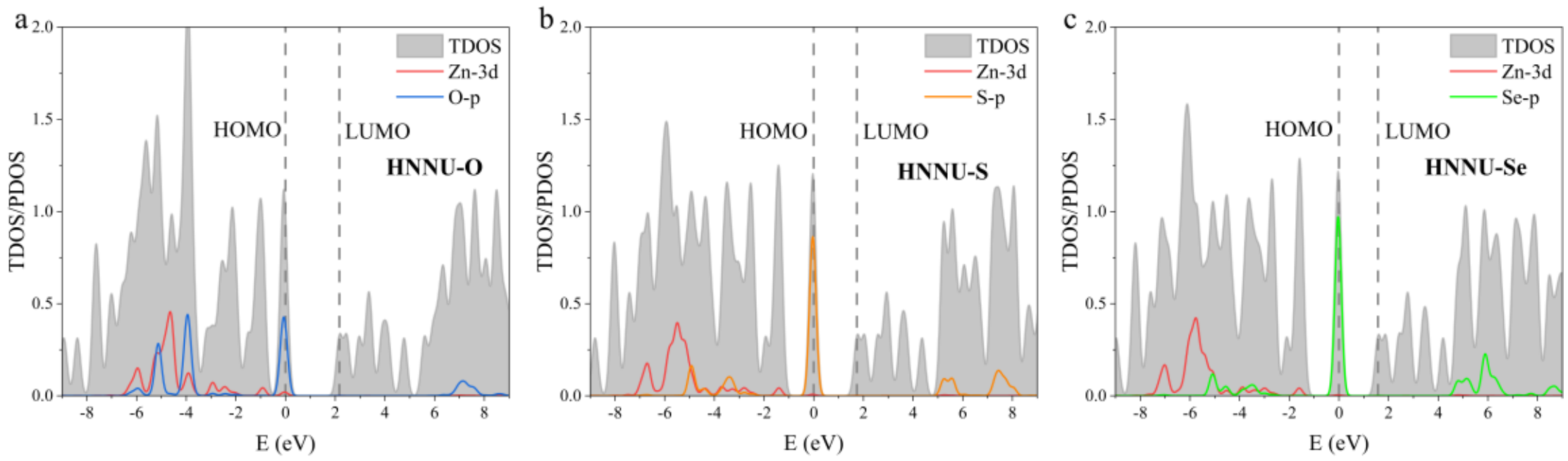

Fig. R13. Density of states of **HNNU-X** (X= O, S, Se)

18. The Reviewers **#3**: Figure 7a: The current free energy profile is extremely crowded, making it nearly impossible to read, with overlapping numbers, structures, and energy levels! I recommend simplifying or enlarging the figure to improve (or allow) visualization.

Thanks for your good suggestion. Figure 7a has been simplified to improve clarity and readability, as shown in Fig. R14. The revised version has been incorporated into the revised manuscript (Fig. 7a in page 20).

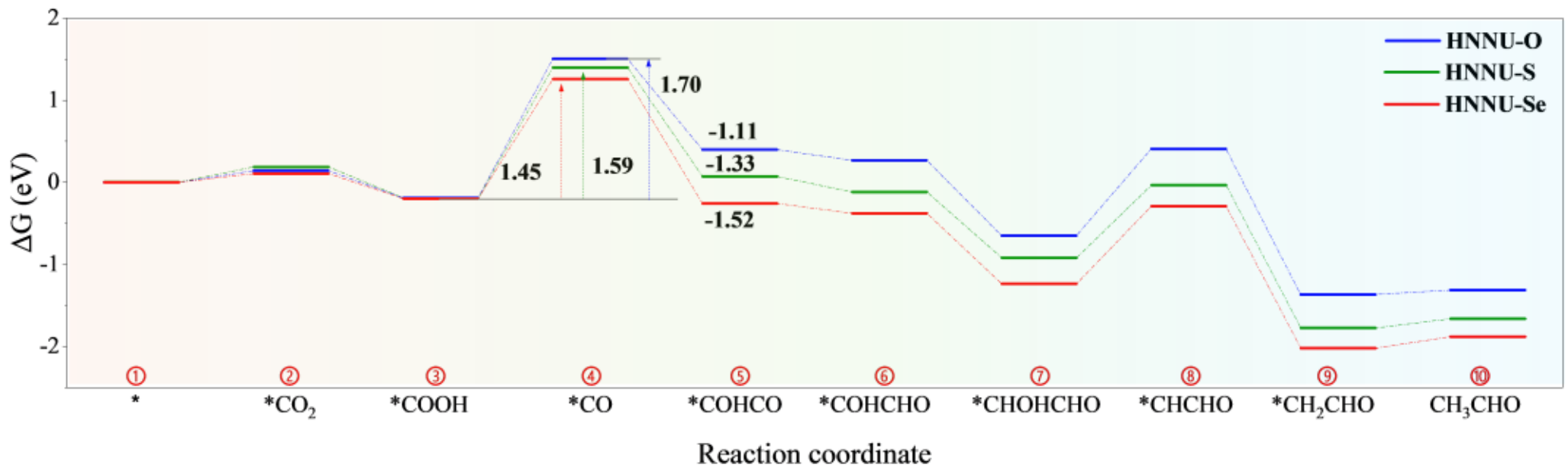

Fig. R14. Calculated free energy diagram for the reduction of CO_2 to CH_3CHO on the **HNNU-X** catalysts

19. The Reviewers **#3**: The labels in Figure 7 do not match those in Figures S32–S34.

I'm sorry for this mistake. We have checked the label carefully, and amended the labels in Figures S32–S34 according to Figure 7a, as shown in Fig. R15. The revised figures have been provided in revised Supporting Information (Supplementary Figs. 37-39 in pages 28-29).

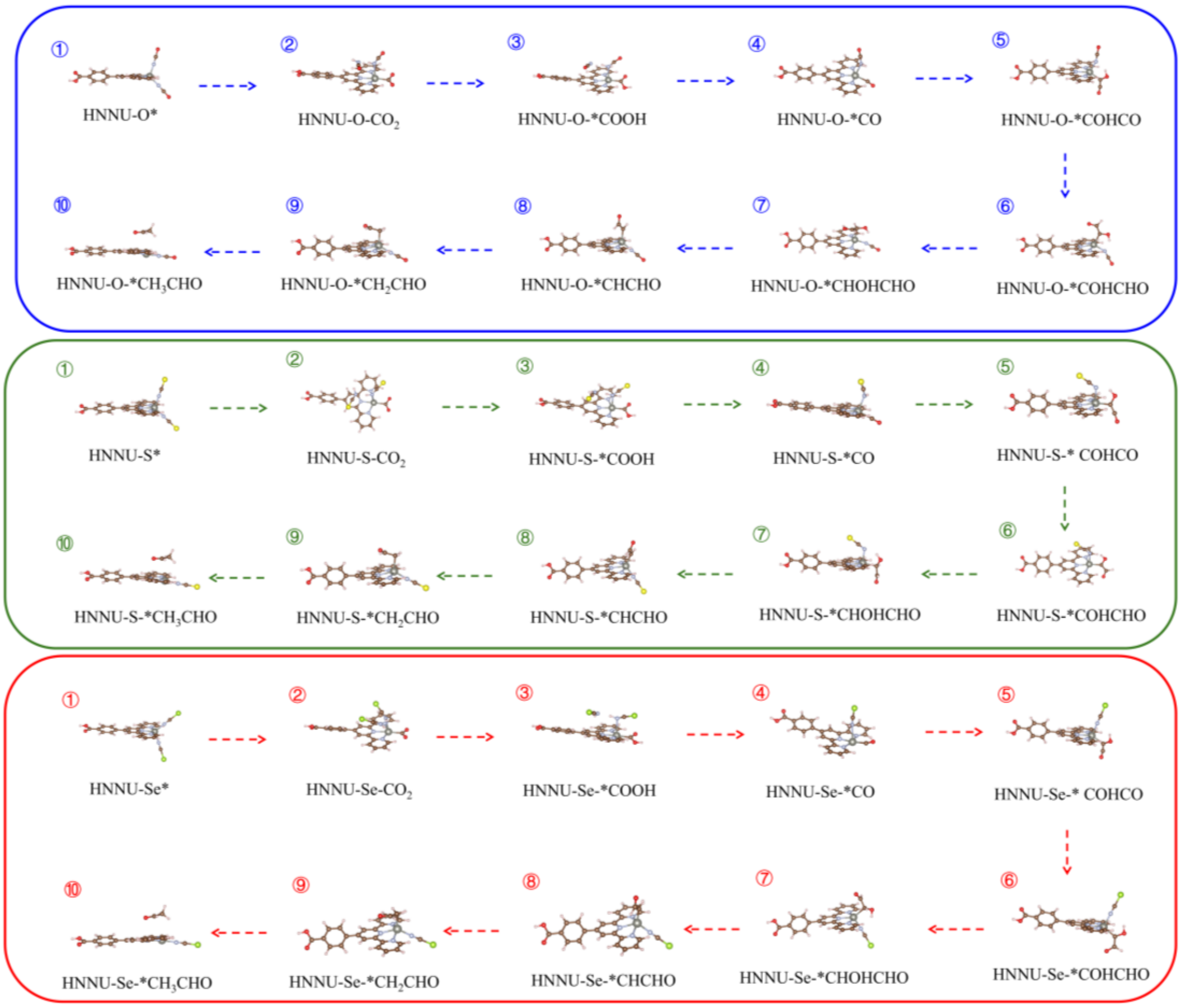

Figs. R15. DFT optimized structure models for every step of catalytic CO_2 reduction on **HNNU-O**, **HNNU-S** and **HNNU-Se**.

20. The Reviewers **#3**: Line 312: The statement "The formation of *CO from *COOH appears to be a rate-determining step, where **HNNU-Se** has the minimum energy barrier (ΔG = 1.45 eV)" is an abuse of language. The reported value represents a thermodynamic energy change, not a kinetic barrier, as no transition state is computed. Therefore, the energy levels in Figure 7a reflect thermodynamics, not kinetics, and the term "rate-determining step" is misleading. If the authors with to discuss kinetics, I suggest them to calculate the transition state for the C-O bond cleavage and check if it involves a free-energy barrier that can be surmountable. Note that the barrier height might depend on the redox state of the catalyst (ie. if it happens before or after a reductive quenching step of the *CO intermediate).

Thanks for the expert's good suggestion. We fully acknowledge the mistake in our original statement at Line 312: the reported value (ΔG = 1.45 eV) corresponds to a thermodynamic free energy change rather than a kinetic barrier, as transition state calculations were not performed in this work. This error led to the inappropriate use of kinetic-related terminology

In response to your valuable suggestion, we have thoroughly revised the relevant content in the manuscript: the incorrectly used terms "rate-determining step" and "minimum energy barrier" have been completely removed, and replaced with the more accurate thermodynamic descriptions "the most thermodynamically uphill step along the reaction pathway" and "minimum ΔG change", respectively. Corresponding revisions have also been made to the related descriptions of Figure 7a

to clearly clarify that the energy levels in the figure reflect thermodynamic characteristics of the reaction process rather than kinetic behavior in revised manuscript (lines 330-338 in page 18).

We would like to further clarify that the primary purpose of the free energy analysis in this work is to provide a comparative thermodynamic evaluation of different catalysts under the identical reaction environment, by quantifying the thermodynamic changes of each elementary step in the CO_2 photoreduction pathway for **HNNU-O**, **HNNU-S** and **HNNU-Se**. Kinetic aspects of the reaction, including transition state energy barriers and reaction rate characteristics, are not involved or discussed in the present study, and we have ensured that this research scope is clearly reflected in the revised manuscript to avoid any potential misunderstanding.

We thank you again for pointing out this critical issue, which has significantly enhanced the terminological precision and scientific rigor of our work. All related revisions have been marked in the revised manuscript for your easy checking.

21. The Reviewers **#3**: While the proposed reaction mechanism provides a reasonable bird-eye view of possible reaction intermediates that may be formed, it still leaves in a blind spot several key aspects of the mechanism, or how some reaction steps take place. For example, how do we go from *CO to *COHCO? Do we form a *CHO intermediate first (not shown in Figure 7a) which then couples with a neighboring *CO? Does the 3Dl structure of the material allow such coupling by placing two metal centers close enough? Or this rather happens on a single site bearing two *CO or a *CO + *CHO? Where do electrons come from to promote these steps? Should we assume that after the formation of each intermediate, the system absorbs light to form an excited state that can be reductively quenched by water?? I strongly believe that these aspects deserve deeper discussion.

Thanks for the expert's good suggestion. Regarding the mechanistic questions raised, we provide detailed explanations as follows:

1. Feasibility of dual-site C–C coupling

In our system, the transition-metal active centers are embedded in a large conjugated framework and coordinated with two additional ligands. Due to the steric hindrance imposed by this coordination environment, two adjacent metal centers cannot approach each other closely enough to enable dual-site C–C coupling between two adsorbed C_1 intermediates. We therefore rule out the coupling pathway between two C_1 species adsorbed on neighboring metal sites.

2. Adsorption limitation of single active site

We further examined whether a single metal center could stabilize two C1 intermediates simultaneously (e.g., *CO + *CO or *CO + *CHO). Our calculations show that such co-adsorption configurations are unstable: one *CO species desorbs spontaneously during structural optimization, owing to the limited coordination capacity of the metal center. Based on these results, we modeled the C–C coupling step following the Eley–Rideal (E–R) mechanism reported in previous literature [Activating *CO by strengthening Fe–CO π-backbonding to enhance two-carbon products formation toward CO_2 electroreduction on $Fe–N_4$ sites, *Adv. Funct. Mater.*, 2024, 34, 2402537; Nature of C-C coupling and strategy of tuning the catalytic activity of Cu-N-C catalysts for electro-reduction of CO_2 to ethanol, *Nano Energy*, 2024, 127, 109699], in which *CO molecule adsorbed on protonated $[XCN]^-$ anions couple directly with Zn-adsorbed *CO to form the C_2 intermediate. This interesting phenomenon reveals that the formation of C_2 products in this reaction system relies on the cooperation of C-C coupling with hydrogenation,

which is also consistent with the computational model we employed. During subsequent structural optimization, we observed spontaneous hydrogen transfer from *XCNH to the *CO moiety, leading to C-C coupling assisted by hydrogenation to form *COHCO. This mechanism is also consistent with our experimental observation that the fast proton transfer rate over **HNNU-Se** favors the formation of *COHCO, a key intermediate for the conversion of CO_2 to multicarbon (C_{2+}) products. We note that the intermediate was erroneously designated *COCHO in the original manuscript; this label has been corrected to *COHCO across the entire text and all figures.

3. Alternative pathways involving other intermediates

We also considered pathways involving *CO → *CHO and *CO → *COH intermediates. Calculations indicate that the *CO → *COH route is thermodynamically unfavorable, while the *CHO intermediate tends to undergo further hydrogenation to form C_1 products rather than contributing to C_2 generation. Notably, the transformation from *CO to *COHCO is a thermochemical process that does not involve distinct proton-coupled electron transfer steps. Therefore, the formation of the C_2 intermediate at this stage does not require photoexcitation or reductive quenching by water.

We sincerely thank the referee for these thoughtful and critical comments, which have greatly improved the mechanistic discussion and overall scientific quality of our manuscript. The discussion have provided in revised manuscript (lines 347-356 in page 19).

22. The Reviewers **#3**: Figure 7b is referenced after Figures 7c/d. Figures should follow a logical order in the text.

Thanks for the expert's good suggestion. Figure 7 has been replenished and rearranged in revised manuscript to ensure that the cited figures follow a logical sequence in the text, as shown in Fig. R17.

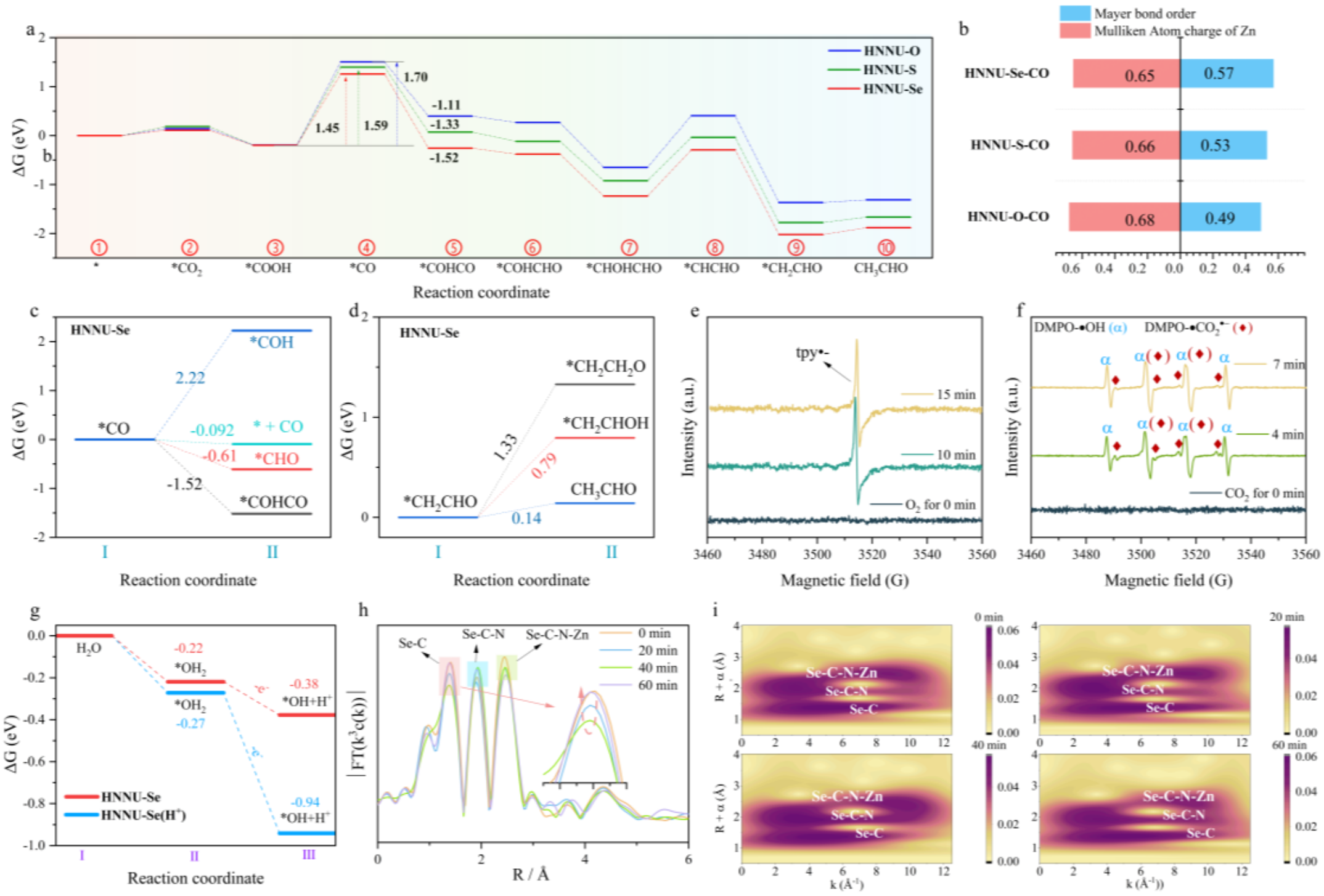

Figs. R15. Calculated free energy diagram for the reduction of CO_2 to CH_3CHO on **HNNU-X**

catalysts (a). Mayer bond orders and Mulliken atomic charges of **HNNU-X** with adsorbed CO (b). Gibbs free energy diagrams for the transformation of *CO into different intermediates over **HNNU-Se** (c). Gibbs free energy diagrams for the transformation of *CH_2CHO into different intermediates over **HNNU-Se** (d). In-situ EPR spectra over **HNNU-Se** in acetonitrile under O_2 atmosphere with TEOA as hole scavenger (e, DMPO was added as the radical trapping agent). In-situ EPR spectra over **HNNU-Se** in water under CO_2 atmosphere without any scavengers (f). Calculated free energy diagram for H_2O oxidation through the formation of *OH on **HNNU-Se** and **HNNU-Se(H^+)** (g). FT of K^3-weighted Operando Se K-edge EXAFS upon light irradiation for different time (h, the spectrum labeled "0 min" was obtained immediately after lighting, the spectra labeled "20 min", "40 min" and "60 min" were collected 20, 40 and 60 min after illumination, respectively). WTs for the k^3-weighted Se K-edge EXAFS signals under different conditions (i).

23. The Reviewers **#3**: In the computational details section:

- What solvent model was used (SMD, PCM, COSMO, ...)?
- Some references are missing (functional, basis set, quantum chemistry code, solvent model…).
- The experimental pH is not zero, yet the authors set pH = 0, making all ΔGpH corrections zero. If the correction is not formally applied, its inclusion is misleading.
- Cartesian coordinates for all computed structures should be included in the Supporting Information or preferably uploaded to an open repository to enable reproducibility.
- Information about charge and spin multiplicity of all computed species should be stated (perhaps a table in the SI, along with electronic and Gibbs free energies?)

Thanks for the expert's good suggestion. We have provided the corresponding computational details as follows:

1. Solvent model and missing references

In the revised Computational details section, we have clearly stated the solvent model used in our calculations, and provided appropriate references for the functional, basis sets, implicit solvation model, and quantum chemistry code employed throughout this work (lines 503-506 in page 26).

2. Treatment of pH effect

We fully agree with the referee's comment that the description of pH correction in the original manuscript was misleading.

In this work, our main purpose is to compare the relative free energy changes of different catalysts under the same reaction environment. Under such conditions, the pH-dependent correction term only introduces a constant shift in free energy and does not affect the qualitative trend among different catalysts. Therefore, no pH correction was applied in our calculations, and ΔGpH was set to 0 eV. To avoid confusion, we have removed the corresponding misleading description of pH correction in Computational details of the revised manuscript.

3. Cartesian coordinates of computed structures

Considering the large size of the relevant files, we have packaged all the log files containing Cartesian coordinates for easy checking (named "Cartesian coordinates of computed structures" as the attachment of Supplementary dataset).

4. Charge, spin multiplicity and energy data

We have added detail tables (Tables R3-6) in revised Supporting Information to summarize the

charge, spin multiplicity, electronic energy and Gibbs free energy of all computed species (Supplementary Tables 7-10 in pages 29-31).

We thank the referee again for these constructive suggestions, which have significantly enhanced the reproducibility and scientific rigor of our computational work.

Table R3. Gibbs free energies (G) of the molecules obtained form the electronic energies (E) with free-energy corrections (ΔGcorr (eV)) , together with their charges and spin multiplicities.

molecule	E (eV)	ΔGcorr (eV)	G (eV)	Charge	Spin multiplicity
CO	-3081.03	-0.38	-3081.41	0	1
CO_2	-5127.87	-0.24	-5128.11	0	1
H_2	-31.87	-0.04	-31.91	0	1
H_2O	-2077.50	0.06	-2077.44	0	1
OCNH	-4586.54	0.07	-4586.47	0	1
SCNH	-13373.26	-0.08	13373.35	0	1
SeCNH	-67883.30	-0.20	67883.50	0	1

Table R4. Gibbs free energies (G) of intermediates bound to **HNNU-O** obtained form the electronic energies (E) with free-energy corrections (ΔGcorr), together with their charges and spin multiplicities.

Intermediate	E (eV)	ΔGcorr (eV)	G (eV)	Charge	Spin multiplicity
HNNU-O	-89189.33	8.21	89181.12	0	1
HNNU-O-CO_2	-94317.47	8.39	94309.09	0	1
HNNU-O-COOH	-94334.05	8.67	94325.37	0	2
HNNU-O-CO	-87683.48	7.76	87675.72	0	1
HNNU-O-COHCO	-90766.36	8.12	90758.24	0	1
HNNU-O-CHO	-87700.36	8.06	87692.31	0	2
HNNU-O-COH	-87697.52	8.03	87689.49	0	2
HNNU-O-COHCHO	-90782.75	8.42	90774.33	0	2
HNNU-O-CHOHCHO	-90799.96	8.76	90791.20	0	1
HNNU-O-CHCHO	-88736.89	8.23	88728.66	0	2
HNNU-O-CH_2CHO	-88755.05	8.67	88746.39	0	1
HNNU-O-CH_2CH_2O	-88769.76	8.85	88760.91	0	2

HNNU-O-CH_2CHOH	-88770.50	8.86	88761.65	0	2
HNNU-O-CH_3CHO	-88771.15	8.86	88762.29	0	2

Table R5. Gibbs free energies (G) of intermediates bound to **HNNU-S** obtained form the electronic energies (E) with free-energy corrections (ΔGcorr), together with their charges and spin multiplicities.

Intermediate	E (eV)	ΔGcorr (eV)	G (eV)	Charge	Spin multiplicity
HNNU-S	106762.61	8.00	106754.61	0	1
HNNU-S-CO_2	111890.78	8.25	111882.53	0	1
HNNU-S-COOH	111907.31	8.44	111898.87	0	2
HNNU-S-CO	-96470.13	7.68	-96462.45	0	1
HNNU-S-COHCO	-99553.26	8.08	-99545.19	0	1
HNNU-S-CHO	-96486.85	8.13	-96478.73	0	2
HNNU-S-COH	-96484.13	7.93	-96476.20	0	2
HNNU-S-COHCHO	-99569.66	8.33	-99561.33	0	2
HNNU-S-CHOHCHO	-99586.78	8.69	-99578.09	0	1
HNNU-S-CHCHO	-97523.86	8.14	-97515.72	0	2
HNNU-S-CH_2CHO	-97542.00	8.59	-97533.41	0	1
HNNU-S-CH_2CH_2O	-97556.79	8.74	-97548.06	0	2
HNNU-S-CH_2CHOH	-97557.39	8.76	-97548.63	0	2
HNNU-S-CH_3CHO	-97558.03	8.77	-97549.26	0	2

Table R6. Gibbs free energies (G) of intermediates bound to **HNNU-Se** obtained form the electronic energies (E) with free-energy corrections (ΔGcorr), together with their charges and spin multiplicities.

Intermediate	E (eV)	ΔGcorr (eV)	G (eV)	Charge	Spin multiplicity
HNNU-Se	215782.59	7.89	215774.70	0	1
HNNU-Se-CO_2	220910.82	8.12	220902.70	0	1
HNNU-Se-COOH	220927.29	8.33	220918.96	0	2

HNNU-Se-CO	150980.16	7.64	150972.52	0	1
HNNU-Se-COHCO	154063.43	7.98	154055.45	0	1
HNNU-Se-CHO	150996.98	7.89	150989.09	0	2
HNNU-Se-COH	150994.12	7.86	150986.25	0	2
HNNU-Se-COHCHO	154079.80	8.27	154071.53	0	2
HNNU-Se-CHOHCHO	154096.98	8.64	154088.34	0	1
HNNU-Se-CHCHO	152034.00	8.09	152025.91	0	2
HNNU-Se-CH_2CHO	152052.12	8.52	152043.60	0	1
HNNU-Se-CH_2CH_2O	152066.86	8.64	152058.22	0	2
HNNU-Se-CH_2CHOH	152067.52	8.76	152058.76	0	2
HNNU-Se-CH_3CHO	152068.14	8.73	152059.41	0	2

25. The Reviewers **#3**: Finally, the manuscript would also benefit from a general revision of English, as there are numerous grammatical issues throughout. The Reviewers **#4**: Please check the language carefully.

Thanks for the referee's good suggestion. We have polished the manuscript, and checked the grammar and expression carefully.

Reviewers #4

26. The Reviewers **#4**: The authors described the MHOFs as "bioinspired metalo hydrogen-bonded organic frameworks". What is the resemblance between MHOFs and natural enzymes for photosynthesis?

Thanks for the referee's good suggestion. In nature, green plants harness solar energy to convert atmospheric CO_2 and water into carbohydrates and biomass. The natural plants have evolved a special CO_2-fixation enzyme that can concentrate CO_2 from atmospheric levels up to 1000-fold near the active sites [Enhancing photosynthetic CO_2 fixation by assembling metal-organic frameworks on Chlorella pyrenoidosa, *Nat. Commun.*, 2023, 14, 5337]. The enriched CO_2 is subsequently transformed in situ into biomass via photosynthetic pathways. Analogously, in the tailored MHOF system, $[Zn(tpy)]^{2+}$ cations are incorporated to chemically capture and activate CO_2 during photocatalysis. The resulting frameworks exhibit high CO_2 binding affinity and selective adsorption capacity, thereby facilitating the efficient conversion of low-concentration CO_2 in air.

Furthermore, a major challenge in artificial CO_2 reduction is the competing hydrogen evolution reaction (HER), which involves a kinetically favorable two-electron/two-proton process compared to the multielectron, multiproton reduction of CO_2. In biological systems, this challenge is addressed by reducing CO_2 not with H_2O but rather with hydrogen (H^+) in the form of NADPH/H^+ arisen from solar water splitting [Artificial photosynthesis at efficiencies greatly exceeding that of natural photosynthesis, *Acc. Chem. Res.*, 2019, 52, 3143-3148]. Inspired by this strategy, in MHOFs the $[XCN]^-$ anions function as proton shuttles, rapidly transferring in situ generated H^+

from water oxidation to adsorbed CO_2. Consequently, CO_2 reduction proceeds via hydrogen equivalents in the form of $N(H^+)$=C=Se/H^+ rather than direct interaction with water, effectively suppressing the competing HER.

Thus, mimicking key features of natural enzymatic systems, the customized MHOF platform utilizes only sunlight, air, and water as inputs to achieve efficient carbon fixation and conversion into value-added chemicals.

11. The Reviewers #4: How can Zn^{2+} ion participate in the photocatalytic CO_2 reduction? Authors should perform the in-situ mechanistic study to clarify the change of coordination environment and electron density of Zn^{2+} ion during the CO_2 photoreduction.

Thanks for the referee's good suggestion. Operando XAFS was employed to probe the electronic structure and local coordination environment of Zn centers in **HNNU-Se** during photocatalytic CO_2 reduction. In the Zn K-edge XANES spectra (Fig. R18a), the white-line intensity gradually decreases over 40 min of illumination, indicative of increased electron density at Zn and/or the coordination of CO_2 and *CO intermediates to Zn^{2+} sites. Upon prolonged irradiation (40 min), the white-line intensity recovers, likely reflecting the desorption of the generated reduction products from the Zn^{2+} centers.

The local structure of Zn was further analyzed by FT-EXAFS. The pristine **HNNU-Se** exhibits a dominant peak at ~1.46 Å, corresponding to the Zn–N coordination distance ($d_{Zn\text{-}N}$). Under illumination, this distance increases to ~1.56 Å after 40 min (Fig. R18b). Consistently, EXAFS fitting reveals an increase in the coordination number of Zn^{2+} from 5.2 to 5.9 (Fig. R18c and Table R7), which can be attributed to the coordination of CO_2 and/or *CO intermediates at the Zn center. With further irradiation, the Zn–N distance decreases, accompanied by a reduction in coordination number (Figs. R18b, c and Table R7), indicating the restoration of Zn^{2+} coordination environment. These local coordination change provides compelling evidence that Zn^{2+} centers serve as the active sites during photocatalytic CO_2 reduction. The operando XAFS results have been incorporated into the revised manuscript (Figs. 6c and d in page 16) and the revised supporting Information (Supplementary Figs. 35 and 36 in page 27, and Supplementary Table 6 in page 28).

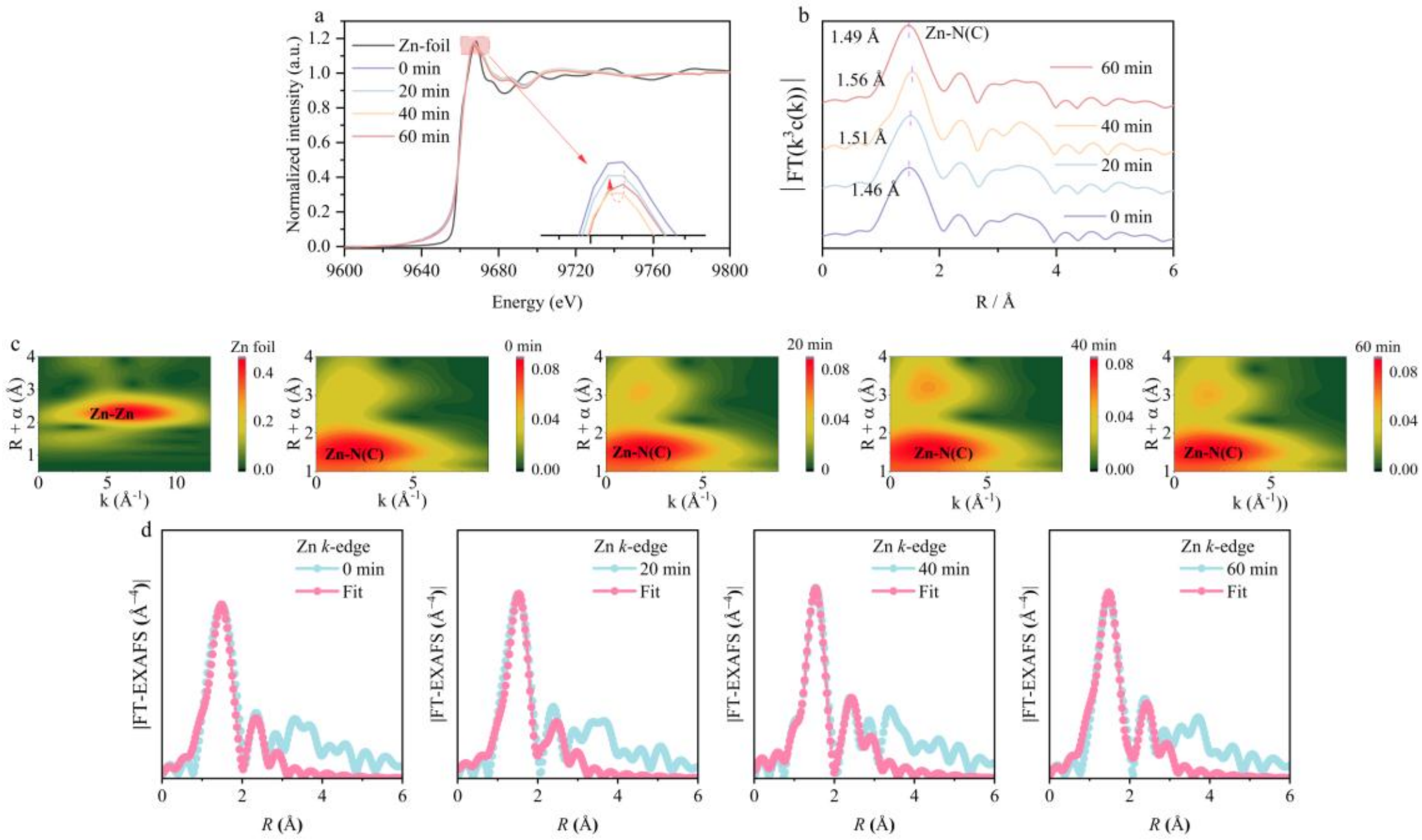

Fig. R18. In-situ Zn K-edge XANES spectra with inset showing white-line peak (a). FT of K^3-weighted Zn K-edge EXAFS upon light irradiation for different time (b). WTs for the k^3-weighted Zn K-edge EXAFS signals under different reaction conditions (c). EXAFS R-space fitting curves of **HNNU-Se** at different states (d).

Table. R7. Zn K-edge EXAFS fitting results at different reaction time periods.

Irradiation time (min)	Path	CN[a]	R (Å)[b]	σ^2 (Å^2)[c]	E_0 (eV)[d]	R factor[e]
0	Zn-N/C	5.2	2.02	0.008	0.5	0.019
20	Zn-N/C	5.6	2.08	0.005	2.00	0.019
40	Zn-N/C	5.9	2.09	0.008	1.06	0.009
60	Zn-N/C	5.4	2.02	0.010	3.5	0.020

[a] CN.: coordination number.

[b] R: the distance between absorber and backscatter atoms.

[c] σ2: Debye-Waller factors.

[d] ΔE: the inner potential correction.

[e] R factor: goodness of fit.

30. The Reviewers **#4**: Could the acetaldehyde production rate be enhanced by applying visible light without compromising selectivity?

Thanks for the referee's good suggestion. Visible light irradiation enhances the acetaldehyde production rate while maintaining high selectivity. As shown in Table R8, upon irradiation with 300 W Xe lamp ($\lambda \geq 420$ nm), the acetaldehyde production rate reaches 602.8 μmol.g^{-1}.h^{-1} (with an electron selectivity of 94.3%) in ambient air and 721.2 μmol.g^{-1}.h^{-1} (with an electron selectivity of 93.0%) in pure CO_2 atmosphere, respectively.

Table R8. Photocatalytic activity comparison of **HNNU-Se** under different reaction conditions.

Entry	Catalyst	Yield. (μmol^{-1}.g^{-1}.h^{-1})	Sel. (%)	Main product	Atmosphere	Light
1	**HNNU-Se**	557.1	95.1	CH_3CHO	air	Natural sunlight
2	**HNNU-Se**	684.1	93.1	CH_3CHO	CO_2	Natural sunlight
3	**HNNU-Se**	602.8	94.3	CH_3CHO	air	300 W Xe lamp
4	**HNNU-Se**	721.2	93.0	CH_3CHO	CO_2	300 W Xe lamp

Reaction conditions (unless otherwise specified): **HNNU-Se** (5 mg), deionized water (2.5 mL), air or CO_2 atmosphere, natural sunlight or 300 W Xe lamp, reaction temperature (25 °C).

31. The Reviewers #4: Can authors analyze the oxidation product, eg. O_2 gas evolution? Does it follow the stoichiometric chemistry?

Thanks for the referee's good suggestion. We have analyzed O_2 gas evolution over **HNNU-Se** under a CO_2 atmosphere by gas chromatography, and found that O_2 amounts gradually increased with reaction time. This suggests that O_2 originates from H_2O oxidation during CO_2 photoreduction. Notably, the O_2 evolution did not exhibit a stoichiometric correlation with acetaldehyde formation: the electrons consumed for acetaldehyde production exceeded the holes accounted for by O_2 generation. This discrepancy likely arises from hole consumption in forming H_2O_2 [Boosting interfacial charge-transfer kinetics for efficient overall CO_2 photoreduction via rational design of coordination spheres on metal-organic frameworks, *J. Am. Chem. Soc.*, 2020, 142, 12515−12523] and other reactive species such as ·OH [Near-infrared-activated multiphoton photocatalytic system for synergistic therapy via CO generation and ·OH production, *Angew. Chem. Int. Ed.*, 2026, e24756]. Indeed, both H_2O_2 and ·OH were detected as oxidation products of H_2O in the CO_2 photoreduction system.

A list of changes in revised manuscript No. NCOMMS-25-099726

page	line	Highlight changes in the revised manuscript
6	106-109	In **HNNU-O**, the [Zn(tpy)](OCN)$_2$ units assemble into supramolecular ribbon chains through π–π stacking interactions (Fig. 1b). In **HNNU-S** and **HNNU-Se**, the presence of σ-holes on S and Se enables additional directional interactions,[27] including chalcogen···π (with aromatic rings) and chalcogen···O (with carboxyl groups),[28] which cooperate with π–π stacking to stabilize the ribbon motifs (Fig. 1b and Supplementary Fig. 2e).
8-9	148-152	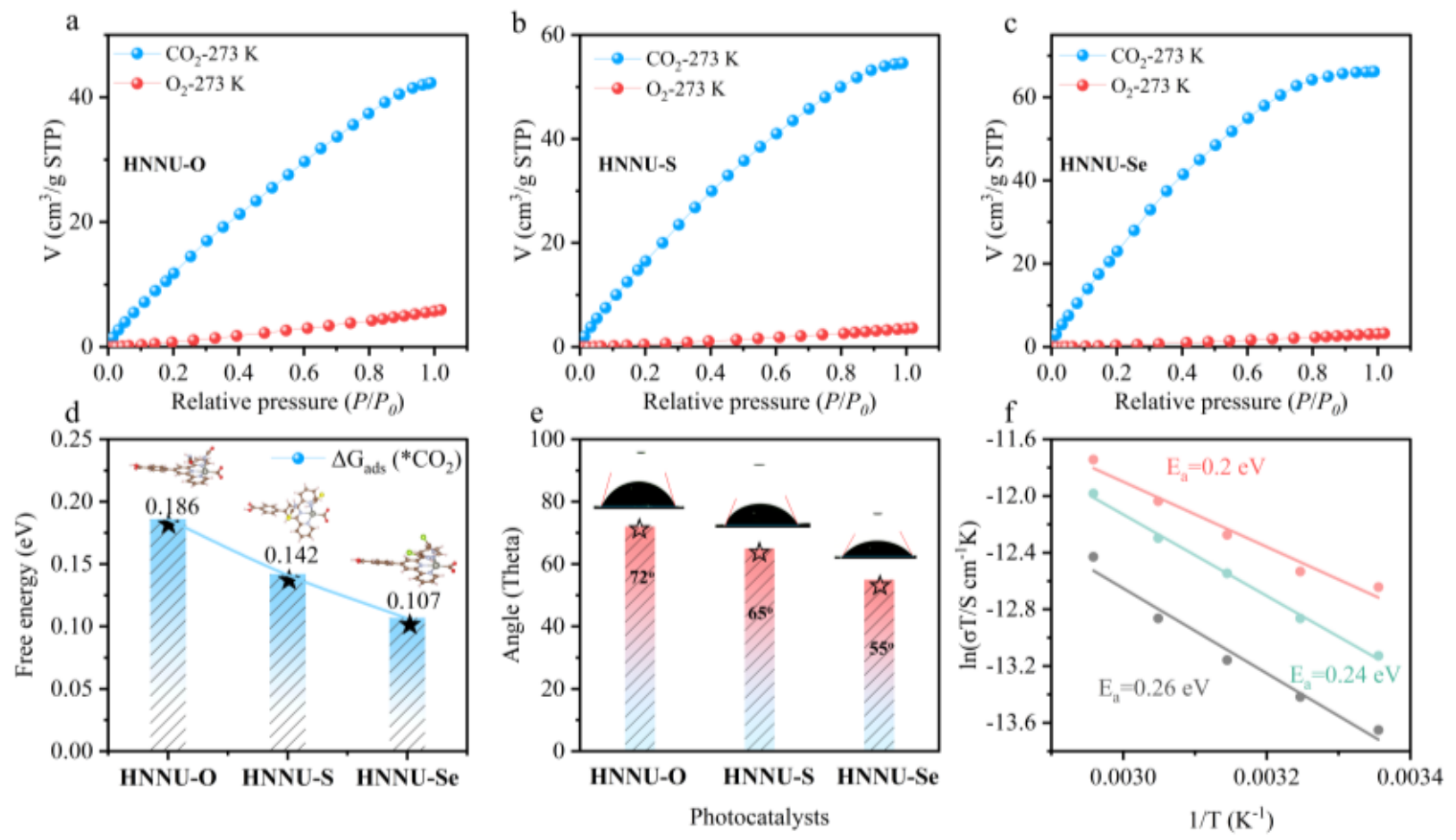<br>**Fig. 2 | Selective CO_2 capture and H_2O adsorption. a-c** The selective CO_2/O_2 adsorption studies for **HNNU-O**, **HNNU-S**, and **HNNU-Se** at 273 K. **d** Comparison of the calculated adsorption free energies of CO_2 on **HNNU-O**, **HNNU-S**, and **HNNU-S**. **e** WCA values of **HNNU-X**. **f** The corresponding Arrhenius plots of **HNNU-X** in proton conductivities.
11	196-197	an efficient electron transfer pathway from the XCN$^-$ anions to [Zn(tpy)]$^{2+}$ centers upon photoexcitation.
11	201-202	The narrower bad gap implies the less radiant energy required for photon excitation, which is beneficial for light absorption.

12	209	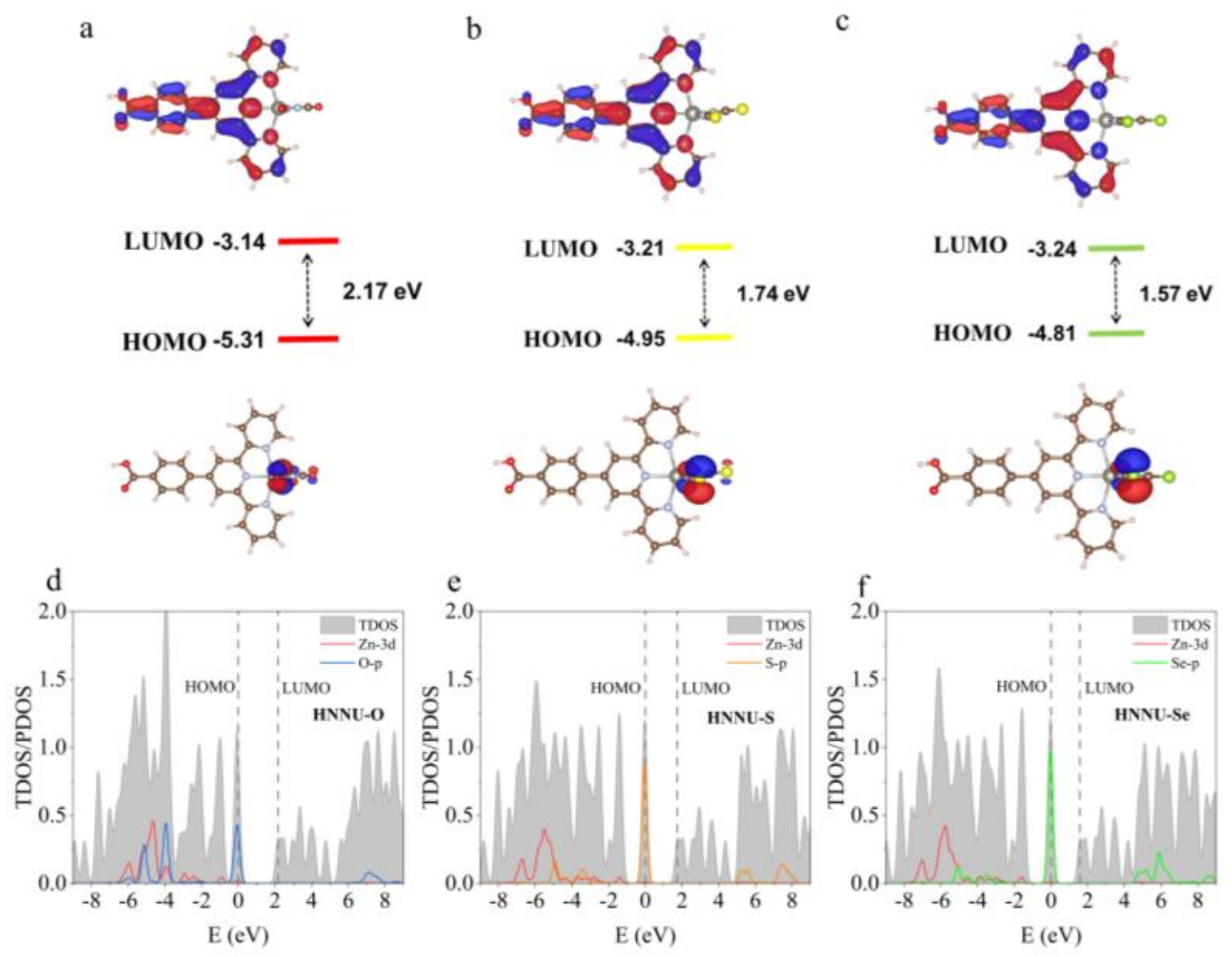
12	218-220	The product was unambiguously identified by gas chromatograph-mass spectrometer (GC-MS) (Figs. 5c and d), with a dominant GC peak at 3.03 min and a molecular ion at m/z = 44, consistent with acetaldehyde.
13	224	The labelled products were determined by GC-MS analysis (Supplementary Fig. 20).
13	225-233	The signal shifts to 48 with the use of D_2O, verifying that the hydrogen in CH_3CHO is derived from H_2O (Fig. 5e). The appearance of +1 amu fragments in the MS is likely due to residual, pre-adsorbed H_2O from ambient air, due to the high water affinity of **HNNU-X**. By replacing $^{12}CO_2$ with $^{13}CO_2$, the peak at m/z= 44 shifts to 46, corresponding to $^{13}CH_3{}^{13}CHO$ (Fig. 5f). Similarly, the presence of a minor m/z = 45 signal is attributed to partially labeled products ($^{13}CH_3{}^{12}CHO$ or $^{12}CH_3{}^{13}CHO$) arising from pre-adsorbed $^{12}CO_2$ on catalyst surface. The protons of ^{13}C-labeled CH_3CHO show ^{13}C-induced splitting in ^1H NMR spectrum (Fig. 5g). ^{13}C NMR spectrum of ^{13}C-labelled CH_3CHO (Fig. 5h) also shows characteristic isotope-induced splitting at ~19.4 and 98.9 ppm, attributable to the $^{13}CH_3$ and ^{13}CHO carbons in $^{13}CH_3{}^{13}CHO$, respectively.
14	246-249	**HNNU-Se** achieves 721.2 μmol g^{-1} h^{-1} of CH_3CHO production rate when the PCO_2R operates under pure CO_2 atmosphere and visible light illumination (Fig. 5a). Furthermore, the activity outperforms the state-of-art photocatalysts for CO_2 reduction to other C_2 products without the add of sacrificial agent (Fig. 5k, Supplementary Table 4).

<table>
<tr><td></td><td></td><td></td></tr>
<tr><td>14-15</td><td>254-263</td><td>
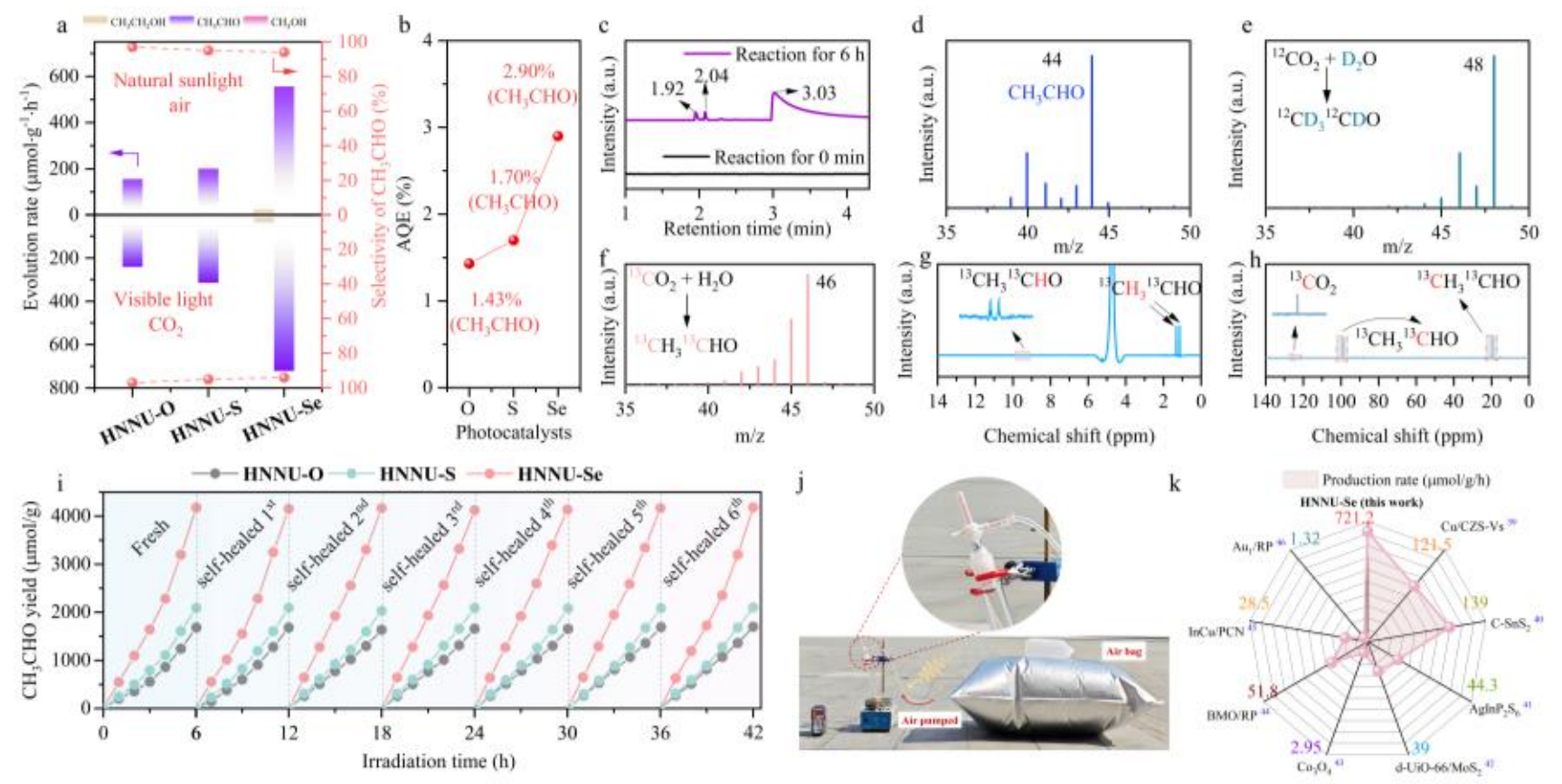

Fig. 5 | Overall photocatalytic CO_2 reduction performance of HNNU-X. a Photocatalytic CO_2 reduction activity of HNNU-X in air under natural sunlight irradiation for 6 h or in pure CO_2 under 300 W Xe lamp irradiation (λ>420 nm) for 6 h. b The catalytic quantum efficiency (AQE) of HNNU-X in the presence of a bandpass filter of 450 nm. c-d GC-MS spectra of liquid product of photocatalytic CO_2 reduction with H_2O in air over HNNU-Se. e MS spectra of produced acetaldehyde from CO_2 coupled with D_2O, f MS spectra of produced acetaldehyde from $^{13}CO_2$ coupled with H_2O. g ^1H NMR and f ^{13}C NMR of the liquid products from $^{13}CO_2$ photoreduction over HNNU-Se. i Reusability of HNNU-X upon self-healing. j Photocatalytic CO_2 reaction operates under air atmosphere and natural sunlight. k Comparison of the state-of-the-art catalysts with this work. Reaction conditions (unless otherwise specified): catalyst (5 mg), deionized water (2.5 mL), air atmosphere, natural sunlight, reaction temperature (25 ^oC).
</td></tr>
<tr><td>15</td><td>270-272</td><td>ICP-OES analysis gave Se content in self-healed HNNU-Se of 22.18 wt%, which was almost identical to that of the fresh one (22.23 wt%). Negligible amount of Se was detected in the supernatant according to ICP-OES analysis, ruling out the leaching loss of Se species in the auqeous photocatalytic CO_2 reduction.</td></tr>
<tr><td>15-17</td><td>276-306</td><td>In-situ X-ray absorption fine structure (XAFS) was employed to probe charge transfer in HNNU-X (X= O, S, Se). As shown in Fig. 6a, the Zn K-edge X-ray absorption near-edge structure (XANES) exhibits a pre-edge feature at ~9661.3 eV, characteristic of Zn(II) specie.[48] Upon illumination, the absorption edge shifts to lower energy with a concomitant decrease in white-line intensity, indicating an increase in electron density around Zn^{2+} centers. In contrast, the Se K-edge XANES shows a positive shift in absorption edge and enhanced white-line intensity under light irradiation (Fig. 6b), reflecting a decrease in electron density at $SeCN^-$ moieties. These in-situ XAFS results provide direct evidence for photoinduced electron transfer from XCN^- anions to $[Zn(tpy)]^{2+}$ center, in agreement with DFT-predicted frontier orbital distribution. This directed charge transfer within HNNU-X (X= O, S, Se) was also confirmed by the negative shifts in</td></tr>
</table>

binding energy of Zn^{2+} species, accompanied by positive shifts in binding energies of X elements (O, S, or Se) in corresponding XCN^- anions in in-situ XPS measurement (Supplementary Fig. 14).[49]

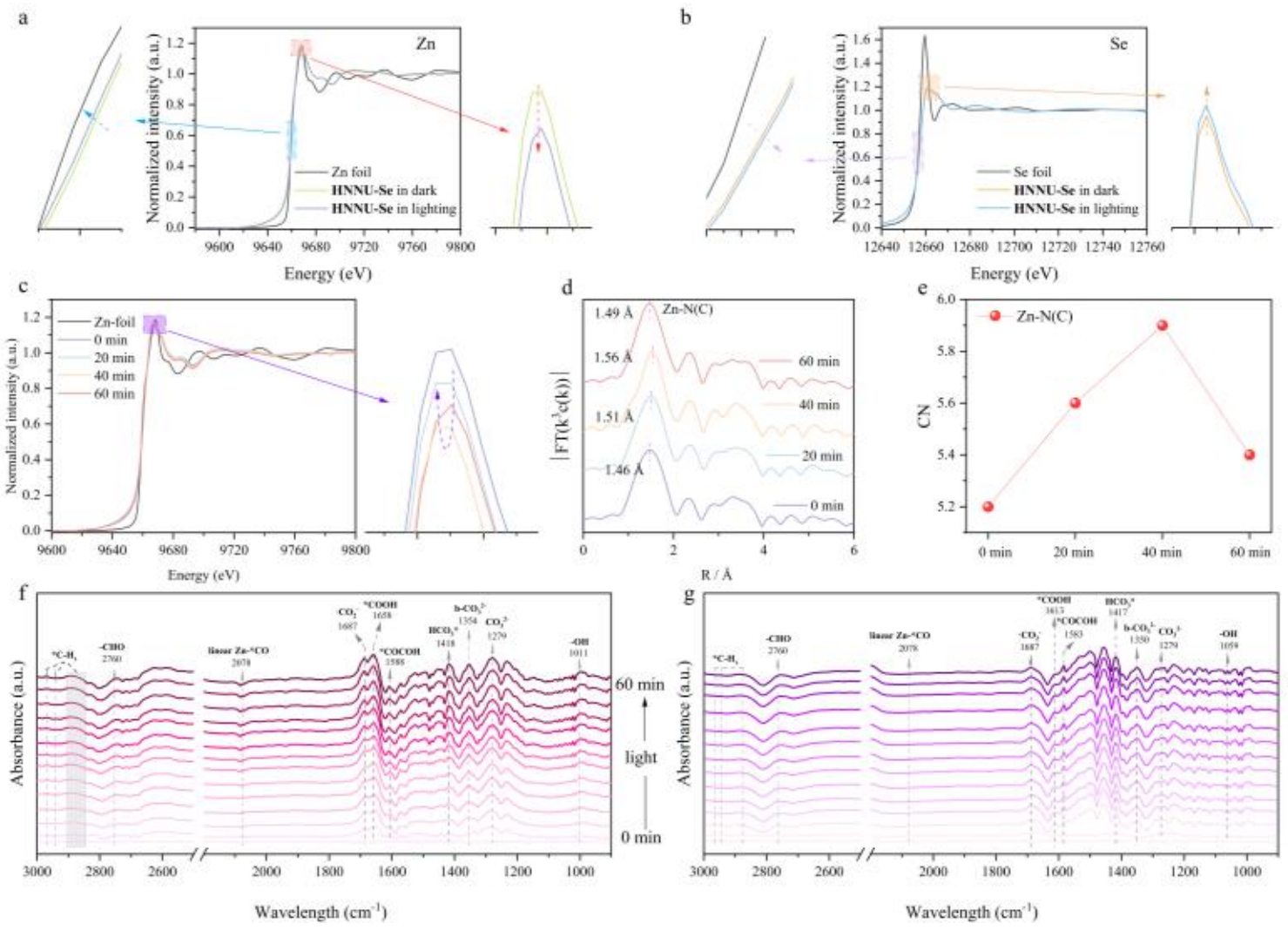

Fig. 6 | In-situ XAFS and in-situ DRIFTS measurement. a, b In-situ Zn and Se K-edge XANES spectra of **HNNU-Se** before and after light irradiation. **c** Operando Zn K-edge XANES spectra with inset showing white-line peak. **d** FT of K^3-weighted Operando Zn K-edge EXAFS upon light irradiation for different time (c) (The spectrum labeled "0 min" was obtained immediately after lighting. The spectra labeled "20 min", "40 min" and "60 min" were collected 20, 40 and 60 min after illumination, respectively). **e** Fitted changes in coordination number (CN) of Zn-N(C) shells at different states. **f** In-situ DRIFTS spectra for photocatalytic CO_2 conversion over **HNNU-Se. g** In-situ DRIFTS spectra for photocatalytic CO_2 conversion over **HNNU-O**.

Operando XAFS further reveals the change in electronic structure and local coordination environment of Zn center in **HNNU-Se** during photocatalytic CO_2 reduction. The measurement was done from 0 to 60 min, and spectra were collected after an interval of 20 min. In Zn K-edge XANES spectra (Fig. 6c), the white-line intensity gradually decreases during the initial 40 min of illumination, reflecting increased electron density and/or coordination of CO_2-derived intermediates at Zn sites, and subsequently recovers upon product desorption. Local structure of Zn was further analyzed by extended X-ray absorption fine structure (EXAFS). The pristine **HNNU-Se** exhibits a dominant peak at ~1.46 Å, corresponding to the Zn–N coordination distance ($d_{Zn\text{-}N}$). Under illumination, this distance increases to ~1.56 Å after 40 min (Fig. 6d, and Supplementary Fig. 35). Consistently, EXAFS fitting reveals an increase in the coordination number of Zn^{2+} from 5.2 to 5.9 (Fig. 6e, Supplementary Fig. 36 and Supplementary Table 6), which can be attributed to the coordination of CO_2-derived intermediates at Zn sites. With further irradiation, the Zn–N distance decreases, accompanied by a reduction in coordination number (Figs. 6d

		and e, Supplementary Fig. 36 and Supplementary Table 6), indicating the restoration of Zn^{2+} coordination environment. These local coordination change provides evidence that Zn^{2+} centers act as the catalytic sites for CO_2 activation and conversion.
18	324-327	All the computations have been performed within the def2-SV(P)[54,55] basis and B3LYP[56] functional using Gaussian 16 software.[57] All calculations used the DFT-D3[58] method to describe weak interactions within the system. To account for the solvation effects, all geometry optimizations and frequency calculations were performed with the IEF-PCM model using water as solvent.
18	330-338	The conversion of *COOH to *CO is identified as the most thermodynamically uphill step along the reaction pathway, where **HNNU-Se** has the minimum Δ*G* change (Δ*G* =1.45 eV). To further probe *CO formation, Mulliken charge and Mayer bond order (MBO) analyses were conducted for the **HNNU-X-*CO** intermediates. As shown in Fig. 7b, the Zn centers in these three **HNNU-X-*CO** intermediates carry positive Mulliken charges, revealing that *CO preferentially coordinates to Zn sites. Notably, the Zn atom in **HNNU-Se-*CO** (0.65552) is less positively charged than in **HNNU-S-*CO** (0.66076) and **HNNUO-*CO** (0.68062), suggesting stronger electron donation and enhanced Zn–CO interaction. Consistently, the MBO of Zn–C bond in **HNNU-Se-*CO** (0.57) exceeds those in **HNNU-S-*CO** (0.53) and **HNNUO-*CO** (0.49) (Fig. 7b), indicative of a more stabilized *CO intermediate. This stabilization accounts for the reduced Δ*G* associated with *CO formation on **HNNU-Se**.
19	347-356	To elucidate the C–C coupling mechanism from *CO to *COHCO, we first considered conventional dual-site pathways. Owing to steric constraints imposed by the coordination environment, adjacent metal centers cannot approach sufficiently to enable coupling between two adsorbed C_1 intermediates, thereby excluding dual-site mechanisms. We further evaluated the possibility of co-adsorption of two C_1 species (e.g., *CO + *CO or *CO + *CHO) on a single metal center; however, such configurations are unstable, with one *CO spontaneously desorbing during structural optimization due to the limited coordination capacity of Zn. Based on these results, we propose an Eley–Rideal (E–R)–type mechanism, consistent with prior reports,[62] in which a *CO species adsorbed on protonated $[XCN]^-$ anions directly couples with a Zn-bound *CO to generate a C_2 intermediate. Notably, structural optimization reveals spontaneous hydrogen transfer from *XCNH to the *CO moiety, promoting hydrogenation-assisted C–C coupling to form *COHCO.

20-21	369-379	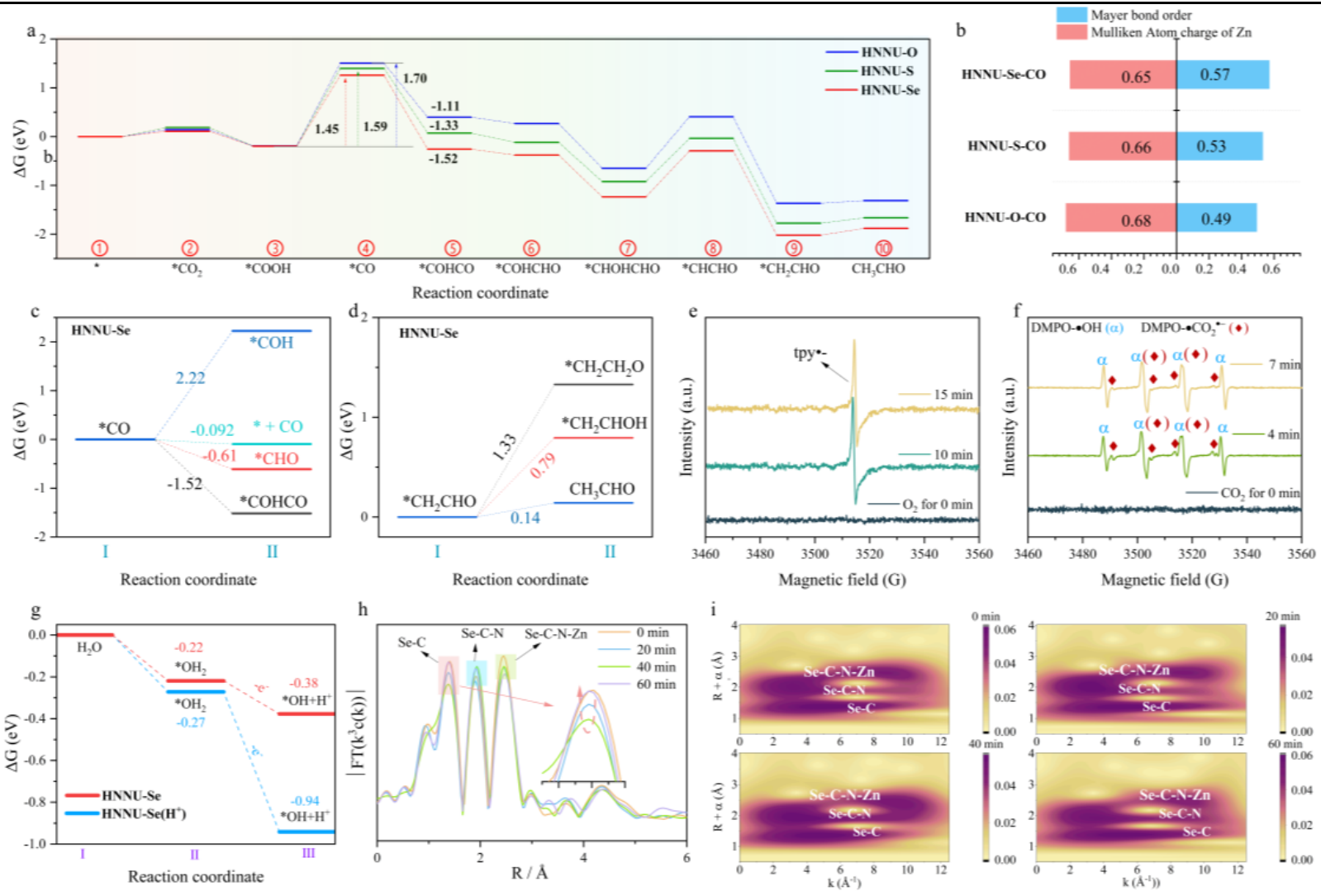 **Fig. 7 | Mechanism studies. a** Calculated free energy diagram for the reduction of CO_2 to CH_3CHO on **HNNU-X** catalysts. **b** Mayer bond orders and Mulliken atomic charges of **HNNU-X** with adsorbed CO. **c** Gibbs free energy diagrams for the transformation of *CO into different intermediates over **HNNU-Se**. **d** Gibbs free energy diagrams for the transformation of $*CH_2CHO$ into different intermediates over **HNNU-Se**. **e** In-situ EPR spectra over **HNNU-Se** in acetonitrile under O_2 atmosphere with TEOA as hole scavenger (DMPO was added as the radical trapping agent). **f** In-situ EPR spectra over **HNNU-Se** in water under CO_2 atmosphere without any scavengers. **g** Calculated free energy diagram for H_2O oxidation through the formation of *OH on **HNNU-Se** and **HNNU-Se(H$^+$)**. **h** FT of K^3-weighted Operando Se K-edge EXAFS upon light irradiation for different time (the spectrum labeled “0 min” was obtained immediately after lighting, the spectra labeled “20 min”, “40 min” and “60 min” were collected 20, 40 and 60 min after illumination, respectively). **i** WTs for the k^3-weighted Se K-edge EXAFS signals under different conditions.
21	382-395	To exclude the possibility that H_2O_2 originates from O_2 reduction via superoxide intermediates, in-situ EPR spectroscopy was employed to probe O_2 reduction pathway in acetonitrile (experimental details in Section S1.2 in the Supplementary Information). No superoxide ($•O_2^-$) signals are detected in O_2-saturated acetonitrile in the presence of triethanolamine (TEOA), thereby excluding the O_2 reduction pathway for $•O_2^-$ formation over **HNNU-Se** (Fig. 7e). In contrast, characteristic signals assigned to DMPO–•OH adducts[63,64] (pear-shaped features) emerge in in-situ EPR spectra over **HNNU-Se** in water under CO_2 atmosphere without any scavengers (Fig. 7f). The DMPO–•OH signals intensify upon light irradiation (Fig. 7f), confirming the generation of •OH radicals via water oxidation. These observations demonstrate that H_2O_2 is produced from water oxidation rather than O_2 reduction during photocatalysis. These results demonstrate that **HNNU-Se** simultaneously drives CO_2 reduction to CH_3CHO via photogenerated electrons and H_2O oxidation to •OH via hole-mediated

		pathways. Indeed, a sextet signal attributed to •CO_2 radicals (square-marked) appears accompanied by •OH radicals,[65] indicating efficient electron transfer to CO_2 during the reduction process. The preferential CO_2 reduction over O_2 reduction is further confirmed by a more positive onset potential and higher cathodic current in linear sweep voltammetry (LSV) measurements (Supplementary Fig. 25).
22	408-409	XPS measurements of pre-protonated **HNNU-X** (X = O, S, Se) reveal a decreasing degree of anion protonation in the order **HNNU-Se** > **HNNU-S** > **HNNU-O** (Supplementary Fig. 32).
22	413-421	Operando XAFS was employed to elucidate proton transfer from in-situ generated $N(H^+)=C=X$ species to adsorbed CO_2 during photocatalysis over **HNNU-Se** (Figs. 7h and i). In the Se K-edge EXAFS spectra, three major peaks are observed at phase-uncorrected radial distances (r) of ~1.39, 1.93, and 2.46 Å, corresponding to the first-shell Se–C, second-shell Se–C–N, and third-shell Se–C–N–Zn scattering paths, respectively. Upon illumination under CO_2 for 40 min, the first-shell Se–C peak gradually weakens and broadens, consistent with protonation of $[SeCN]^-$ by photogenerated H^+ to form $N(H^+)=C=Se$ species. With prolonged irradiation (60 min), the Se–C peak intensity partially recovers, indicating deprotonation of $N(H^+)=C=Se$ due to proton transfer to adsorbed CO_2. These operando XAS results provide direct evidence that CO_2 photoreduction proceeds via participation of in-situ generated protons in the form of $N(H^+)=C=Se/H^+$.
25	479-480	The photocatalytic CO_2RR reaction was carried out in a homemade quartz glass reactor under natural sunlight irradiation in atmospheric air, as shown in Supplementary Fig. 16a.
25-26	483-492	The liquid products were quantified by NMR spectroscopy, in which dimethyl sulfoxide (DMSO, Macklin, 99.99%) was used as the internal standard. Aliquots at an interval of 1 h were drawn from the reaction mixture and analyzed by ^1H NMR. For ^1H NMR measurement, 0.5 mL of reaction solution was mixed with 0.1 mL of D_2O, and 10 μL of DMSO (equivalent to 140.8 μmol) was added as the internal standard. Detailed ^1H NMR spectra of liquid products were available in Supplementary Figs. 17-19. The area of product peaks was compared to the area of DMSO (at a chemical shift of 2.6 ppm). In our case, the double peak of acetaldehyde at a chemical shift of 1.22 ppm was used to calculate the generation rate (N) as the following equation: $$\mathrm{N} = \frac{S \times V2 \times 140.8 \times 6}{2 \times V1 \times t \times m}$$ where S is the area of double peak of acetaldehyde compared to DMSO (identified to 1 as reference) (0.007 for **HNNU-Se**, 0.003 for **HNNU-S**, 0.002 for **HNNU-O**), V2 is the volume of water (2.5 mL) used during

		photocatalysis, V1 is the volume of reaction solution tested (500 μL), t is reaction time (6 h) and m is the mass of catalyst (0.005 g).
26	497-501	The photocatalytic CO_2RR reaction was also carried out in a self-designed quartz reactor equipped with a xenon lamp as light source in pure CO_2 atmosphere, as shown in Supplementary Fig. 16b. **HNNU-X** powder (0.005 g) was uniformly dispersed in deionized water (2.5 mL). The suspension was stirred in pure CO_2 atmosphere. Xenon lamp (300 W) was used as light source to simulate sunlight. Condensate water was introduced to ensure the photoreduction at room temperature.
26	503-506	All the computations have been performed by the DFT within the def2-SV(P) basis and B3LYP functional using Gaussian 16 software. All calculations used the DFT-D3 method to describe weak interactions within the system. To account for the solvation effects, all geometry optimizations and frequency calculations were performed with the IEF-PCM model using water as solvent.
30	592-595	27 Torubaev, Y. V. *et al.* Heterovalent chalcogen bonding: supramolecular assembly driven by the occurrence of a tellurium(ii)...Ch(i) (Ch = S, Se, Te) linkage. *Inorg. Chem. Front.* **9**, 5635-5644 (2022).<br>28 Dong, T. *et al.* Noncovalent Se...O conformational locks for constructing high-performing optoelectronic conjugated polymers. *Adv. Mater.* **29**, 1606025 (2017).
31	616-633	39 Lei, J. et al. Visible Light-Driven Acetaldehyde Production from CO_2 and H_2O via Synergistic Vacancies and Atomically Dispersed Cu Sites. *Angew. Chem. Int. Ed.* **64**, e202422667 (2025).<br>40 Shown, I. et al. Carbon-doped SnS_2 nanostructure as a high-efficiency solar fuel catalyst under visible light. *Nat. Commun.* **9**, 169 (2018).<br>41 Gao, W. et al. Vacancy-defect modulated pathway of photoreduction of CO_2 on single atomically thin $AgInP_2S_6$ sheets into olefiant gas. *Nat. Commun.* **12**, 4747 (2021).<br>42 Yu, F., Jing, X., Wang, Y., Sun, M. & Duan, C. Hierarchically porous metal–organic framework/MoS_2 interface for selective photocatalytic conversion of CO_2 with H_2O into CH_3COOH. *Angew. Chem. Int. Ed.* **60**, 24849-24853 (2021).<br>43 Zhu, S. et al. Selective CO_2 photoreduction into C_2 product enabled by charge-polarized metal pair sites. *Nano Lett.* **21**, 2324-2331 (2021).<br>44 Das, R., Das, K., Ray, B., Vinod, C. P. & Peter, S. C. Green transformation of CO_2 to ethanol using water and sunlight by the combined effect of naturally abundant red phosphorus and Bi_2MoO_6. *Energy Environ. Sci.* **15**, 1967-1976 (2022).<br>45 Shi, H. et al. Atomically dispersed indium-copper dual-metal active sites promoting C−C coupling for CO_2 photoreduction to ethanol. *Angew. Chem. Int. Ed.* **61**, e202208904 (2022).<br>46 Ou, H. et al. Atomically dispersed Au-assisted C–C coupling on red

		phosphorus for CO_2 photoreduction to C_2H_6. *J. Am. Chem. Soc.* **144**, 22075-22082 (2022).
32	649-665	54 Hohenberg, P. & Kohn, W. Inhomogeneous electron gas. *PhRv* **136**, B864-B871 (1964).<br>55 Weigend, F. & Ahlrichs, R. Balanced basis sets of split valence, triple zeta valence and quadruple zeta valence quality for H to Rn: Design and assessment of accuracy. *Phys. Chem. Chem. Phys.* **7**, 3297-3305 (2005)<br>56 Stephens, P. J., Devlin, F. J., Chabalowski, C. F. & Frisch, M. J. Ab Initio calculation of vibrational absorption and circular dichroism spectra using density functional force fields. *J. Chem. Phys.* **98**, 11623-11627 (1994).<br>57 Gaussian 16 Rev. C.01 (Wallingford, CT, 2016).<br>58 Grimme, S., Antony, J., Ehrlich, S. & Krieg, H. A consistent and accurate ab initio parametrization of density functional dispersion correction (DFT-D) for the 94 elements H-Pu. *J. Chem. Phys.* **132** (2010).<br>59 Cancès, E., Mennucci, B. & Tomasi, J. A new integral equation formalism for the polarizable continuum model: Theoretical background and applications to isotropic and anisotropic dielectrics. *J. Chem. Phys.* **107**, 3032-3041 (1997).<br>60 Miertuš, S., Scrocco, E. & Tomasi, J. Electrostatic interaction of a solute with a continuum. A direct utilizaion of AB initio molecular potentials for the prevision of solvent effects. *Chem. Phys.* **55**, 117-129 (1981).<br>61 Cossi, M., Barone, V., Cammi, R. & Tomasi, J. Ab initio study of solvated molecules: a new implementation of the polarizable continuum model. *Chem. Phys. Lett.* **255**, 327-335 (1996).<br>62 Zhu, W. *et al.* Activating *CO by strengthening Fe–CO π-backbonding to enhance two-carbon products formation toward CO_2 electroreduction on Fe–N_4 sites. *Adv. Funct. Mater.* **34**, 2402537 (2024).
page	line	Highlight changes in the revised Supporting Information
3-5	75-132	**Electrochemical measurements.** Photoelectrochemical measurements were performed on an electrochemical workstation (CHI650E, Shanghai) with a standard three-electrode cell setup. A platinum plate and an Ag/AgCl electrode were used as the counter electrode and reference electrode, respectively. The working electrode was prepared by coating the test samples on a 1 cm × 1 cm indium-tin oxide (ITO) glass substrate via a typical slurry method (2.5 mg of samples were dispersed in a mixture consisting of 700 μL H_2O, 200 μL acetonitrile and 100 μL 5% Nafion by ultrasonication for 4 h). A mixture aqueous solution ($K_4Fe(CN)_6$, 0.5 mM, Na_2SO_4, 0.5 M) was used as the electrolyte solution. A Xe lamp (300 W,

Perfectling) was used as the light source. Mott–Schottky plots were collected in the dark with a perturbation signal of 5 mV at different frequencies (500, 750 and 1000 Hz). EIS results were recorded over the frequency range from 0.05 Hz to 100 Hz with an amplitude of 5 mV at the same bias voltages. Linear sweep voltammetry measurements were carried out in 0.5 M Na_2SO_4 aqueous solution with a scan rate of 5 $mV \cdot s^{-1}$ using Ag/AgCl electrode and Pt electrode as the reference electrode and counter electrode, respectively.

Femtosecond transient absorption spectroscopy (fs-TA). Femtosecond (fs) transient absorption spectroscopy measurements were investigated by a Helios ultrafast Yb:KGW laser (Light Conversion) with a regenerative amplifier, which produces IR pulses (10 W) centered at 1030 nm wavelength (200 kHz, pulse width < 290 fs). A portion of this beam (80%) was sent through an optical parametric amplifier (OPA, ORPHEUS, Light Conversion) to generate the pump beam of 320 nm for the excitation (pulse duration ~70 fs), and the residual was used as the probe beam. The resulting pump and probe pulses were directed into a TA spectrometer (Harpia, Light Conversion). Both pump and probe pulses were sent into an optical bench. The probe pulse was focused into a sapphire crystal, generating a continuum white light as the probe light. The frequency of pump pulse was reduced to 95 Hz, using a chopper, and the absorption with and without pumping light was measured and compared. For TA measurement for 100 ps, a time delay of the probe pulse with respect to the pump pulse was regulated by a computer-controlled optical delay stage. Pump and probe pulses were focused and overlapped on the sample plane, and the sample is placed on a glass carrier. Transmitting white-light probe pulses were directed into a photodiode array detector (Kymera, Andor). The resulting absorption differences were calibrated and analyzed using software (Harpia service app, Light Conversion).

In-situ diffuse reflectance infrared Fourier transform spectroscopy (DRIFTS). In-situ DRIFTS of the photocatalytic process over **HNNU-X** was performed on an iS50 FTIR spectrometer (Thermo Fisher, USA) by equipment of Praying Mantis diffuse reflectance accessory (Harrick) and specimen chamber (two ZnSe windows, one SiO_2 window). All spectra were recorded at a resolution of 4 cm^{-1} with an average of 128 scans. CO_2 gas at a flow rate of 20 mL/min was bubbled through deionized water and then passed through a sample cell loaded with **HNNU-X** powder. Visible light irradiation was applied to the powder through the quartz window of the cell. The gaseous mixture of CO_2 and H_2O vapor was stabilized prior to light illumination.

In-situ Electron paramagnetic resonance (EPR). EPR spectra were measured by a Bruker-BioSpin E500 spectrometer at room temperature. To evaluate the capability of **HNNU-Se** to reduce O_2 to superoxide ($\bullet O_2^-$), in-situ electron paramagnetic resonance (EPR) spectroscopy was employed

		using 5,5-dimethyl-1-pyrroline N-oxide (DMPO) as the spin-trapping agent. The measurements were conducted in acetonitrile under a pure O_2 atmosphere with triethanolamine (TEOA) as a hole scavenger. Specifically, **HNNU-Se** (5 mg) was dispersed in acetonitrile (2 mL), followed by the addition of TEOA (0.5 mL). The suspension was bubbled with O_2 for 1 h prior to transfer into an O_2-filled quartz tube. A 300 W xenon lamp was used for irradiation during the in situ measurements.<br>**In-situ X-ray photoelectron spectrometer (XPS).** In-situ XPS measurement was performed at a Thermo Fisher Scientific ESCALAB 250Xi spectrometer (Waltham, MA, USA) equipped with 300 W Xe lamp (full arc) as light source. For sample preparation, approximately 10-15 mg of catalyst was pressed into a self-supporting wafer, mounted on a sample holder, and transferred into the introduction chamber. The entire introduction chamber was then evacuated for 30 min to remove surface-adsorbed species, after which the sample holder was transferred into the analysis chamber. After marking the sample position, a set of dark-state XPS data, including survey spectra and high-resolution spectra for O, N, S, Se and Zn elements, was collected at room temperature (30 °C). Subsequently, a 300 W Xenon lamp (equipped with optional cut-off filters for 420 nm) was positioned at the window of the analysis chamber. The sample was irradiated for 20 min while continuously acquiring XPS spectra to monitor the dynamic evolution of surface chemical states and binding energy shifts under light illumination.<br>**Operando XAFS.** All XAFS data were collected at the 1W1B XAFS beamline of the Beijing Synchrotron Radiation Facility (BSRF), National Center for Nanoscience and Technology (NCNST), Chinese Academy of Sciences, Beijing, China. The storage ring of BSRF operates at 2.5 GeV with a maximum current of 250 mA. The in-situ photocatalytic XAFS measurements were carried out in a custom-fabricated in-situ reaction cell, where the catalyst was dispersed in aqueous solution with a continuous CO_2 purging. Prior to light irradiation, a set of XAFS spectra was collected under dark conditions as the baseline. A 300 W xenon lamp was then applied to irradiate the reaction cell. The measurement was done from 0 to 60 min, and spectra were collected after an interval of 20 min. For the in-situ XAFS spectral data acquisition at Zn K-edge and Se K-edge, we calibrated the position of absorption edge (E_0) using Zn foil and Se foil standard samples, respectively. All spectra were collected in fluorescence mode during the same beam time to ensure data comparability.
7	164-168	*3.1 Single-crystal X-ray diffraction view and SEM images of **HNNU-X** (X= O, S)*

		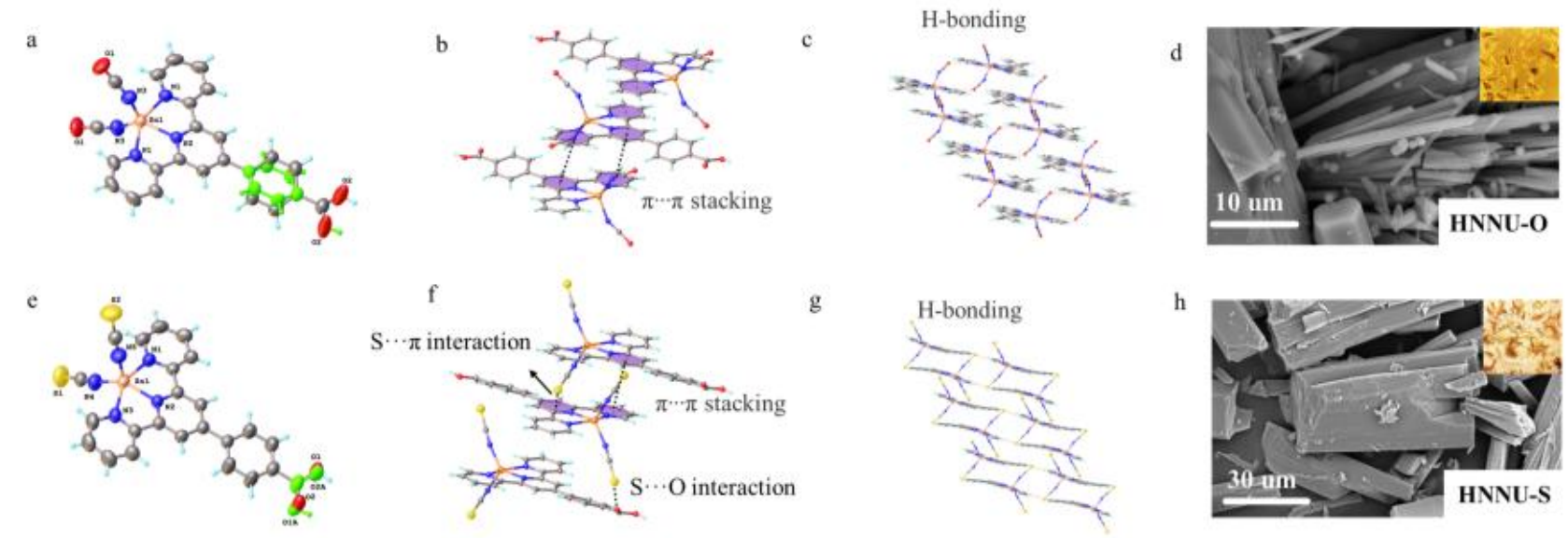<br><br>**Supplementary Fig. 2 |** Single-crystal X-ray diffraction view of the coordination mode of building blocks, the nodes and the topologies of **HNNU-O** (a-c) and **HNNU-S** (e-f) (gray C, cyan H, red O, blue N, yellow S, green Se, orange Zn). SEM images of **HNNU-O** (d) and **HNNU-S** (h) (insets are the corresponding single crystal images).
10	198-201	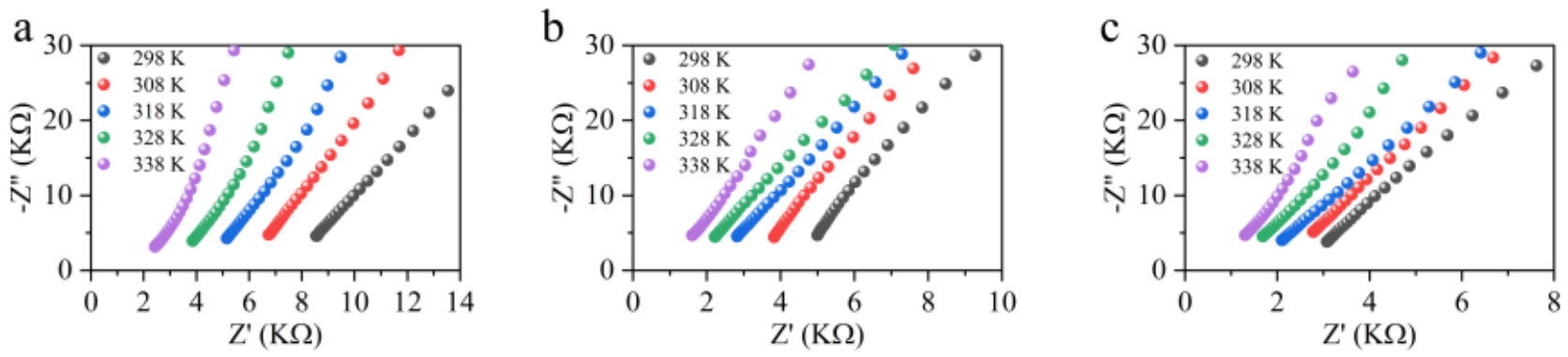<br><br>**Supplementary Fig. 8 |** Proton conductivity of **HNNU-O** (a), **HNNU-S** (b), and **HNNU-Se** (c) at different temperatures with 99% of relative humidity.
12	233-241	For Femtosecond transient absorption (fs-TA) spectroscopy, double-exponential fitting of the 602 nm decay profiles reveals two distinct relaxation pathways (Supplementary Figs. 12a-c). The fast component ($\tau_1 < 0.5$ ps) corresponds to exciton-exciton (hole and electron) annihilation, and the slower process (τ_2) may be attributed to the electron trapping, which often occurs in organic semiconductors, especially, in conjugated polymers. Additional ESA signals in the 750-800 nm range exhibit nanosecond-scale quenching dynamics (1.74-3.9 ns) (Supplementary Figs. 12d-f), which should correspond to the recombination of electrons and holes.<br>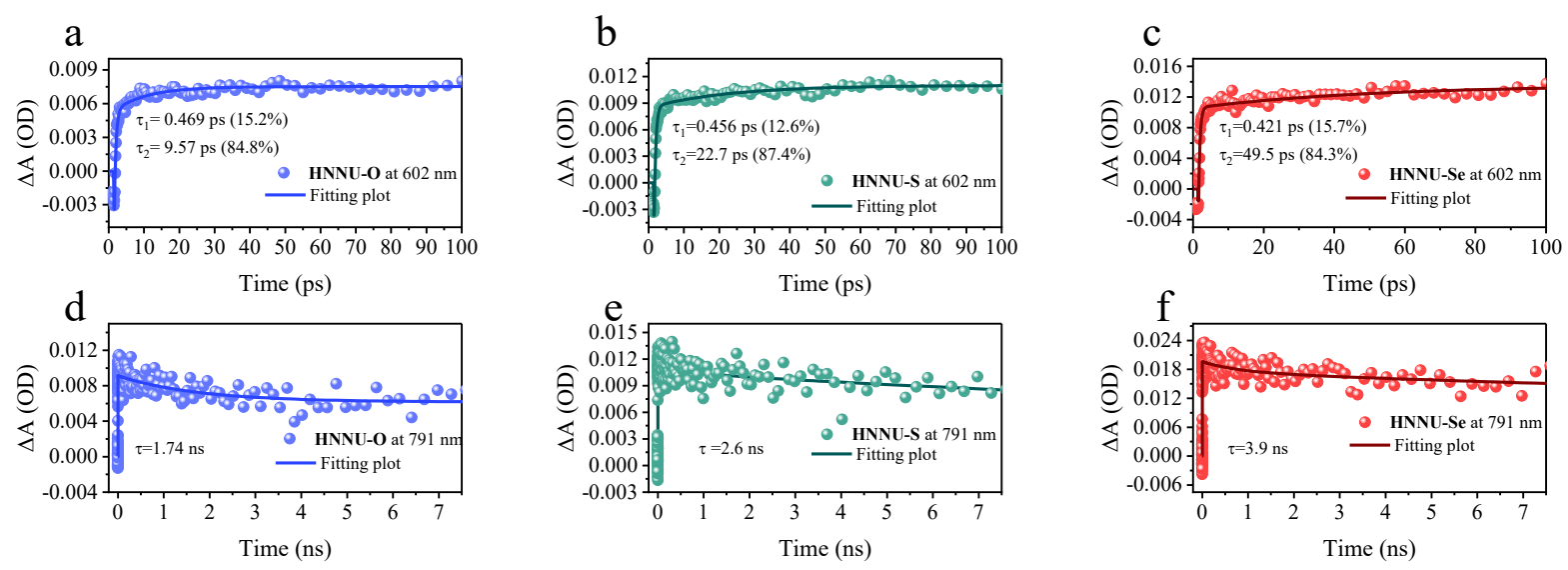<br><br>**Supplementary Fig. 12 |** Comparative kinetics decay curves of **HNNU-X** monitoring at 606 nm (a-c) and 791 nm (d-f) in the ESA region.
13-14	254-275	*In-situ* XPS measurements were performed to investigate the charge transfer in **HNNU-X** (X= O, S, Se). As shown in Supplementary Fig. 14, all **HNNU-X** exhibit two peaks in Zn 2p high-resolution spectra in dark, corresponding to Zn $2p_{3/2}$ (around 1044 eV) and Zn $2p_{1/2}$ (around 1021 eV) for Zn^{2+} state. Upon light excitation, the fitting peaks of Zn^{2+} shift to lower binding energy (Zn $2p_{3/2}$ from 1044.87 to 1044.50 eV for **HNNU-Se**, from

1044.79 to 1044.63 eV for **HNNU-S**, and from 1044.74 to 1044.66 eV for **HNNU-O**) (Supplementary Figs. 14a-c). The decrease in binding energy indicates the increase in electron density around Zn^{2+} center in **HNNU-X**.[3] Correspondingly, the binding energies of X (O, S, or Se) atoms in XCN^- anion shifts to higher binding energy, implying the decreased electron concentration of XCN^- anion (Supplementary Figs. 14d-f). The results provide convincing evidence for the transfer of photo-excited electrons from XCN^- anions to $[Zn(tpy)]^{2+}$ center in **HNNU-X**, which is consistent with the DFT calculation of frontier molecular orbitals (Figs. 4a-c). Notably, **HNNU-Se** shows the lowest binding energy of Zn 2p (1044.50 eV for Zn $2p_{3/2}$), followed by **HNNU-S** (1044.63 eV for Zn $2p_{3/2}$), and **HNNU-O** (1044.66 eV for Zn $2p_{3/2}$) under light irradiation (Supplementary Figs. 14a-c). It implies that electronic density around Zn^{2+} center follows the sequence of **HNNU-O** < **HNNU-S** < **HNNU-Se**. We reasonably assume that the higher electronic density should favor the C-end adsorption of CO_2 on Zn^{2+} sites to generate carbophilic center which is essential for C-C coupling.

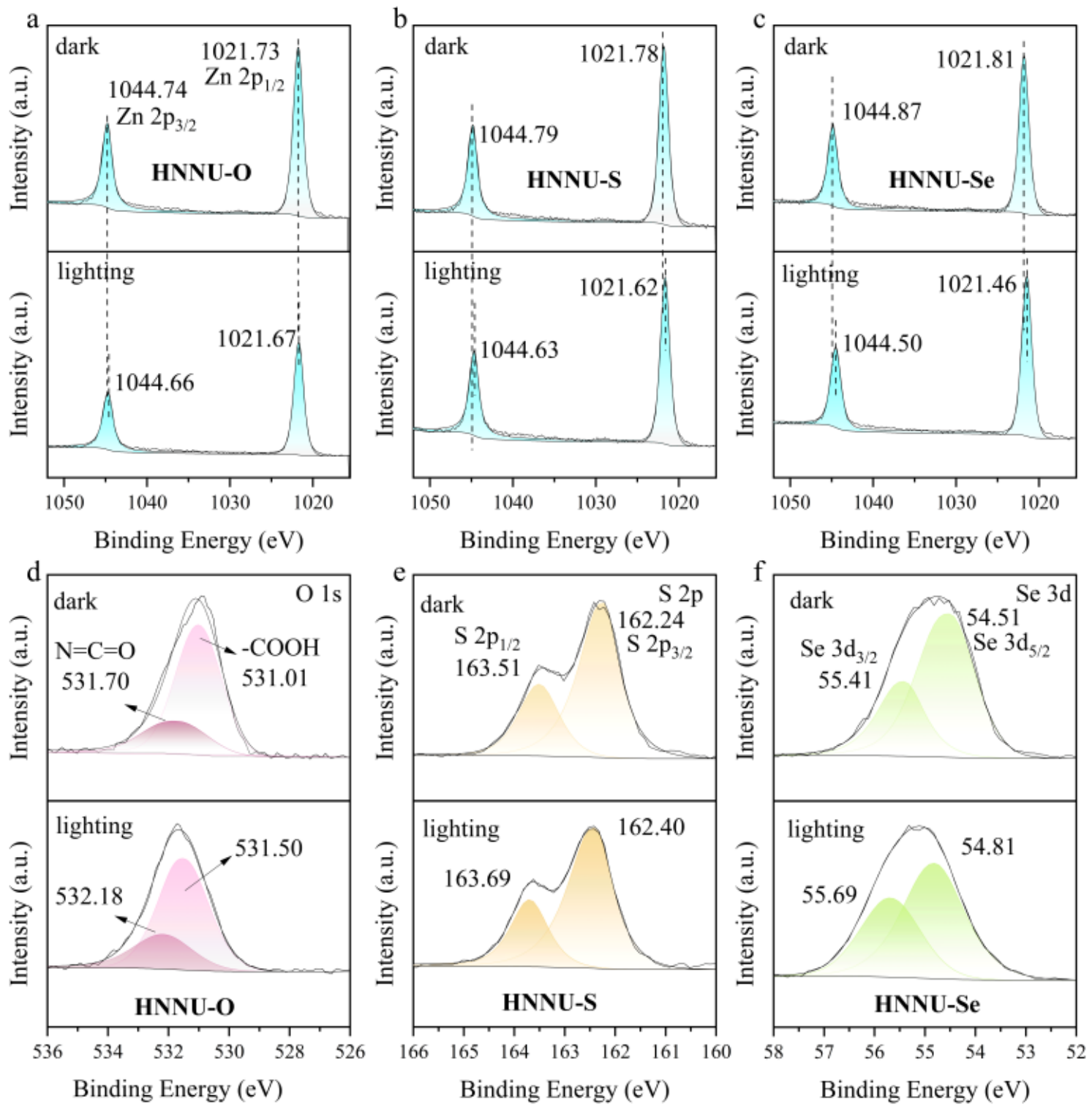

Supplementary Fig. 14 | *In-situ* XPS spectra of Zn 2p (a) and O 1s (d) over **HNNU-O**, Zn 2p (b) and S 2p (e) over **HNNU-S**, and Zn 2p (c) and Se 3d (f) over **HNNU-Se** before and after irradiation.

In-situ X-ray absorption fine structure (XAFS) was performed to investigate the charge transfer in **HNNU-X** (X= O, S, Se). The measurement setup is shown in Supplementary Figs. 15.

		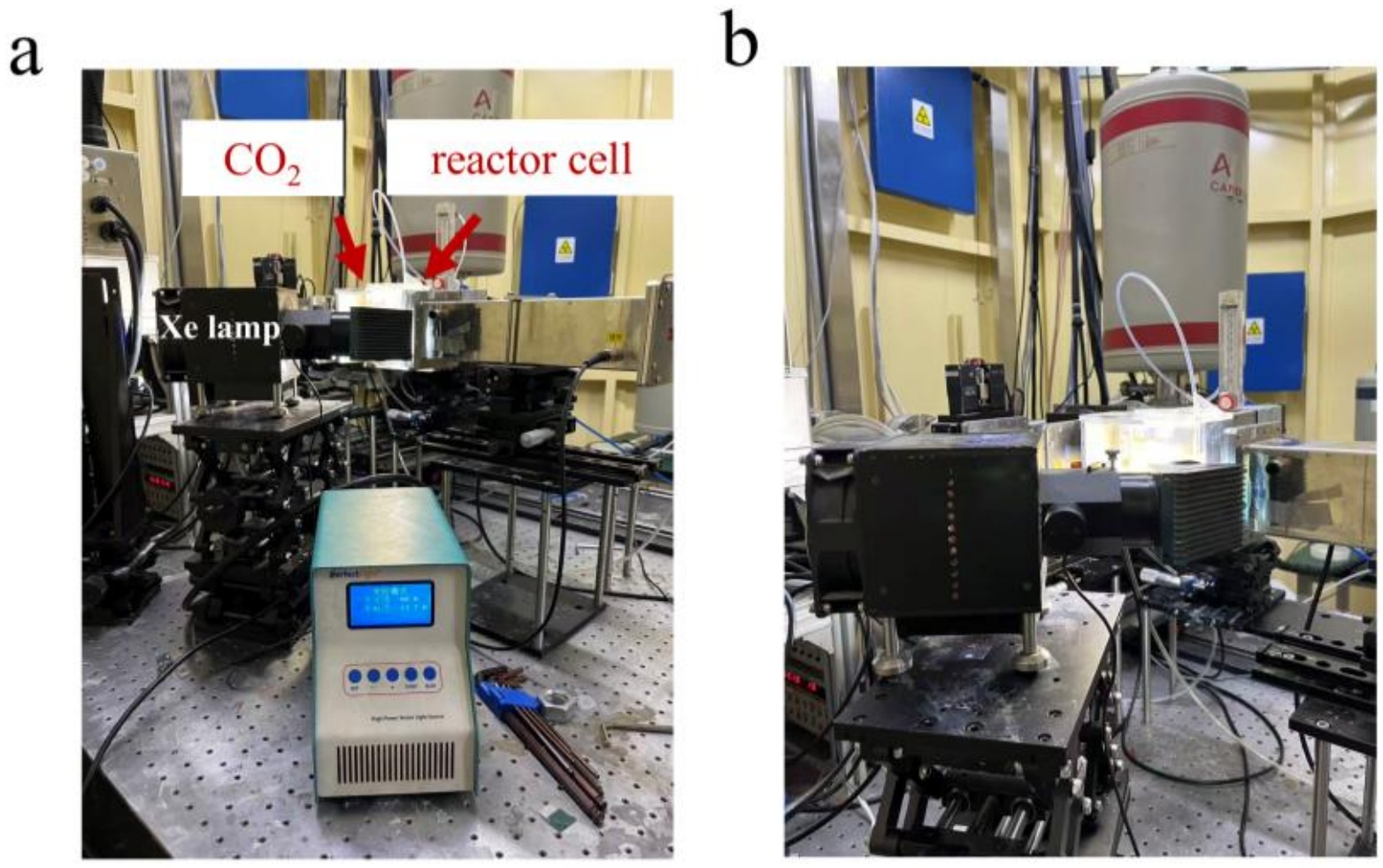<br><br>**Supplementary Fig. 15 |** Photograph of the in-situ XAFS measurement setup (a and b).
14-15	288-293	Furthermore, the photocatalytic CO_2RR reaction was carried out in a self-designed quartz reactor equipped with a xenon lamp as light source in pure CO_2 atmosphere, as shown in Supplementary Fig. 16b.<br>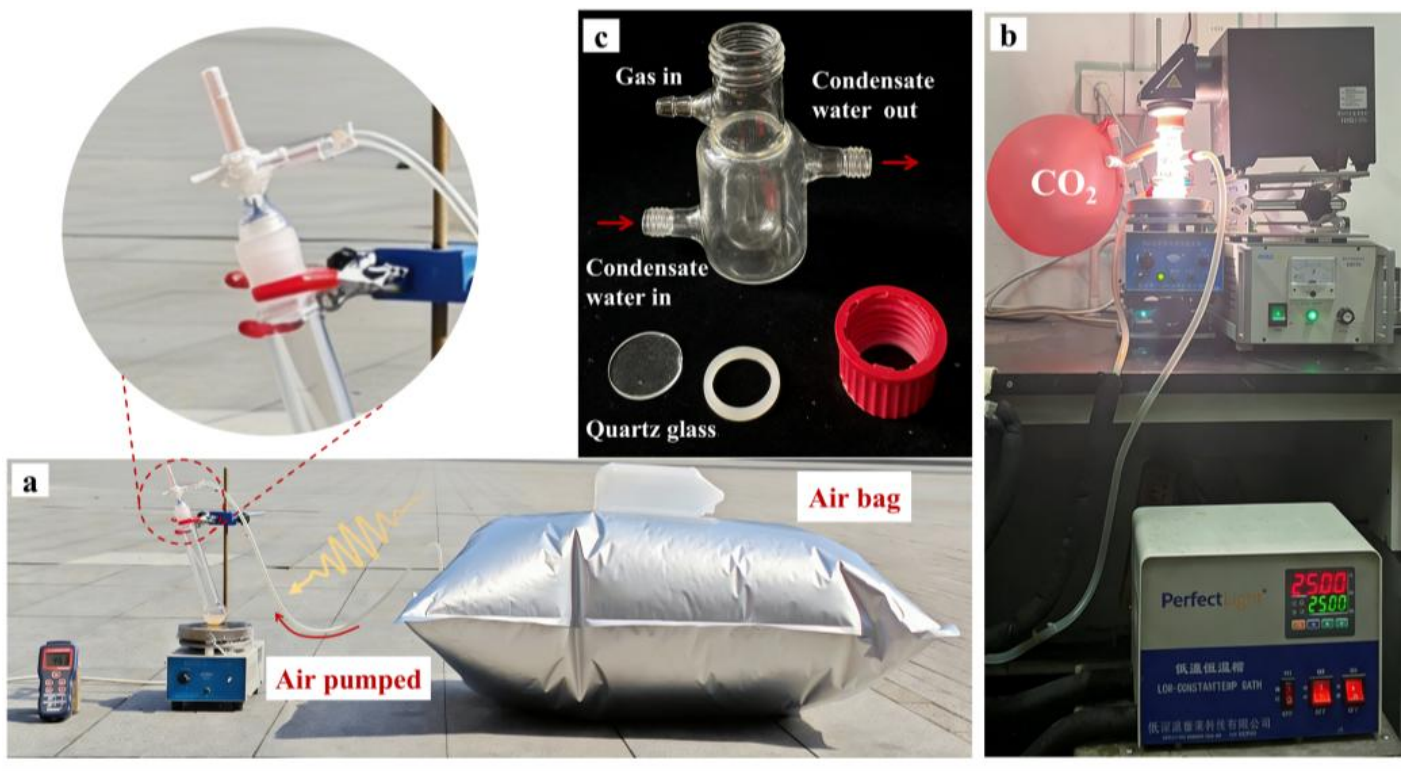<br><br>**Supplementary Fig. 16 |** Demonstration of CO_2 reduction under natural sunlight in air atmosphere and the structure of photoreactor (a). Demonstration of CO_2 reduction under xenon lamp in CO_2 atmosphere (b) and the structure of photoreactor (c).
16-18	303-327	To further confirm the formation of CH_3CHO, we recorded ^1H NMR spectrum of the reaction solution over **HNNU-X** with a water suppression mode (Supplementary Figs. 17-19). Distinct proton signals at 1.22 and 9.57 ppm are observed, corresponding to the CH_3 and CHO protons of CH_3CHO, respectively.

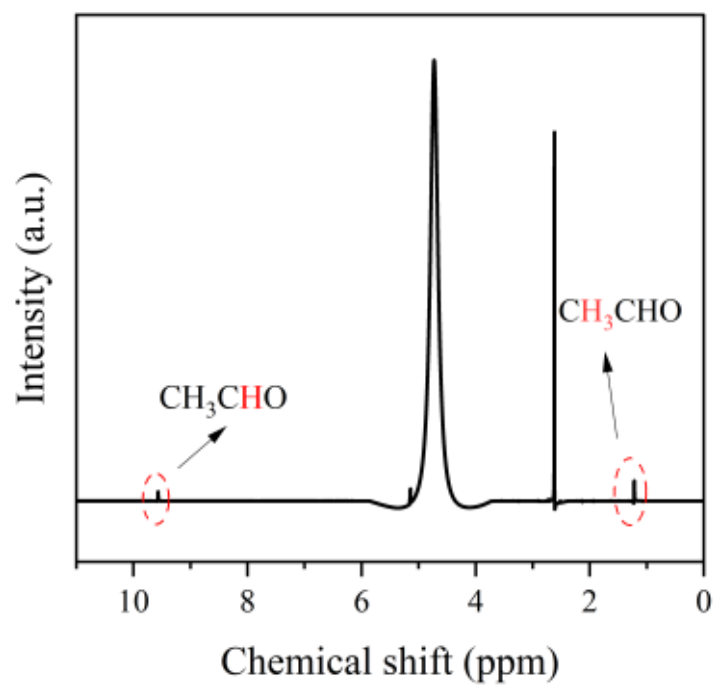

Supplementary Fig. 17 | ^{1}H NMR spectrum of the liquid products over **HNNU-O**.

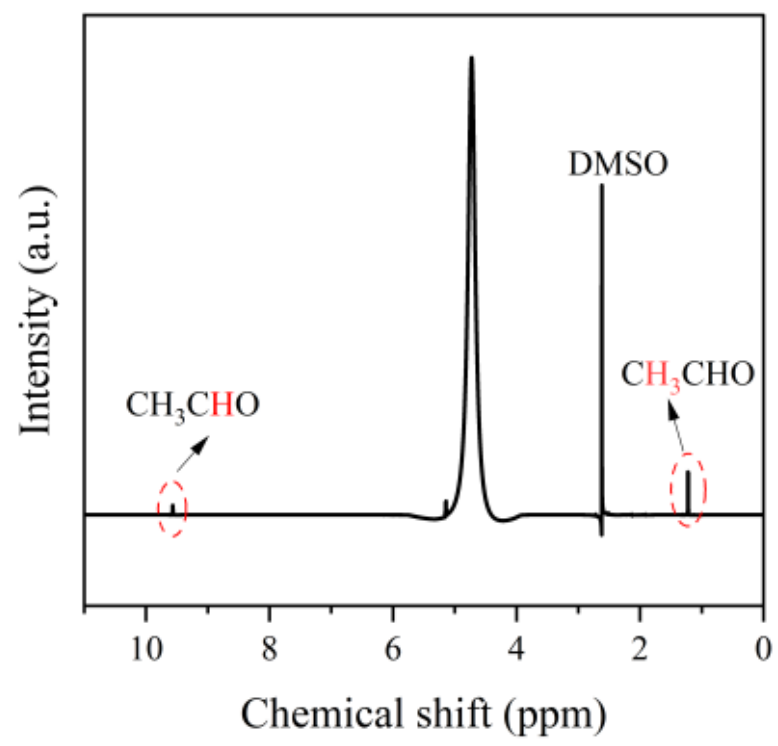

Supplementary Fig. 18 | ^{1}H NMR spectrum of the liquid products over **HNNU-S**.

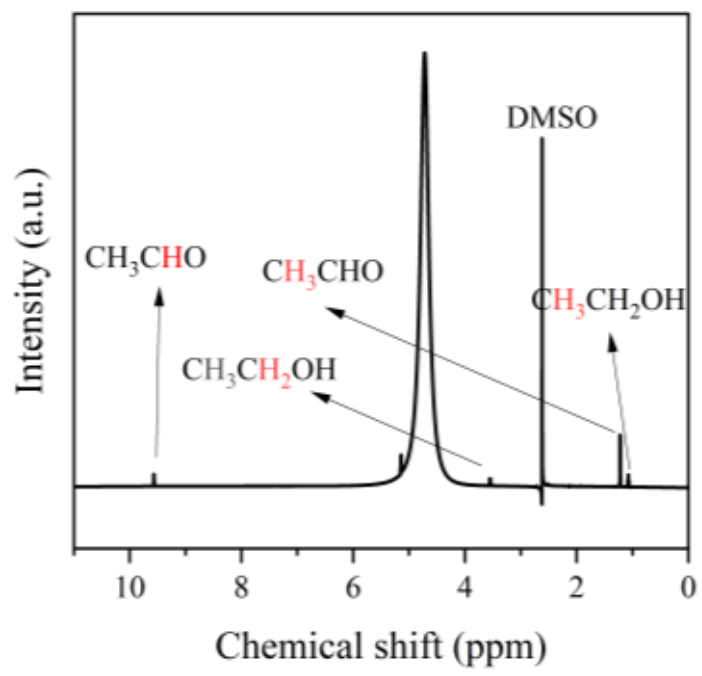

Supplementary Fig. 19 | ^{1}H NMR spectrum of the liquid products over **HNNU-Se**.

To verify that acetaldehyde originates from CO_2 photoreduction, isotopic labeling experiments were performed using $^{13}CO_2$ instead of air over **HNNU-Se**. The labelled products were determined by GC-MS analysis. When $^{13}CO_2$ was used as carbon source, the dominant GC peak at 3.03 min is assigned to ^{13}C-labelled acetaldehyde (Supplementary Fig. 20a). The dominant signal appears at m/z = 46, consistent with the molecular weight of $^{13}CH_3{}^{13}CHO$ (Supplementary Fig. 20b). ^{13}C NMR spectrum of the ^{13}C-labelled acetaldehyde shows characteristic isotope-induced splitting at ~19.4 and 98.9 ppm (Fig. 5h), attributable to the *$^{13}CH_3$* and *^{13}CHO* carbons

in $^{13}CH_3{}^{13}CHO$, respectively. It confirms that CO_2 is the carbon source of CH_3CHO. When D_2O was employed, the peak at m/z= 44 shifts to 48 (Supplementary Figs. 20c and d), corresponding to CD_3CDO. These results verified that water is the real proton source of such photocatalytic CO_2 conversion.

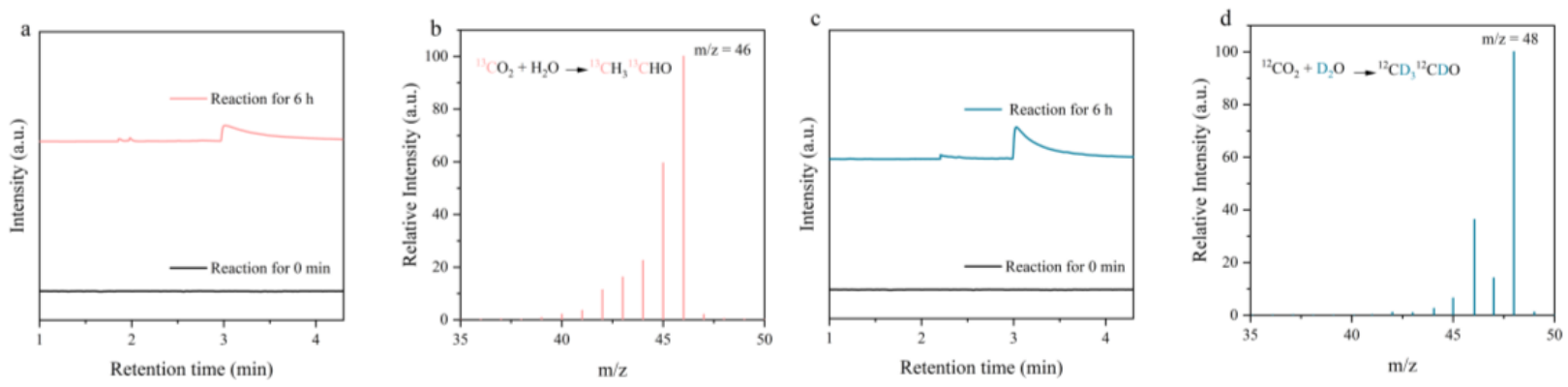

Supplementary Fig. 20 | GC (a) and corresponding MS spectrum (b) of produced acetaldehyde from $^{13}CO_2$ coupled with H_2O over **HNNU-Se**. GC (c) and corresponding MS spectrum (d) of produced acetaldehyde from $^{12}CO_2$ coupled with D_2O over **HNNU-Se.**

18-19 | 330-352

HNNU-Se outperforms the state-of-the-art photocatalysts reported for CO_2 reduction to CH_3CHO and the other C_2 products in the absence of sacrificial agents, as summarized in Supplementary Table 4.

Supplementary Table 4. Comparison of the state-of-the-art catalysts with **HNNU-Se** under CO_2 atmosphere without Sacrificial agent

st	Rate (μmol g^{-1} h^{-1})	Sel. (%)	AQE (%)	Main product	Gas
**Se** [a]	557.1	95.1	2.9 (450 nm)	CH_3CHO	air
**Se** [b]	721.2	93.0	N.A.	CH_3CHO	CO_2
A [c]	No detected	/	N.A.	CH_3CHO	CO_2
-Vs [d]	121.5	80	0.53 (400 nm)	CH_3CHO	CO_2
$_2$ [e]	139	N.A.	0.72 (420 nm)	CH_3CHO	CO_2
S_6 [f]	44.3	73	0.15 (420 nm)	C_2H_4	CO_2
D S_2 [g]	39.0	94	N.A.	CH_3COOH	CO_2
$_4$ [h]	2.95	92.5	N.A.	CH_3COOH	0.03% CO_2
RP [i]	51.8	<50	N.A.	C_2H_5OH	CO_2
CN [j]	28.5	92	N.A.	C_2H_5OH	CO_2
P [k]	1.32	96	N.A.	C_2H_6	CO_2

[a] **HNNU-Se** (5 mg), deionized water (2.5 mL), air atmosphere, natural sunlight, reaction temperature (25 °C) (This work).

[b] **HNNU-Se** (5 mg), deionized water (2.5 mL), CO_2 atmosphere, 300 W Xe lamp ($\lambda \geq 400$ nm), reaction temperature (25 °C) (This work).

[c] HCN-A (15 mg), acetonitrile (3.0 mL), deionized water (0.3 mL), CO_2 atmosphere, 5 W white LED lamp, reaction temperature (10 °C).[4]

[d] Cu/CZS-Vs (15 mg), acetonitrile (9.0 mL), deionized water (1.0 mL), CO_2 atmosphere, 300 W Xe lamp ($\lambda \geq 400$ nm; light intensity: 200 mW/cm^2.[5]

[e] C-SnS_2 (0.1 g), water vapour, CO_2 atmosphere, 300 W Halogen lamp, reaction temperature (25 °C).[6]

		[f] $AgInP_2S_6$ (5 mg), deionized water (0.4 mL), CO_2 atmosphere, 300 W Xe lamp.[7]<br>[g] d-UiO 66/MoS_2 (2 mg), water-vapor-saturated CO_2 gas (pressure reached 0.1 MPa), 300 W Xe lamp ((λ > 400 nm, 100 mW/cm^{-2}).[8]<br>[h] Co_3O_4 (2.5 mg), deionized water (5 mL), simulated air (0.03% CO_2, 79.98% N_2 and 19.99% O_2), 300 W Xe lamp (AM 1.5G filter, 100 mW/cm^{-2}), reaction temperature (25 °C).[9]<br>[i] BMO/RP (5 mg), deionized water (20 mL), CO_2 atmosphere, 450 W Xe lamp (with UV cut-off filter (KG-2 filter and CGA-400) filter), reaction temperature (25 °C).[10]<br>[j] InCu/PCN (50 mg), DMF/H_2O (24 mL, 12.5 vol% H_2O), CO_2 atmosphere (0.8 MPa), 300 W Xe lamp (1 W/cm^{-2}).[11]<br>[k] Au_1/RP (0.05 g), 0.5 g of $KHCO_3$ and 6 M HCl to produce high-purity CO_2 and water vapor atmosphere, 300 W Xe lamp (300 mW·cm^{-2}).[12]
20	364-368	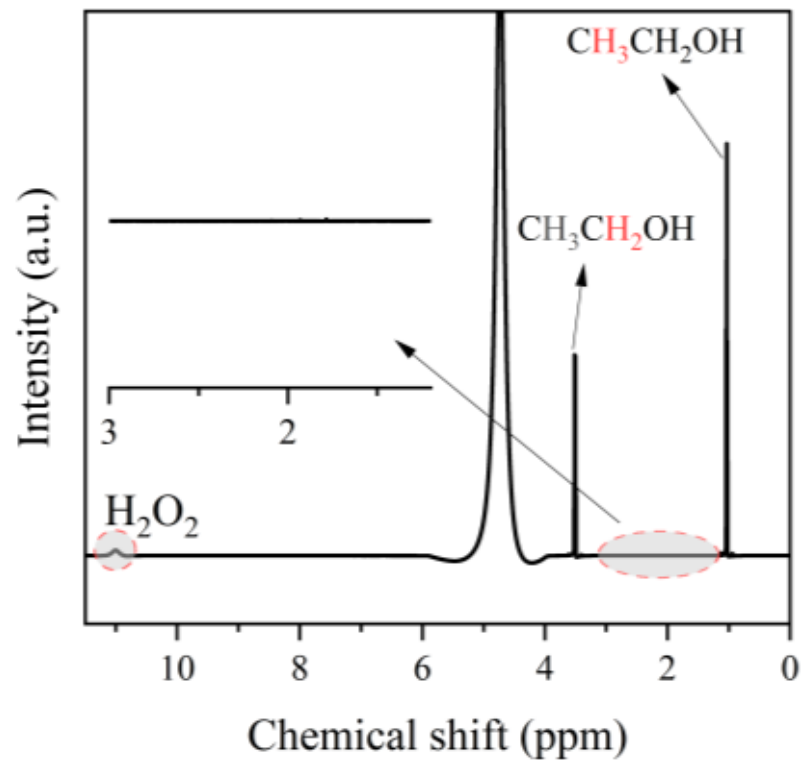<br>**Supplementary Fig. 21** | ^{1}H NMR spectrum of ethanol oxidation in aqueous H_2O_2 solution over **HNNU-Se**. Reaction condition: **HNNU-Se** (5 mg), H_2O_2 (1 M, 0.1 mL), ethanol (10 μL), CO_2 atmosphere, 300 W Xe lamp (full arc), reaction time (6 hours), reaction temperature (25 °C)
22	401-412	*Comparison the activity of CO_2 reduction and O_2 reduction.* To evaluate the competitive reduction of CO_2 and O_2, linear sweep voltammetry (LSV) measurements were conducted using an **HNNU-Se**-modified electrode in CO_2- or O_2-saturated Na_2SO_4 solution (1 M). As shown in Supplementary Fig. 25, the onset of cathodic current occurs at -1.33 and -1.47 V vs Ag/AgCl, KCl_{sat} for CO_2- or O_2-saturated electrolyte solution, respectively. It suggests that CO_2 reduction is easier to be triggered than O_2 reduction in the aqueous photocatalytic system. Moreover, only a slight increase in current density is observed in O_2-saturated solution at potentials more negative than -1.47 V, suggesting that O_2 reduction rarely occurs in the aqueous photocatalytic system. In contrast, the current density in CO_2-saturated solution increases markedly with more negative potentials. These observations demonstrate that CO_2 reduction predominates over O_2 reduction in the aqueous photocatalytic system, in agreement with the properties of **HNNU-X**.

		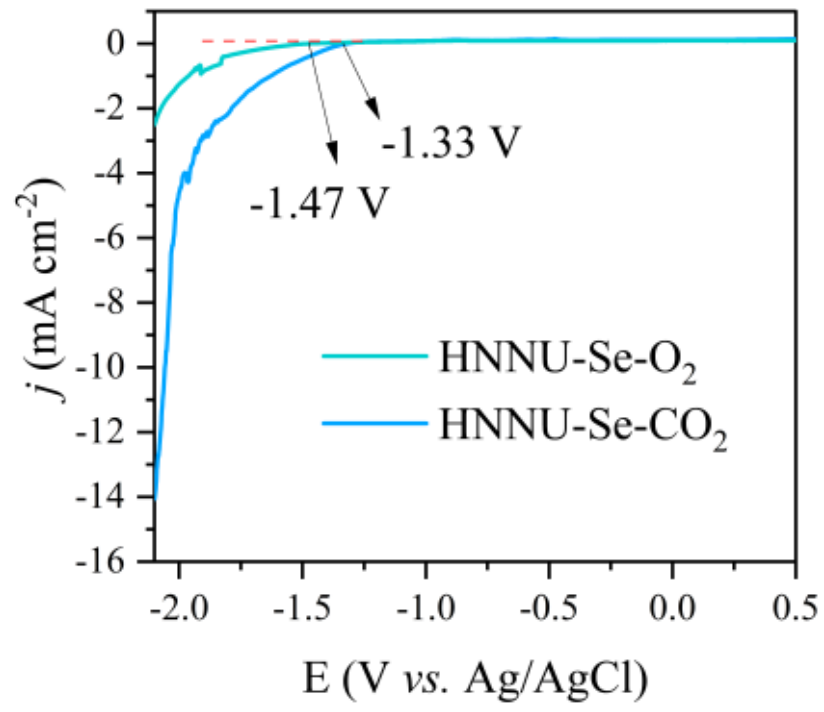<br>**Supplementary Fig. 25 |** LSV curves at **HNNU-Se**-modified electrodes (The tests were conducted in a CO_2- or O_2-saturated Na_2SO_4 aqueous solution (1 M) under visible light irradiation).
23	416-419	The supernatant was taken and placed in a 50 mL glass tube. The vial was immersed in a beaker filled with acetone, sealed, and allowed to undergo vapor diffusion at room temperature. The mixture stands at room temperature for 36 h to afford crystalline **HNNU-X**. XRD analysis shows that these **HNNU-X** materials can be self-healing through simple recrystallization (Supplementary Fig. 26).
26	467-476	X-ray photoelectron spectroscopy (XPS) measurements were conducted to further probe the protonation ability of **HNNU-X** (X = O, S, Se) (Supplementary Fig. 32). Prior to analysis, the samples were pre-protonated in dilute hydrochloric acid. The N 1s spectra can be deconvoluted into three components at ~398 eV, ~399 eV, and ~405 eV, assigned to N=C=X, pyridinic N, and protonated N(H^+)=C=X species, respectively. According to deconvolution of the peak areas, N(H^+)=C=X/N=C=X ratios are calculated to be 0.76, 0.29, and 0.16 and for **HNNU-Se** (Supplementary Fig. 32a), **HNNU-S** (Supplementary Fig. 32b), and **HNNU-O** (Supplementary Fig. 32c), respectively. These results indicate a progressively decreased protonation degree of [XCN]$^-$ anions in the order **HNNU-Se** > **HNNU-S** > **HNNU-O**.<br>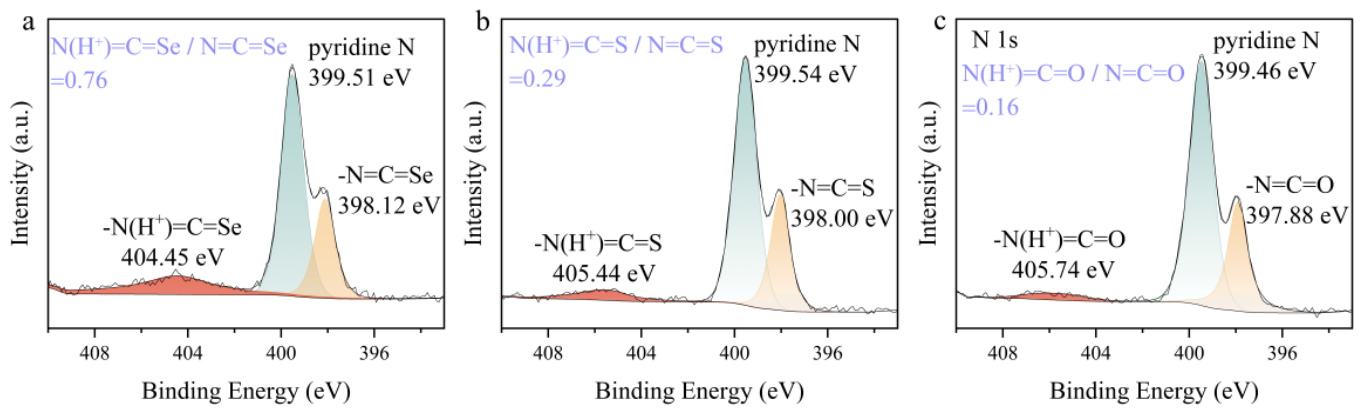<br>**Supplementary Fig. 32 |** N 1s XPS spectra of pre-acidified **HNNU-Se** (a), **HNNU-S** (b) and **HNNU-O** (c).
27-28	488-502	*5.2 EXAFS R-space fitting curves of **HNNU-Se** at different states*<br>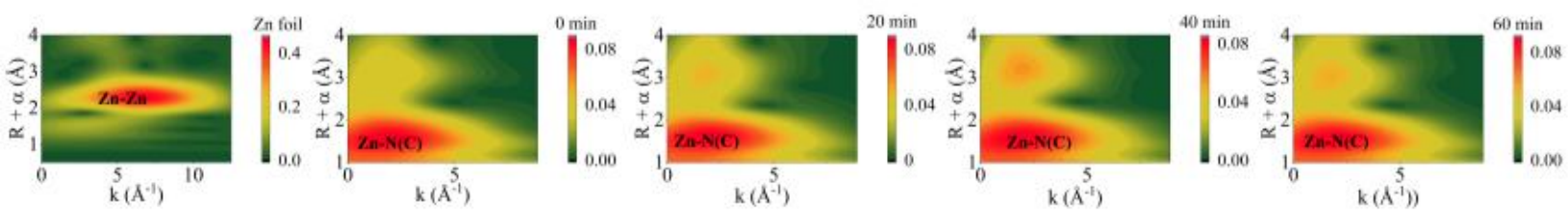<br>**Supplementary Fig. 35 |** WTs for the k^3-weighted Zn K-edge EXAFS signals under different states

<table>
<tr><td></td><td></td><td>

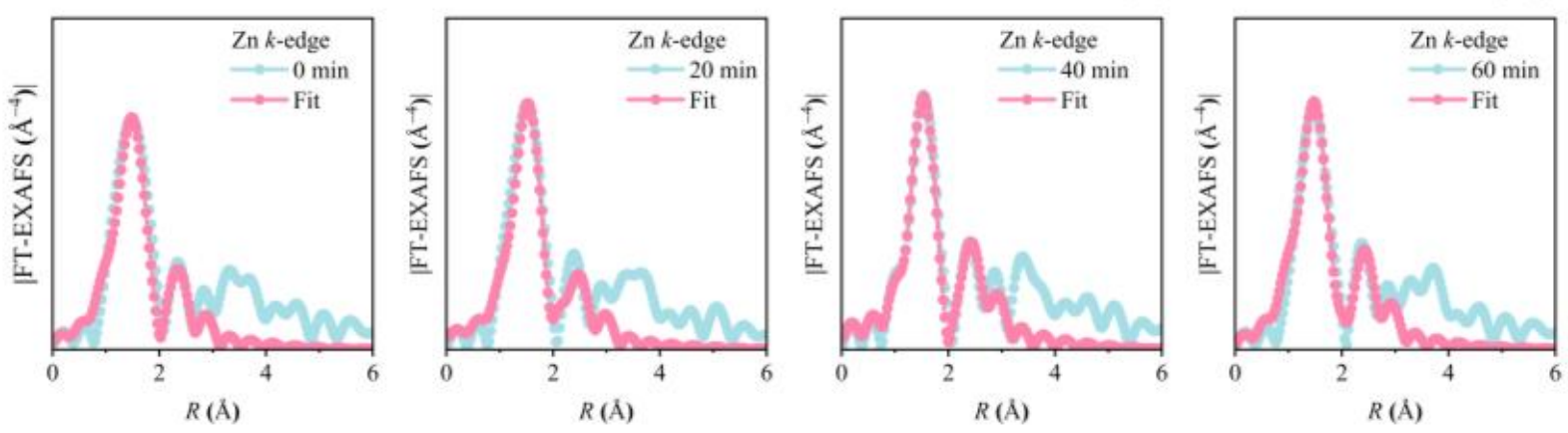

Supplementary Fig. 36 | Zn K-edge EXAFS R-space fitting curves of **HNNU-Se** at different states (The spectrum labeled “0 min” was obtained immediately after lighting, the spectra labeled “20 min”, “40 min” and “60 min” were collected 20, 40 and 60 min after illumination, respectively).

Supplementary Table 6. Fitted changes in coordination number (CN) of Zn-N shell at different states

<table>
<tr><th>Sample</th><th>Path</th><th>CN[a]</th><th>R (Å)[b]</th><th>σ^2 (Å^2)[c]</th><th>E_0 (eV)[d]</th><th>R factor[e]</th></tr>
<tr><td>0 min</td><td>Zn-N/C</td><td>5.2</td><td>2.02</td><td>0.008</td><td>0.5</td><td>0.019</td></tr>
<tr><td>20 min</td><td>Zn-N/C</td><td>5.6</td><td>2.08</td><td>0.005</td><td>2.00</td><td>0.019</td></tr>
<tr><td>40 min</td><td>Zn-N/C</td><td>5.9</td><td>2.09</td><td>0.008</td><td>1.06</td><td>0.009</td></tr>
<tr><td>60 min</td><td>Zn-N/C</td><td>5.4</td><td>2.02</td><td>0.010</td><td>3.5</td><td>0.020</td></tr>
</table>

[a] CN.: coordination number.
[b] R: the distance between absorber and backscatter atoms.
[c] $\sigma 2$: Debye-Waller factors.
[d] ΔE: the inner potential correction.
[e] R factor: goodness of fit.

</td></tr>
<tr><td>28-29</td><td>504-512</td><td>

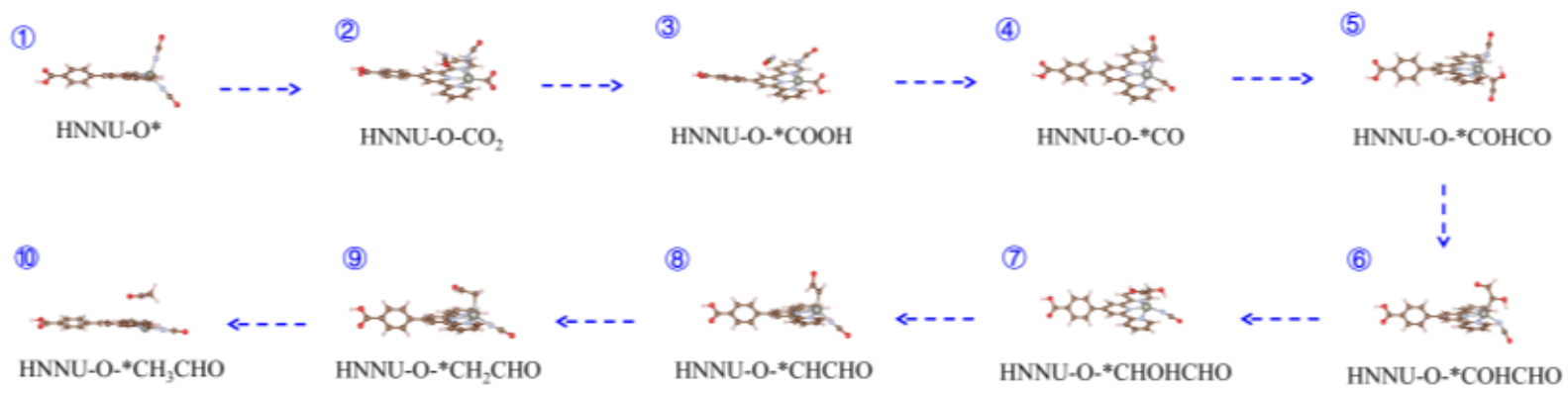

Supplementary Fig. 37 | DFT optimized structure models for every step of catalytic CO_2 reduction on **HNNU-O**.

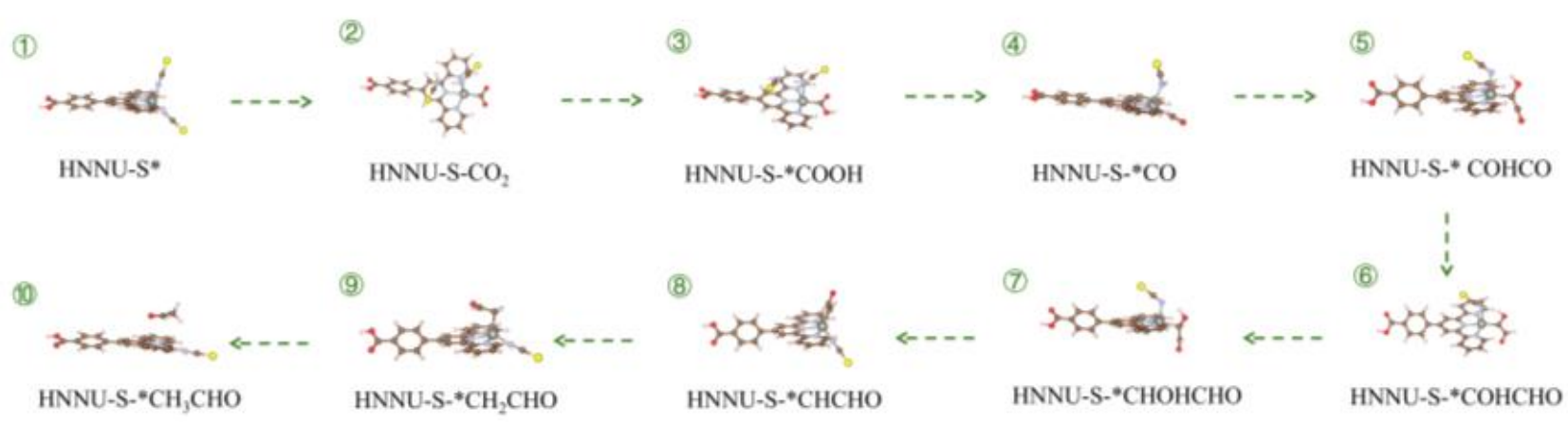

Supplementary Fig. 38 | DFT optimized structure models for every step of catalytic CO_2 reduction on **HNNU-S**.

</td></tr>
</table>

<table>
<tr><td></td><td></td><td>
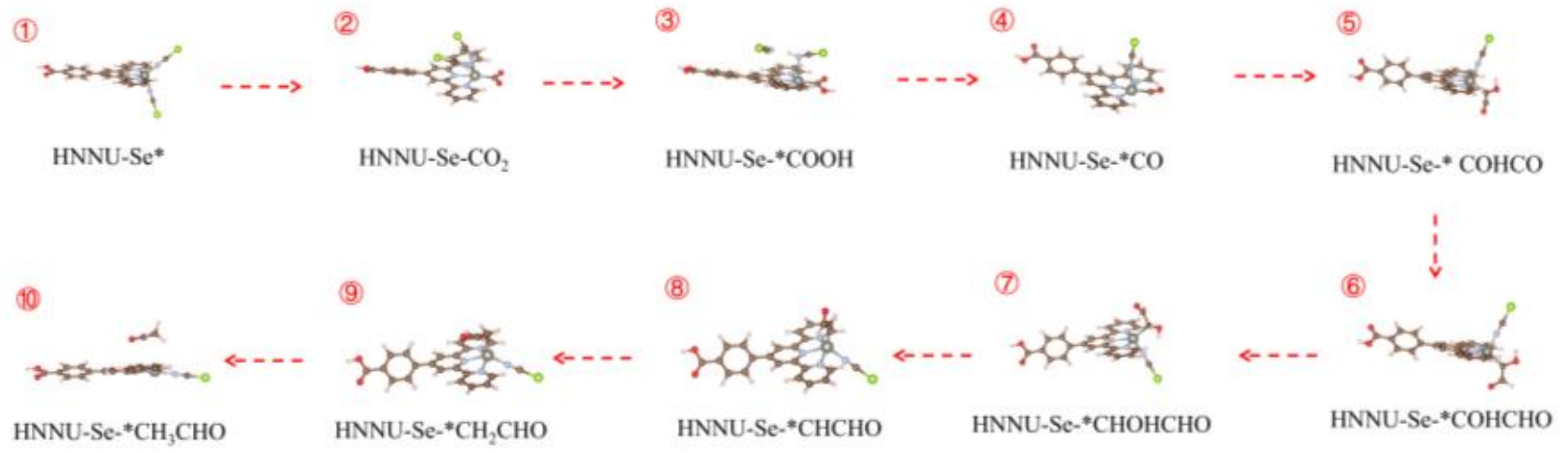

Supplementary Fig. 39 | DFT optimized structure models for every step of catalytic CO_2 reduction on HNNU-Se.
</td></tr>
<tr><td>29-31</td><td>514-525</td><td>The contents of Supplementary Tables 7-10</td></tr>
</table>

Response to the reviewers' comments

Dear reviewers,

Thanks for your good suggestions to revise our manuscript (NCOMMS-25-099726A). We have revised this manuscript carefully by point-by-point response to your comments, and listed the changes in detail made in the revised manuscript.

Reviewers #1:

1. The Reviewers **#1**: The authors have taken my previous report in a constructive manner and have performed the requested additional measurements to address my comments. I appreciate this constructive revision very much. However, the authors have to understand that direct reduction of CO_2 from air in 400 ppms concentration in the presence of a very large excess of far more reactive O_2 using solar light forming a extremely valuable C-C bond product with a photocatalyst that do not seem very stable in water since it is based on hydrogen bridges is like the most difficult photocatalytic process that deserves all the necessary controls before it can be published. I am satisfied with most of the responses except in the key product identification.

To start with, although the caption does not indicate the solvent, from the original manuscript it is supposed that the ^1H NMR spectrum of CH_3CHO shown in Figure 5 g is recorded in water. However, it is well known that acetaldehyde in H_2O is in equilibrium with the corresponding 1,1-ethanediol that is the prevalent species. Why Fig. 5 g does not show this species as the major one and shows exclusively the carbonylic form? Second, in the ^{13}C NMR spectrum of $^{13}CH_3{}^{13}CHO$ shown in Fig. 5 h and in more detail in the response, this diol form should also be observed, what is not the case.

Thanks for the expert's positive evaluation and valuable suggestions. The ^1H NMR spectrum of CH_3CHO shown in Figure 5g was recorded in water. Indeed, aldehyde undergoes reversible hydration in aqueous solution according to the following equilibrium:

$$RCHO + H_2O \leftrightharpoons RCH(OH)_2$$

In 2022, Ralf I. Kaiser *et al* synthesized methylene glycol ($CH_2(OH)_2$) for the first time via hydration of formaldehyde (HCHO) at extremely low temperature [Synthesis of methanediol [$CH_2(OH)_2$]: The simplest geminal diol, *Proc. Natl. Acad. Sci. U.S.A.*, 2022, 119, e2111938119]. However, hydration of acetaldehyde (CH_3CHO) is considerably less favorable because the electron-donating and steric effects of methyl group disfavor nucleophilic addition to the carbonyl carbon. Moreover, at room temperature, the resulting geminal diol is thermodynamically unstable, owing to the high electron density associated with the two hydroxyl groups. Only when the carbonyl carbon is connected to strong electron-withdrawing groups (such as -CHO, -CCl_3), the stability can be enhanced, for example, hydrated indoxyl, the hydrate of trichloroacetaldehyde, *etc*. Consequently, the hydration equilibrium is significantly shifted toward acetaldehyde at ambient condition in water with the equilibrium fraction of 1,1-ethanediol below 1% [Hydration of acetaldehyde. I. Equilibrium thermodynamic parameters., *J. Am. Chem. Soc.*, 1967, 89, 3524-3528]. As a result, the geminal diol species is not detectable in either ^1H NMR (Fig. 5g) or ^{13}C NMR spectrum (Fig. 5h) of the liquid product.

This observation is also consistent with previously reported photocatalytic systems for CO_2 reduction to acetaldehyde, in which the ^1H NMR spectra exhibit only acetaldehyde signals without any detectable geminal diol peaks (Fig. R1) [Visible light-driven acetaldehyde production from

CO_2 and H_2O via synergistic vacancies and atomically dispersed Cu sites, *Angew. Chem. Int. Ed.,* 2025, e202422667; Regulating the *OCCHO intermediate pathway towards highly selective photocatalytic CO_2 reduction to CH_3CHO over locally crystallized carbon nitride, *Energy Environ. Sci.*, 2022, 15, 225–233].

Published Data Figure Redacted

Fig. R1. (A) ^{1}H NMR spectra of photocatalytic CO_2 conversion with pure water over the Cu/CZS-Vs (*Angew. Chem. Int. Ed.,* 2025, e202422667); (B) ^{1}H NMR spectral of (a) 0.1mL of (10%) TEOA/MeCN TEOA/MeCN (triethanolamine/acetonitrile) solvent before reaction in 0.5 mL D_2O. (b) 0.1 mL of the reaction solvent mixed with 0.5 mL D_2O, the reaction solvent was collected from the employed reaction system after 1 hour visible light illumination by using the HCN-A catalyst and under the CO_2 atmosphere (*Energy Environ. Sci.*, 2022, 15, 225–233).

2. The Reviewers **#1**: Furthermore, in the expansion provided in the response (it should also be shown in the main text), the C atoms appear as doublet with different coupling constants. This is not possible, since the coupling should be exactly the same for the two C atoms in the system.

Thank you for this valuable suggestion. In ^{13}C NMR spectrum of liquid product derived from $^{13}CO_2$ photoreduction, the carbon resonances appear as doublet because of spin-spin coupling between two adjacent ^{13}C nuclei. In fact, the two carbon atoms exhibit identical coupling constants ($^1J_{C1\text{-}C2}$ = 45.8 Hz) (C_1: *C*HO, C_2: *C*H_3). The apparent discrepancy in the enlarged insets of the previous response arose from the different magnification (Fig. R2a). We apologize for this misunderstanding and have now replotted the spectra using the same magnification to enable a more direct comparison (Fig. R2b).

Similar spin-spin coupling splitting patterns in ^{13}C NMR spectra have been widely reported in isotope-labeling studies of CO_2 reduction (Fig. R3) [Asymmetric Bi and S single atoms over porous single-crystal TiO_2 for efficient CO_2 photoreduction to acetic acid, *Adv. Mater.*, 2026, 38, e17586; CO_2 reduction selective for C≥2 products on polycrystalline copper with N-Substituted pyridinium additives, *ACS Cent. Sci.*, 2017, 3, 853-859]. In particular, Agapie group emphasized that "$^1J_{C\text{-}C}$ splitting is observed, indicating that the products are essentially fully ^{13}C labeled" (Fig. R3b) [CO_2 Reduction Selective for C≥2 products on polycrystalline copper with N-Substituted pyridinium additives, *ACS Cent. Sci.*, 2017, 3, 853-859].

According to the expert's good suggestion, we have now included the ^{13}C NMR spectrum of ^{13}C-labeled CH_3CHO exhibiting the characteristic spin-coupling splitting pattern in the revised manuscript (see Fig. 5h in page 14).

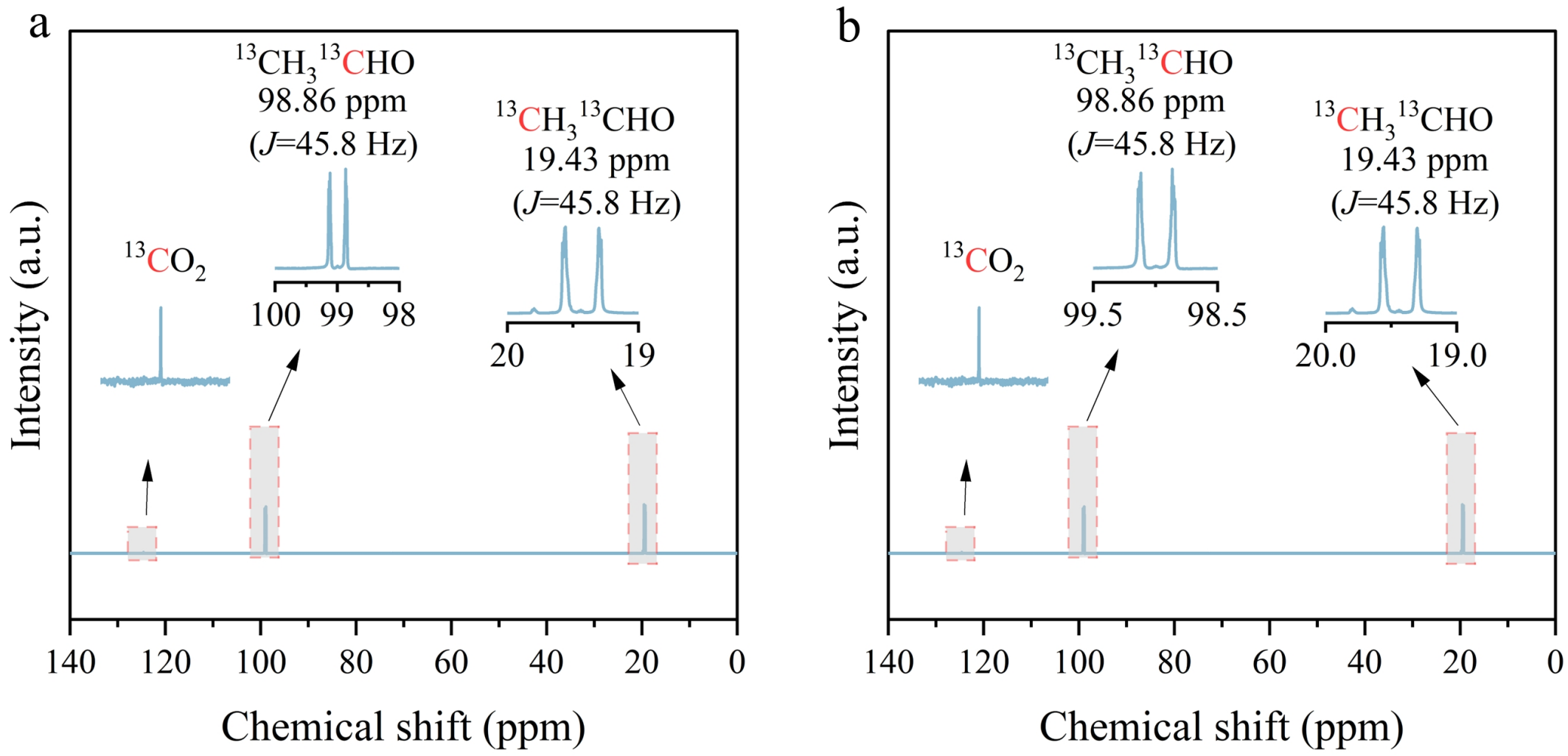

Fig. R2. ^{13}C NMR spectrum of ^{13}C labelled CH_3CHO from $^{13}CO_2$ photoreduction. (a) The enlarged ^{13}C peak of $^{13}CH_3{}^{13}CHO$ with different magnification. (b) The enlarged ^{13}C peak of $^{13}CH_3{}^{13}CHO$ with same magnification.

Published Data Figure Redacted

Fig. R3. (a) The photocatalytic ^{13}C-labeled CO_2 reduction to acetic acid of (Bi, S)TiO_2 under UV-vis light (*Adv. Mater.*, 2026, 38, e17586); (b) ^{13}C NMR spectrum (101 MHz, H_2O: D_2O = 9:1)

of catholyte after bulk electrolysis at -1.09 V vs RHE with 1 (10 mM, triangle) and ^{13}C-enriched 0.1 M of $KHCO_{3(aq)}$ and CO_2 (*ACS Cent. Sci.*, 2017, 3, 853-859).

Reviewers #3:

3. The Reviewers **#3**: Although the authors have made an effort to enhance the readability of the figures, the text in Scheme 1 remains difficult to read. For example, "high purity CO_2" in panel (a) is only visible upon significant zooming, and the label on the lamp is not legible. Similarly, the text in the cycles of panel (b) is too small, and the use of white text on a dark green background in the boxes at the bottom does not help much... The same applies to Figures 6f-g, where the assignment of bands is rather difficult to read.

Thank you for the expert's valuable suggestion. We have increased the font size of all text labels in Scheme and figures, and adjusted the text color and background contrast of the bottom boxes in Scheme 1b to improve clarity and readability (Figs. R4 and R5). These revisions have been incorporated into the revised manuscript (see Scheme 1 in page 5, and Fig. 6 in page 16).

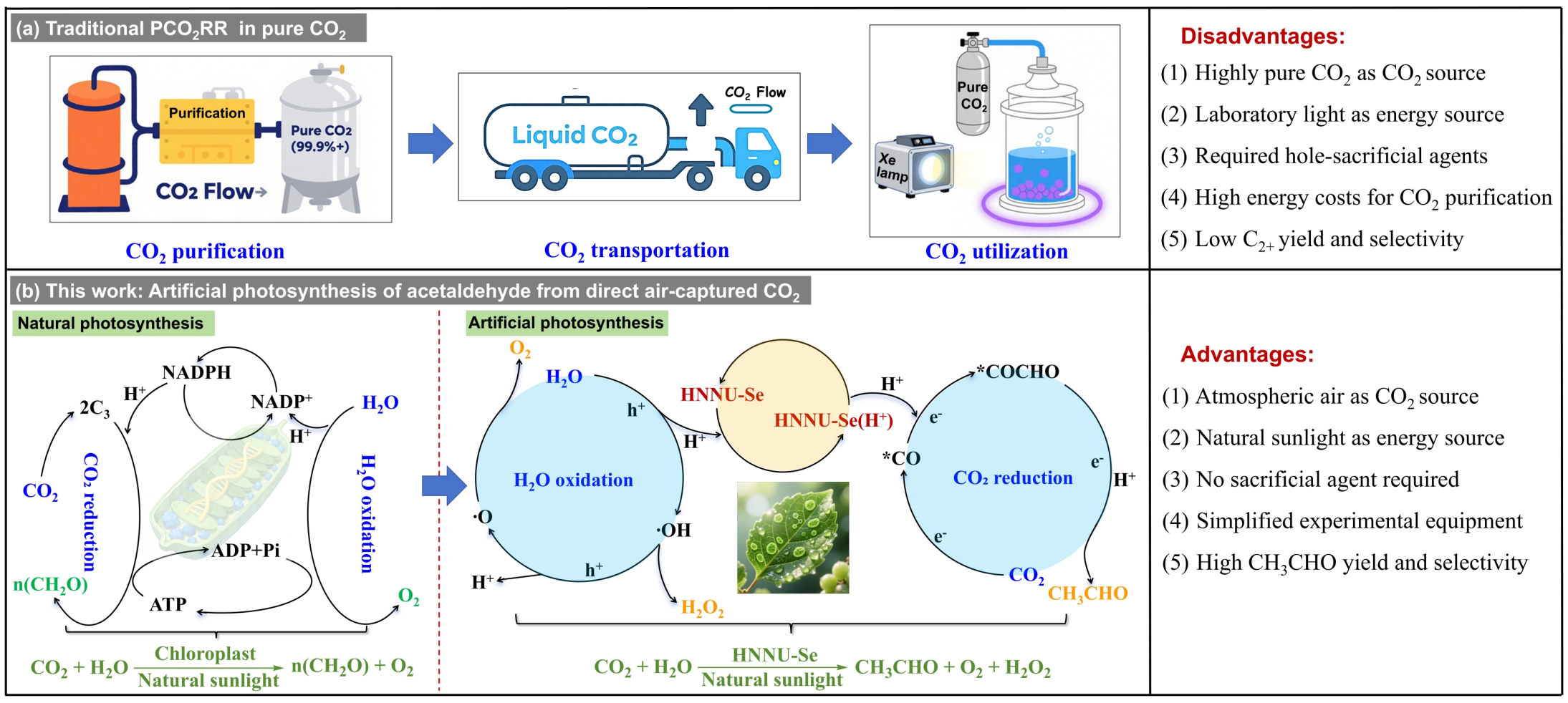

Fig. R4. a Schematic diagrams of traditional PCO_2R reaction. **b** Artificial photosynthesis of acetaldehyde from atmospheric CO_2 in this work.

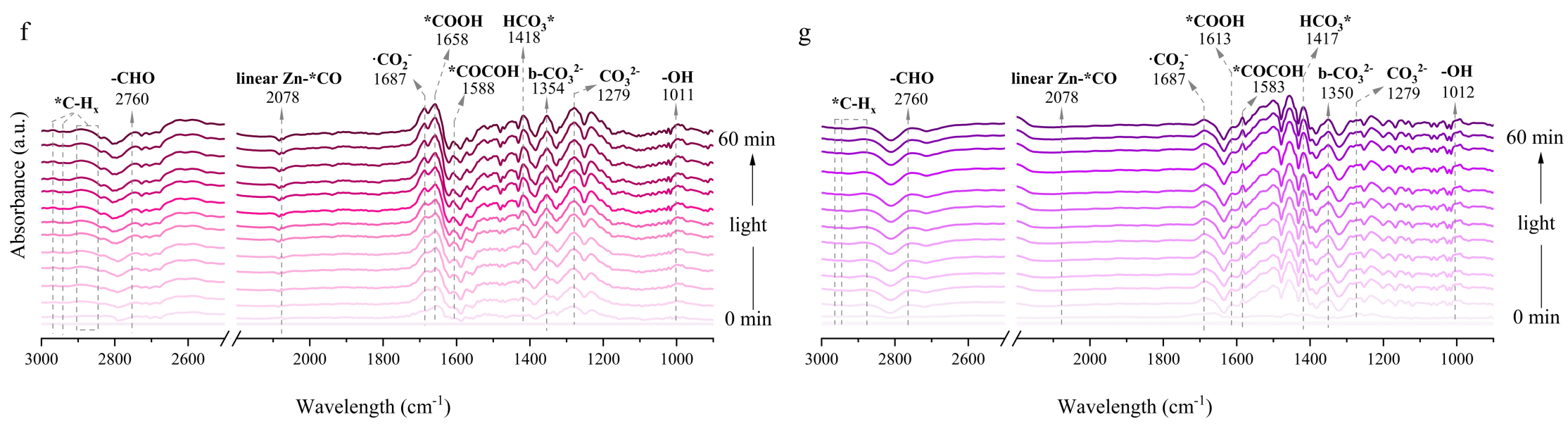

Fig. R5. f In-situ DRIFTS spectra for photocatalytic CO_2 conversion over **HNNU-Se**. **g** In-situ DRIFTS spectra for photocatalytic CO_2 conversion over **HNNU-O**.

4. The Reviewers **#3**: Regarding the revised DOS figures, they are much improved, and the HOMO-LUMO gaps can now be easily observed due to the reduced Gaussian smearing. However, there was no need of shifting the HOMO levels to 0 eV. If done, though, it should be explicitly stated in the caption, and the x-axis label should be changed from E (eV) to deltaE (eV) to clarify

that the energies are relative to the HOMO level rather than absolute values.

Thanks for the expert's valuable suggestion. We agree that shifting the HOMO levels to 0 eV was unnecessary. Accordingly, we have revised the relevant figures and adopted a DOS representation based on the absolute energy scale (Fig. R6), which provides a more standardized and clearer presentation of the results. All related revisions have been highlighted in the revised manuscript for ease of reference (see Fig. 4 in page 12).

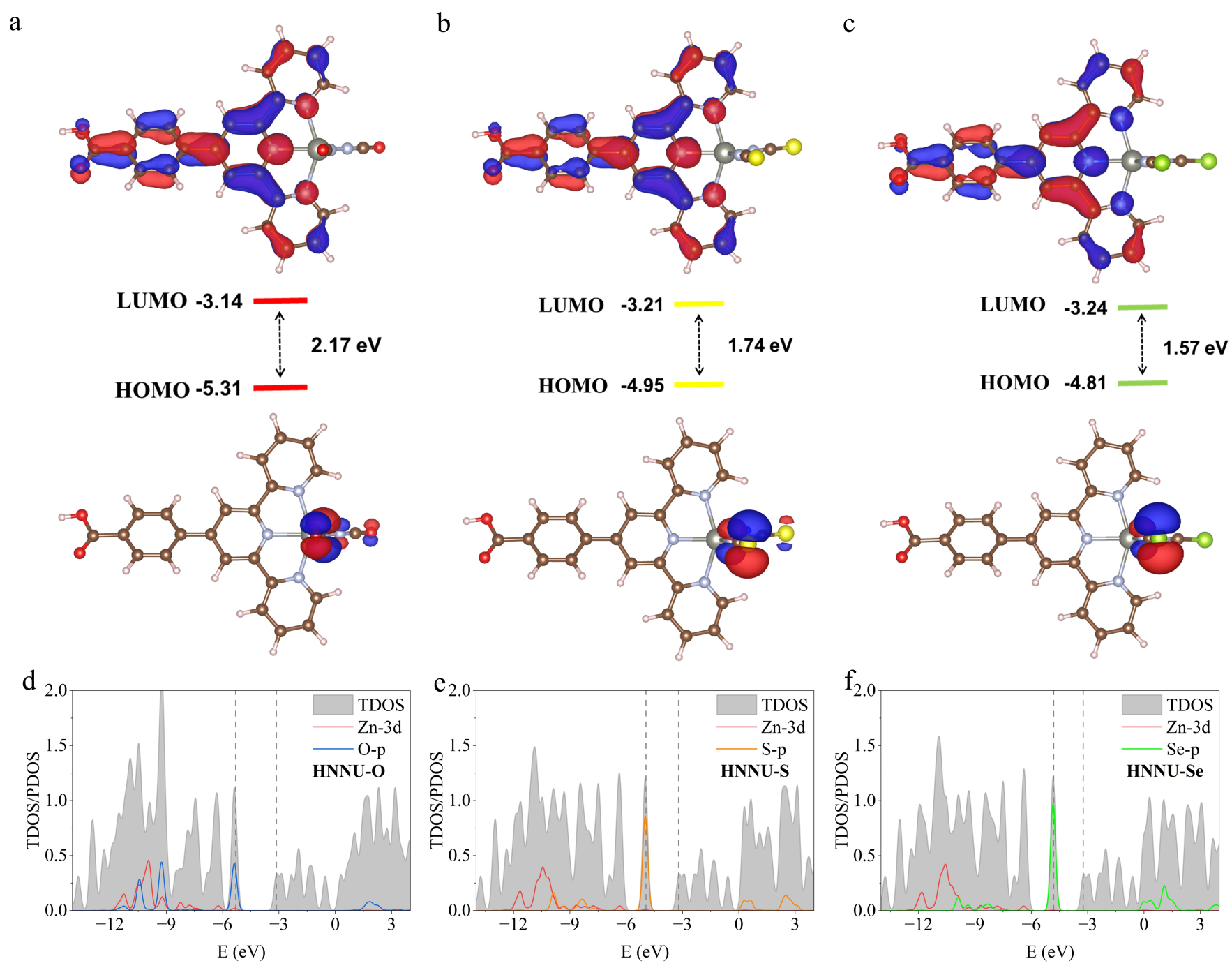

Fig. R6. a-c HOMO and LUMO distributions of **HNNU-X** (X= O, S, Se) (the positive and negative isosurfaces represented in red and blue, carbon, hydrogen, oxygen, nitrogen, sulfur, selenium and zinc atom are depicted in brown, beige, red, light blue, yellow, green, and gray, respectively). **d-f** Density of states of **HNNU-X** (X= O, S, Se).

5. The Reviewers **#3**: Finally, I strongly encourage the authors to include appropriate references in the computational details section for the methods, codes, algorithms, and approaches employed, as their developers deserve proper credit.

According to the expert's good suggestion, we have added appropriate references [refs. 54-64 in revised manuscript] for all methods, codes, algorithms, and approaches employed in the Computational Details section of the revised manuscript to ensure full academic credit is properly attributed to their original developers (see lines 324-330 in page 18, lines 652-671 in page 32).

A list of changes in revised manuscript No. NCOMMS-25-099726A

page	line	Highlight changes in the revised manuscript
5	85	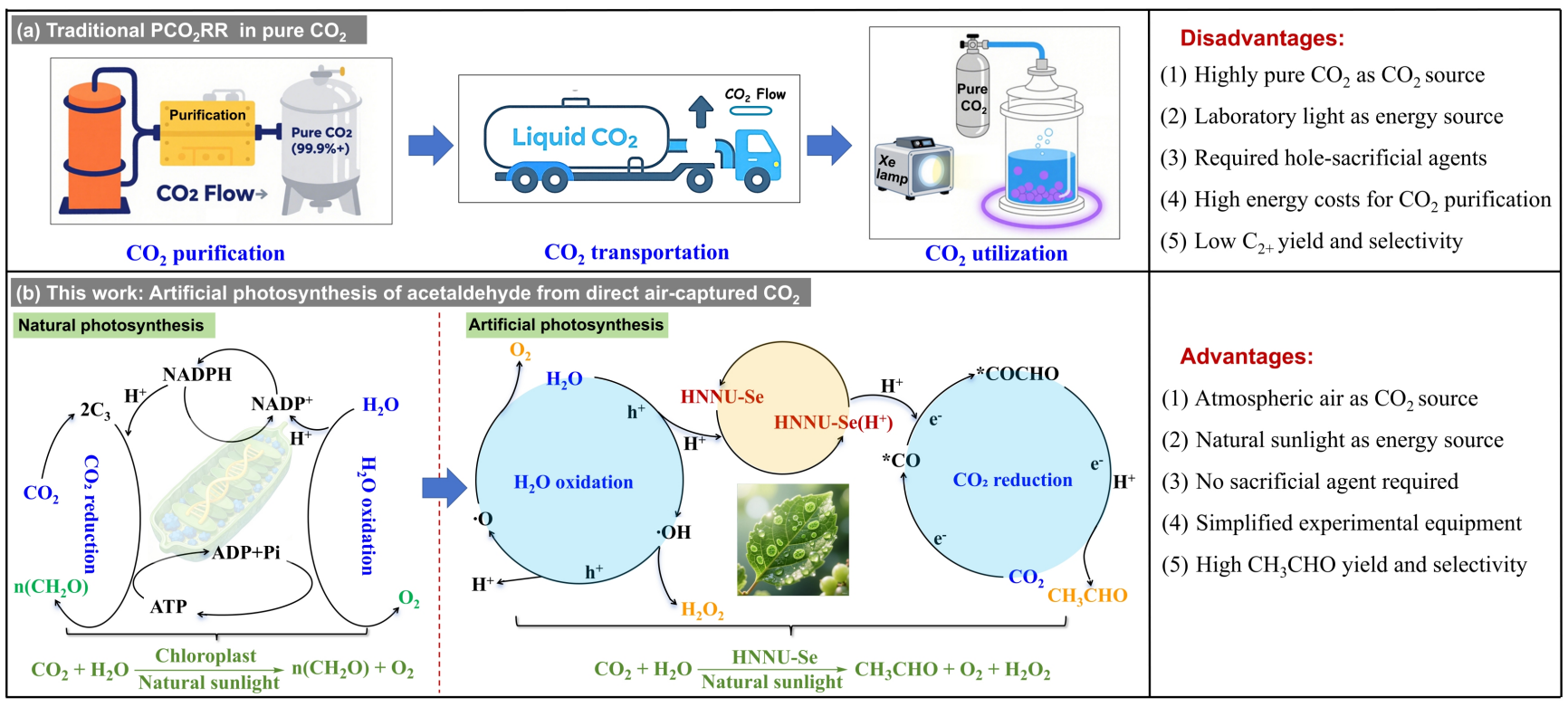
12	209	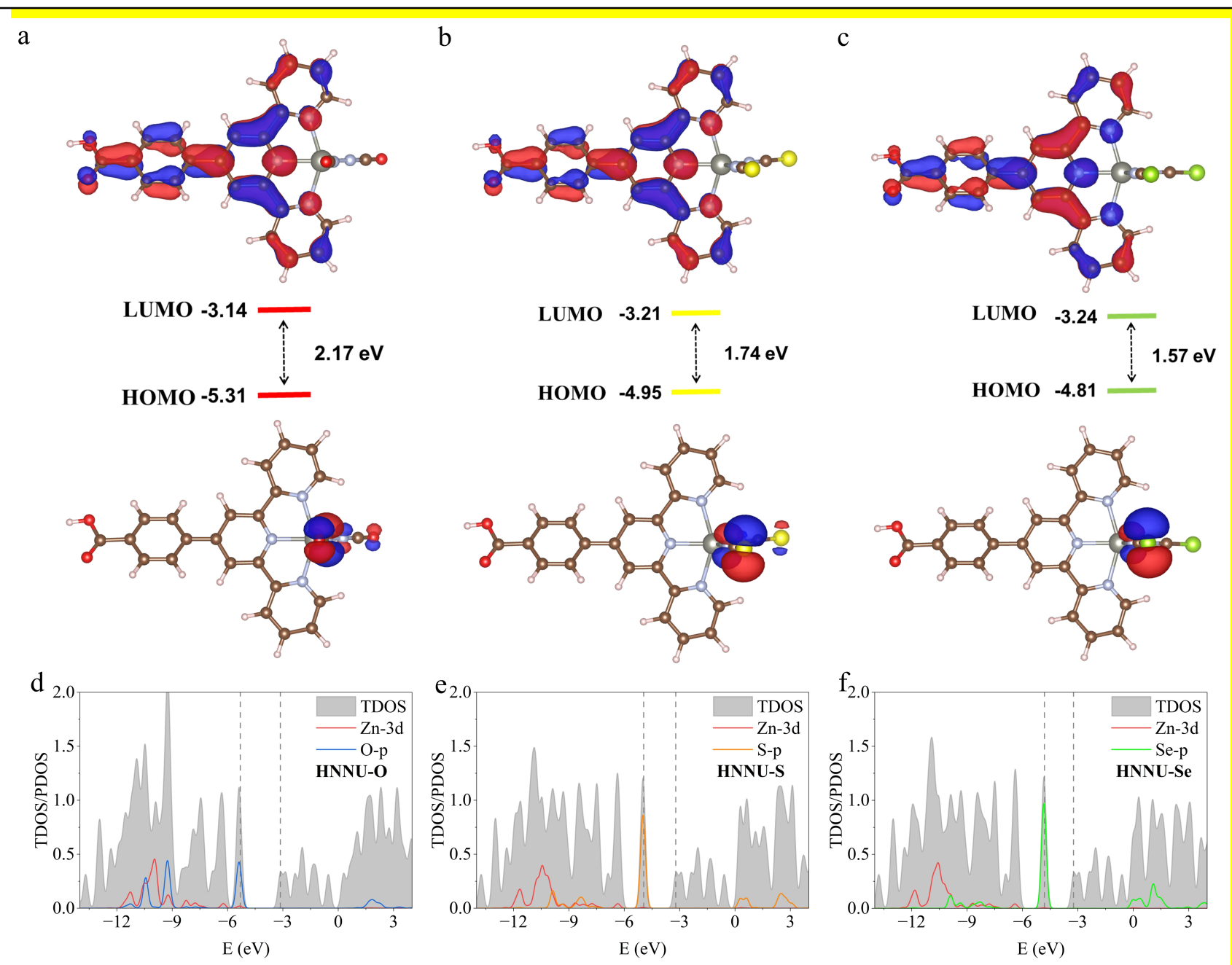
14	254	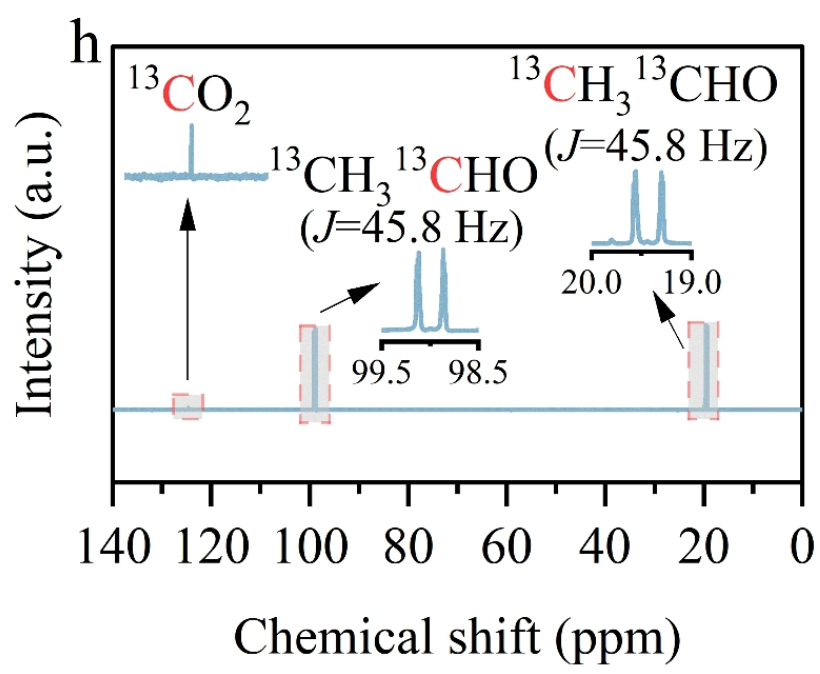

16	286	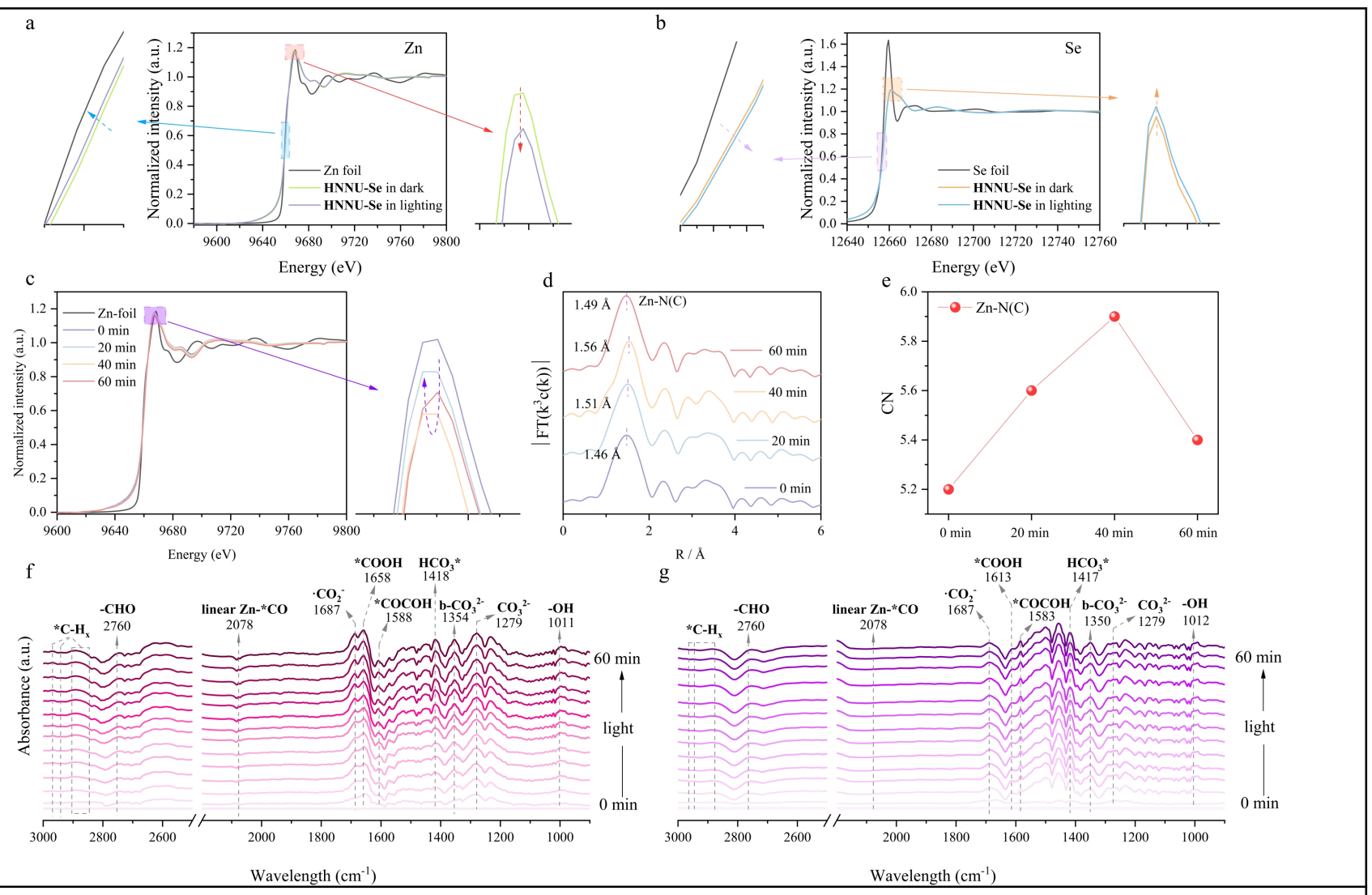
18	324-330	All the computations have been performed by the Density functional theory (DFT)[54] within the def2-SV(P)[55] basis and B3LYP[56] functional using Gaussian 16 software.[57] All calculations used the DFT-D3[58] method to describe weak interactions within the system. To account for the solvation effects, all geometry optimizations and frequency calculations were performed with the IEF-PCM model using water as solvent.[59-61] For all calculations of Gibbs free energy changes associated with proton-coupled electron transfer (PCET) steps, we used the computational hydrogen electrode model proposed by Nørskov et al.[62] All electronic property analyses were conducted by processing the fchk files using Multiwfn.[63,64]
32	652-671	54 Hohenberg, P. & Kohn, W. Inhomogeneous electron gas. *PhRv* **136**, B864-B871 (1964).<br>55 Weigend, F. & Ahlrichs, R. Balanced basis sets of split valence, triple zeta valence and quadruple zeta valence quality for H to Rn: Design and assessment of accuracy. *Phys. Chem. Chem. Phys.* **7**, 3297-3305 (2005)<br>56 Stephens, P. J., Devlin, F. J., Chabalowski, C. F. & Frisch, M. J. Ab Initio calculation of vibrational absorption and circular dichroism spectra using density functional force fields. *J. Chem. Phys.* **98**, 11623-11627 (1994).<br>57 Gaussian 16 Rev. C.01 (Wallingford, CT, 2016).<br>58 Grimme, S., Antony, J., Ehrlich, S. & Krieg, H. A consistent and accurate ab initio parametrization of density functional dispersion correction (DFT-D) for the 94 elements H-Pu. *J. Chem. Phys.* **132** (2010).<br>59 Cancès, E., Mennucci, B. & Tomasi, J. A new integral equation formalism for the polarizable continuum model: Theoretical background and applications to isotropic and anisotropic dielectrics. *J. Chem. Phys.* **107**, 3032-3041 (1997).<br>60 Miertuš, S., Scrocco, E. & Tomasi, J. Electrostatic interaction of a solute with a continuum. A direct utilizaion of AB initio molecular

		potentials for the prevision of solvent effects. *Chem. Phys.* **55**, 117-129 (1981).<br>61 Cossi, M., Barone, V., Cammi, R. & Tomasi, J. Ab initio study of solvated molecules: a new implementation of the polarizable continuum model. *Chem. Phys. Lett.* **255**, 327-335 (1996).<br>62 Kulkarni, A., Siahrostami, S., Patel, A. & Nørskov, J. K. Understanding catalytic activity trends in the oxygen reduction reaction. *Chem. Rev.* **118**, 2302-2312 (2018).<br>63 Lu, T. & Chen, F. Multiwfn: A multifunctional wavefunction analyzer. *J. Comput. Chem.* **33**, 580-592 (2012).<br>64 Lu, T. A comprehensive electron wavefunction analysis toolbox for chemists, Multiwfn. *J. Chem. Phys.* **161** (2024).

Response to reviewer #1's comment

Dear reviewer **#1**,

Thanks for your good suggestions to revise our manuscript (NCOMMS-25-099726B). We have revised this manuscript carefully according to your comment.

Reviewers #1:

1. The Reviewers **#1**: The authors have solved my last concerns about the ^{13}C NMR spectrum of the double ^{13}C labeled acetaldehyde. Given the importance of forming acetaldehyde from ambinet CO_2, I would recommend, however, to include also the ^{1}H NMR spectrum of this double labelled acetaldehyde. Apart from this addition that can be made in the proof correction stage, publication is now recommended.

Thanks for your good suggestion. We have added the ^{1}H NMR spectrum of the double ^{13}C-labelled acetaldehyde (Fig. R1) in revised manuscript (Fig. 6g).

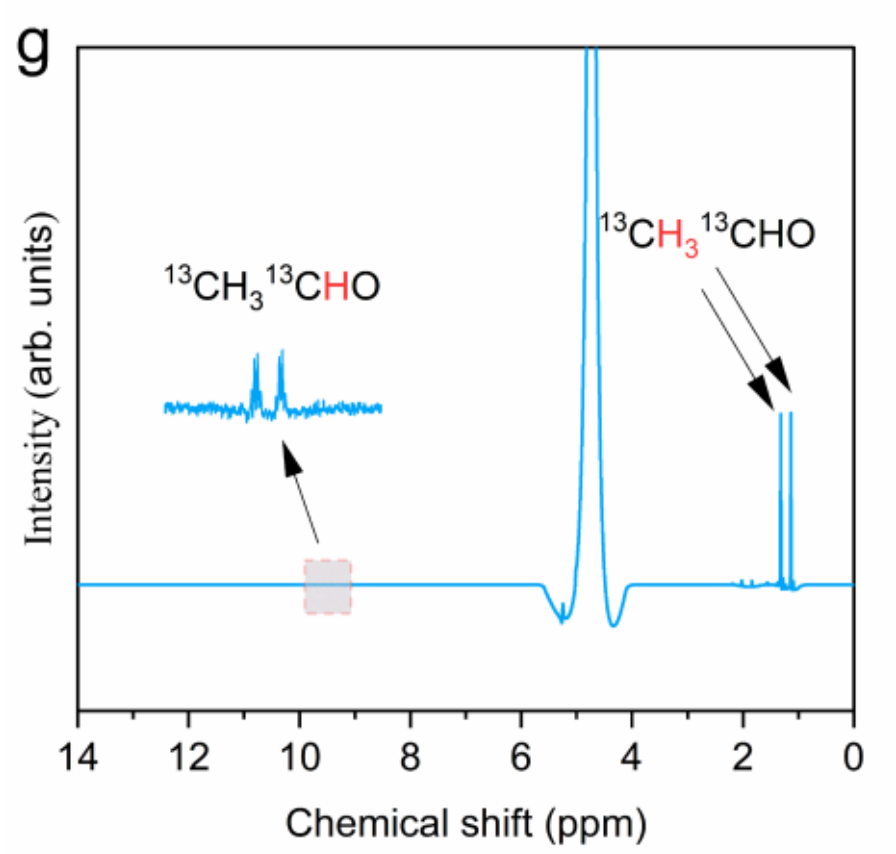

Fig. R1. ^{1}H NMR of the liquid products from $^{13}CO_2$ photoreduction over HNNU-Se.
